# Supplementary material for: Celf4 controls mRNA translation underlying synaptic development in the prenatal mammalian neocortex
Source: Nat Commun. 2023 Sep 27;14:6025. doi: 10.1038/s41467-023-41730-8 (PMC10533865; doi:10.1038/s41467-023-41730-8)
Supplement: Supplementary file 1 — Supplementary Information [file 41467_2023_41730_MOESM1_ESM.pdf]

# Supplementary Figures

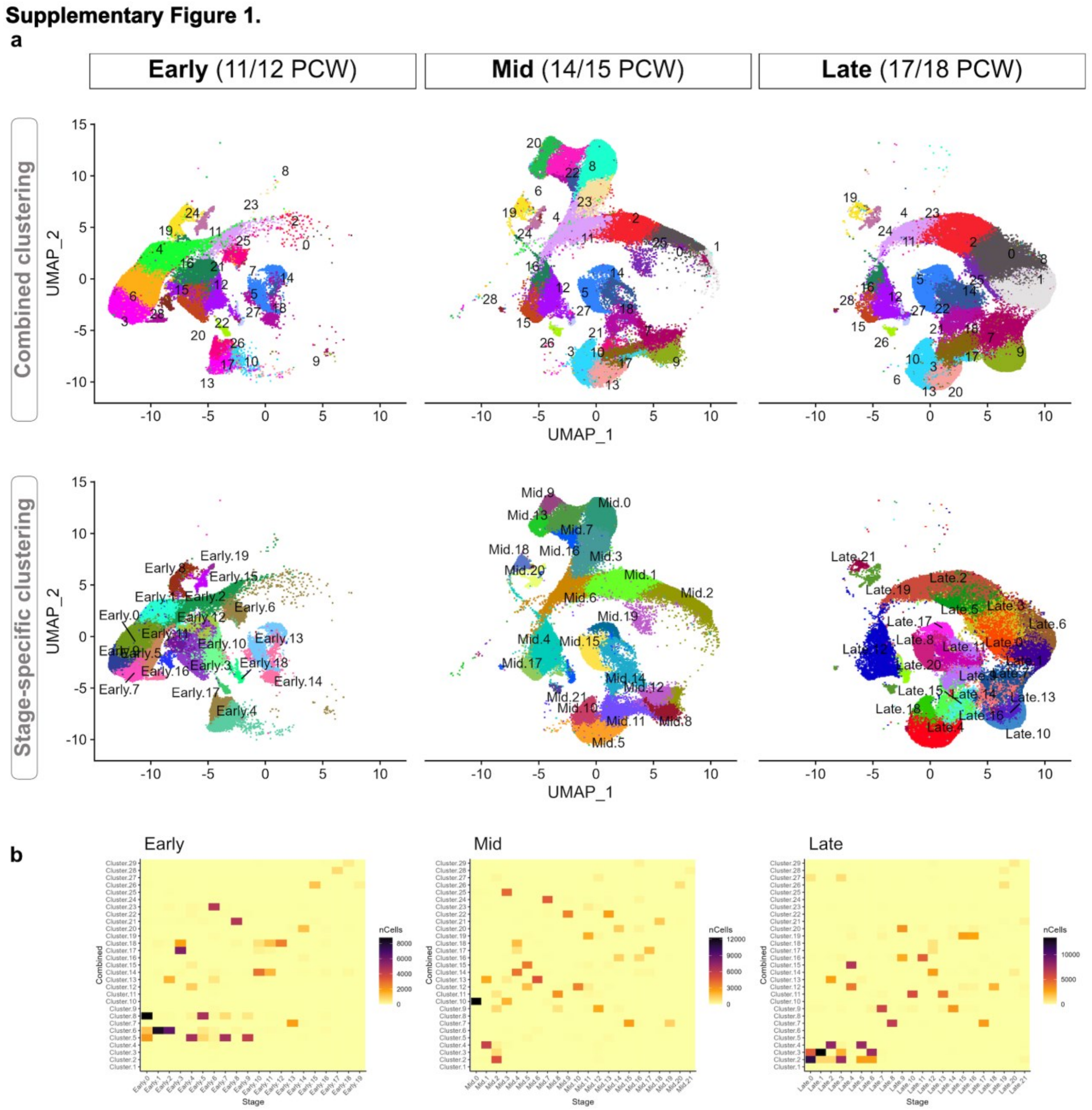


**Supplementary figure 1. UMAP visualization of integrated or phase-specific clustering of nuclei from human neocortical sample across three developmental phases of gestation.**

**(a)** UMAP projections, split by phase, showing clustering of the integrated samples identified 29 clusters (**top**), while developmental phase-specific clustering (**bottom**) identified 19 early-, 21-mid and 21 late-specific clusters (n= 2 neocortices per each developmental phase). Nuclei within each developmental group (early, mid, or late) are colored by SNN cluster assignment (using Seurat FindClusters).

**(b)** Heatmaps showing the numbers of cells from each phase-specific cluster intersecting clusters of the integrated analysis. For example, early cluster 0 from phase-specific analysis includes the

cells from combined early clusters 5, 6, and 8. Nuclei from the combined early cluster 5 can be found in early phase-specific clusters 0, 4, 5, 6, 7, and 9.

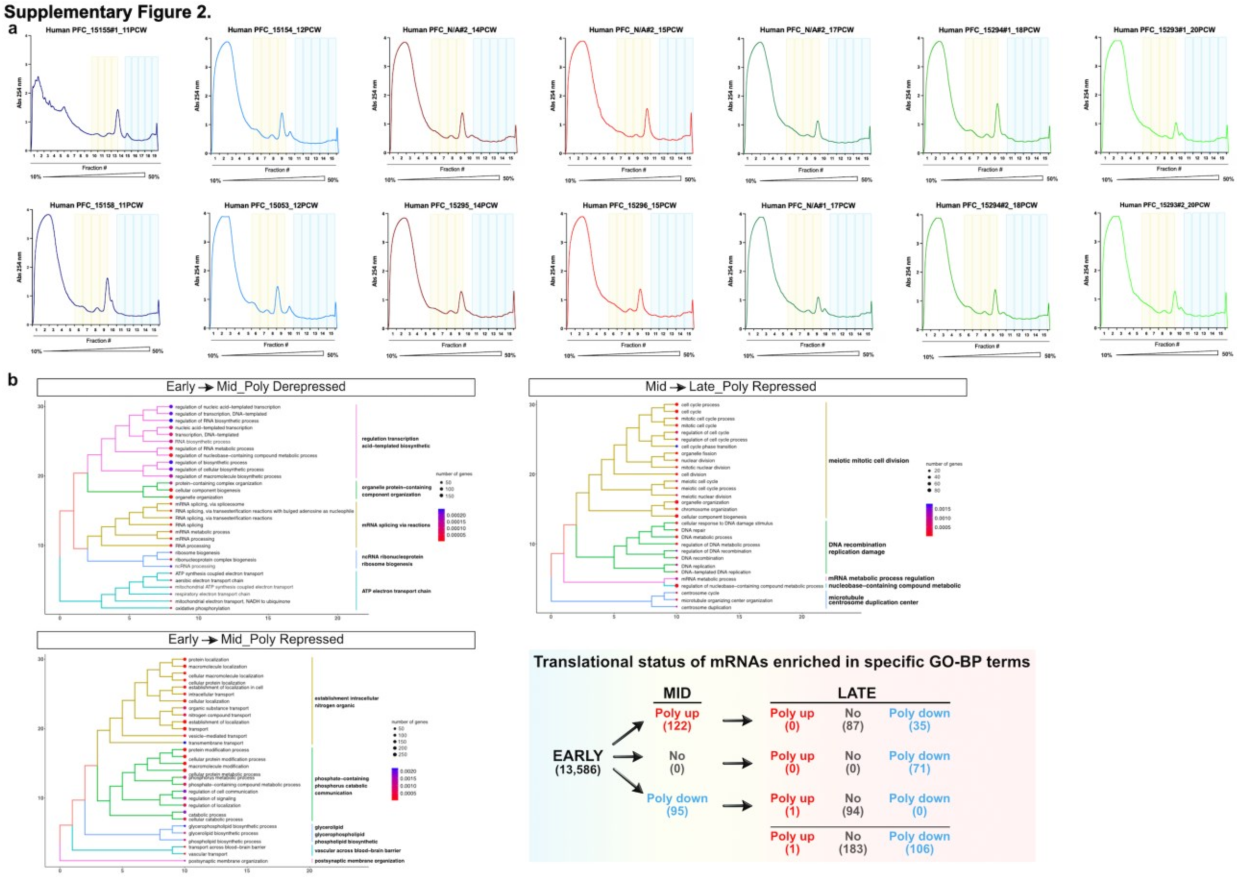


**Supplementary figure 2. Polysome profiling captures active translation in human fetal neocortices.**

**(a)** Individual human polysome profiles for each postconceptional week (11-20 PCW) using a linear 10% to 50% sucrose gradient were run in duplicates. Fractions pooled together for isolation of 40S–60S–80S are marked in yellow, and fractions combined for isolation of polysome cytoplasmic components are marked in blue.

**(b)** The tree plots made with the clusterProfiler package, showing hierarchies of gene ontology (GO)-biological process (BP) terms for the steady-state mRNAs that were highly enriched (derepressed) or significantly depleted (repressed) in the polysome fractions between early-to-mid (**left up** and **down**) and mid-to-late (**right up**) (Supplementary Data 4). Mid-to-late group of mRNAs that were derepressed in the polysome fractions (not shown) were significantly enriched only for GO-BP term "regulation of biological quality". The significance criterion is set at an adjusted p value < 0.05 with enrichment calculated using the hypergeometric p-value adjusted for multiple measurements using Benjamini-Hochberg (p.adjust). GO terms are hierarchically clustered and shown by their "parent" terms, along with p-values (dot color) and numbers of genes (diameter of dot). Summary table (**right bottom**) shows the change of GO-BP terms according to the polysome status of steady-state mRNAs during development.

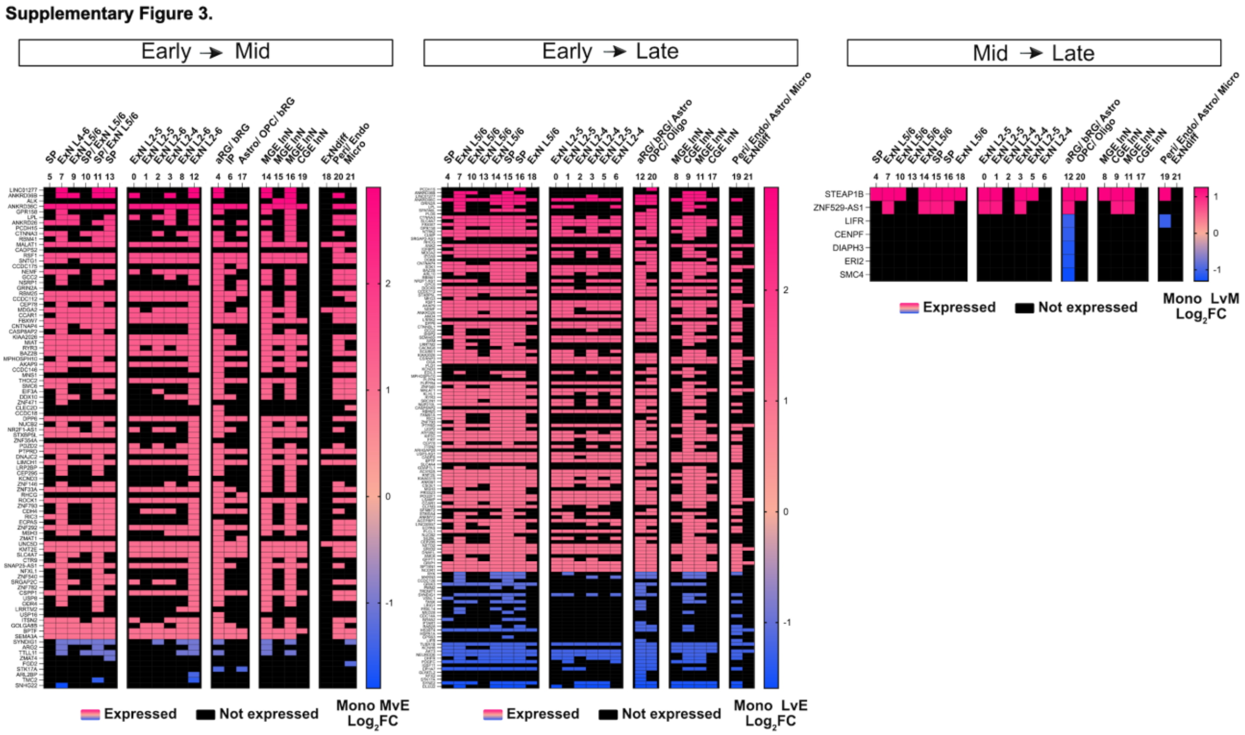


**Supplementary figure 3. Translational changes of monosome-associated transcripts across neocortical development.**

Heatmaps showing the translational changes of monosome-associated mRNAs with comparable levels of expression in the input in each cell type at mid- (left) or late-phase (mid and right) snRNAseq clusters. The criteria used in Mono MvE (left), Mono LvE (middle), or Mono LvM (right) comparisons were $|log_2FC| > 1$ and adjusted p-value $< 0.05$. The minimum mean expression threshold by snRNAseq clusters was set to $> 0.1$ with at least 30% of cells in a cluster expressing the gene. $Log_2FC$ depicted using a color scale.

**Supplementary Figure 4.**

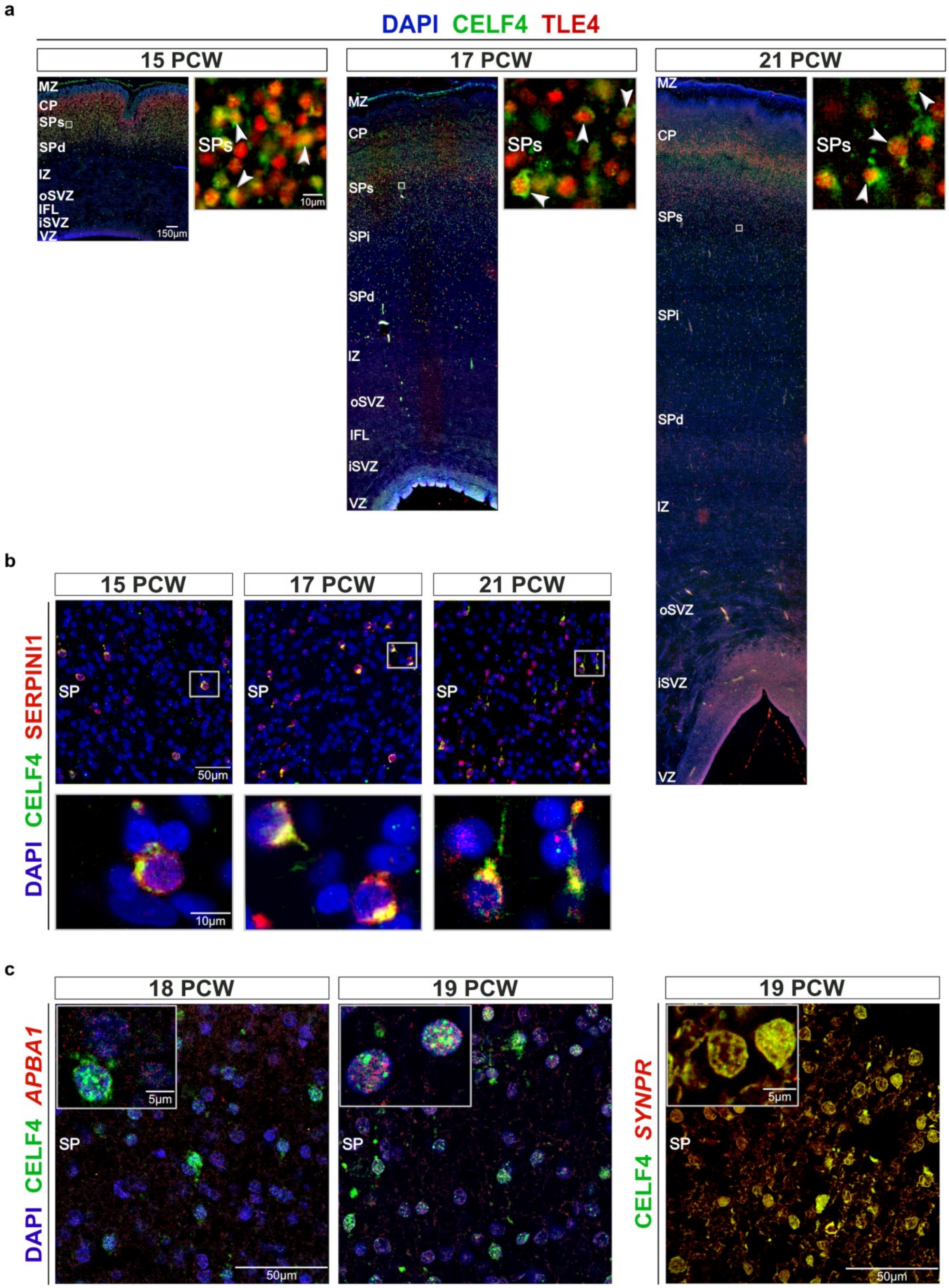

**Supplementary figure 4. RNA-binding protein CELF4 co-localizes with deep layer and SP markers and presynaptic target mRNAs in the subplate of developing neocortex.**

**(a)** Panoramic view of fetal prospective dorsolateral prefrontal neocortex at 10, 15, 17 and 21 post-conceptional weeks (PCW) showing colocalization between CELF4 (green) and TLE4 (red) in the deep cortical layers and the subplate (SP) area. DAPI is in blue. Enlarged insets (right) of the grey boxes in the full-sized images (left) show a high magnification view of CELF4+TLE4+ neurons (arrowheads) in the SP. Scale bar of 10x objective lens: 150 µm; scale bar of 40x objective lens; 10 um (inset). MZ, marginal zone. CP, cortical plate. PSP, presubplate. SPs, superficial subplate. SPd, deep subplate. SPi, intermediate subplate. IZ, intermediate zone. oSVZ, outer subventricular zone. IFL, inner fibrillar layer. iSVZ, inner subventricular zone. VZ, ventricular zone.

**(b)** Representative coronal images of 15 PCW, 17 PCW and 21 PCW human fetal cortical sections immunostained for CELF4 (green) and SERPINI1 (red). DAPI staining is in blue. CELF4 protein is coexpressed with the subset of SERPINI1+ neurons in the SP area (insets) throughout human neocortical development. Scale bar of 40x objective lens: 50 µm; scale bar of 80x objective lens: 10µm (insets).

**(c)** Representative confocal images of coronal human fetal cortical sections showing fluorescence labeling for CELF4 immunoreactivity (green) and *in situ* hybridization signal for *APBA1* mRNA (red) at 18 PCW (right) and 19 PCW (middle), as well as *SYNPR* mRNA (red). *APBA1* and *SYNPR* mRNAs were identified as CELF4-binding targets via RIP-RNAseq screen, and further validated by qRT-PCR. DAPI staining showed in blue. Scale bar of 60x objective lens: 50 µm or 5 µm (inset).

**Supplementary Figure 5.**

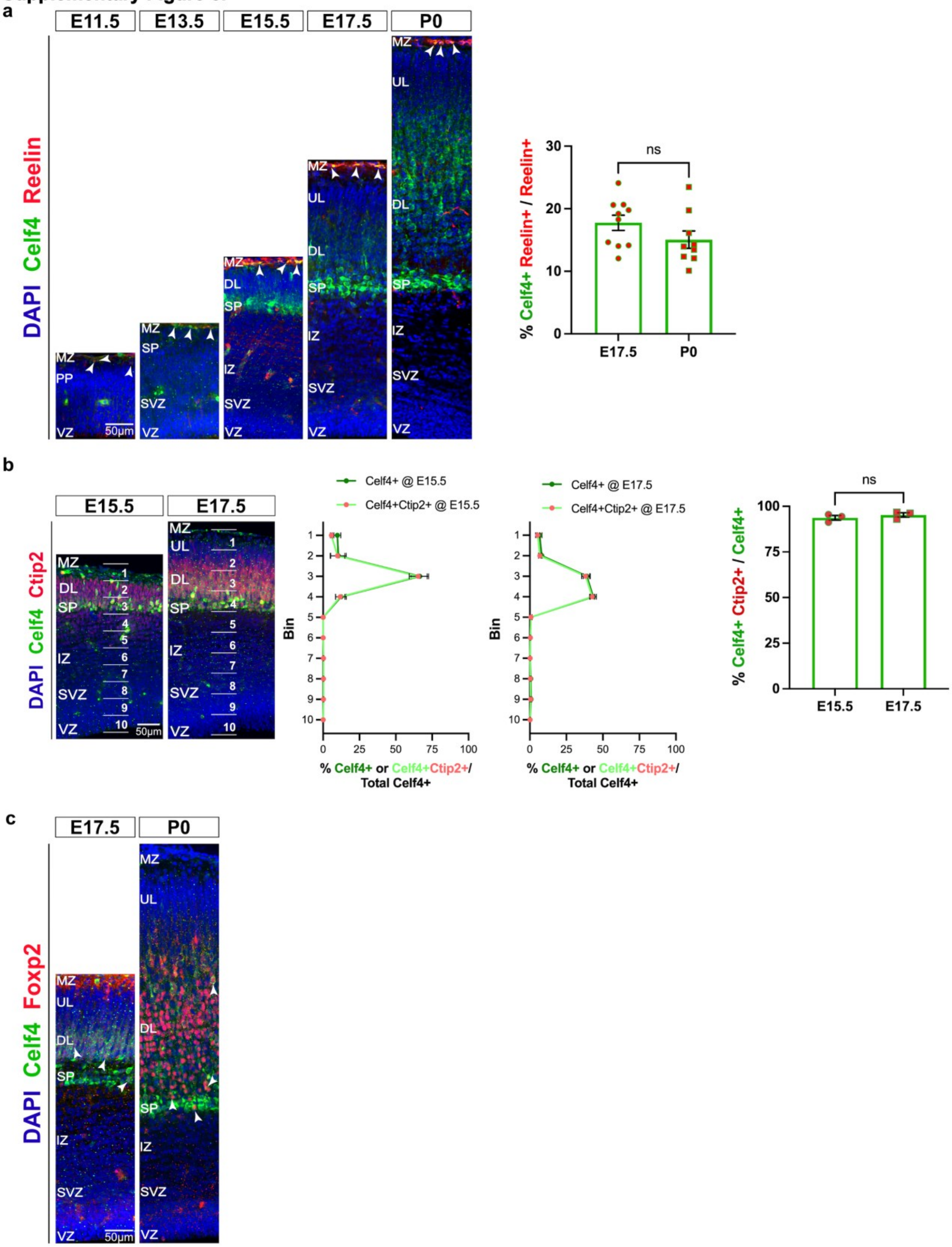


**Supplementary figure 5. Celf4 localization is associated with the marginal zone and the subplate during neocortical development.**

**(a) Left**: Celf4 (green) and Reelin protein expression patterns (red, the established marker of Cajal-Retzius cells) in the coronal sections of the E11.5, E13.5, E15.5 and E17.5 and P0 wild-type mouse neocortices. Arrowheads point out the co-localization of Celf4 with Reelin in the marginal zone (MZ). Sample size: n= 3 animals per developmental stage. **Right**: Quantification of the proportion of Celf4+Reelin+ cells from total labeled Reelin+ cells in developing neocortex at E17.5 and P0. Data represent the mean ±SEM. Statistical significance was determined by unpaired, two-tailed Welch's t-test (ns $p = 0.1640 > 0.05$).
**(b) Left and middle**: Quantification of the distribution of Celf4+ (dark green) and Celf4+Ctip2+ (light green) in E15.5 and E17.5 neocortex determined as a percentage of total number of Celf4+ cells. The grid was divided into 10 bins of equal height from pia (above MZ) (bin 1) to ventricular zone (bin 10). Sample size: n= 3 animals per developmental stage. Statistics: unpaired, two-tailed Welch's t-test (ns $p > 0.05$) for each bin separately. **Right**: Quantification of the proportion of Celf4+Ctip2+ cells from total labeled Celf4+ cells in developing neocortex at E15.5 and E17.5. Statistical significance was determined by unpaired, two-tailed Welch's t-test (ns $p > 0.05$). Data represent the mean ±SEM.
**(c)** Representative coronal images showing colocalization of Celf4 (green) with FoxP2 (red) in wild-type neocortices at E17.5 and P0. FoxP2 protein expression is mostly restricted to layer 6 cortical neurons. Arrowheads indicate subplate and layer 6 neurons that co-express Celf4 and Foxp2 proteins. DAPI label is in blue. Scale bar of 20x objective lens: 50 µm. PP, preplate. VZ, ventricular zone. SVZ, subventricular zone. IZ, intermediate zone. DL, deep layer. UL, upper layer.

**Supplementary Figure 6.**

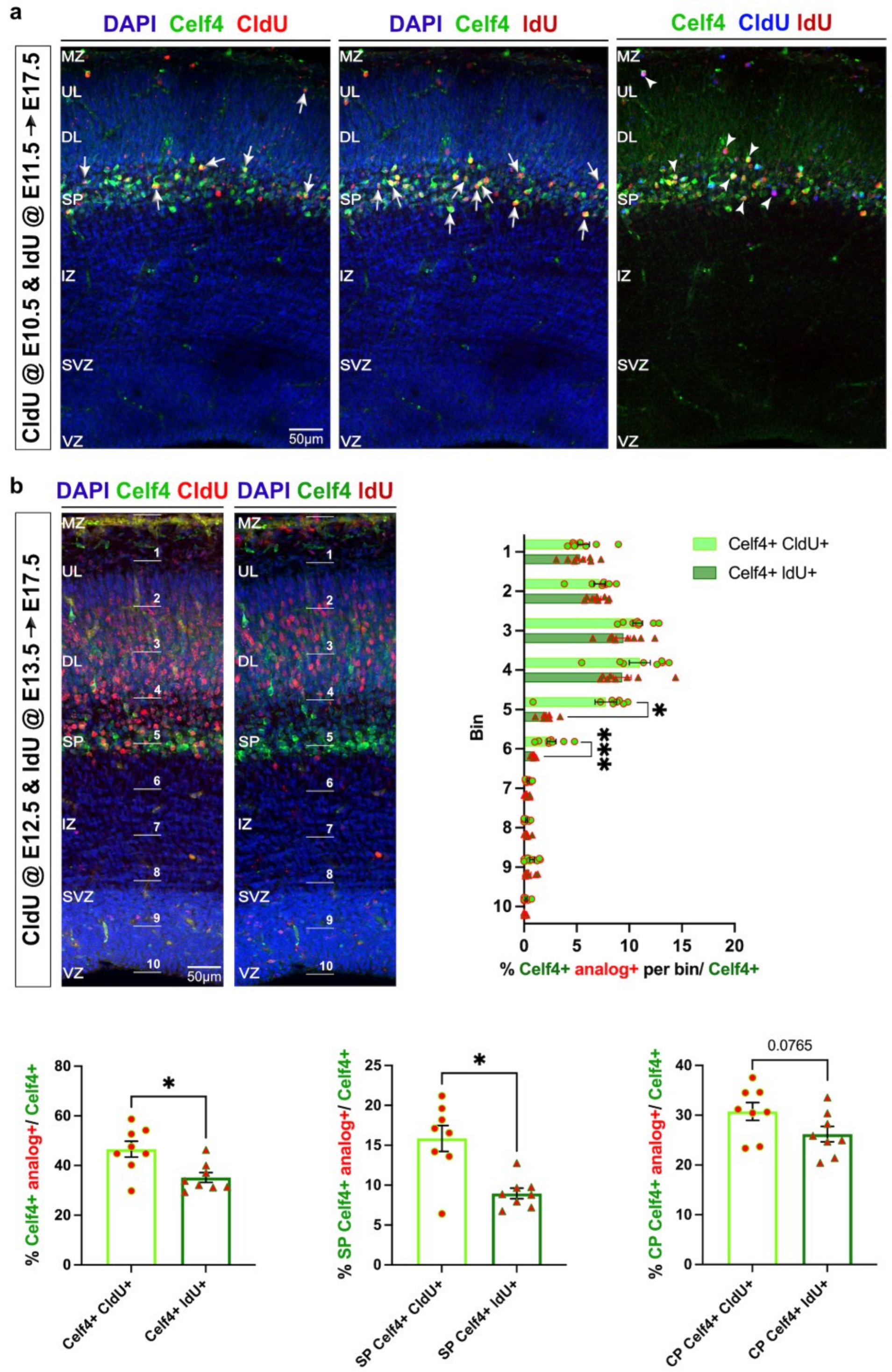


**Supplementary figure 6. Celf4-expressing neurons are one of the earliest-born neurons in the developing neocortex.**

**(a)** Representative confocal images of neuronal birth-date analysis of Celf4-positive (+) neurons (green) at E17.5 mouse neocortex using nuclei stain DAPI (blue), anti-CldU and anti-IdU

antibodies co-staining after single pulse injections of CldU on E10.5 (red, 24 hours before embryo collection; left) and IdU on E11.5 (red, 23 hours after the CldU injection and 1 hour before collection; middle). Some Celf4+CldU+ neurons (arrows, left) exited cell cycle and appeared in the subplate (SP) at E17.5. More Celf4+IdU+ neurons (arrows, middle) that were cycling at 24 hours but were not in S-phase at the time of CldU injection were detected in the SP. Celf4+CldU+IdU+ SP neurons (arrowheads, right) re-entered cell cycle and were detected in SP and marginal zone (MZ) at E17.5. Sample size: n= 2 animals.

**(b) Top**: distribution of thymidine analogs+ nuclei in mouse neocortex at E17.5 (left). Celf4+ neurons (green) born at E12.5 were labeled by injection of CldU (red; left) while the ones born at E13.5 were labeled by IdU injection (red; right). Graphical representation of Celf4+CldU+ (light green) and Celf4+IdU+ (dark green) distribution in E17.5 neocortex across 10 equally-sized bins from MZ (bin 1) to ventricular zone (VZ, bin 10; right). Double-labeled cells in each bin are given as the percentage of Celf4+analogue+ cells from total Celf4+ cells. Statistics: unpaired, two-tailed Welch's t-test (parametric analysis), or Mann-Whitney test (non-parametric analysis). *$p \leq 0.05$, ***$p \leq 0.001$. Data represent mean ±SEM for each bin across 4 sections per brain; n= 2 brains per developmental stage. **Bottom**: the bar graphs show the quantification of co-localization of thymidine analogs+ cells with all Celf4+ neurons in the neocortex (left), Celf4+ neurons only in the SP area (SP Celf4+, middle), or Celf4+ neurons only in the cortical plate (CP Celf4+, right). Statistics: unpaired, two-tailed Welch's t-test for parametric analysis, or Mann-Whitney test for non-parametric analysis. *$p \leq 0.05$, ***$p \leq 0.001$. Data represent mean ±SEM. P-values are shown in Source data. Scale bar of 20x objective lens: 50µm. SVZ, subventricular zone. IZ, intermediate zone. DL, deep layer. UL, upper layer.

**Supplementary Figure 7.**

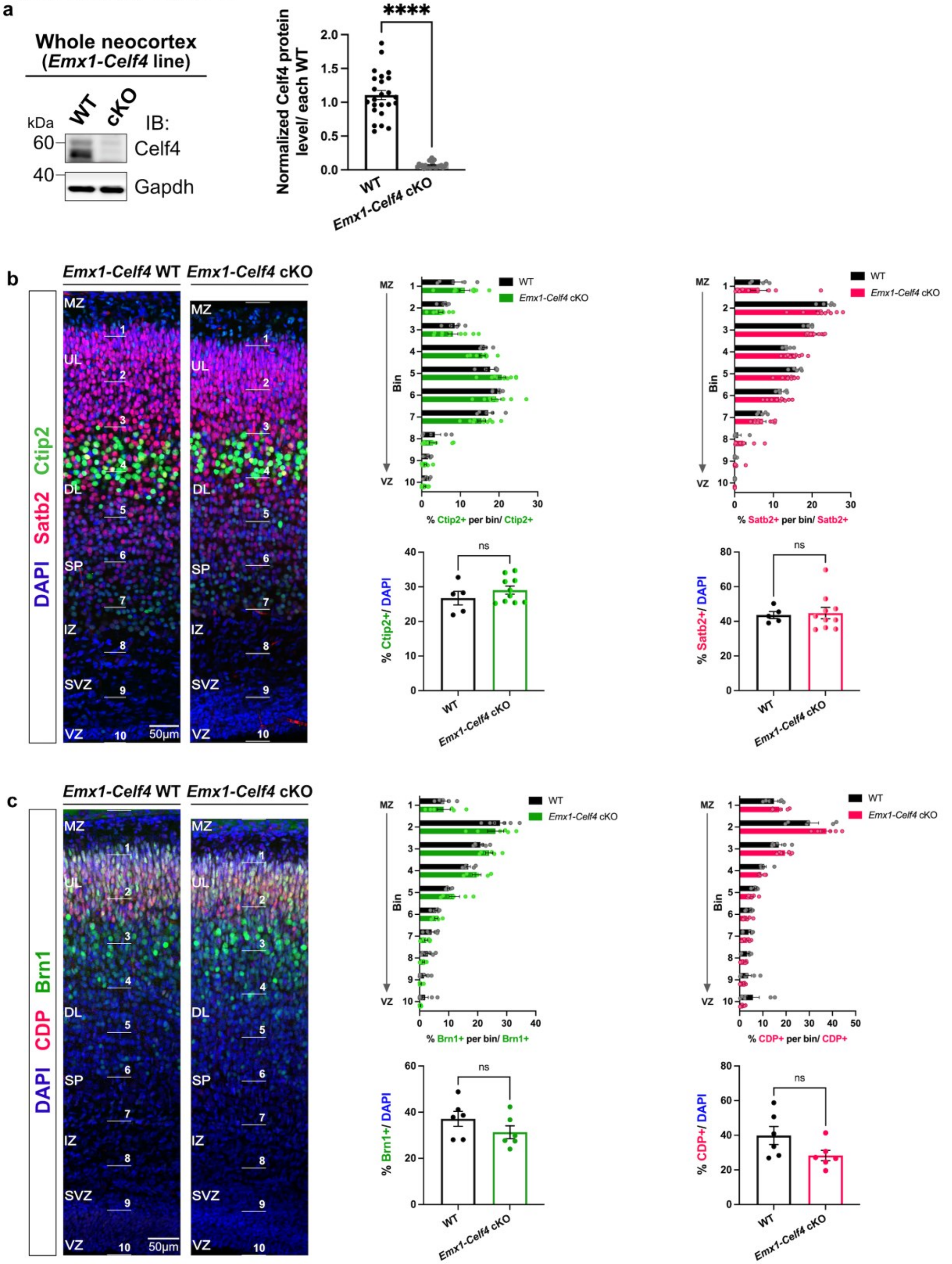


**Supplementary figure 7. Selective ablation of *Celf4* by E10.5 from dorsal telencephalon does not affect neuronal migration and laminar fate decision at P0.**

**(a)** Western blot analysis of total neocortical lysates collected from WT and *Celf4* knock-out mice (*Emx1-Celf4* cKO) at P0 (**left**). The presence of a Celf4 band indicated WT while the absence of a band indicated successful deletion of *Celf4*. The densitometry analysis of relative Celf4 protein levels normalized to the loading control Gapdh (**right**). Sample size: n= 6 animals for each sex and genotype. The graph (both sexes included) was generated by plotting averaged values from individual Celf4 image that was normalized to several Gapdh exposure times. Data represent the mean ±SEM. Statistics: non-parametric unpaired, two-tailed Mann-Whitney test. ****$p \leq 0.0001$. **(b)** Representative confocal images of neocortical sections coimmunostained for upper-layer intracortical (callosal) projection neuron marker Satb2 (red) and deep layer (primarily layer 5) marker Bcl11b/Ctip2 (green) [n= 3 WT animals, n= 4 *Emx1-Celf4* cKO animals], or **(c)** upper layer markers CDP (red) and Pou3f3/Brn1 (green) [n= 3 animals per genotype] in the *Emx1-Celf4* cKO and their control littermates at P0. The number of each layer-specific marker was quantified from pia to ventricular zone (VZ) throughout 10 equally sized bins, based on the total number of marker-positive (+) cells (horizontal bar graphs). Total number of marker-positive (+) cells in the neocortex given as a percentage from total DAPI+ cells (vertical bar graphs). No significant differences were found. Data represent mean of sections ± SEM. Statistics: unpaired, two-tailed Welch's t-test or Mann-Whitney test per each bin and for total percentage of layer-marker + cells. P-values are shown in Source data. Scale bar of 20x objective lens: 50µm. VZ, ventricular zone. SVZ, subventricular zone. IZ, intermediate Zone. SP, subplate. DL, deep-layer. UL, upper-layer. MZ, marginal zone.

**Supplementary Figure 8.**

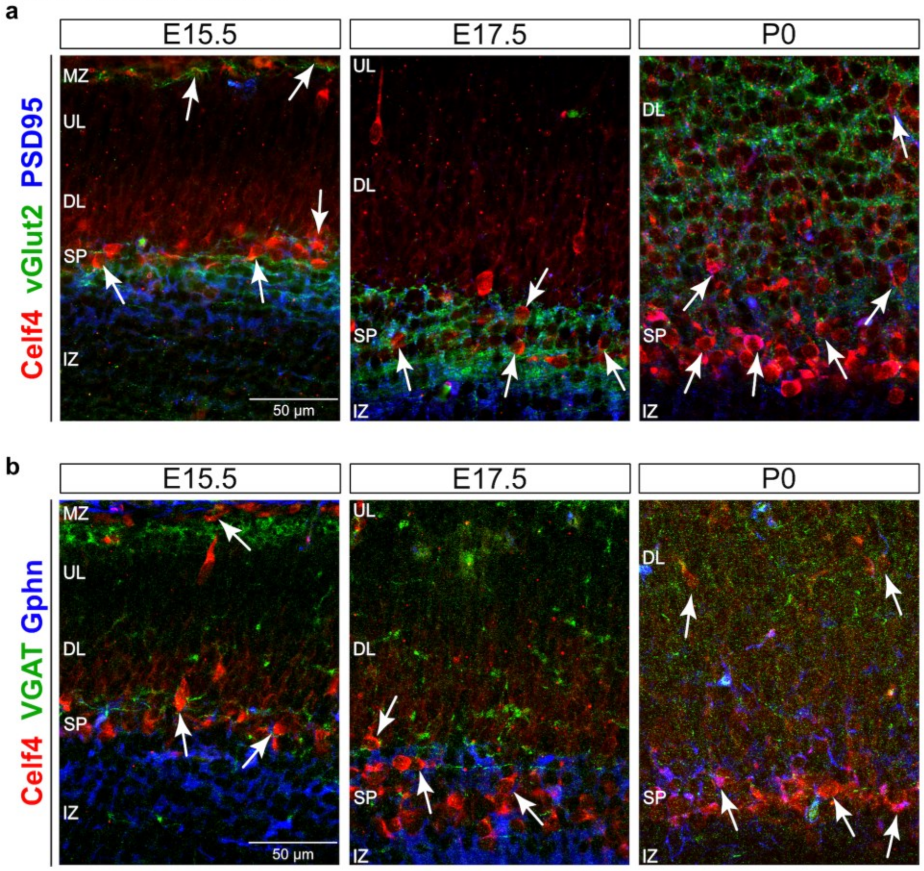


**Supplementary figure 8. Celf4-positive neurons express pre- and post-synaptic markers of thalamo-cortical glutamatergic and GABAergic synapses during neocortical development.**
**(a)** When thalamic fibers arrive at the cortex at E15.5 and start to accumulate in the subplate (SP), the Celf4-positive (+) neurons (red) co-localize with thalamo-cortical synapses identified by pre-synaptic marker vGlut2 (green) and post-synaptic marker PSD95 (blue) in the marginal zone (MZ) and the SP (arrows). In the developing cortex between E17.5 and P0, the thalamocortical fibers reach the cortical plate (layer 5 and 6) by starting their radial growth into the cortex. At these developmental time points, the Celf4+ SP neurons show more prominent co-localization with vGlut2+PSD95+ puncta (arrows). At P0, Celf4+ neurons in the deep cortical layers show overlapping signal with vGlut2 and PSD95 (arrows).
**(b)** Representative confocal images at E15.5, E17.5 and P0 showing co-localization of Celf4+ neurons (red) with pre-synaptic marker VGAT (green) and post-synaptic marker gephyrin (Gphn, blue) that characterize developing GABAergic synapses in the prenatal neocortex. At E15.5 and E17.5, the MZ and SP layer are rich in Gphn, while VGAT protein expression shows higher signal in the MZ and relatively low yet detectable signal in the SP. During these developmental stages, the Celf4 protein expression was co-localized with VGAT and Gphn punctate expression patterns in the SP (arrows). As VGAT protein expression becomes more prominent in the SP and deep cortical layers at P0, higher number of Celf4+ SP neurons together with Celf4+ neurons in the deep cortical layers show co-localization with both pre- and post-synaptic GABAergic markers

(arrows). Sample size: n = 4 animals per developmental stage. Scale bar of 60x objective lens: 50µm. VZ, ventricular zone. SVZ, subventricular zone. IZ, intermediate Zone. DL, deep-layer. UL, upper-layer.

Supplementary Figure 9.

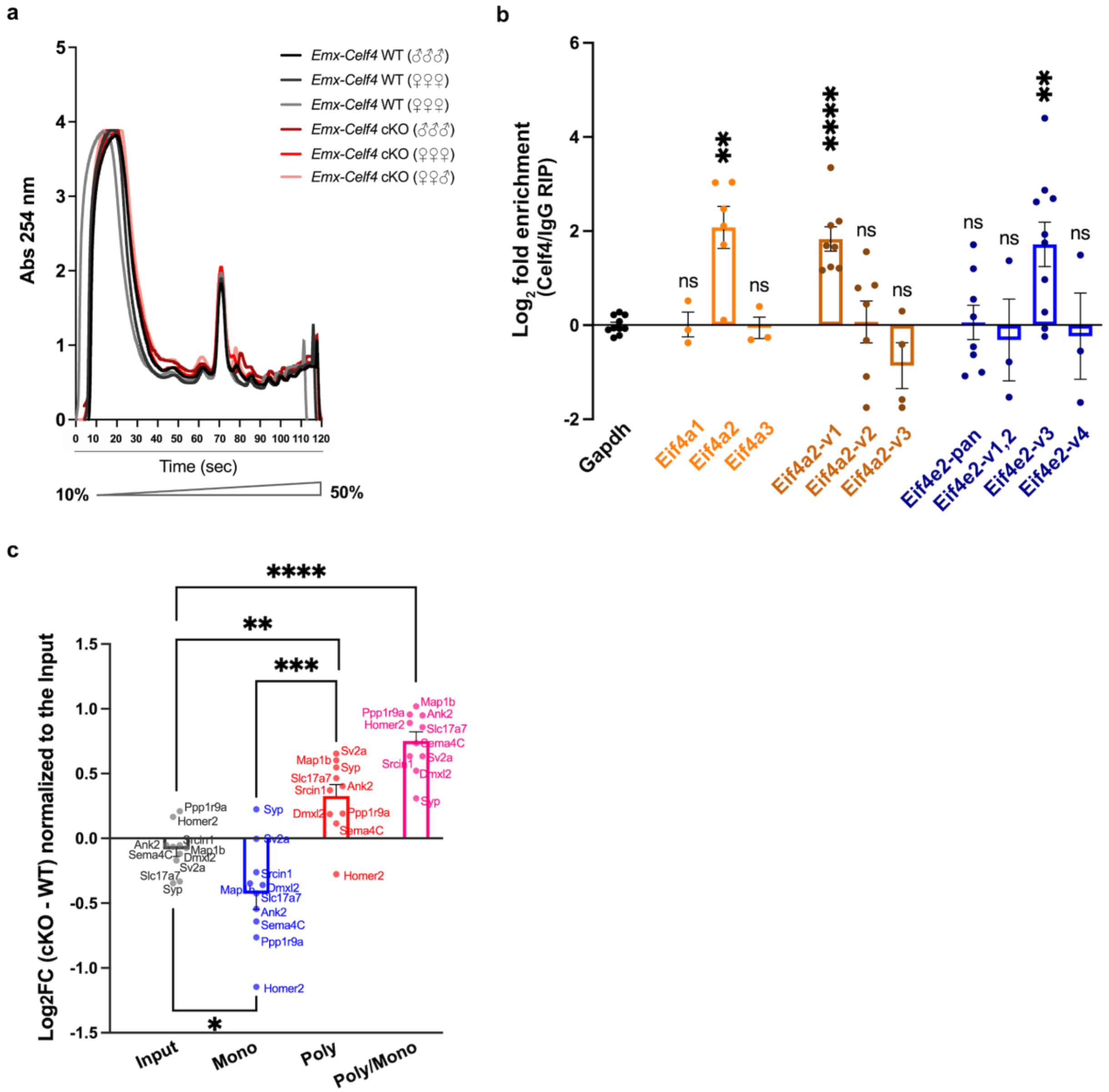


**Supplementary figure 9. Celf4 binds to and regulates the expression of synapse-associated mRNAs at the level of translation.**

**(a)** Schematic overview of polysome profiles obtained after density gradient fractionation of WT and *Emx1-Celf4* cKO neocortical lysates at P0. A254 curves are centered at onset of 80S peak. RNA was prepared from 40S-60S-80S (F6–9) and polysome fractions (F11-15), then subjected to RNAseq analysis. Sample size: n= 3 spins per genotype; biological replicates were prepared using 3 pooled neocortices with the sex indicated on in the graph legend.

**(b)** qRT-PCR validation of mouse Celf4 RIP-RNAseq data confirmed that Celf4 directly binds eukaryotic translation initiation factors *Eif4a2* and *Eif4e2* in an isoform-specific manner (*Eif4a2-v1* and *Eif4e2-v3,* respectively). Relative mRNA levels were calculated from Celf4 vs. IgG RIPs and normalized to the internal control *Gapdh*. Sample size: n= 3-5 animals. qRT-PCR run with ≥ 3 technical replicates per target isoform. Data presented as means and SEM. P-values are shown in Source data. Statistics: unpaired, two-tailed Welch's t-test (parametric analysis), or unpaired, two-tailed Mann-Whitney test (non-parametric analysis). ns $p > 0.05$, **$p \leq 0.01$, ****$p \leq 0.0001$.
**(c)** Bar graph showing the general effect of *Celf4* deletion on the translational status of the synapse-associated mRNAs. In *Emx1-Celf4* cKOs at P0, mRNA levels of these synapse-associated targets are significantly decreased in the 40S-60S-80S fractions (mono), but significantly increased in the polysome fractions (poly). The poly/mono ratio additionally supports that Celf4 drives a translational repression of specific synaptic mRNAs in WT. These mRNA levels in each fraction were first normalized to the total RNA (input) and then to the corresponding littermate control, as follows: $Log_2FC$ [(cKO-Input)/(WT-Input)]. Sample size: n= 3 spins per condition, 3 neocortices pooled together as one biological sample. Data on the bar graph represent the mean and SEM. P-values are shown in Source data. Statistics: unpaired, two-tailed Welch's t-test or Mann-Whitney test. *$p \leq 0.05$, **$p \leq 0.01$, ***$p \leq 0.001$, ****$p \leq 0.0001$.

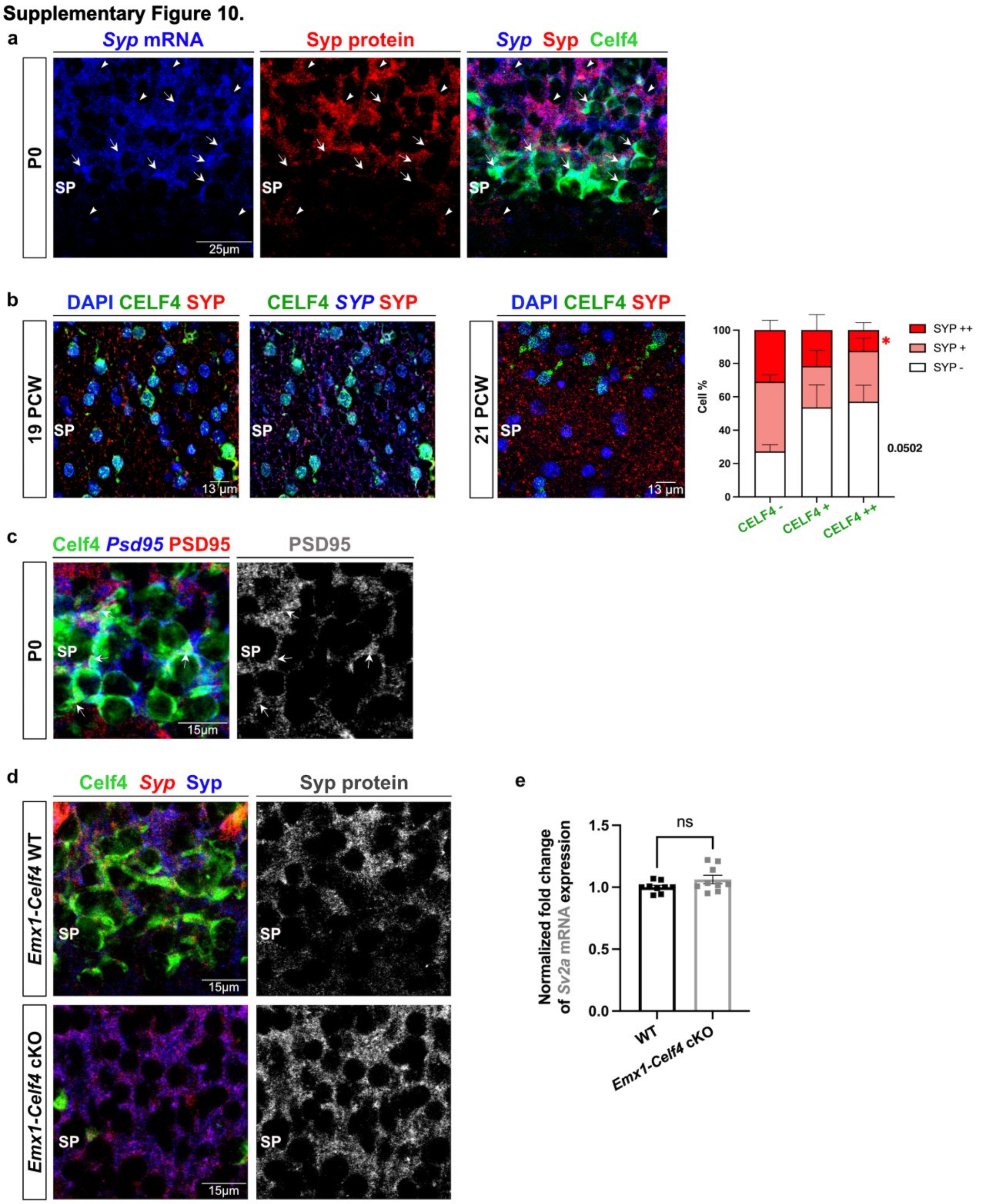


**Supplementary figure 10. Pre-synaptic target *Syp* mRNA is translationally repressed by Celf4 during cortical development.**

**(a)** At P0, immunoFISH revealed low Syp protein labeling (red) where strong Syp mRNA (blue) and Celf4 proteins (green) coexpressed (arrows) in the mouse SP. Areas with low Celf4 protein

show strong Syp mRNA and protein (arrowheads). Sample size: n = 3 male animals. Scale bar of 60x objective lens: 25µm.

**(b)** ImmunoFISH showing the expression of CELF4 protein (green), SYP protein (red) and *SYP* mRNA (blue) in the human SP at 19 PCW and 21 PCW. DAPI nuclei are in blue. Scale bar of 60x objective lens: 13 µm. The stacked graph displays the percentage of SP neuron that are CELF4- (none), CELF4+ (low expression), CELF4++ (strong expression), and their SYP punctate surroundings: SYP- (white), SYP+ (light red), SYP++ (red). The number of "CELF4++SYP-" SP neurons is higher when compared to the counted number of "CELF4-SYP-" cells ($p = 0.0502$). Sample size: n= 3-4 brain sections. Data represent the mean and SEM. P-values are shown in Source data. Statistics: unpaired, two-tailed Welch's t-test or unpaired, two-tailed Mann-Whitney test. *$p \leq 0.05$.

**(c)** Representative immunoFISH images showing extensive overlap between Celf4 protein (green) with *Dlg4/PSD95* mRNA (blue) and PSD95 protein (red and grey) in the SP of P0 neocortex (n = 3 male animals per genotype). Scale bar of 60x objective lens: 15µm.

**(d)** Celf4 represses translation of *Syp* mRNAs in the SP of P0 mouse neocortex. **Top**: immunoFISH showing colocalization of Celf4 (green) with *Syp* mRNA (red) in the SP of P0 WT, but not with Syp protein (blue and grey). **Bottom**: Syp protein expression (blue and grey) is enhanced from *Syp* mRNAs in the SP of P0 mutant (top). Sample size: n= 3 male animals per genotype. Scale bar of 60x objective lens: 15µm.

**(e)** *Sv2a* mRNA levels determined by qRT-PCR of P0 WT and *Emx1-Celf4* cKO cortices (n = 3 male animals per genotype). Data normalized to *Gapdh* and then to each WT, and plotted on the graph using the mean and SEM. Statistics: unpaired, two-tailed Mann-Whitney test non-parametric analysis. $p = 0.1351$; ns $p > 0.05$.

**Supplementary Figure 11.**

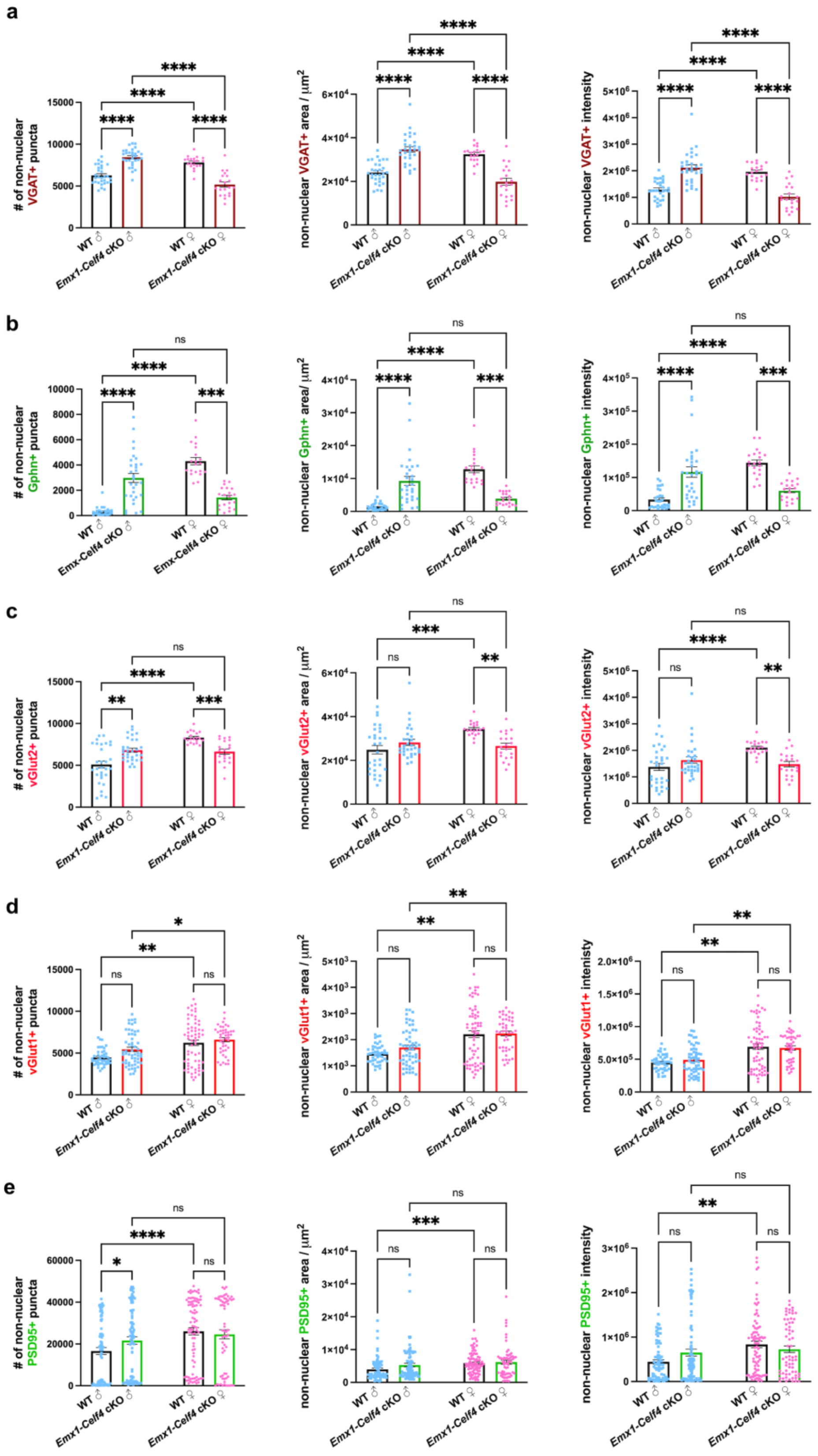

**Supplementary figure 11. Automated Cx7 Cellinsight findings indicate the sex-dependent differences in the individual synaptic markers upon *Celf4* deletion.**

**Left:** The bar graphs show Cx7 Cellinsight sex-based quantification of non-nuclear count values for individual synaptic puncta in P0 littermate control (WT) and *Emx1-Celf4* cKO, and for pre-synaptic marker VGAT+ **(a)**, post-synaptic marker Gphn+ **(b)**, pre-synaptic marker vGlut2+ **(c)**, pre-synaptic marker vGlut1+ **(d)**, and post-synaptic marker PSD95+ **(e)**.

**Middle**: Quantification of the non-nuclear surface area (µm) in male and female WT and *Emx1-Celf4* neocortices for each individual synaptic marker.

**Right:** The bar graphs display non-nuclear fluorescence intensity for individual synaptic marker separated by the sex and genotype.

Samples size: n= 3 male animals and n= 2 female animals per genotype. All images are acquired in the SP area of developing neocortices at P0. Analyzed >1000 puncta/image; processed 20-57 images per sex/genotype. Each dot represents values obtained from one cell level ± SEM. P-values are shown in Source data. Statistics for the bar graphs (a-e): no matching or pairing Brown-Forsythe and Welch ANOVA tests (parametric multiple comparisons) or Kruskal-Wallis test with Dunn's post-hoc test (non-parametric multiple comparisons). ns $p > 0.05$, *$p \leq 0.05$, **$p \leq 0.01$, ****$p \leq 0.0001$.

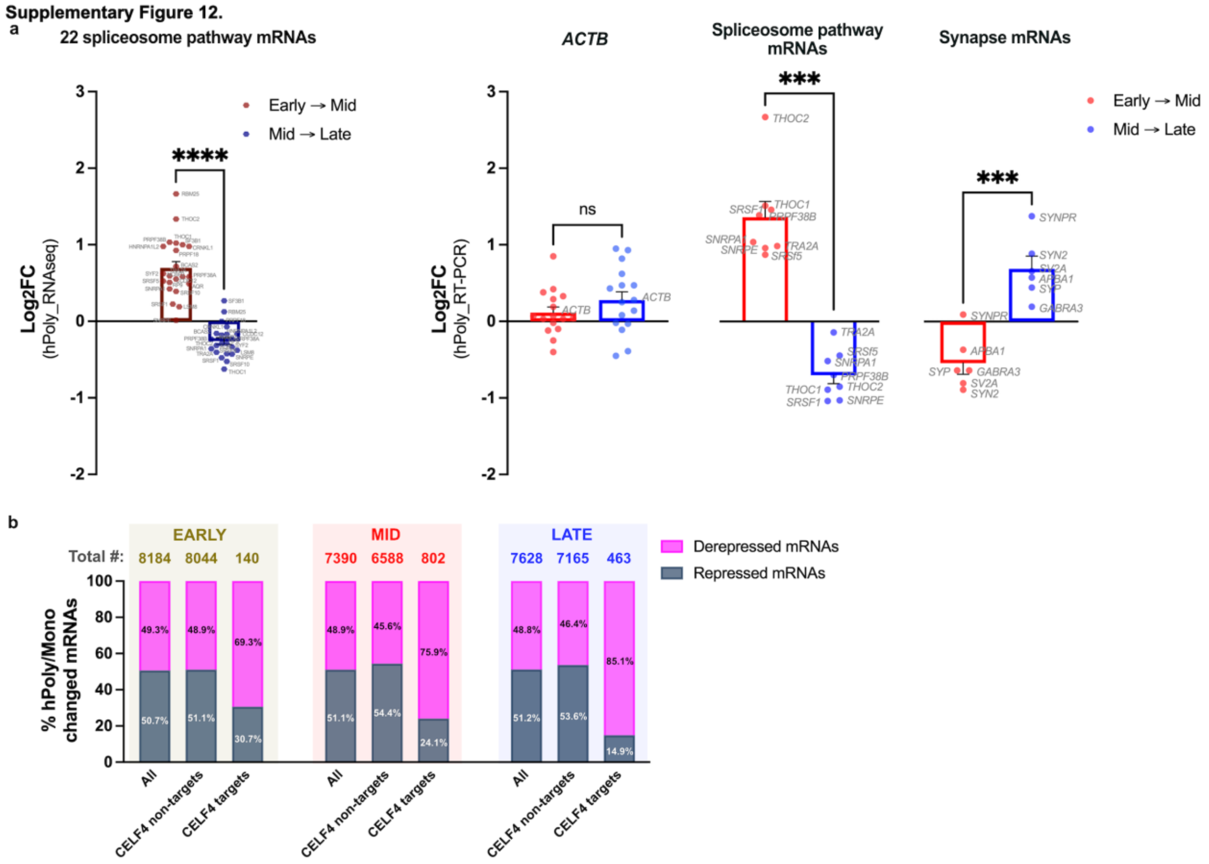


**Supplementary figure 12. Spliceosome and synapse-associated mRNAs are dynamically associated with actively translating polysomes during human neocortical development.**

**(a) Left:** bar graph shows 22 human spliceosome mRNAs significantly associated with polysome fractions in human polysome RNAseq (adjusted p value < 0.05 in KEGG pathway analysis), showing translational derepression from early-to-mid, and translational repression from mid-to-late phase. Sample size: n= 2 cortices per early and mid phases, n= 6 cortices per late phase (2 cortices per each developmental phase). Statistics: unpaired, two-tailed Mann-Whitney test. ****p≤ 0.0001. **Right:** Relative mRNA levels of spliceosome and synaptic mRNAs from early-, mid- and late-phase polysome fractions using qRT-PCR. *ACTB* mRNA levels (negative control) remained stable during human cortical development. Spliceosome mRNAs (*THOC1, SRSf5, SNRPA1, SNRPE, SRSF1, THOC2*), with two of which are also CELF4 targets (*TRA2A* and *PRPF38B*), are translationally derepressed from early-to-mid- phase but show significant repression in mid-to-late phase. CELF4-target mRNAs associated with synapse functions (*SYP, SV2A, APBA1, SYN2, GABRA3, SYNPR* in Fig. 2g) are translationally repressed in early-to-mid-phase, and derepressed in mid-to-late phase. Data were normalized to the housekeeping gene *Gapdh*. Data represent mean $Log_2FC$ ratio in relation to the earlier developmental phase (mean ± SEM). Sample size: n= 2 cortices per early and mid phases, and n= 6 cortices per late phase (2 cortices per each developmental phase); tested mRNAs were run as 4-16 replicas. P-values are shown in Source data. Statistics: either unpaired, two-tailed Welch's t-test for parametric analysis, or unpaired, two-tailed Mann-Whitney test for non-parametric analysis. ns p> 0.05, ***p≤ 0.001.

**(b)** Stacked graph shows the percentage of polysomic change (Poly/Mono ratio) of human mRNAs with adjusted p value < 0.05 identified from polysome RNAseq comparisons (from Fig. 1d & Supplementary Data 3). Human CELF4 non-target mRNAs show almost equal levels of translational derepression (pink) and repression (grey) across early, mid and late phases. CELF4 preferentially binds more actively translating target mRNAs (from Fig. 2f) that exhibit an increasing trend in translational derepression from early (69.3%) to late phase (85.1%) during human neocortical development. Total number indicates developmentally changing mRNAs used in the analysis (adjusted p value < 0.05).
