# Supplementary material for: Celf4 controls mRNA translation underlying synaptic development in the prenatal mammalian neocortex
Source: Nat Commun. 2023 Sep 27;14:6025. doi: 10.1038/s41467-023-41730-8 (PMC10533865; doi:10.1038/s41467-023-41730-8)
Supplement: Supplementary file 3 — Description of Additional Supplementary Files [file 41467_2023_41730_MOESM3_ESM.pdf]

Description of Additional Supplementary Files

File Name: Supplementary Data 1
Description: snRNAseq DEG analysis
-Tabs: EARLY (11 12 PCW), MID (14 15 PCW), LATE (17 18 PCW).

File Name: Supplementary Data 2
Description: ASD and NDD gene set from snRNAseq
-Tabs: ASD-risk genes in early clusters tab, ASD-risk genes in mid clusters, ASD-risk genes in late clusters, Enrichment of ASD-risk genes in early, mid and later clusters, DDG2P-risk genes in early clusters, DDG2P-risk genes in mid clusters, DDG2P-risk genes in late clusters, Enrichment of DDG2P-risk genes in early, mid and later clusters.

File Name: Supplementary Data 3
Description: Human polysome DEG excels (MvE, LvE, LvM)
-Tabs: List of mRNAs that change their association with monosomes MvE, List of mRNAs that change their association with Mono LvE, List of mRNAs that change their association with Mono LvM, List of mRNAs that change their association with Poly MvE, List of mRNAs that change their association with Poly LvE, List of mRNAs that change their association with Poly LvM, Polysomic change (Poly/Mono ratio) of all human mRNAs with adjusted p value < 0.05.

File Name: Supplementary Data 4
Description: Human Poly GO-BP tables
-Tabs: GO terms enriched in biological processes for derepressed mRNAs in Poly MvE, GO terms enriched in biological processes for repressed mRNAs in Poly MvE, GO term enriched in biological processes for derepressed mRNAs in Poly LvM, GO terms enriched in biological processes for repressed mRNAs in Poly LvM, Comparison of GO terms enriched in biological processes for derepressed early-to-mid mRNAs and repressed mid-to-late mRNAs in polysomes, Comparison of GO terms enriched in biological processes for repressed early-to-mid mRNAs and derepressed mid-to-late mRNAs in polysomes.

File Name: Supplementary Data 5
Description: Intersection tables snRNAseq (Early, Mid, Late) and human polysome profiling (MvE, LvE, LvM)
-Tabs: EARLY clusters vs. Monosomes, MID clusters vs. Monosomes, LATE clusters vs. Monosomes, EARLY clusters vs. Polysomes, MID clusters vs. Polysomes, LATE clusters vs. Polysomes.

File Name: Supplementary Data 6
Description: SynGO EARLY and LATE SP & ExN L56 genes
-Tabs: EARLY SP&ExNL56_genelist, EARLY SP&ExNL56_syngo_ontolog., EARLY SP&ExNL56_annotations, EARLY ExN L56_genelist, EARLY ExN L56_syngo_ontologies, EARLY ExN L56_syngo_annotations, LATE genelist, LATE syngo_ontologies, LATE annotations, LATE ExN L56_genelist, LATE ExN L56_syngo_ontologies, LATE ExN L56_syngo_annotations.

File Name: Supplementary Data 7
Description: DEG SP vs Neuro by stage reduced LFC with GO
-Tabs: List of 6100 human RBP candidates from RBP2GO, Sorted differential gene expression analysis between early "SP&ExNL5/6" versus other early neuronal clusters, Sorted differential gene expression analysis between mid "SP" clusters versus other mid neuronal clusters, Sorted differential gene expression analysis between late "SP" clusters versus other late neuronal clusters.

File Name: Supplementary Data 8
Description: hCELF4 RIP-RNAseq analysis + SynGO CELF4-RIP Early, mid and late stages
-Tabs: CELF4 RIP-RNAseq data per each devo-stage, EARLY CELF4-RIP_genelist, EARLY CELF4-RIP_syngo_ontologies, EARLY CELF4-RIP_syngo_annotat., MID CELF4-RIP_genelist, MID CELF4-RIP_syngo_ontologies, MID CELF4-RIP_syngo_annotat., LATE CELF4-RIP_genelist, LATE CELF4-RIP_syngo_ontologies, LATE CELF4-RIP_syngo_annotat., Shared CELF4-RIP_genelist, Shared CELF4-RIP_syngo_ontologies, Shared CELF4-RIP_syngo_annotat.

File Name: Supplementary Data 9
Description: mCelf4 RIP-RNAseq
-Tabs: mCelf4/IgG RIP-RNAseq analysis, mCelf4 vs hCELF4 targets from RIP-RNAseq analyses.

File Name: Supplementary Data 10
Description: Polysome isoform-level DEG analysis and gene-level analysis (1,069 Poly vs Mono(cKOvsWT))
-Tabs: Levels of mRNA isoforms in the input in Emx1-Cre Celf4 cKO compared to WTs, Levels of mRNA isoforms in the monosomes in Emx1-Cre Celf4 cKO compared to WTs, Levels of mRNA isoforms in the polysomes in Emx1-Cre Celf4 cKO compared to WTs, List of transcriptionally-stable isoform mRNAs associated with monosomes in Emx1-Cre Celf4 cKO, List of transcriptionally-stable isoform mRNAs associated with polysomes in Emx1-Cre Celf4 cKO, Gene-level analysis_1,069 Poly/Mono (cKO/WT), p<0.05.

File Name: Supplementary Data 11
Description: Primers for RT-PCR and probes for FISH
-Tabs: List of RT-PCR primers and FISH probes used in mouse experiments, List of RT-PCR primers and FISH probes used in human experiments.

File Name: Supplementary Data 12
Description: CellInsight CX7 protocol parameters
