# Supplementary material for: Celf4 controls mRNA translation underlying synaptic development in the prenatal mammalian neocortex
Source: Nat Commun. 2023 Sep 27;14:6025. doi: 10.1038/s41467-023-41730-8 (PMC10533865; doi:10.1038/s41467-023-41730-8)
Supplement: Supplementary file 4 — Supplementary data 1-12 [file 41467_2023_41730_MOESM4_ESM.zip › 5. Supplementary data_Celf4 manuscript/Supplementary data 12_CellInsight CX7 protocol parameters.htm]

iva synNP\_SYNAP\_RHN-Spots-PDLam\_20x\_3Ch\_X1\_2x2\_1x


## iva synNP\_SYNAP\_RHN-Spots-PDLam\_20x\_3Ch\_X1\_2x2\_1x [Version 1]

Last Modified on 7/9/2020 3:49:21 PM

---


NOTE: The information here represents data that is saved in the
database with an Assay Protocol. This data does not include scan
settings like Field Offset, Form Factor, Scan Area, Store Images or Show
Composite Image.

|  |  |  |  |
| --- | --- | --- | --- |
| | Comments | | --- | | Test images run with Rat Hippocampal Neurons | | |
| | Image Acquisition | | | --- | --- | | Objective | 20x | | Camera Name | X1;1.00 | | Acquisition Camera Mode | 1104x1104 (2x2 binning) (2208x2208;2x2) | | AutoFocus Camera Mode | 1104x1104 (2x2 binning) (2208x2208;2x2) | | AutoFocus Field Interval | 0 |      | AutoFocus Parameters | | | --- | --- | | Fine Focus Step Size | 9.9 | | Fine Focus Plane Count | 9 | | Coarse Focus Step Size | 39.6 | | Coarse Focus Plane Count | 16 | | Smart Focus Plane Count | 21 | | Use Extended Range Focusing | False | | Apply Backlash Correction | False | | AutoFocus Method | STANDARD | | Laser AutoFocus Method | LASER | | How To Focus During Scan | 2 | | Use Relaxed Pass/Fail Criteria | False | | Focus Edge Threshhold | 0 | | Focus Adjustment | 0 | | Focus Score Min Ratio | 0.25 | | Focus Score Mid Ratio | 0.4 | | Focus Score Max Ratio | 0.5 | | Focus Exposure Time for AutoExpose (seconds) | 6.51393456288515E-02 |      | Scan Limits | | | --- | --- | | Max Fields for Well | 9 | | Min Objects for Well | No Limit | | Max Sparse Fields for Well | No Limit | | Min Objects for Field | N/A | | Max Sparse Wells for Plate | N/A | | | Channel 1: Nucleus | | | | --- | --- | --- | | Dye | 386-23\_BGRFRN\_BGRFRN | | | Apply Illumination Correction | False | | | Apply Background Correction | True | | | Gain | 2 | | | Z Offset | 0.00 | | | Step Size | 0.00 | | | Number of Steps | 0 | | | Projection Method |  | | | Projection Direction | None | | | Detection Mode | Widefield | | | Grid Type |  | | | Pin Hole Size |  | | | Intensity Percent | 100 | | | Exposure Parameters | | | | --- | --- | --- | | Method | AutoExpose | | | AutoExpose Method | Peak Target(Pixel Correction) | | | Initial Exposure Time (seconds) | 0.097709 | | | Wells to AutoExpose | B2 F11 | | | Fields to AutoExpose | 5 6 | | | Target | 25 | | | Tolerance | 2 | | | Pixels To Skip | 25 | | | Max Exposure Time | 16 | | | Max Iterations | 8 | | | Object Identification | | | | --- | --- | --- | | Method | FixedThreshold | | | Value | 1 | | | Object Selection Parameter | Min | Max | | --- | --- | --- | | NucAreaCh1 | 0 | 1000000000000 | | NucShapeP2ACh1 | 0 | 1000 | | NucShapeLWRCh1 | 0 | 1000 | | NucAvgIntenCh1 | 0 | 65535 | | NucVarIntenCh1 | 0 | 1000000 | | NucTotalIntenCh1 | 0 | 1000000000000 | | Display Options | | | | --- | --- | --- | | Composite Color (Hex) | #0000FF |  | | ValidNucleus | #0000FF |  | | RejectedNucleus | #FF7F00 |  |     | Channel 2: Neuron | | | | --- | --- | --- | | Dye | 549-15\_BGRFRN\_BGRFRN | | | Apply Illumination Correction | False | | | Apply Background Correction | True | | | Gain | 2 | | | Z Offset | 0.00 | | | Step Size | 0.00 | | | Number of Steps | 0 | | | Projection Method |  | | | Projection Direction | None | | | Detection Mode | Widefield | | | Grid Type |  | | | Pin Hole Size |  | | | Intensity Percent | 100 | | | Exposure Parameters | | | | --- | --- | --- | | Method | AutoExpose | | | AutoExpose Method | Peak Target(Pixel Correction) | | | Initial Exposure Time (seconds) | 0.260557 | | | Wells to AutoExpose | B2 F11 | | | Fields to AutoExpose | 5 6 | | | Target | 25 | | | Tolerance | 2 | | | Pixels To Skip | 25 | | | Max Exposure Time | 16 | | | Max Iterations | 8 | | | Object Identification | | | | --- | --- | --- | | Method | FixedThreshold | | | Value | 1 | | | Object Selection Parameter | Min | Max | | --- | --- | --- | | NeuriteLengthCh2 | 0 | 10000000000 | | NeuriteWidthCh2 | 0 | 10000000000 | | NeuriteAvgIntenCh2 | 0 | 65535 | | NeuriteTotalIntenCh2 | 0 | 1000000000000 | | NeuriteBranchPointCountCh2 | 0 | 10000000000 | | CellBodyNucCountCh2 | 0 | 65535 | | CellBodyAreaCh2 | 0 | 1000000000000 | | CellBodyShapeP2ACh2 | 0 | 1000 | | CellBodyShapeLWRCh2 | 0 | 1000 | | CellBodyAvgIntenCh2 | 0 | 65535 | | CellBodyVarIntenCh2 | 0 | 1000000 | | CellBodyTotalIntenCh2 | 0 | 1000000000000 | | Display Options | | | | --- | --- | --- | | Composite Color (Hex) | #FFFFFF |  | | Neurite/SpotOverlap | #00FF00 |  | | Neurite\_\_/SpotCh4 | #00FFFF |  | | SelectedCellBody/Neurite | #0080FF |  | | RejectedCellBody | #FF0000 |  | | NeuritePoint | #FFFF00 |  |     | Channel 3: vgat | | | | --- | --- | --- | | Dye | 485-20\_BGRFRN\_BGRFRN | | | Apply Illumination Correction | False | | | Apply Background Correction | True | | | Gain | 2 | | | Z Offset | 2.00 | | | Step Size | 0.00 | | | Number of Steps | 0 | | | Projection Method |  | | | Projection Direction | None | | | Detection Mode | Widefield | | | Grid Type |  | | | Pin Hole Size |  | | | Intensity Percent | 100 | | | Exposure Parameters | | | | --- | --- | --- | | Method | AutoExpose | | | AutoExpose Method | Peak Target(Pixel Correction) | | | Initial Exposure Time (seconds) | 0.162848 | | | Wells to AutoExpose | B2 F11 | | | Fields to AutoExpose | 5 6 | | | Target | 25 | | | Tolerance | 2 | | | Pixels To Skip | 25 | | | Max Exposure Time | 16 | | | Max Iterations | 8 | | | Object Identification | | | | --- | --- | --- | | Method | FixedThreshold | | | Value | 5 | | | Object Selection Parameter | Min | Max | | --- | --- | --- | | SpotAreaCh3 | 0 | 1000000000000 | | SpotShapeP2ACh3 | 0 | 1000 | | SpotShapeLWRCh3 | 0 | 1000 | | SpotAvgIntenCh3 | 0 | 65535 | | SpotTotalIntenCh3 | 0 | 1000000000000 | | AvgIntenCh3 | 0 | 65535 | | TotalIntenCh3 | 0 | 1000000000000 | | Display Options | | | | --- | --- | --- | | Composite Color (Hex) | #FF0000 |  | | Neurite\_/SpotCh3 | #FF00FF |  |     | Channel 4: gephy | | | | --- | --- | --- | | Dye | 386-23\_BGRFRN\_BGRFRN | | | Apply Illumination Correction | False | | | Apply Background Correction | True | | | Gain | 2 | | | Z Offset | 0.00 | | | Step Size | 0.00 | | | Number of Steps | 0 | | | Projection Method |  | | | Projection Direction | none | | | Detection Mode | widefield | | | Grid Type |  | | | Pin Hole Size |  | | | Intensity Percent | 100 | | | Exposure Parameters | | | | --- | --- | --- | | Method | Fixed | | | Exposure Time (seconds) | 0.1 | | | Object Identification | | | | --- | --- | --- | | Method | FixedThreshold | | | Value | 5 | | | Object Selection Parameter | Min | Max | | --- | --- | --- | | SpotAreaCh4 | 0 | 1000000000000 | | SpotShapeP2ACh4 | 0 | 1000 | | SpotShapeLWRCh4 | 0 | 1000 | | SpotAvgIntenCh4 | 0 | 65535 | | SpotVarIntenCh4 | 0 | 65535 | | SpotTotalIntenCh4 | 0 | 1000000000000 | | AvgIntenCh4 | 0 | 65535 | | TotalIntenCh4 | 0 | 1000000000000 | | Display Options | | | | --- | --- | --- | | Composite Color (Hex) | #FFFF00 |  | |
| ---  | Assay | | | --- | --- | | Assay Algorithm | NeuronalProfiling.V4.2 | | Assay Version | 6.1 (Locally Installed Version: 6.1.0.4201) | | Focus Channel | 1 | | #Channels | 4 | | Assay Parameters | | | --- | --- | | PixelSize | 0.454 | | Type\_1\_EventDefinition | 90304.032 | | Type\_2\_EventDefinition | 0 | | Type\_3\_EventDefinition | 0 | | AvgIntenCh3LevelHigh | 32767 | | AvgIntenCh3LevelHigh\_CC | 1 | | AvgIntenCh4LevelHigh | 65535 | | AvgIntenCh4LevelHigh\_CC | 1 | | BranchPointAvgCountCh2LevelHigh | 32767 | | BranchPointAvgCountCh2LevelHigh\_CC | 1 | | BranchPointAvgDistFromCellBodyCh2LevelHigh | 10000000000 | | BranchPointAvgDistFromCellBodyCh2LevelHigh\_CC | 1 | | BranchPointCountPerNeuriteLengthCh2LevelHigh | 23064 | | BranchPointCountPerNeuriteLengthCh2LevelHigh\_CC | 1 | | BranchPointTotalCountCh2LevelHigh | 32767 | | BranchPointTotalCountCh2LevelHigh\_CC | 1 | | CellBodyAreaCh2LevelHigh | 1000000000000 | | CellBodyAreaCh2LevelHigh\_CC | 1 | | CellBodyAvgIntenCh2LevelHigh | 32767 | | CellBodyAvgIntenCh2LevelHigh\_CC | 1 | | CellBodyNucAvgIntenCh1LevelHigh | 32767 | | CellBodyNucAvgIntenCh1LevelHigh\_CC | 1 | | CellBodyNucCountCh2LevelHigh | 32767 | | CellBodyNucCountCh2LevelHigh\_CC | 1 | | CellBodyNucTotalAreaCh1LevelHigh | 1000000000000 | | CellBodyNucTotalAreaCh1LevelHigh\_CC | 1 | | CellBodyNucTotalIntenCh1LevelHigh | 1000000000000 | | CellBodyNucTotalIntenCh1LevelHigh\_CC | 1 | | CellBodyShapeLWRCh2LevelHigh | 1000 | | CellBodyShapeLWRCh2LevelHigh\_CC | 1 | | CellBodyShapeP2ACh2LevelHigh | 1000 | | CellBodyShapeP2ACh2LevelHigh\_CC | 1 | | CellBodySpot%OverlapAreaCh3LevelHigh | 100 | | CellBodySpot%OverlapAreaCh3LevelHigh\_CC | 1 | | CellBodySpot%OverlapAreaCh4LevelHigh | 100 | | CellBodySpot%OverlapAreaCh4LevelHigh\_CC | 1 | | CellBodySpot%OverlapCountCh3LevelHigh | 100 | | CellBodySpot%OverlapCountCh3LevelHigh\_CC | 1 | | CellBodySpot%OverlapCountCh4LevelHigh | 100 | | CellBodySpot%OverlapCountCh4LevelHigh\_CC | 1 | | CellBodySpotAvgIntenCh3LevelHigh | 32767 | | CellBodySpotAvgIntenCh3LevelHigh\_CC | 1 | | CellBodySpotAvgIntenCh4LevelHigh | 65535 | | CellBodySpotAvgIntenCh4LevelHigh\_CC | 1 | | CellBodySpotOverlapAreaCh3LevelHigh | 1000000000000 | | CellBodySpotOverlapAreaCh3LevelHigh\_CC | 1 | | CellBodySpotOverlapAreaCh4LevelHigh | 1000000000000 | | CellBodySpotOverlapAreaCh4LevelHigh\_CC | 1 | | CellBodySpotOverlapCountCh3LevelHigh | 65535 | | CellBodySpotOverlapCountCh3LevelHigh\_CC | 1 | | CellBodySpotOverlapCountCh4LevelHigh | 65535 | | CellBodySpotOverlapCountCh4LevelHigh\_CC | 1 | | CellBodySpotTotalAreaCh3LevelHigh | 1000000000000 | | CellBodySpotTotalAreaCh3LevelHigh\_CC | 1 | | CellBodySpotTotalAreaCh4LevelHigh | 1000000000000 | | CellBodySpotTotalAreaCh4LevelHigh\_CC | 1 | | CellBodySpotTotalAreaRatioCh4Ch3LevelHigh | 1000000000000 | | CellBodySpotTotalAreaRatioCh4Ch3LevelHigh\_CC | 1 | | CellBodySpotTotalCountCh3LevelHigh | 32767 | | CellBodySpotTotalCountCh3LevelHigh\_CC | 1 | | CellBodySpotTotalCountCh4LevelHigh | 65535 | | CellBodySpotTotalCountCh4LevelHigh\_CC | 1 | | CellBodySpotTotalIntenCh3LevelHigh | 1000000000000 | | CellBodySpotTotalIntenCh3LevelHigh\_CC | 1 | | CellBodySpotTotalIntenCh4LevelHigh | 1000000000000 | | CellBodySpotTotalIntenCh4LevelHigh\_CC | 1 | | CellBodySpotTotalIntenRatioCh3Ch2LevelHigh | 1000000000000 | | CellBodySpotTotalIntenRatioCh3Ch2LevelHigh\_CC | 1 | | CellBodySpotTotalIntenRatioCh4Ch2LevelHigh | 1000000000000 | | CellBodySpotTotalIntenRatioCh4Ch2LevelHigh\_CC | 1 | | CellBodySpotTotalIntenRatioCh4Ch3LevelHigh | 1000000000000 | | CellBodySpotTotalIntenRatioCh4Ch3LevelHigh\_CC | 1 | | CellBodyTotalIntenCh2LevelHigh | 1000000000000 | | CellBodyTotalIntenCh2LevelHigh\_CC | 1 | | CellBodyVarIntenCh2LevelHigh | 65535 | | CellBodyVarIntenCh2LevelHigh\_CC | 1 | | CrossPointAvgCountCh2LevelHigh | 32767 | | CrossPointAvgCountCh2LevelHigh\_CC | 1 | | CrossPointTotalCountCh2LevelHigh | 32767 | | CrossPointTotalCountCh2LevelHigh\_CC | 1 | | MinRefAvgNeuronCountPerField | 2 | | NeuriteAvgIntenCh2LevelHigh | 32767 | | NeuriteAvgIntenCh2LevelHigh\_CC | 1 | | NeuriteAvgLengthCh2LevelHigh | 10000000000 | | NeuriteAvgLengthCh2LevelHigh\_CC | 1 | | NeuriteCriticalValueCh2LevelHigh | 10000000000 | | NeuriteCriticalValueCh2LevelHigh\_CC | 1 | | NeuriteDendriteMaxCh2LevelHigh | 32767 | | NeuriteDendriteMaxCh2LevelHigh\_CC | 1 | | NeuriteMaxLengthWithBranchesCh2LevelHigh | 10000000000 | | NeuriteMaxLengthWithBranchesCh2LevelHigh\_CC | 1 | | NeuriteMaxLengthWithoutBranchesCh2LevelHigh | 10000000000 | | NeuriteMaxLengthWithoutBranchesCh2LevelHigh\_CC | 1 | | NeuriteRamificationIndexCh2LevelHigh | 10000000000 | | NeuriteRamificationIndexCh2LevelHigh\_CC | 1 | | NeuriteSpot%OverlapAreaCh3LevelHigh | 100 | | NeuriteSpot%OverlapAreaCh3LevelHigh\_CC | 1 | | NeuriteSpot%OverlapAreaCh4LevelHigh | 100 | | NeuriteSpot%OverlapAreaCh4LevelHigh\_CC | 1 | | NeuriteSpot%OverlapCountCh3LevelHigh | 100 | | NeuriteSpot%OverlapCountCh3LevelHigh\_CC | 1 | | NeuriteSpot%OverlapCountCh4LevelHigh | 100 | | NeuriteSpot%OverlapCountCh4LevelHigh\_CC | 1 | | NeuriteSpotAvgAreaCh3LevelHigh | 1000000000000 | | NeuriteSpotAvgAreaCh3LevelHigh\_CC | 1 | | NeuriteSpotAvgAreaCh4LevelHigh | 1000000000000 | | NeuriteSpotAvgAreaCh4LevelHigh\_CC | 1 | | NeuriteSpotAvgCountCh3LevelHigh | 32767 | | NeuriteSpotAvgCountCh3LevelHigh\_CC | 1 | | NeuriteSpotAvgCountCh4LevelHigh | 65535 | | NeuriteSpotAvgCountCh4LevelHigh\_CC | 1 | | NeuriteSpotAvgIntenCh3LevelHigh | 32767 | | NeuriteSpotAvgIntenCh3LevelHigh\_CC | 1 | | NeuriteSpotAvgIntenCh4LevelHigh | 65535 | | NeuriteSpotAvgIntenCh4LevelHigh\_CC | 1 | | NeuriteSpotOverlapAreaCh3LevelHigh | 1000000000000 | | NeuriteSpotOverlapAreaCh3LevelHigh\_CC | 1 | | NeuriteSpotOverlapAreaCh4LevelHigh | 1000000000000 | | NeuriteSpotOverlapAreaCh4LevelHigh\_CC | 1 | | NeuriteSpotOverlapCountCh3LevelHigh | 65535 | | NeuriteSpotOverlapCountCh3LevelHigh\_CC | 1 | | NeuriteSpotOverlapCountCh4LevelHigh | 65535 | | NeuriteSpotOverlapCountCh4LevelHigh\_CC | 1 | | NeuriteSpotTotalAreaCh3LevelHigh | 1000000000000 | | NeuriteSpotTotalAreaCh3LevelHigh\_CC | 1 | | NeuriteSpotTotalAreaCh4LevelHigh | 1000000000000 | | NeuriteSpotTotalAreaCh4LevelHigh\_CC | 1 | | NeuriteSpotTotalAreaRatioCh4Ch3LevelHigh | 1000000000000 | | NeuriteSpotTotalAreaRatioCh4Ch3LevelHigh\_CC | 1 | | NeuriteSpotTotalCountCh3LevelHigh | 32767 | | NeuriteSpotTotalCountCh3LevelHigh\_CC | 1 | | NeuriteSpotTotalCountCh4LevelHigh | 65535 | | NeuriteSpotTotalCountCh4LevelHigh\_CC | 1 | | NeuriteSpotTotalIntenCh3LevelHigh | 1000000000000 | | NeuriteSpotTotalIntenCh3LevelHigh\_CC | 1 | | NeuriteSpotTotalIntenCh4LevelHigh | 1000000000000 | | NeuriteSpotTotalIntenCh4LevelHigh\_CC | 1 | | NeuriteSpotTotalIntenRatioCh3Ch2LevelHigh | 1000000000000 | | NeuriteSpotTotalIntenRatioCh3Ch2LevelHigh\_CC | 1 | | NeuriteSpotTotalIntenRatioCh4Ch2LevelHigh | 1000000000000 | | NeuriteSpotTotalIntenRatioCh4Ch2LevelHigh\_CC | 1 | | NeuriteSpotTotalIntenRatioCh4Ch3LevelHigh | 1000000000000 | | NeuriteSpotTotalIntenRatioCh4Ch3LevelHigh\_CC | 1 | | NeuriteTotalAreaCh2LevelHigh | 1000000000000 | | NeuriteTotalAreaCh2LevelHigh\_CC | 1 | | NeuriteTotalCountCh2LevelHigh | 32767 | | NeuriteTotalCountCh2LevelHigh\_CC | 1 | | NeuriteTotalIntenCh2LevelHigh | 1000000000000 | | NeuriteTotalIntenCh2LevelHigh\_CC | 1 | | NeuriteTotalLengthCh2LevelHigh | 10000000000 | | NeuriteTotalLengthCh2LevelHigh\_CC | 1 | | NeuriteVarIntenCh2LevelHigh | 32767 | | NeuriteVarIntenCh2LevelHigh\_CC | 1 | | NeuriteWidthCh2LevelHigh | 10000000000 | | NeuriteWidthCh2LevelHigh\_CC | 1 | | TotalIntenCh3LevelHigh | 1000000000000 | | TotalIntenCh3LevelHigh\_CC | 1 | | TotalIntenCh4LevelHigh | 1000000000000 | | TotalIntenCh4LevelHigh\_CC | 1 | | UseMicrometers | 0 | | BackgroundCorrectionCh1 | 107 | | BackgroundCorrectionCh2 | 107 | | BackgroundCorrectionCh3 | 225 | | BackgroundCorrectionCh4 | 225 | | CellBodyAndNeuriteTypeCh2 | 0 | | CellBodyDemarcationCh2 | 7 | | CellBodyMaskModifierCh2 | 55 | | CellBodyMaskModifierCh3 | 0 | | CellBodyMaskModifierCh4 | 0 | | CellBodySegmentationCh2 | 0 | | CellBodySmoothFactorCh2 | 1 | | DisplaySpotOverlapCh3Ch4 | 4 | | MinAreaSpotOverlapCh3Ch4 | -1 | | MinCellBodyNucOverlapCh2 | 40 | | NA\_StrayNeuriteAndSpotDisplayCh2 | 0 | | NeuriteAggressiveTracingCh2 | 0 | | NeuriteDetectMethodCh2 | 2 | | NeuriteDetectRadiusCh2 | 4 | | NeuriteDirectionCh2 | 3 | | NeuriteGapToleranceCh2 | 1 | | NeuriteIdentificationModifierCh2 | -0.98 | | NeuritePointDisplayModeCh2 | 0 | | NeuritePointResolutionCh2 | 1 | | NeuriteSmoothFactorCh2 | 1 | | NeuriteSpotDomainModifierCh3 | 0 | | NeuriteSpotDomainModifierCh4 | 0 | | NeuriteTracingWithoutSeedsCh2 | 0 | | NucCleanupCh1 | 1 | | NucSegmentationCh1 | 0 | | NucSmoothFactorCh1 | 1 | | NucTypeCh1 | 0 | | RejectBorderCellBodiesCh2 | 0 | | RejectBorderNucsCh1 | 0 | | RejectMultiplyTracedNeuritesCh2 | 0 | | SpotDetectMethodCh3 | 1 | | SpotDetectMethodCh4 | 1 | | SpotDetectRadiusCh3 | 3 | | SpotDetectRadiusCh4 | 3 | | SpotSegmentationCh3 | 0 | | SpotSegmentationCh4 | 0 | | SpotSmoothFactorCh3 | 1 | | SpotSmoothFactorCh4 | 1 | | SpotTypeCh3 | 0 | | SpotTypeCh4 | 0 | | UseCellBodyZOIForNeuriteTracingCh2 | 0 | | UseNucForCellBodySegmentationCh2 | 0 | | UseReferenceWells | 0 |      | Well Feature Extents | | | | --- | --- | --- | | Feature Name | Lower Extent | Upper Extent | | --- | --- | --- | | %HIGH\_CellBodyNucTotalAreaCh1 | 1 | 100 | | %HIGH\_CellBodyNucTotalIntenCh1 | 1 | 100 | | %HIGH\_CellBodySpot%OverlapCountCh4 | 1 | 100 | | %HIGH\_CellBodySpotAvgIntenCh3 | 1 | 100 | | %HIGH\_CellBodySpotOverlapAreaCh4 | 1 | 100 | | %HIGH\_CellBodySpotOverlapCountCh4 | 1 | 100 | | %HIGH\_CellBodySpotTotalIntenCh3 | 1 | 100 | | %HIGH\_CellBodySpotTotalIntenRatioCh3Ch2 | 1 | 100 | | %HIGH\_E1BranchPointTotalCountCh2 | 1 | 100 | | %HIGH\_E1CellBodyAvgIntenCh2 | 1 | 100 | | %HIGH\_E1CellBodySpot%OverlapCountCh3 | 1 | 100 | | %HIGH\_E1CellBodySpot%OverlapCountCh4 | 1 | 100 | | %HIGH\_E1CellBodySpotOverlapCountCh3 | 1 | 100 | | %HIGH\_E1CellBodySpotOverlapCountCh4 | 1 | 100 | | %HIGH\_E1CellBodySpotTotalAreaRatioCh4Ch3 | 1 | 100 | | %HIGH\_E1CellBodySpotTotalIntenRatioCh3Ch2 | 1 | 100 | | %HIGH\_E1NeuriteDendriteMaxCh2 | 1 | 100 | | %HIGH\_E1NeuriteRamificationIndexCh2 | 1 | 100 | | %HIGH\_E1NeuriteSpot%OverlapAreaCh3 | 1 | 100 | | %HIGH\_E1NeuriteSpot%OverlapCountCh3 | 1 | 100 | | %HIGH\_E1NeuriteSpotOverlapAreaCh3 | 1 | 100 | | %HIGH\_E1NeuriteSpotOverlapCountCh4 | 1 | 100 | | %HIGH\_E1NeuriteSpotTotalAreaRatioCh4Ch3 | 1 | 100 | | %HIGH\_E1NeuriteSpotTotalIntenRatioCh4Ch3 | 1 | 100 | | %HIGH\_E1NeuriteTotalCountCh2 | 1 | 100 | | %HIGH\_E1NeuriteTotalLengthCh2 | 1 | 100 | | CV\_E1CrossPointAvgCountCh2 | 1 | 100 | | CV\_E1CrossPointTotalCountCh2 | 1 | 100 | | CV\_E1NeuriteMaxLengthWithoutBranchesCh2 | 1 | 100 | | CV\_E1NeuriteSpotAvgAreaCh3 | 1 | 100 | | CV\_E1NeuriteSpotAvgCountCh3 | 1 | 100 | | CV\_E1NeuriteSpotAvgCountCh4 | 1 | 100 | | CV\_E1NeuriteSpotTotalAreaCh3 | 1 | 100 | | CV\_E1NeuriteSpotTotalAreaCh4 | 1 | 100 | | CV\_E1NeuriteSpotTotalCountCh4 | 1 | 100 | | CV\_E1NeuriteTotalAreaCh2 | 1 | 100 | | CV\_E1NeuriteWidthCh2 | 1 | 100 | | CV\_E1TotalIntenCh4 | 1 | 100 | | CV\_E2CellBodyAreaCh2 | 1 | 100 | | CV\_E2CellBodyNucAvgIntenCh1 | 1 | 100 | | CV\_E2CellBodyNucCountCh2 | 1 | 100 | | CV\_E2CellBodySpot%OverlapAreaCh4 | 1 | 100 | | CV\_E2CellBodySpotTotalAreaCh3 | 1 | 100 | | CV\_E2CellBodySpotTotalCountCh3 | 1 | 100 | | CV\_E2CellBodySpotTotalCountCh4 | 1 | 100 | | CV\_E2CellBodySpotTotalIntenCh3 | 1 | 100 | | CV\_E2CrossPointAvgCountCh2 | 1 | 100 | | CV\_E2NeuriteSpot%OverlapAreaCh4 | 1 | 100 | | CV\_E2NeuriteSpotAvgAreaCh3 | 1 | 100 | | CV\_E2NeuriteSpotAvgCountCh4 | 1 | 100 | | CV\_E2NeuriteSpotAvgIntenCh3 | 1 | 100 | | %HIGH\_CellBodyNucCountCh2 | 1 | 100 | | %HIGH\_CellBodySpot%OverlapAreaCh4 | 1 | 100 | | %HIGH\_CellBodySpot%OverlapCountCh3 | 1 | 100 | | %HIGH\_CellBodySpotTotalAreaCh3 | 1 | 100 | | %HIGH\_CellBodySpotTotalCountCh4 | 1 | 100 | | %HIGH\_CellBodySpotTotalIntenRatioCh4Ch3 | 1 | 100 | | %HIGH\_E1BranchPointCountPerNeuriteLengthCh2 | 1 | 100 | | %HIGH\_E1CellBodyNucTotalAreaCh1 | 1 | 100 | | %HIGH\_E1CellBodyShapeP2ACh2 | 1 | 100 | | %HIGH\_E1CellBodySpot%OverlapAreaCh3 | 1 | 100 | | %HIGH\_E1CellBodySpot%OverlapAreaCh4 | 1 | 100 | | %HIGH\_E1CellBodySpotAvgIntenCh3 | 1 | 100 | | %HIGH\_E1CellBodySpotAvgIntenCh4 | 1 | 100 | | %HIGH\_E1CellBodySpotTotalAreaCh3 | 1 | 100 | | %HIGH\_E1CellBodySpotTotalIntenRatioCh4Ch3 | 1 | 100 | | %HIGH\_E1CellBodyTotalIntenCh2 | 1 | 100 | | %HIGH\_E1NeuriteAvgIntenCh2 | 1 | 100 | | %HIGH\_E1NeuriteCriticalValueCh2 | 1 | 100 | | %HIGH\_E1NeuriteMaxLengthWithBranchesCh2 | 1 | 100 | | %HIGH\_E1NeuriteSpotOverlapAreaCh4 | 1 | 100 | | %HIGH\_E1NeuriteSpotTotalIntenCh4 | 1 | 100 | | %HIGH\_E1NeuriteSpotTotalIntenRatioCh3Ch2 | 1 | 100 | | %HIGH\_E1NeuriteSpotTotalIntenRatioCh4Ch2 | 1 | 100 | | CV\_E1NeuriteAvgIntenCh2 | 1 | 100 | | CV\_E1NeuriteMaxLengthWithBranchesCh2 | 1 | 100 | | CV\_E1NeuriteSpotOverlapAreaCh4 | 1 | 100 | | CV\_E1NeuriteSpotOverlapCountCh4 | 1 | 100 | | CV\_E1NeuriteSpotTotalIntenCh3 | 1 | 100 | | CV\_E1NeuriteSpotTotalIntenCh4 | 1 | 100 | | CV\_E1NeuriteSpotTotalIntenRatioCh3Ch2 | 1 | 100 | | CV\_E1NeuriteTotalLengthCh2 | 1 | 100 | | CV\_E1TotalIntenCh3 | 1 | 100 | | CV\_E2AvgIntenCh4 | 1 | 100 | | CV\_E2BranchPointCountPerNeuriteLengthCh2 | 1 | 100 | | CV\_E2CellBodyNucTotalAreaCh1 | 1 | 100 | | CV\_E2CellBodyShapeP2ACh2 | 1 | 100 | | CV\_E2CellBodySpot%OverlapCountCh4 | 1 | 100 | | CV\_E2CellBodySpotAvgIntenCh3 | 1 | 100 | | CV\_E2CellBodySpotAvgIntenCh4 | 1 | 100 | | CV\_E2CellBodySpotOverlapCountCh3 | 1 | 100 | | CV\_E2CellBodySpotTotalAreaCh4 | 1 | 100 | | CV\_E2CellBodyTotalIntenCh2 | 1 | 100 | | CV\_E2CellBodyVarIntenCh2 | 1 | 100 | | CV\_E2CrossPointTotalCountCh2 | 1 | 100 | | CV\_E2NeuriteAvgIntenCh2 | 1 | 100 | | CV\_E2NeuriteCriticalValueCh2 | 1 | 100 | | CV\_E2NeuriteMaxLengthWithBranchesCh2 | 1 | 100 | | CV\_E2NeuriteSpot%OverlapCountCh3 | 1 | 100 | | CV\_E2NeuriteSpotAvgCountCh3 | 1 | 100 | | CV\_E2NeuriteSpotOverlapCountCh4 | 1 | 100 | | CV\_E2NeuriteSpotTotalAreaCh4 | 1 | 100 | | %HIGH\_CellBodySpotOverlapAreaCh3 | 1 | 100 | | %HIGH\_CellBodySpotOverlapCountCh3 | 1 | 100 | | %HIGH\_CellBodySpotTotalAreaRatioCh4Ch3 | 1 | 100 | | %HIGH\_CellBodySpotTotalCountCh3 | 1 | 100 | | %HIGH\_CellBodySpotTotalIntenRatioCh4Ch2 | 1 | 100 | | %HIGH\_E1AvgIntenCh3 | 1 | 100 | | %HIGH\_E1BranchPointAvgCountCh2 | 1 | 100 | | %HIGH\_E1BranchPointAvgDistFromCellBodyCh2 | 1 | 100 | | %HIGH\_E1CellBodyNucTotalIntenCh1 | 1 | 100 | | %HIGH\_E1CellBodyShapeLWRCh2 | 1 | 100 | | %HIGH\_E1CellBodySpotOverlapAreaCh3 | 1 | 100 | | %HIGH\_E1CellBodySpotOverlapAreaCh4 | 1 | 100 | | %HIGH\_E1CellBodySpotTotalIntenCh3 | 1 | 100 | | %HIGH\_E1CellBodySpotTotalIntenRatioCh4Ch2 | 1 | 100 | | %HIGH\_E1CellBodyVarIntenCh2 | 1 | 100 | | %HIGH\_E1NeuriteAvgLengthCh2 | 1 | 100 | | %HIGH\_E1NeuriteMaxLengthWithoutBranchesCh2 | 1 | 100 | | %HIGH\_E1NeuriteSpot%OverlapAreaCh4 | 1 | 100 | | %HIGH\_E1NeuriteSpot%OverlapCountCh4 | 1 | 100 | | %HIGH\_E1NeuriteSpotAvgIntenCh3 | 1 | 100 | | %HIGH\_E1NeuriteSpotAvgIntenCh4 | 1 | 100 | | %HIGH\_E1NeuriteSpotOverlapCountCh3 | 1 | 100 | | %HIGH\_E1NeuriteSpotTotalCountCh3 | 1 | 100 | | %HIGH\_E1NeuriteSpotTotalCountCh4 | 1 | 100 | | %HIGH\_E1NeuriteVarIntenCh2 | 1 | 100 | | CV\_E1NeuriteAvgLengthCh2 | 1 | 100 | | CV\_E1NeuriteSpot%OverlapAreaCh3 | 1 | 100 | | CV\_E1NeuriteSpot%OverlapAreaCh4 | 1 | 100 | | CV\_E1NeuriteSpot%OverlapCountCh4 | 1 | 100 | | CV\_E1NeuriteSpotAvgAreaCh4 | 1 | 100 | | CV\_E1NeuriteSpotAvgIntenCh3 | 1 | 100 | | CV\_E1NeuriteSpotAvgIntenCh4 | 1 | 100 | | CV\_E1NeuriteSpotTotalCountCh3 | 1 | 100 | | CV\_E1NeuriteTotalIntenCh2 | 1 | 100 | | CV\_E1NeuriteVarIntenCh2 | 1 | 100 | | CV\_E2BranchPointAvgDistFromCellBodyCh2 | 1 | 100 | | CV\_E2CellBodyNucTotalIntenCh1 | 1 | 100 | | CV\_E2CellBodyShapeLWRCh2 | 1 | 100 | | CV\_E2CellBodySpotOverlapAreaCh4 | 1 | 100 | | CV\_E2CellBodySpotOverlapCountCh4 | 1 | 100 | | CV\_E2CellBodySpotTotalIntenCh4 | 1 | 100 | | CV\_E2CellBodySpotTotalIntenRatioCh3Ch2 | 1 | 100 | | CV\_E2NeuriteAvgLengthCh2 | 1 | 100 | | CV\_E2NeuriteDendriteMaxCh2 | 1 | 100 | | CV\_E2NeuriteMaxLengthWithoutBranchesCh2 | 1 | 100 | | CV\_E2NeuriteSpot%OverlapCountCh4 | 1 | 100 | | CV\_E2NeuriteSpotAvgAreaCh4 | 1 | 100 | | CV\_E2NeuriteSpotOverlapAreaCh3 | 1 | 100 | | CV\_E2NeuriteSpotOverlapCountCh3 | 1 | 100 | | CV\_E2NeuriteSpotTotalAreaCh3 | 1 | 100 | | CV\_E2NeuriteSpotTotalAreaRatioCh4Ch3 | 1 | 100 | | CV\_E2NeuriteSpotOverlapAreaCh4 | 1 | 100 | | CV\_E2NeuriteSpotTotalCountCh4 | 1 | 100 | | CV\_E2NeuriteSpotTotalIntenCh3 | 1 | 100 | | MAD\_E2NeuriteSpotTotalIntenCh4 | 1 | 100 | | MAD\_E2NeuriteVarIntenCh2 | 4 | 400 | | MAD\_E3AvgIntenCh3 | 4 | 400 | | MAD\_E3AvgIntenCh4 | 1 | 100 | | MAD\_E3CellBodyAvgIntenCh2 | 4 | 400 | | MAD\_E3CellBodySpot%OverlapAreaCh3 | 1 | 100 | | MAD\_E3CellBodySpotOverlapAreaCh3 | 2.01840225892216 | 201.840225892216 | | MAD\_E3CellBodySpotTotalAreaCh4 | 1 | 100 | | MAD\_E3CellBodySpotTotalCountCh4 | 1 | 100 | | MAD\_E3CellBodySpotTotalIntenCh4 | 1 | 100 | | MAD\_E3CellBodyTotalIntenCh2 | 8.07360903568864 | 807.360903568864 | | MAD\_E3CellBodyVarIntenCh2 | 1 | 100 | | MAD\_E3NeuriteCriticalValueCh2 | 1.42070484581498 | 142.070484581498 | | MAD\_E3NeuriteDendriteMaxCh2 | 1 | 100 | | MAD\_E3NeuriteRamificationIndexCh2 | 1 | 100 | | MAD\_E3NeuriteSpot%OverlapAreaCh3 | 1 | 100 | | MAD\_E3NeuriteSpotAvgAreaCh4 | 1 | 100 | | MAD\_E3NeuriteSpotAvgCountCh4 | 1 | 100 | | MAD\_E3NeuriteSpotTotalAreaCh4 | 1 | 100 | | MAD\_E3NeuriteSpotTotalCountCh3 | 1 | 100 | | MAD\_E3NeuriteSpotTotalIntenCh4 | 1 | 100 | | MAD\_E3NeuriteTotalCountCh2 | 1 | 100 | | MAD\_E3TotalIntenCh3 | 8.07360903568864 | 807.360903568864 | | MAD\_NeuriteAvgIntenCh2 | 4 | 400 | | MAD\_NeuriteSpotAvgAreaCh3 | 2.01840225892216 | 201.840225892216 | | MEAN\_NeuriteSpotTotalIntenCh4 | 1 | 100 | | MEAN\_NeuriteSpotTotalIntenRatioCh4Ch2 | 1 | 100 | | MEAN\_NeuriteTotalIntenCh2 | 8.07360903568864 | 807.360903568864 | | MEAN\_NeuriteWidthCh2 | 1.42070484581498 | 142.070484581498 | | MEDIAN\_BranchPointAvgCountCh2 | 1 | 100 | | MEDIAN\_BranchPointCountPerNeuriteLengthCh2 | 0.703875968992248 | 70.3875968992248 | | MEDIAN\_BranchPointTotalCountCh2 | 1 | 100 | | MEDIAN\_CellBodyAreaCh2 | 2.01840225892216 | 201.840225892216 | | MEDIAN\_CellBodyNucAvgIntenCh1 | 4 | 400 | | MEDIAN\_CellBodyNucCountCh2 | 1 | 100 | | MEDIAN\_CellBodyNucTotalIntenCh1 | 8.07360903568864 | 807.360903568864 | | MEDIAN\_CellBodySpot%OverlapAreaCh4 | 1 | 100 | | MEDIAN\_CellBodySpot%OverlapCountCh3 | 1 | 100 | | MEDIAN\_CellBodySpotOverlapCountCh3 | 1 | 100 | | MEDIAN\_CellBodySpotTotalIntenRatioCh3Ch2 | 1 | 100 | | MEDIAN\_E1AvgIntenCh3 | 4 | 400 | | MEDIAN\_E1BranchPointAvgCountCh2 | 1 | 100 | | MEDIAN\_E1BranchPointAvgDistFromCellBodyCh2 | 1.42070484581498 | 142.070484581498 | | MEDIAN\_E1BranchPointCountPerNeuriteLengthCh2 | 0.703875968992248 | 70.3875968992248 | | MEDIAN\_E1CellBodyNucTotalAreaCh1 | 2.01840225892216 | 201.840225892216 | | MEDIAN\_E1CellBodySpot%OverlapAreaCh3 | 1 | 100 | | MEDIAN\_E1CellBodySpot%OverlapAreaCh4 | 1 | 100 | | MEDIAN\_E1CellBodySpot%OverlapCountCh4 | 1 | 100 | | MEDIAN\_E1CellBodySpotOverlapAreaCh3 | 2.01840225892216 | 201.840225892216 | | %HIGH\_E1NeuriteWidthCh2 | 1 | 100 | | %HIGH\_E1TotalIntenCh4 | 1 | 100 | | %HIGH\_E2AvgIntenCh4 | 1 | 100 | | %HIGH\_E2CellBodyAreaCh2 | 1 | 100 | | %HIGH\_E2CellBodyNucCountCh2 | 1 | 100 | | %HIGH\_E2CellBodyShapeP2ACh2 | 1 | 100 | | %HIGH\_E2CellBodySpotAvgIntenCh3 | 1 | 100 | | %HIGH\_E2CellBodySpotTotalAreaCh3 | 1 | 100 | | %HIGH\_E2CellBodySpotTotalCountCh4 | 1 | 100 | | %HIGH\_E2CellBodySpotTotalIntenCh3 | 1 | 100 | | %HIGH\_E2CrossPointAvgCountCh2 | 1 | 100 | | %HIGH\_E2NeuriteAvgIntenCh2 | 1 | 100 | | %HIGH\_E2NeuriteSpot%OverlapAreaCh4 | 1 | 100 | | %HIGH\_E2NeuriteSpotAvgCountCh4 | 1 | 100 | | %HIGH\_E2NeuriteSpotAvgIntenCh3 | 1 | 100 | | %HIGH\_E2NeuriteSpotTotalAreaCh4 | 1 | 100 | | %HIGH\_E2NeuriteSpotTotalCountCh4 | 1 | 100 | | %HIGH\_E2NeuriteSpotTotalIntenCh3 | 1 | 100 | | %HIGH\_E2NeuriteSpotTotalIntenRatioCh3Ch2 | 1 | 100 | | %HIGH\_E2NeuriteTotalIntenCh2 | 1 | 100 | | %HIGH\_E2NeuriteWidthCh2 | 1 | 100 | | %HIGH\_E2TotalIntenCh4 | 1 | 100 | | %HIGH\_E3CellBodyAreaCh2 | 1 | 100 | | %HIGH\_E3CellBodyShapeLWRCh2 | 1 | 100 | | %HIGH\_E3CellBodyShapeP2ACh2 | 1 | 100 | | %HIGH\_E3CellBodySpot%OverlapAreaCh4 | 1 | 100 | | %HIGH\_E3CellBodySpotAvgIntenCh3 | 1 | 100 | | %HIGH\_E3CellBodySpotOverlapAreaCh4 | 1 | 100 | | %HIGH\_E3CellBodySpotOverlapCountCh3 | 1 | 100 | | CV\_E2NeuriteSpotTotalIntenRatioCh4Ch2 | 1 | 100 | | CV\_E2NeuriteSpotTotalIntenRatioCh4Ch3 | 1 | 100 | | CV\_E2NeuriteTotalCountCh2 | 1 | 100 | | CV\_E2NeuriteTotalLengthCh2 | 1 | 100 | | CV\_E2TotalIntenCh3 | 1 | 100 | | CV\_E3AvgIntenCh3 | 1 | 100 | | CV\_E3BranchPointAvgCountCh2 | 1 | 100 | | CV\_E3BranchPointCountPerNeuriteLengthCh2 | 1 | 100 | | CV\_E3BranchPointTotalCountCh2 | 1 | 100 | | CV\_E3CellBodySpotAvgIntenCh4 | 1 | 100 | | CV\_E3CellBodySpotTotalIntenCh4 | 1 | 100 | | CV\_E3CellBodySpotTotalIntenRatioCh4Ch2 | 1 | 100 | | CV\_E3NeuriteAvgLengthCh2 | 1 | 100 | | CV\_E3NeuriteSpotAvgCountCh3 | 1 | 100 | | CV\_E3NeuriteSpotAvgIntenCh4 | 1 | 100 | | CV\_E3NeuriteSpotTotalAreaCh3 | 1 | 100 | | CV\_E3NeuriteSpotTotalCountCh3 | 1 | 100 | | CV\_E3NeuriteSpotTotalIntenCh4 | 1 | 100 | | CV\_E3NeuriteSpotTotalIntenRatioCh4Ch2 | 1 | 100 | | CV\_E3NeuriteTotalCountCh2 | 1 | 100 | | CV\_E3NeuriteTotalLengthCh2 | 1 | 100 | | CV\_NeuriteCriticalValueCh2 | 1 | 100 | | CV\_NeuriteDendriteMaxCh2 | 1 | 100 | | CV\_E2NeuriteSpotTotalIntenCh4 | 1 | 100 | | CV\_E2NeuriteSpotTotalIntenRatioCh3Ch2 | 1 | 100 | | MAD\_E2NeuriteSpotTotalCountCh4 | 1 | 100 | | MAD\_E2NeuriteSpotTotalIntenCh3 | 8.07360903568864 | 807.360903568864 | | MAD\_E2NeuriteSpotTotalIntenRatioCh3Ch2 | 1 | 100 | | MAD\_E2NeuriteSpotTotalIntenRatioCh4Ch3 | 1 | 100 | | MAD\_E2NeuriteTotalLengthCh2 | 1.42070484581498 | 142.070484581498 | | MAD\_E2NeuriteWidthCh2 | 1.42070484581498 | 142.070484581498 | | MAD\_E2TotalIntenCh3 | 8.07360903568864 | 807.360903568864 | | MAD\_E3BranchPointCountPerNeuriteLengthCh2 | 0.703875968992248 | 70.3875968992248 | | MAD\_E3BranchPointTotalCountCh2 | 1 | 100 | | MAD\_E3CellBodyNucTotalAreaCh1 | 2.01840225892216 | 201.840225892216 | | MAD\_E3CellBodyShapeP2ACh2 | 1 | 100 | | MAD\_E3CellBodySpotOverlapAreaCh4 | 1 | 100 | | MAD\_E3CellBodySpotOverlapCountCh3 | 1 | 100 | | MAD\_E3CellBodySpotTotalIntenRatioCh4Ch2 | 1 | 100 | | MAD\_E3NeuriteAvgIntenCh2 | 4 | 400 | | MAD\_E3NeuriteMaxLengthWithBranchesCh2 | 1.42070484581498 | 142.070484581498 | | MAD\_E3NeuriteSpot%OverlapAreaCh4 | 1 | 100 | | MAD\_E3NeuriteSpot%OverlapCountCh3 | 1 | 100 | | MAD\_E3NeuriteSpotAvgAreaCh3 | 2.01840225892216 | 201.840225892216 | | MAD\_E3NeuriteSpotAvgCountCh3 | 1 | 100 | | MAD\_E3NeuriteSpotAvgIntenCh4 | 1 | 100 | | MAD\_E3NeuriteSpotTotalCountCh4 | 1 | 100 | | MAD\_E3NeuriteSpotTotalIntenRatioCh4Ch3 | 1 | 100 | | MAD\_E3NeuriteTotalLengthCh2 | 1.42070484581498 | 142.070484581498 | | MAD\_NeuriteDendriteMaxCh2 | 1 | 100 | | MAD\_NeuriteMaxLengthWithoutBranchesCh2 | 1.42070484581498 | 142.070484581498 | | MAD\_NeuriteSpotAvgIntenCh3 | 4 | 400 | | MEAN\_NeuriteSpotTotalIntenRatioCh4Ch3 | 1 | 100 | | MEAN\_NeuriteVarIntenCh2 | 4 | 400 | | MEDIAN\_AvgIntenCh3 | 4 | 400 | | MEDIAN\_AvgIntenCh4 | 1 | 100 | | MEDIAN\_BranchPointAvgDistFromCellBodyCh2 | 1.42070484581498 | 142.070484581498 | | MEDIAN\_CellBodyShapeLWRCh2 | 1 | 100 | | MEDIAN\_CellBodySpot%OverlapCountCh4 | 1 | 100 | | MEDIAN\_CellBodySpotOverlapAreaCh3 | 2.01840225892216 | 201.840225892216 | | MEDIAN\_CellBodySpotTotalAreaCh4 | 1 | 100 | | MEDIAN\_CellBodySpotTotalIntenCh3 | 8.07360903568864 | 807.360903568864 | | MEDIAN\_CellBodyVarIntenCh2 | 1 | 100 | | MEDIAN\_CrossPointAvgCountCh2 | 1 | 100 | | MEDIAN\_E1AvgIntenCh4 | 1 | 100 | | MEDIAN\_E1BranchPointTotalCountCh2 | 1 | 100 | | MEDIAN\_E1CellBodyAvgIntenCh2 | 4 | 400 | | MEDIAN\_E1CellBodyNucCountCh2 | 1 | 100 | | MEDIAN\_E1CellBodyNucTotalIntenCh1 | 8.07360903568864 | 807.360903568864 | | MEDIAN\_E1CellBodySpot%OverlapCountCh3 | 1 | 100 | | MEDIAN\_E1CellBodySpotTotalAreaCh4 | 1 | 100 | | MEDIAN\_E1CellBodySpotTotalAreaRatioCh4Ch3 | 1 | 100 | | MEDIAN\_E1CellBodySpotTotalIntenRatioCh3Ch2 | 1 | 100 | | MEDIAN\_E1CrossPointAvgCountCh2 | 1 | 100 | | CV\_E2NeuriteSpotTotalCountCh3 | 1 | 100 | | MAD\_E2NeuriteSpotTotalIntenRatioCh4Ch2 | 1 | 100 | | MAD\_E2NeuriteTotalCountCh2 | 1 | 100 | | MAD\_E2NeuriteTotalIntenCh2 | 8.07360903568864 | 807.360903568864 | | MAD\_E2TotalIntenCh4 | 1 | 100 | | MAD\_E3BranchPointAvgCountCh2 | 1 | 100 | | MAD\_E3BranchPointAvgDistFromCellBodyCh2 | 1.42070484581498 | 142.070484581498 | | MAD\_E3CellBodyAreaCh2 | 2.01840225892216 | 201.840225892216 | | MAD\_E3CellBodyShapeLWRCh2 | 1 | 100 | | MAD\_E3CellBodySpot%OverlapAreaCh4 | 1 | 100 | | MAD\_E3CellBodySpot%OverlapCountCh3 | 1 | 100 | | MAD\_E3CellBodySpotAvgIntenCh4 | 1 | 100 | | MAD\_E3CellBodySpotTotalIntenRatioCh3Ch2 | 1 | 100 | | MAD\_E3CellBodySpotTotalIntenRatioCh4Ch3 | 1 | 100 | | MAD\_E3NeuriteAvgLengthCh2 | 1.42070484581498 | 142.070484581498 | | MAD\_E3NeuriteSpotOverlapAreaCh3 | 2.01840225892216 | 201.840225892216 | | MAD\_E3NeuriteSpotOverlapAreaCh4 | 1 | 100 | | MAD\_E3NeuriteSpotOverlapCountCh3 | 1 | 100 | | MAD\_E3NeuriteSpotTotalAreaCh3 | 2.01840225892216 | 201.840225892216 | | MAD\_E3NeuriteSpotTotalIntenRatioCh4Ch2 | 1 | 100 | | MAD\_E3NeuriteTotalIntenCh2 | 8.07360903568864 | 807.360903568864 | | MAD\_E3NeuriteVarIntenCh2 | 4 | 400 | | MAD\_E3TotalIntenCh4 | 1 | 100 | | MAD\_NeuriteCriticalValueCh2 | 1.42070484581498 | 142.070484581498 | | MAD\_NeuriteRamificationIndexCh2 | 1 | 100 | | MAD\_NeuriteSpot%OverlapCountCh4 | 1 | 100 | | MAD\_NeuriteSpotAvgAreaCh4 | 1 | 100 | | MAD\_NeuriteSpotAvgCountCh3 | 1 | 100 | | MEAN\_NeuriteSpotTotalIntenCh3 | 8.07360903568864 | 807.360903568864 | | MEAN\_NeuriteSpotTotalIntenRatioCh3Ch2 | 1 | 100 | | MEAN\_NeuriteTotalAreaCh2 | 2.01840225892216 | 201.840225892216 | | MEAN\_TotalIntenCh3 | 8.07360903568864 | 807.360903568864 | | MEAN\_TotalIntenCh4 | 1 | 100 | | MEDIAN\_CellBodyNucTotalAreaCh1 | 2.01840225892216 | 201.840225892216 | | MEDIAN\_CellBodyShapeP2ACh2 | 1 | 100 | | MEDIAN\_CellBodySpot%OverlapAreaCh3 | 1 | 100 | | MEDIAN\_CellBodySpotAvgIntenCh3 | 4 | 400 | | MEDIAN\_CellBodySpotOverlapAreaCh4 | 1 | 100 | | MEDIAN\_CellBodySpotOverlapCountCh4 | 1 | 100 | | MEDIAN\_CellBodySpotTotalAreaCh3 | 2.01840225892216 | 201.840225892216 | | MEDIAN\_CellBodySpotTotalCountCh4 | 1 | 100 | | MEDIAN\_CellBodySpotTotalIntenCh4 | 1 | 100 | | MEDIAN\_CellBodyTotalIntenCh2 | 8.07360903568864 | 807.360903568864 | | MEDIAN\_CrossPointTotalCountCh2 | 1 | 100 | | MEDIAN\_E1CellBodyNucAvgIntenCh1 | 4 | 400 | | MEDIAN\_E1CellBodySpotOverlapCountCh3 | 1 | 100 | | MEDIAN\_E1CellBodySpotOverlapCountCh4 | 1 | 100 | | MEDIAN\_E1CellBodySpotTotalCountCh3 | 1 | 100 | | MEDIAN\_E1CellBodySpotTotalCountCh4 | 1 | 100 | | MEDIAN\_E1CellBodySpotTotalIntenRatioCh4Ch3 | 1 | 100 | | MEDIAN\_E1CellBodyTotalIntenCh2 | 8.07360903568864 | 807.360903568864 | | MEDIAN\_E1CellBodySpotOverlapAreaCh4 | 1 | 100 | | %EventType2Neurons | 1 | 100 | | %EventType3Neurons | 1 | 100 | | CellBodySpotOverlapTotalAreaPerWellCh3 | 2.01840225892216 | 201.840225892216 | | CellBodySpotOverlapTotalCountPerFieldCh3 | 1 | 100 | | CellBodySpotOverlapTotalCountPerNeuronCh4 | 1 | 100 | | CellBodySpotTotalAreaPerFieldCh4 | 1 | 100 | | CellBodySpotTotalAreaPerNeuronCh3 | 2.01840225892216 | 201.840225892216 | | CellBodySpotTotalAreaPerWellCh3 | 2.01840225892216 | 201.840225892216 | | CellBodySpotTotalIntenPerWellCh4 | 1 | 100 | | CV\_AvgIntenCh3 | 1 | 100 | | CV\_AvgIntenCh4 | 1 | 100 | | CV\_BranchPointAvgDistFromCellBodyCh2 | 1 | 100 | | CV\_CellBodyAreaCh2 | 1 | 100 | | CV\_CellBodyShapeLWRCh2 | 1 | 100 | | CV\_CellBodyShapeP2ACh2 | 1 | 100 | | CV\_CellBodySpot%OverlapAreaCh3 | 1 | 100 | | CV\_CellBodySpotOverlapAreaCh3 | 1 | 100 | | CV\_CellBodySpotTotalAreaCh4 | 1 | 100 | | CV\_CellBodySpotTotalCountCh4 | 1 | 100 | | CV\_CellBodySpotTotalIntenCh4 | 1 | 100 | | CV\_CellBodyVarIntenCh2 | 1 | 100 | | CV\_CrossPointAvgCountCh2 | 1 | 100 | | CV\_CrossPointTotalCountCh2 | 1 | 100 | | CV\_E1AvgIntenCh4 | 1 | 100 | | CV\_E1CellBodyNucAvgIntenCh1 | 1 | 100 | | CV\_E1CellBodyNucCountCh2 | 1 | 100 | | CV\_E1CellBodyNucTotalIntenCh1 | 1 | 100 | | CV\_E1CellBodySpotTotalAreaCh4 | 1 | 100 | | CV\_E1CellBodySpotTotalCountCh4 | 1 | 100 | | MAD\_E1CellBodyTotalIntenCh2 | 8.07360903568864 | 807.360903568864 | | MAD\_E1NeuriteAvgIntenCh2 | 4 | 400 | | MAD\_E1NeuriteCriticalValueCh2 | 1.42070484581498 | 142.070484581498 | | MAD\_E1NeuriteSpotAvgIntenCh3 | 4 | 400 | | MAD\_E1NeuriteSpotAvgIntenCh4 | 1 | 100 | | MAD\_E1NeuriteSpotOverlapCountCh3 | 1 | 100 | | MAD\_E1NeuriteSpotTotalIntenCh4 | 1 | 100 | | MAD\_E1NeuriteSpotTotalIntenRatioCh3Ch2 | 1 | 100 | | MAD\_E1NeuriteSpotTotalIntenRatioCh4Ch2 | 1 | 100 | | MAD\_E1NeuriteVarIntenCh2 | 4 | 400 | | MAD\_E2CellBodyAvgIntenCh2 | 4 | 400 | | MAD\_E2CellBodyShapeLWRCh2 | 1 | 100 | | MAD\_E2CellBodySpot%OverlapCountCh3 | 1 | 100 | | MAD\_E2CellBodySpotAvgIntenCh4 | 1 | 100 | | MAD\_E2CellBodySpotOverlapCountCh3 | 1 | 100 | | MAD\_E2CellBodySpotTotalAreaCh4 | 1 | 100 | | MAD\_E2CellBodySpotTotalIntenCh4 | 1 | 100 | | MAD\_E2CellBodySpotTotalIntenRatioCh3Ch2 | 1 | 100 | | MAD\_E2CellBodySpotTotalIntenRatioCh4Ch2 | 1 | 100 | | MAD\_E2CellBodyTotalIntenCh2 | 8.07360903568864 | 807.360903568864 | | MAD\_E2CellBodyVarIntenCh2 | 1 | 100 | | MAD\_E2NeuriteCriticalValueCh2 | 1.42070484581498 | 142.070484581498 | | CV\_NeuriteSpot%OverlapCountCh4 | 1 | 100 | | CV\_NeuriteSpotAvgIntenCh3 | 1 | 100 | | CV\_NeuriteSpotOverlapCountCh4 | 1 | 100 | | MAD\_NeuriteSpotOverlapAreaCh3 | 2.01840225892216 | 201.840225892216 | | MAD\_NeuriteSpotTotalCountCh4 | 1 | 100 | | MAD\_NeuriteTotalLengthCh2 | 1.42070484581498 | 142.070484581498 | | MAD\_TotalIntenCh4 | 1 | 100 | | MEAN\_BranchPointAvgCountCh2 | 1 | 100 | | MEAN\_BranchPointTotalCountCh2 | 1 | 100 | | MEAN\_CellBodyNucAvgIntenCh1 | 4 | 400 | | MEAN\_CellBodyNucCountCh2 | 1 | 100 | | MEAN\_CellBodyNucTotalIntenCh1 | 8.07360903568864 | 807.360903568864 | | MEAN\_CellBodySpot%OverlapAreaCh4 | 1 | 100 | | MEAN\_CellBodySpotAvgIntenCh3 | 4 | 400 | | MEAN\_CellBodySpotOverlapAreaCh4 | 1 | 100 | | MEAN\_CellBodySpotOverlapCountCh3 | 1 | 100 | | MEAN\_CellBodySpotTotalIntenRatioCh3Ch2 | 1 | 100 | | MEAN\_E1BranchPointAvgCountCh2 | 1 | 100 | | MEAN\_E1BranchPointCountPerNeuriteLengthCh2 | 0.703875968992248 | 70.3875968992248 | | MEAN\_E1BranchPointTotalCountCh2 | 1 | 100 | | MEAN\_E1CellBodySpot%OverlapAreaCh3 | 1 | 100 | | MEAN\_E1CellBodySpot%OverlapAreaCh4 | 1 | 100 | | MEAN\_E1CellBodySpot%OverlapCountCh3 | 1 | 100 | | MEAN\_E1CellBodySpot%OverlapCountCh4 | 1 | 100 | | MEAN\_E1CellBodySpotOverlapAreaCh3 | 2.01840225892216 | 201.840225892216 | | MEAN\_E1CellBodySpotOverlapAreaCh4 | 1 | 100 | | MEAN\_E1NeuriteAvgLengthCh2 | 1.42070484581498 | 142.070484581498 | | MEAN\_E1NeuriteMaxLengthWithBranchesCh2 | 1.42070484581498 | 142.070484581498 | | MEAN\_E1NeuriteSpot%OverlapAreaCh3 | 1 | 100 | | MEDIAN\_E1NeuriteSpotAvgIntenCh3 | 4 | 400 | | MEDIAN\_E1NeuriteSpotAvgIntenCh4 | 1 | 100 | | MEDIAN\_E1NeuriteSpotTotalIntenCh3 | 8.07360903568864 | 807.360903568864 | | MEDIAN\_E1NeuriteSpotTotalIntenCh4 | 1 | 100 | | MEDIAN\_E1NeuriteSpotTotalIntenRatioCh3Ch2 | 1 | 100 | | MEDIAN\_E1NeuriteSpotTotalIntenRatioCh4Ch2 | 1 | 100 | | MEDIAN\_E1NeuriteVarIntenCh2 | 4 | 400 | | MEDIAN\_E2AvgIntenCh4 | 1 | 100 | | MEDIAN\_E2CellBodyShapeLWRCh2 | 1 | 100 | | MEDIAN\_E2CellBodyShapeP2ACh2 | 1 | 100 | | MEDIAN\_E2CellBodySpotAvgIntenCh3 | 4 | 400 | | MEDIAN\_E2CellBodySpotAvgIntenCh4 | 1 | 100 | | MEDIAN\_E2CellBodySpotOverlapCountCh3 | 1 | 100 | | MEDIAN\_E2CellBodySpotTotalAreaCh4 | 1 | 100 | | MEDIAN\_E2CellBodySpotTotalIntenCh4 | 1 | 100 | | MEDIAN\_E2CellBodySpotTotalIntenRatioCh3Ch2 | 1 | 100 | | MEDIAN\_E2CellBodyTotalIntenCh2 | 8.07360903568864 | 807.360903568864 | | MEDIAN\_E2CellBodyVarIntenCh2 | 1 | 100 | | MEDIAN\_E2NeuriteCriticalValueCh2 | 1.42070484581498 | 142.070484581498 | | MEDIAN\_E2NeuriteDendriteMaxCh2 | 1 | 100 | | MEDIAN\_E2NeuriteSpot%OverlapCountCh3 | 1 | 100 | | MEDIAN\_E2NeuriteSpotAvgAreaCh4 | 1 | 100 | | MEDIAN\_E1CrossPointTotalCountCh2 | 1 | 100 | | %HIGH\_E2BranchPointAvgDistFromCellBodyCh2 | 1 | 100 | | %HIGH\_E2CellBodyAvgIntenCh2 | 1 | 100 | | %HIGH\_E2CellBodyNucTotalIntenCh1 | 1 | 100 | | %HIGH\_E2CellBodyShapeLWRCh2 | 1 | 100 | | %HIGH\_E2CellBodySpot%OverlapCountCh3 | 1 | 100 | | %HIGH\_E2CellBodySpotOverlapAreaCh4 | 1 | 100 | | %HIGH\_E2CellBodySpotTotalIntenCh4 | 1 | 100 | | %HIGH\_E2CellBodySpotTotalIntenRatioCh3Ch2 | 1 | 100 | | %HIGH\_E2NeuriteDendriteMaxCh2 | 1 | 100 | | %HIGH\_E2NeuriteMaxLengthWithoutBranchesCh2 | 1 | 100 | | %HIGH\_E2NeuriteSpot%OverlapCountCh4 | 1 | 100 | | %HIGH\_E2NeuriteSpotAvgAreaCh4 | 1 | 100 | | %HIGH\_E2NeuriteSpotAvgIntenCh4 | 1 | 100 | | %HIGH\_E2NeuriteSpotOverlapAreaCh3 | 1 | 100 | | %HIGH\_E2NeuriteSpotOverlapCountCh3 | 1 | 100 | | %HIGH\_E2NeuriteSpotTotalAreaCh3 | 1 | 100 | | %HIGH\_E2NeuriteVarIntenCh2 | 1 | 100 | | %HIGH\_E3BranchPointAvgDistFromCellBodyCh2 | 1 | 100 | | %HIGH\_E3CellBodyAvgIntenCh2 | 1 | 100 | | %HIGH\_E3CellBodyNucCountCh2 | 1 | 100 | | %HIGH\_E3CellBodyNucTotalIntenCh1 | 1 | 100 | | %HIGH\_E3CellBodySpot%OverlapAreaCh3 | 1 | 100 | | %HIGH\_E3CellBodySpot%OverlapCountCh3 | 1 | 100 | | %HIGH\_E3CellBodySpotOverlapCountCh4 | 1 | 100 | | CV\_E2NeuriteVarIntenCh2 | 1 | 100 | | CV\_E3BranchPointAvgDistFromCellBodyCh2 | 1 | 100 | | CV\_E3CellBodyAvgIntenCh2 | 1 | 100 | | CV\_E3CellBodyNucTotalIntenCh1 | 1 | 100 | | CV\_E3CellBodyShapeLWRCh2 | 1 | 100 | | CV\_E3CellBodySpot%OverlapAreaCh3 | 1 | 100 | | CV\_E3CellBodySpot%OverlapCountCh3 | 1 | 100 | | CV\_E3CellBodySpotOverlapCountCh4 | 1 | 100 | | CV\_E3CellBodySpotTotalAreaCh3 | 1 | 100 | | CV\_E3CellBodySpotTotalCountCh4 | 1 | 100 | | CV\_E3CellBodySpotTotalIntenRatioCh4Ch3 | 1 | 100 | | CV\_E3CellBodyVarIntenCh2 | 1 | 100 | | CV\_E3CrossPointAvgCountCh2 | 1 | 100 | | CV\_E3NeuriteDendriteMaxCh2 | 1 | 100 | | CV\_E3NeuriteMaxLengthWithoutBranchesCh2 | 1 | 100 | | CV\_E3NeuriteSpotAvgAreaCh4 | 1 | 100 | | CV\_E3NeuriteSpotAvgCountCh4 | 1 | 100 | | CV\_E3NeuriteSpotOverlapAreaCh3 | 1 | 100 | | CV\_E3NeuriteSpotTotalAreaRatioCh4Ch3 | 1 | 100 | | CV\_E3NeuriteSpotTotalIntenCh3 | 1 | 100 | | CV\_E3TotalIntenCh3 | 1 | 100 | | CV\_E3TotalIntenCh4 | 1 | 100 | | CV\_NeuriteAvgLengthCh2 | 1 | 100 | | CV\_NeuriteRamificationIndexCh2 | 1 | 100 | | CV\_NeuriteSpot%OverlapAreaCh4 | 1 | 100 | | CV\_NeuriteSpot%OverlapCountCh3 | 1 | 100 | | %HIGH\_CellBodyShapeLWRCh2 | 1 | 100 | | %HIGH\_CellBodyShapeP2ACh2 | 1 | 100 | | %HIGH\_CellBodySpot%OverlapAreaCh3 | 1 | 100 | | %HIGH\_CellBodySpotAvgIntenCh4 | 1 | 100 | | %HIGH\_CellBodySpotTotalAreaCh4 | 1 | 100 | | %HIGH\_CellBodySpotTotalIntenCh4 | 1 | 100 | | %HIGH\_CellBodyTotalIntenCh2 | 1 | 100 | | %HIGH\_CellBodyVarIntenCh2 | 1 | 100 | | %HIGH\_CrossPointAvgCountCh2 | 1 | 100 | | %HIGH\_CrossPointTotalCountCh2 | 1 | 100 | | %HIGH\_E1AvgIntenCh4 | 1 | 100 | | %HIGH\_E1CellBodyAreaCh2 | 1 | 100 | | %HIGH\_E1CellBodyNucAvgIntenCh1 | 1 | 100 | | %HIGH\_E1CellBodyNucCountCh2 | 1 | 100 | | %HIGH\_E1CellBodySpotTotalAreaCh4 | 1 | 100 | | %HIGH\_E1CellBodySpotTotalCountCh3 | 1 | 100 | | %HIGH\_E1CellBodySpotTotalCountCh4 | 1 | 100 | | %HIGH\_E1CellBodySpotTotalIntenCh4 | 1 | 100 | | %HIGH\_E1CrossPointAvgCountCh2 | 1 | 100 | | %HIGH\_E1CrossPointTotalCountCh2 | 1 | 100 | | %HIGH\_E1NeuriteSpotAvgAreaCh3 | 1 | 100 | | %HIGH\_E1NeuriteSpotAvgAreaCh4 | 1 | 100 | | %HIGH\_E1NeuriteSpotAvgCountCh3 | 1 | 100 | | %HIGH\_E1NeuriteSpotAvgCountCh4 | 1 | 100 | | %HIGH\_E1NeuriteSpotTotalAreaCh3 | 1 | 100 | | %HIGH\_E1NeuriteSpotTotalAreaCh4 | 1 | 100 | | %HIGH\_E1NeuriteSpotTotalIntenCh3 | 1 | 100 | | %HIGH\_E1NeuriteTotalAreaCh2 | 1 | 100 | | %HIGH\_E1NeuriteTotalIntenCh2 | 1 | 100 | | CV\_E1NeuriteCriticalValueCh2 | 1 | 100 | | CV\_E1NeuriteDendriteMaxCh2 | 1 | 100 | | CV\_E1NeuriteRamificationIndexCh2 | 1 | 100 | | CV\_E1NeuriteSpot%OverlapCountCh3 | 1 | 100 | | CV\_E1NeuriteSpotOverlapAreaCh3 | 1 | 100 | | CV\_E1NeuriteSpotOverlapCountCh3 | 1 | 100 | | CV\_E1NeuriteSpotTotalAreaRatioCh4Ch3 | 1 | 100 | | CV\_E1NeuriteSpotTotalIntenRatioCh4Ch2 | 1 | 100 | | CV\_E1NeuriteSpotTotalIntenRatioCh4Ch3 | 1 | 100 | | CV\_E1NeuriteTotalCountCh2 | 1 | 100 | | CV\_E2AvgIntenCh3 | 1 | 100 | | CV\_E2BranchPointAvgCountCh2 | 1 | 100 | | CV\_E2BranchPointTotalCountCh2 | 1 | 100 | | CV\_E2CellBodyAvgIntenCh2 | 1 | 100 | | CV\_E2CellBodySpot%OverlapAreaCh3 | 1 | 100 | | CV\_E2CellBodySpot%OverlapCountCh3 | 1 | 100 | | CV\_E2CellBodySpotOverlapAreaCh3 | 1 | 100 | | CV\_E2CellBodySpotTotalAreaRatioCh4Ch3 | 1 | 100 | | CV\_E2CellBodySpotTotalIntenRatioCh4Ch2 | 1 | 100 | | CV\_E2CellBodySpotTotalIntenRatioCh4Ch3 | 1 | 100 | | CV\_E2NeuriteRamificationIndexCh2 | 1 | 100 | | CV\_E2NeuriteSpot%OverlapAreaCh3 | 1 | 100 | | CV\_E2NeuriteSpotAvgIntenCh4 | 1 | 100 | | MAD\_E2NeuriteTotalAreaCh2 | 2.01840225892216 | 201.840225892216 | | MAD\_E3CellBodyNucAvgIntenCh1 | 4 | 400 | | MAD\_E3CellBodyNucCountCh2 | 1 | 100 | | MAD\_E3CellBodyNucTotalIntenCh1 | 8.07360903568864 | 807.360903568864 | | MAD\_E3CellBodySpot%OverlapCountCh4 | 1 | 100 | | MAD\_E3CellBodySpotAvgIntenCh3 | 4 | 400 | | MAD\_E3CellBodySpotOverlapCountCh4 | 1 | 100 | | MAD\_E3CellBodySpotTotalAreaCh3 | 2.01840225892216 | 201.840225892216 | | MAD\_E3CellBodySpotTotalAreaRatioCh4Ch3 | 1 | 100 | | MAD\_E3CellBodySpotTotalCountCh3 | 1 | 100 | | MAD\_E3CellBodySpotTotalIntenCh3 | 8.07360903568864 | 807.360903568864 | | MAD\_E3CrossPointAvgCountCh2 | 1 | 100 | | MAD\_E3CrossPointTotalCountCh2 | 1 | 100 | | MAD\_E3NeuriteMaxLengthWithoutBranchesCh2 | 1.42070484581498 | 142.070484581498 | | MAD\_E3NeuriteSpot%OverlapCountCh4 | 1 | 100 | | MAD\_E3NeuriteSpotAvgIntenCh3 | 4 | 400 | | MAD\_E3NeuriteSpotOverlapCountCh4 | 1 | 100 | | MAD\_E3NeuriteSpotTotalAreaRatioCh4Ch3 | 1 | 100 | | MAD\_E3NeuriteSpotTotalIntenCh3 | 8.07360903568864 | 807.360903568864 | | MAD\_E3NeuriteSpotTotalIntenRatioCh3Ch2 | 1 | 100 | | MAD\_E3NeuriteTotalAreaCh2 | 2.01840225892216 | 201.840225892216 | | MAD\_E3NeuriteWidthCh2 | 1.42070484581498 | 142.070484581498 | | MAD\_NeuriteAvgLengthCh2 | 1.42070484581498 | 142.070484581498 | | MAD\_NeuriteMaxLengthWithBranchesCh2 | 1.42070484581498 | 142.070484581498 | | MAD\_NeuriteSpot%OverlapAreaCh3 | 1 | 100 | | MAD\_NeuriteSpot%OverlapAreaCh4 | 1 | 100 | | MAD\_NeuriteSpot%OverlapCountCh3 | 1 | 100 | | MAD\_NeuriteSpotAvgCountCh4 | 1 | 100 | | MEAN\_NeuriteSpotTotalCountCh4 | 1 | 100 | | MEAN\_NeuriteTotalCountCh2 | 1 | 100 | | MEAN\_NeuriteTotalLengthCh2 | 1.42070484581498 | 142.070484581498 | | MEDIAN\_CellBodyAvgIntenCh2 | 4 | 400 | | MEDIAN\_CellBodySpotAvgIntenCh4 | 1 | 100 | | MEDIAN\_CellBodySpotTotalAreaRatioCh4Ch3 | 1 | 100 | | MEDIAN\_CellBodySpotTotalCountCh3 | 1 | 100 | | MEDIAN\_CellBodySpotTotalIntenRatioCh4Ch2 | 1 | 100 | | MEDIAN\_CellBodySpotTotalIntenRatioCh4Ch3 | 1 | 100 | | MEDIAN\_E1CellBodyAreaCh2 | 2.01840225892216 | 201.840225892216 | | MEDIAN\_E1CellBodyShapeLWRCh2 | 1 | 100 | | MEDIAN\_E1CellBodyShapeP2ACh2 | 1 | 100 | | MEDIAN\_E1CellBodySpotAvgIntenCh3 | 4 | 400 | | MEDIAN\_E1CellBodySpotAvgIntenCh4 | 1 | 100 | | MEDIAN\_E1CellBodySpotTotalAreaCh3 | 2.01840225892216 | 201.840225892216 | | MEDIAN\_E1CellBodySpotTotalIntenCh3 | 8.07360903568864 | 807.360903568864 | | MEDIAN\_E1CellBodySpotTotalIntenCh4 | 1 | 100 | | MEDIAN\_E1CellBodySpotTotalIntenRatioCh4Ch2 | 1 | 100 | | MEDIAN\_E1CellBodyVarIntenCh2 | 1 | 100 | | MEDIAN\_E1NeuriteAvgIntenCh2 | 4 | 400 | | MEDIAN\_E1NeuriteCriticalValueCh2 | 1.42070484581498 | 142.070484581498 | | MEDIAN\_E1NeuriteSpotAvgAreaCh4 | 1 | 100 | | MAD\_E2NeuriteDendriteMaxCh2 | 1 | 100 | | MAD\_E2NeuriteSpot%OverlapCountCh3 | 1 | 100 | | MAD\_E2NeuriteSpotAvgAreaCh4 | 1 | 100 | | MAD\_E2NeuriteSpotAvgIntenCh4 | 1 | 100 | | MAD\_E2NeuriteSpotOverlapAreaCh3 | 2.01840225892216 | 201.840225892216 | | MAD\_E2NeuriteSpotOverlapCountCh3 | 1 | 100 | | MEAN\_E1NeuriteSpotAvgAreaCh3 | 2.01840225892216 | 201.840225892216 | | MEAN\_E1NeuriteSpotAvgAreaCh4 | 1 | 100 | | MEAN\_E1NeuriteSpotAvgIntenCh3 | 4 | 400 | | MEAN\_E1NeuriteSpotAvgIntenCh4 | 1 | 100 | | MEAN\_E1NeuriteSpotTotalIntenCh3 | 8.07360903568864 | 807.360903568864 | | MEAN\_E1NeuriteSpotTotalIntenCh4 | 1 | 100 | | MEAN\_E1NeuriteTotalIntenCh2 | 8.07360903568864 | 807.360903568864 | | MEAN\_E1NeuriteVarIntenCh2 | 4 | 400 | | MEAN\_E2AvgIntenCh4 | 1 | 100 | | MEAN\_E2CellBodyShapeLWRCh2 | 1 | 100 | | MEAN\_E2CellBodyShapeP2ACh2 | 1 | 100 | | MEAN\_E2CellBodySpotAvgIntenCh3 | 4 | 400 | | MEAN\_E2CellBodySpotOverlapCountCh3 | 1 | 100 | | MEAN\_E2CellBodySpotTotalAreaCh4 | 1 | 100 | | MEAN\_E2CellBodySpotTotalCountCh4 | 1 | 100 | | MEAN\_E2CellBodySpotTotalIntenCh3 | 8.07360903568864 | 807.360903568864 | | MEAN\_E2CellBodySpotTotalIntenCh4 | 1 | 100 | | MEAN\_E2CellBodySpotTotalIntenRatioCh3Ch2 | 1 | 100 | | MEAN\_E2CellBodyTotalIntenCh2 | 8.07360903568864 | 807.360903568864 | | MEAN\_E2CellBodyVarIntenCh2 | 1 | 100 | | MEAN\_E2NeuriteAvgIntenCh2 | 4 | 400 | | MEAN\_E2NeuriteCriticalValueCh2 | 1.42070484581498 | 142.070484581498 | | MEAN\_E2NeuriteSpot%OverlapCountCh3 | 1 | 100 | | MEAN\_E2NeuriteSpotAvgAreaCh4 | 1 | 100 | | MEAN\_E2NeuriteSpotAvgCountCh4 | 1 | 100 | | MEAN\_E2NeuriteSpotOverlapCountCh3 | 1 | 100 | | MEAN\_E2NeuriteSpotTotalAreaCh4 | 1 | 100 | | MEAN\_E2NeuriteSpotTotalIntenRatioCh3Ch2 | 1 | 100 | | MEDIAN\_E3BranchPointAvgDistFromCellBodyCh2 | 1.42070484581498 | 142.070484581498 | | MEDIAN\_E3CellBodyNucAvgIntenCh1 | 4 | 400 | | MEDIAN\_E3CellBodyNucTotalAreaCh1 | 2.01840225892216 | 201.840225892216 | | MEDIAN\_E3CellBodyNucTotalIntenCh1 | 8.07360903568864 | 807.360903568864 | | MEDIAN\_E3CellBodySpot%OverlapCountCh4 | 1 | 100 | | MEDIAN\_E3CellBodySpotOverlapCountCh4 | 1 | 100 | | MEDIAN\_E3CellBodySpotTotalAreaCh3 | 2.01840225892216 | 201.840225892216 | | MEDIAN\_E3CellBodySpotTotalAreaRatioCh4Ch3 | 1 | 100 | | MEDIAN\_E3CellBodySpotTotalCountCh3 | 1 | 100 | | MEDIAN\_E3CellBodySpotTotalIntenCh3 | 8.07360903568864 | 807.360903568864 | | MEDIAN\_E3CrossPointAvgCountCh2 | 1 | 100 | | MEDIAN\_E3CrossPointTotalCountCh2 | 1 | 100 | | MEDIAN\_E3NeuriteMaxLengthWithoutBranchesCh2 | 1.42070484581498 | 142.070484581498 | | MEDIAN\_E3NeuriteSpotAvgAreaCh3 | 2.01840225892216 | 201.840225892216 | | MEDIAN\_E3NeuriteSpotAvgIntenCh3 | 4 | 400 | | MEDIAN\_E3NeuriteSpotOverlapCountCh4 | 1 | 100 | | MEDIAN\_E3NeuriteSpotTotalAreaRatioCh4Ch3 | 1 | 100 | | CV\_NeuriteSpotAvgAreaCh3 | 1 | 100 | | CV\_NeuriteSpotAvgCountCh3 | 1 | 100 | | CV\_NeuriteSpotAvgIntenCh4 | 1 | 100 | | MAD\_NeuriteSpotAvgIntenCh4 | 1 | 100 | | MAD\_NeuriteSpotTotalIntenRatioCh3Ch2 | 1 | 100 | | MAD\_NeuriteTotalAreaCh2 | 2.01840225892216 | 201.840225892216 | | MAD\_NeuriteTotalCountCh2 | 1 | 100 | | MAD\_TotalIntenCh3 | 8.07360903568864 | 807.360903568864 | | MEAN\_BranchPointCountPerNeuriteLengthCh2 | 0.703875968992248 | 70.3875968992248 | | MEAN\_CellBodyNucTotalAreaCh1 | 2.01840225892216 | 201.840225892216 | | MEAN\_CellBodyShapeP2ACh2 | 1 | 100 | | MEAN\_CellBodySpot%OverlapAreaCh3 | 1 | 100 | | MEAN\_CellBodySpot%OverlapCountCh3 | 1 | 100 | | MEAN\_CellBodySpotOverlapCountCh4 | 1 | 100 | | MEAN\_CellBodySpotTotalAreaCh3 | 2.01840225892216 | 201.840225892216 | | MEAN\_CellBodySpotTotalCountCh4 | 1 | 100 | | MEAN\_CellBodySpotTotalIntenCh4 | 1 | 100 | | MEAN\_CrossPointTotalCountCh2 | 1 | 100 | | MEAN\_E1CellBodyNucAvgIntenCh1 | 4 | 400 | | MEAN\_E1CellBodyNucTotalAreaCh1 | 2.01840225892216 | 201.840225892216 | | MEAN\_E1CellBodySpotAvgIntenCh3 | 4 | 400 | | MEAN\_E1CellBodySpotOverlapCountCh3 | 1 | 100 | | MEAN\_E1CellBodySpotOverlapCountCh4 | 1 | 100 | | MEAN\_E1CellBodySpotTotalCountCh4 | 1 | 100 | | MEAN\_E1CellBodySpotTotalIntenRatioCh4Ch3 | 1 | 100 | | MEAN\_E1CellBodyTotalIntenCh2 | 8.07360903568864 | 807.360903568864 | | MEAN\_E1CrossPointTotalCountCh2 | 1 | 100 | | MEAN\_E1NeuriteCriticalValueCh2 | 1.42070484581498 | 142.070484581498 | | MEAN\_E1NeuriteRamificationIndexCh2 | 1 | 100 | | MEDIAN\_E1NeuriteSpotAvgCountCh4 | 1 | 100 | | MEDIAN\_E1NeuriteSpotTotalAreaRatioCh4Ch3 | 1 | 100 | | MEDIAN\_E1NeuriteTotalAreaCh2 | 2.01840225892216 | 201.840225892216 | | MEDIAN\_E1NeuriteTotalCountCh2 | 1 | 100 | | MEDIAN\_E1NeuriteTotalIntenCh2 | 8.07360903568864 | 807.360903568864 | | MEDIAN\_E1TotalIntenCh4 | 1 | 100 | | MEDIAN\_E2AvgIntenCh3 | 4 | 400 | | MEDIAN\_E2BranchPointAvgCountCh2 | 1 | 100 | | MEDIAN\_E2CellBodyAreaCh2 | 2.01840225892216 | 201.840225892216 | | MEDIAN\_E2CellBodyNucAvgIntenCh1 | 4 | 400 | | MEDIAN\_E2CellBodySpot%OverlapAreaCh4 | 1 | 100 | | MEDIAN\_E2CellBodySpotOverlapAreaCh3 | 2.01840225892216 | 201.840225892216 | | MEDIAN\_E2CellBodySpotTotalCountCh3 | 1 | 100 | | MEDIAN\_E2CellBodySpotTotalCountCh4 | 1 | 100 | | MEDIAN\_E2CellBodySpotTotalIntenCh3 | 8.07360903568864 | 807.360903568864 | | MEDIAN\_E2CellBodySpotTotalIntenRatioCh4Ch3 | 1 | 100 | | MEDIAN\_E2NeuriteRamificationIndexCh2 | 1 | 100 | | MEDIAN\_E2NeuriteSpot%OverlapAreaCh3 | 1 | 100 | | MEDIAN\_E2NeuriteSpotAvgAreaCh3 | 2.01840225892216 | 201.840225892216 | | MEDIAN\_E2NeuriteSpotAvgCountCh4 | 1 | 100 | | MEDIAN\_E2NeuriteSpotAvgIntenCh3 | 4 | 400 | | MEDIAN\_E2NeuriteSpotOverlapAreaCh4 | 1 | 100 | | NeuriteSpotTotalIntenPerNeuronCh4 | 1 | 100 | | %HIGH\_AvgIntenCh3 | 1 | 100 | | %HIGH\_BranchPointAvgDistFromCellBodyCh2 | 1 | 100 | | CellBodySpotOverlapTotalAreaPerWellCh4 | 1 | 100 | | CellBodySpotOverlapTotalCountPerFieldCh4 | 1 | 100 | | CellBodySpotOverlapTotalCountPerNeuronCh3 | 1 | 100 | | CellBodySpotTotalAreaPerFieldCh3 | 2.01840225892216 | 201.840225892216 | | CellBodySpotTotalAreaPerNeuronCh4 | 1 | 100 | | CellBodySpotTotalAreaPerWellCh4 | 1 | 100 | | CellBodySpotTotalCountPerNeuronCh3 | 1 | 100 | | CV\_CellBodyNucTotalAreaCh1 | 1 | 100 | | CV\_CellBodySpot%OverlapCountCh4 | 1 | 100 | | CV\_CellBodySpotAvgIntenCh3 | 1 | 100 | | CV\_CellBodySpotOverlapCountCh4 | 1 | 100 | | CV\_CellBodySpotTotalAreaCh3 | 1 | 100 | | CV\_CellBodySpotTotalAreaRatioCh4Ch3 | 1 | 100 | | CV\_CellBodySpotTotalIntenCh3 | 1 | 100 | | CV\_E1BranchPointTotalCountCh2 | 1 | 100 | | CV\_E1CellBodyAvgIntenCh2 | 1 | 100 | | CV\_E1CellBodySpot%OverlapCountCh3 | 1 | 100 | | CV\_E1CellBodySpotOverlapCountCh3 | 1 | 100 | | CV\_E1CellBodySpotOverlapCountCh4 | 1 | 100 | | CV\_E1CellBodySpotTotalAreaRatioCh4Ch3 | 1 | 100 | | CV\_E1CellBodySpotTotalIntenRatioCh3Ch2 | 1 | 100 | | CV\_E1CellBodySpotTotalIntenRatioCh4Ch3 | 1 | 100 | | CV\_E1CellBodyTotalIntenCh2 | 1 | 100 | | MAD\_E1CrossPointTotalCountCh2 | 1 | 100 | | MAD\_E1NeuriteAvgLengthCh2 | 1.42070484581498 | 142.070484581498 | | MAD\_E1NeuriteMaxLengthWithBranchesCh2 | 1.42070484581498 | 142.070484581498 | | MAD\_E1NeuriteMaxLengthWithoutBranchesCh2 | 1.42070484581498 | 142.070484581498 | | MAD\_E1NeuriteSpot%OverlapAreaCh4 | 1 | 100 | | MAD\_E1NeuriteSpot%OverlapCountCh4 | 1 | 100 | | MAD\_E1NeuriteSpotAvgCountCh3 | 1 | 100 | | MAD\_E1NeuriteSpotOverlapAreaCh4 | 1 | 100 | | MAD\_E1NeuriteSpotTotalCountCh3 | 1 | 100 | | MAD\_E1NeuriteSpotTotalCountCh4 | 1 | 100 | | MAD\_E1TotalIntenCh3 | 8.07360903568864 | 807.360903568864 | | MAD\_E2BranchPointAvgDistFromCellBodyCh2 | 1.42070484581498 | 142.070484581498 | | MAD\_E2BranchPointCountPerNeuriteLengthCh2 | 0.703875968992248 | 70.3875968992248 | | MAD\_E2CellBodyNucAvgIntenCh1 | 4 | 400 | | MAD\_E2CellBodyNucTotalAreaCh1 | 2.01840225892216 | 201.840225892216 | | MAD\_E2CellBodyNucTotalIntenCh1 | 8.07360903568864 | 807.360903568864 | | MAD\_E2CellBodySpot%OverlapAreaCh4 | 1 | 100 | | MAD\_E2CellBodySpot%OverlapCountCh4 | 1 | 100 | | MAD\_E2CellBodySpotOverlapAreaCh4 | 1 | 100 | | MAD\_E2CellBodySpotTotalCountCh3 | 1 | 100 | | MAD\_E2CrossPointAvgCountCh2 | 1 | 100 | | MAD\_E2CrossPointTotalCountCh2 | 1 | 100 | | MAD\_E2NeuriteMaxLengthWithBranchesCh2 | 1.42070484581498 | 142.070484581498 | | MAD\_E2NeuriteMaxLengthWithoutBranchesCh2 | 1.42070484581498 | 142.070484581498 | | MAD\_E2NeuriteSpot%OverlapCountCh4 | 1 | 100 | | MEDIAN\_E2NeuriteSpotTotalIntenRatioCh4Ch2 | 1 | 100 | | MEDIAN\_E2NeuriteTotalCountCh2 | 1 | 100 | | %HIGH\_AvgIntenCh4 | 1 | 100 | | %HIGH\_BranchPointAvgCountCh2 | 1 | 100 | | %HIGH\_CellBodyAreaCh2 | 1 | 100 | | %HIGH\_CellBodyNucAvgIntenCh1 | 1 | 100 | | CellBodySpotAvgIntenPerFieldCh4 | 1 | 100 | | CellBodySpotOverlapTotalAreaPerFieldCh3 | 2.01840225892216 | 201.840225892216 | | CellBodySpotOverlapTotalAreaPerNeuronCh3 | 2.01840225892216 | 201.840225892216 | | CellBodySpotOverlapTotalCountPerWellCh4 | 1 | 100 | | CellBodySpotTotalCountPerFieldCh3 | 1 | 100 | | CellBodySpotTotalCountPerWellCh3 | 1 | 100 | | CellBodySpotTotalIntenPerFieldCh4 | 1 | 100 | | CellBodySpotTotalIntenPerNeuronCh4 | 1 | 100 | | CV\_BranchPointAvgCountCh2 | 1 | 100 | | CV\_CellBodyNucAvgIntenCh1 | 1 | 100 | | CV\_CellBodySpotOverlapAreaCh4 | 1 | 100 | | CV\_CellBodySpotOverlapCountCh3 | 1 | 100 | | CV\_CellBodySpotTotalCountCh3 | 1 | 100 | | CV\_CellBodySpotTotalIntenRatioCh4Ch2 | 1 | 100 | | CV\_CellBodyTotalIntenCh2 | 1 | 100 | | CV\_E1AvgIntenCh3 | 1 | 100 | | CV\_E1BranchPointAvgCountCh2 | 1 | 100 | | CV\_E1BranchPointAvgDistFromCellBodyCh2 | 1 | 100 | | CV\_E1CellBodyAreaCh2 | 1 | 100 | | CV\_E1CellBodyShapeLWRCh2 | 1 | 100 | | CV\_E1CellBodySpotOverlapAreaCh3 | 1 | 100 | | CV\_E1CellBodySpotOverlapAreaCh4 | 1 | 100 | | CV\_E1CellBodySpotTotalCountCh3 | 1 | 100 | | CV\_E1CellBodySpotTotalIntenCh3 | 1 | 100 | | CV\_E1CellBodySpotTotalIntenCh4 | 1 | 100 | | CV\_E1CellBodySpotTotalIntenRatioCh4Ch2 | 1 | 100 | | CV\_E1CellBodyVarIntenCh2 | 1 | 100 | | MAD\_E1CellBodyVarIntenCh2 | 1 | 100 | | MAD\_E1NeuriteRamificationIndexCh2 | 1 | 100 | | MAD\_E1NeuriteSpot%OverlapAreaCh3 | 1 | 100 | | MAD\_E1NeuriteSpot%OverlapCountCh3 | 1 | 100 | | MAD\_E1NeuriteSpotAvgAreaCh3 | 2.01840225892216 | 201.840225892216 | | MAD\_E1NeuriteSpotAvgAreaCh4 | 1 | 100 | | MAD\_E1NeuriteSpotAvgCountCh4 | 1 | 100 | | MAD\_E1NeuriteSpotTotalAreaRatioCh4Ch3 | 1 | 100 | | MAD\_E1NeuriteTotalAreaCh2 | 2.01840225892216 | 201.840225892216 | | MAD\_E1NeuriteTotalCountCh2 | 1 | 100 | | MAD\_E1NeuriteTotalIntenCh2 | 8.07360903568864 | 807.360903568864 | | MAD\_E1TotalIntenCh4 | 1 | 100 | | MAD\_E2AvgIntenCh3 | 4 | 400 | | MAD\_E2BranchPointAvgCountCh2 | 1 | 100 | | MAD\_E2CellBodyAreaCh2 | 2.01840225892216 | 201.840225892216 | | MAD\_E2CellBodySpotOverlapAreaCh3 | 2.01840225892216 | 201.840225892216 | | MAD\_E2CellBodySpotOverlapCountCh4 | 1 | 100 | | MAD\_E2CellBodySpotTotalCountCh4 | 1 | 100 | | MAD\_E2CellBodySpotTotalIntenCh3 | 8.07360903568864 | 807.360903568864 | | MAD\_E2CellBodySpotTotalIntenRatioCh4Ch3 | 1 | 100 | | MAD\_E2NeuriteAvgLengthCh2 | 1.42070484581498 | 142.070484581498 | | MAD\_E2NeuriteRamificationIndexCh2 | 1 | 100 | | MAD\_E2NeuriteSpot%OverlapAreaCh3 | 1 | 100 | | MAD\_E2NeuriteSpotAvgCountCh4 | 1 | 100 | | MAD\_E2NeuriteSpotAvgIntenCh3 | 4 | 400 | | MAD\_E2NeuriteSpotTotalAreaRatioCh4Ch3 | 1 | 100 | | MAD\_E2NeuriteSpotTotalCountCh3 | 1 | 100 | | MEAN\_E1NeuriteSpot%OverlapCountCh3 | 1 | 100 | | MEAN\_E1NeuriteSpotAvgCountCh3 | 1 | 100 | | MEAN\_E1NeuriteSpotAvgCountCh4 | 1 | 100 | | MEAN\_E1NeuriteSpotTotalAreaRatioCh4Ch3 | 1 | 100 | | MEAN\_E1NeuriteSpotTotalIntenRatioCh3Ch2 | 1 | 100 | | MEAN\_E1NeuriteSpotTotalIntenRatioCh4Ch2 | 1 | 100 | | MEAN\_E1NeuriteTotalAreaCh2 | 2.01840225892216 | 201.840225892216 | | MEAN\_E1NeuriteTotalCountCh2 | 1 | 100 | | MEAN\_E1TotalIntenCh4 | 1 | 100 | | MEAN\_E2CellBodyAreaCh2 | 2.01840225892216 | 201.840225892216 | | MEAN\_E2CellBodyNucAvgIntenCh1 | 4 | 400 | | MEAN\_E2CellBodySpot%OverlapAreaCh4 | 1 | 100 | | MEAN\_E2CellBodySpotAvgIntenCh4 | 1 | 100 | | MEAN\_E2CellBodySpotOverlapAreaCh3 | 2.01840225892216 | 201.840225892216 | | MEAN\_E2CellBodySpotTotalCountCh3 | 1 | 100 | | MEAN\_E2CellBodySpotTotalIntenRatioCh4Ch3 | 1 | 100 | | MEAN\_E2CrossPointTotalCountCh2 | 1 | 100 | | MEAN\_E2NeuriteRamificationIndexCh2 | 1 | 100 | | MEAN\_E2NeuriteSpot%OverlapAreaCh3 | 1 | 100 | | MEAN\_E2NeuriteSpotAvgAreaCh3 | 2.01840225892216 | 201.840225892216 | | MEAN\_E2NeuriteSpotAvgIntenCh3 | 4 | 400 | | MEAN\_E2NeuriteSpotOverlapAreaCh4 | 1 | 100 | | MEAN\_E2NeuriteSpotTotalIntenCh4 | 1 | 100 | | MEAN\_E2NeuriteSpotTotalIntenRatioCh4Ch2 | 1 | 100 | | MEAN\_E2NeuriteTotalAreaCh2 | 2.01840225892216 | 201.840225892216 | | MEAN\_E2NeuriteTotalCountCh2 | 1 | 100 | | MEAN\_E2NeuriteTotalIntenCh2 | 8.07360903568864 | 807.360903568864 | | MEDIAN\_E3AvgIntenCh3 | 4 | 400 | | MEDIAN\_E3BranchPointAvgCountCh2 | 1 | 100 | | MEDIAN\_E3CellBodyAreaCh2 | 2.01840225892216 | 201.840225892216 | | MEDIAN\_E3CellBodySpot%OverlapAreaCh4 | 1 | 100 | | MEDIAN\_E3CellBodySpotAvgIntenCh4 | 1 | 100 | | MEDIAN\_E3CellBodySpotTotalIntenRatioCh3Ch2 | 1 | 100 | | MEDIAN\_E3CellBodySpotTotalIntenRatioCh4Ch3 | 1 | 100 | | MEDIAN\_E3NeuriteAvgLengthCh2 | 1.42070484581498 | 142.070484581498 | | MEDIAN\_E3NeuriteSpotOverlapAreaCh4 | 1 | 100 | | MEDIAN\_E3NeuriteSpotOverlapCountCh3 | 1 | 100 | | MEDIAN\_E3NeuriteSpotTotalAreaCh3 | 2.01840225892216 | 201.840225892216 | | MEDIAN\_E3NeuriteSpotTotalCountCh3 | 1 | 100 | | MEDIAN\_E3NeuriteSpotTotalIntenCh4 | 1 | 100 | | MEDIAN\_E3NeuriteSpotTotalIntenRatioCh4Ch2 | 1 | 100 | | MEDIAN\_E3NeuriteTotalCountCh2 | 1 | 100 | | MEDIAN\_E3NeuriteTotalIntenCh2 | 8.07360903568864 | 807.360903568864 | | MEDIAN\_E3NeuriteSpotTotalIntenCh3 | 8.07360903568864 | 807.360903568864 | | MEDIAN\_E3NeuriteSpotTotalIntenRatioCh4Ch3 | 1 | 100 | | %HIGH\_E2AvgIntenCh3 | 1 | 100 | | %HIGH\_E2BranchPointAvgCountCh2 | 1 | 100 | | %HIGH\_E2BranchPointTotalCountCh2 | 1 | 100 | | %HIGH\_E2CellBodySpot%OverlapAreaCh3 | 1 | 100 | | %HIGH\_E2CellBodySpotOverlapAreaCh3 | 1 | 100 | | %HIGH\_E2CellBodySpotOverlapCountCh4 | 1 | 100 | | %HIGH\_E2CellBodySpotTotalAreaRatioCh4Ch3 | 1 | 100 | | %HIGH\_E2CellBodySpotTotalIntenRatioCh4Ch2 | 1 | 100 | | %HIGH\_E2CellBodySpotTotalIntenRatioCh4Ch3 | 1 | 100 | | %HIGH\_E2NeuriteAvgLengthCh2 | 1 | 100 | | %HIGH\_E2NeuriteRamificationIndexCh2 | 1 | 100 | | %HIGH\_E2NeuriteSpot%OverlapAreaCh3 | 1 | 100 | | %HIGH\_E2NeuriteSpotTotalAreaRatioCh4Ch3 | 1 | 100 | | %HIGH\_E2NeuriteSpotTotalCountCh3 | 1 | 100 | | %HIGH\_E2NeuriteSpotTotalIntenRatioCh4Ch2 | 1 | 100 | | %HIGH\_E2NeuriteSpotTotalIntenRatioCh4Ch3 | 1 | 100 | | %HIGH\_E2NeuriteTotalCountCh2 | 1 | 100 | | %HIGH\_E2NeuriteTotalLengthCh2 | 1 | 100 | | %HIGH\_E2TotalIntenCh3 | 1 | 100 | | %HIGH\_E3BranchPointAvgCountCh2 | 1 | 100 | | %HIGH\_E3BranchPointCountPerNeuriteLengthCh2 | 1 | 100 | | %HIGH\_E3BranchPointTotalCountCh2 | 1 | 100 | | %HIGH\_E3CellBodyNucTotalAreaCh1 | 1 | 100 | | %HIGH\_E3CellBodySpotAvgIntenCh4 | 1 | 100 | | CV\_E2NeuriteTotalAreaCh2 | 1 | 100 | | CV\_E2NeuriteTotalIntenCh2 | 1 | 100 | | CV\_E2NeuriteWidthCh2 | 1 | 100 | | CV\_E2TotalIntenCh4 | 1 | 100 | | CV\_E3CellBodyAreaCh2 | 1 | 100 | | CV\_E3CellBodyNucCountCh2 | 1 | 100 | | CV\_E3CellBodyShapeP2ACh2 | 1 | 100 | | CV\_E3CellBodySpot%OverlapAreaCh4 | 1 | 100 | | CV\_E3CellBodySpotAvgIntenCh3 | 1 | 100 | | CV\_E3CellBodySpotOverlapAreaCh4 | 1 | 100 | | CV\_E3CellBodySpotOverlapCountCh3 | 1 | 100 | | CV\_E3CellBodySpotTotalIntenRatioCh3Ch2 | 1 | 100 | | CV\_E3NeuriteAvgIntenCh2 | 1 | 100 | | CV\_E3NeuriteSpot%OverlapAreaCh4 | 1 | 100 | | CV\_E3NeuriteSpot%OverlapCountCh3 | 1 | 100 | | CV\_E3NeuriteSpot%OverlapCountCh4 | 1 | 100 | | CV\_E3NeuriteSpotOverlapAreaCh4 | 1 | 100 | | CV\_E3NeuriteSpotOverlapCountCh3 | 1 | 100 | | CV\_E3NeuriteSpotTotalCountCh4 | 1 | 100 | | CV\_E3NeuriteSpotTotalIntenRatioCh3Ch2 | 1 | 100 | | CV\_E3NeuriteTotalIntenCh2 | 1 | 100 | | CV\_E3NeuriteVarIntenCh2 | 1 | 100 | | CV\_E3NeuriteWidthCh2 | 1 | 100 | | CV\_NeuriteMaxLengthWithBranchesCh2 | 1 | 100 | | CV\_NeuriteMaxLengthWithoutBranchesCh2 | 1 | 100 | | MAD\_E2NeuriteSpotAvgAreaCh3 | 2.01840225892216 | 201.840225892216 | | MAD\_E2NeuriteSpotAvgCountCh3 | 1 | 100 | | MAD\_E2NeuriteSpotOverlapAreaCh4 | 1 | 100 | | MAD\_E2NeuriteSpotOverlapCountCh4 | 1 | 100 | | MAD\_E2NeuriteSpotTotalAreaCh3 | 2.01840225892216 | 201.840225892216 | | MEAN\_E1NeuriteSpot%OverlapCountCh4 | 1 | 100 | | MEAN\_E1NeuriteSpotOverlapAreaCh3 | 2.01840225892216 | 201.840225892216 | | MEAN\_E1NeuriteSpotOverlapAreaCh4 | 1 | 100 | | MEAN\_E1NeuriteSpotOverlapCountCh4 | 1 | 100 | | MEAN\_E1NeuriteTotalLengthCh2 | 1.42070484581498 | 142.070484581498 | | MEAN\_E1TotalIntenCh3 | 8.07360903568864 | 807.360903568864 | | MEAN\_E2AvgIntenCh3 | 4 | 400 | | MEAN\_E2BranchPointAvgCountCh2 | 1 | 100 | | MEAN\_E2BranchPointAvgDistFromCellBodyCh2 | 1.42070484581498 | 142.070484581498 | | MEAN\_E2BranchPointCountPerNeuriteLengthCh2 | 0.703875968992248 | 70.3875968992248 | | MEAN\_E2CellBodyNucTotalAreaCh1 | 2.01840225892216 | 201.840225892216 | | MEAN\_E2CellBodySpot%OverlapCountCh4 | 1 | 100 | | MEAN\_E2CellBodySpotOverlapCountCh4 | 1 | 100 | | MEAN\_E2CellBodySpotTotalAreaRatioCh4Ch3 | 1 | 100 | | MEAN\_E2NeuriteAvgLengthCh2 | 1.42070484581498 | 142.070484581498 | | MEAN\_E2NeuriteMaxLengthWithBranchesCh2 | 1.42070484581498 | 142.070484581498 | | MEAN\_E2NeuriteMaxLengthWithoutBranchesCh2 | 1.42070484581498 | 142.070484581498 | | MEAN\_E2NeuriteSpot%OverlapCountCh4 | 1 | 100 | | MEAN\_E2NeuriteSpotAvgCountCh3 | 1 | 100 | | MEAN\_E2NeuriteSpotOverlapCountCh4 | 1 | 100 | | MEAN\_E2NeuriteSpotTotalAreaCh3 | 2.01840225892216 | 201.840225892216 | | MEAN\_E2NeuriteSpotTotalAreaRatioCh4Ch3 | 1 | 100 | | MEAN\_E2NeuriteSpotTotalCountCh3 | 1 | 100 | | MEDIAN\_E3AvgIntenCh4 | 1 | 100 | | MEDIAN\_E3CellBodyAvgIntenCh2 | 4 | 400 | | MEDIAN\_E3CellBodyShapeLWRCh2 | 1 | 100 | | MEDIAN\_E3CellBodySpot%OverlapAreaCh3 | 1 | 100 | | MEDIAN\_E3CellBodySpot%OverlapCountCh3 | 1 | 100 | | MEDIAN\_E3CellBodySpotOverlapAreaCh3 | 2.01840225892216 | 201.840225892216 | | MEDIAN\_E3CellBodySpotTotalAreaCh4 | 1 | 100 | | MEDIAN\_E3CellBodySpotTotalCountCh4 | 1 | 100 | | MEDIAN\_E3CellBodyTotalIntenCh2 | 8.07360903568864 | 807.360903568864 | | MEDIAN\_E3CellBodyVarIntenCh2 | 1 | 100 | | MEDIAN\_E3NeuriteCriticalValueCh2 | 1.42070484581498 | 142.070484581498 | | MEDIAN\_E3NeuriteDendriteMaxCh2 | 1 | 100 | | MEDIAN\_E3NeuriteRamificationIndexCh2 | 1 | 100 | | MEDIAN\_E3NeuriteSpot%OverlapAreaCh3 | 1 | 100 | | MEDIAN\_E3NeuriteSpotAvgAreaCh4 | 1 | 100 | | MEDIAN\_E3NeuriteSpotAvgCountCh4 | 1 | 100 | | MEDIAN\_E3NeuriteSpotOverlapAreaCh3 | 2.01840225892216 | 201.840225892216 | | MEDIAN\_E3NeuriteSpotTotalAreaCh4 | 1 | 100 | | MEDIAN\_E3TotalIntenCh3 | 8.07360903568864 | 807.360903568864 | | MEDIAN\_E3TotalIntenCh4 | 1 | 100 | | MEDIAN\_NeuriteAvgIntenCh2 | 4 | 400 | | MEDIAN\_NeuriteSpotAvgAreaCh3 | 2.01840225892216 | 201.840225892216 | | MEDIAN\_NeuriteSpotAvgCountCh3 | 1 | 100 | | MEDIAN\_E3NeuriteVarIntenCh2 | 4 | 400 | | MEDIAN\_NeuriteCriticalValueCh2 | 1.42070484581498 | 142.070484581498 | | %HIGH\_E3CellBodySpotTotalAreaCh3 | 1 | 100 | | %HIGH\_E3CellBodySpotTotalCountCh4 | 1 | 100 | | %HIGH\_E3CellBodySpotTotalIntenCh4 | 1 | 100 | | %HIGH\_E3CellBodyVarIntenCh2 | 1 | 100 | | %HIGH\_E3CrossPointAvgCountCh2 | 1 | 100 | | %HIGH\_E3NeuriteDendriteMaxCh2 | 1 | 100 | | %HIGH\_E3NeuriteMaxLengthWithoutBranchesCh2 | 1 | 100 | | %HIGH\_E3NeuriteSpot%OverlapCountCh4 | 1 | 100 | | %HIGH\_E3NeuriteSpotAvgAreaCh4 | 1 | 100 | | %HIGH\_E3NeuriteSpotAvgCountCh4 | 1 | 100 | | %HIGH\_E3NeuriteSpotTotalAreaRatioCh4Ch3 | 1 | 100 | | %HIGH\_E3NeuriteSpotTotalIntenCh3 | 1 | 100 | | %HIGH\_E3NeuriteSpotTotalIntenRatioCh3Ch2 | 1 | 100 | | %HIGH\_E3NeuriteWidthCh2 | 1 | 100 | | %HIGH\_E3TotalIntenCh3 | 1 | 100 | | %HIGH\_NeuriteAvgLengthCh2 | 1 | 100 | | %HIGH\_NeuriteSpot%OverlapAreaCh3 | 1 | 100 | | %HIGH\_NeuriteSpot%OverlapAreaCh4 | 1 | 100 | | %HIGH\_NeuriteSpot%OverlapCountCh3 | 1 | 100 | | %HIGH\_NeuriteSpotAvgAreaCh3 | 1 | 100 | | %HIGH\_NeuriteSpotAvgIntenCh4 | 1 | 100 | | %HIGH\_NeuriteSpotTotalCountCh4 | 1 | 100 | | %HIGH\_NeuriteSpotTotalIntenRatioCh4Ch3 | 1 | 100 | | %HIGH\_NeuriteTotalIntenCh2 | 1 | 100 | | %HIGH\_NeuriteVarIntenCh2 | 1 | 100 | | %SelectedNeurons | 1 | 100 | | CellBodySpot%OverlapAreaPerWellCh3 | 1 | 100 | | CellBodySpot%OverlapCountPerWellCh4 | 1 | 100 | | CV\_NeuriteSpotTotalCountCh4 | 1 | 100 | | CV\_NeuriteSpotTotalIntenRatioCh4Ch3 | 1 | 100 | | CV\_NeuriteTotalCountCh2 | 1 | 100 | | CV\_NeuriteTotalIntenCh2 | 1 | 100 | | E1\_%EventType2Neurons | 1 | 100 | | E1\_%EventType3Neurons | 1 | 100 | | E1\_EventType2NeuronCount | 1 | 100 | | E1\_EventType3NeuronCount | 1 | 100 | | E2\_%EventType1Neurons | 1 | 100 | | E2\_%EventType2Neurons | 1 | 100 | | E2\_EventType2NeuronCount | 1 | 100 | | E2\_EventType3NeuronCount | 1 | 100 | | E3\_%EventType1Neurons | 1 | 100 | | E3\_%EventType2Neurons | 1 | 100 | | E3\_EventType1NeuronCount | 1 | 100 | | E3\_EventType2NeuronCount | 1 | 100 | | MAD\_CellBodyNucTotalAreaCh1 | 2.01840225892216 | 201.840225892216 | | MAD\_CellBodyShapeP2ACh2 | 1 | 100 | | MAD\_CellBodySpot%OverlapAreaCh3 | 1 | 100 | | MAD\_CellBodySpotAvgIntenCh3 | 4 | 400 | | MAD\_CellBodySpotOverlapAreaCh4 | 1 | 100 | | MAD\_CellBodySpotOverlapCountCh4 | 1 | 100 | | %HIGH\_E3CellBodySpotTotalIntenRatioCh3Ch2 | 1 | 100 | | %HIGH\_E3NeuriteAvgIntenCh2 | 1 | 100 | | %HIGH\_E3NeuriteSpot%OverlapAreaCh4 | 1 | 100 | | %HIGH\_E3NeuriteSpot%OverlapCountCh3 | 1 | 100 | | %HIGH\_E3NeuriteSpotOverlapAreaCh3 | 1 | 100 | | %HIGH\_E3NeuriteSpotOverlapAreaCh4 | 1 | 100 | | %HIGH\_E3NeuriteSpotOverlapCountCh3 | 1 | 100 | | %HIGH\_E3NeuriteSpotTotalCountCh4 | 1 | 100 | | %HIGH\_E3NeuriteTotalIntenCh2 | 1 | 100 | | %HIGH\_E3NeuriteVarIntenCh2 | 1 | 100 | | %HIGH\_E3TotalIntenCh4 | 1 | 100 | | %HIGH\_NeuriteMaxLengthWithoutBranchesCh2 | 1 | 100 | | %HIGH\_NeuriteSpotAvgAreaCh4 | 1 | 100 | | %HIGH\_NeuriteSpotAvgCountCh3 | 1 | 100 | | %HIGH\_NeuriteSpotTotalAreaCh4 | 1 | 100 | | %HIGH\_NeuriteSpotTotalCountCh3 | 1 | 100 | | %HIGH\_NeuriteSpotTotalIntenCh4 | 1 | 100 | | %HIGH\_NeuriteTotalAreaCh2 | 1 | 100 | | %HIGH\_NeuriteWidthCh2 | 1 | 100 | | %HIGH\_TotalIntenCh3 | 1 | 100 | | %NonNeuronalCells | 1 | 100 | | CellBodySpot%OverlapCountPerWellCh3 | 1 | 100 | | CV\_NeuriteSpotTotalAreaRatioCh4Ch3 | 1 | 100 | | CV\_NeuriteSpotTotalIntenCh3 | 1 | 100 | | CV\_NeuriteSpotTotalIntenRatioCh3Ch2 | 1 | 100 | | CV\_NeuriteVarIntenCh2 | 1 | 100 | | E1\_%EventType1Neurons | 1 | 100 | | E3\_EventType3NeuronCount | 1 | 100 | | EventType2NeuronCount | 1 | 100 | | EventType3NeuronCount | 1 | 100 | | MAD\_AvgIntenCh4 | 1 | 100 | | MAD\_BranchPointAvgCountCh2 | 1 | 100 | | MAD\_BranchPointCountPerNeuriteLengthCh2 | 0.703875968992248 | 70.3875968992248 | | MAD\_BranchPointTotalCountCh2 | 1 | 100 | | MAD\_CellBodyAreaCh2 | 2.01840225892216 | 201.840225892216 | | MAD\_CellBodyNucAvgIntenCh1 | 4 | 400 | | MAD\_CellBodyNucCountCh2 | 1 | 100 | | MAD\_CellBodySpot%OverlapAreaCh4 | 1 | 100 | | MAD\_CellBodySpot%OverlapCountCh3 | 1 | 100 | | MAD\_CellBodySpotOverlapAreaCh3 | 2.01840225892216 | 201.840225892216 | | MAD\_CellBodySpotOverlapCountCh3 | 1 | 100 | | MAD\_CellBodySpotTotalCountCh4 | 1 | 100 | | MAD\_E1AvgIntenCh3 | 4 | 400 | | MAD\_E1BranchPointAvgCountCh2 | 1 | 100 | | MAD\_E1BranchPointAvgDistFromCellBodyCh2 | 1.42070484581498 | 142.070484581498 | | MAD\_E1BranchPointCountPerNeuriteLengthCh2 | 0.703875968992248 | 70.3875968992248 | | MAD\_E1CellBodyNucTotalAreaCh1 | 2.01840225892216 | 201.840225892216 | | MAD\_E1CellBodyNucTotalIntenCh1 | 8.07360903568864 | 807.360903568864 | | MAD\_E1CellBodySpot%OverlapAreaCh3 | 1 | 100 | | MAD\_E1CellBodySpot%OverlapAreaCh4 | 1 | 100 | | MAD\_E1CellBodySpotOverlapAreaCh3 | 2.01840225892216 | 201.840225892216 | | MAD\_E1CellBodySpotOverlapAreaCh4 | 1 | 100 | | CV\_NeuriteSpot%OverlapAreaCh3 | 1 | 100 | | CV\_NeuriteSpotAvgAreaCh4 | 1 | 100 | | MAD\_NeuriteSpotTotalAreaCh3 | 2.01840225892216 | 201.840225892216 | | MAD\_NeuriteSpotTotalAreaRatioCh4Ch3 | 1 | 100 | | MAD\_NeuriteSpotTotalIntenCh3 | 8.07360903568864 | 807.360903568864 | | MAD\_NeuriteSpotTotalIntenRatioCh4Ch2 | 1 | 100 | | MAD\_NeuriteSpotTotalIntenRatioCh4Ch3 | 1 | 100 | | MAD\_NeuriteTotalIntenCh2 | 8.07360903568864 | 807.360903568864 | | MAD\_NeuriteVarIntenCh2 | 4 | 400 | | MEAN\_CellBodyAvgIntenCh2 | 4 | 400 | | MEAN\_CellBodySpotAvgIntenCh4 | 1 | 100 | | MEAN\_CellBodySpotTotalIntenRatioCh4Ch2 | 1 | 100 | | MEAN\_CellBodySpotTotalIntenRatioCh4Ch3 | 1 | 100 | | MEAN\_CellBodyTotalIntenCh2 | 8.07360903568864 | 807.360903568864 | | MEAN\_CellBodyVarIntenCh2 | 1 | 100 | | MEAN\_CrossPointAvgCountCh2 | 1 | 100 | | MEAN\_E1CellBodyAreaCh2 | 2.01840225892216 | 201.840225892216 | | MEAN\_E1CellBodyNucCountCh2 | 1 | 100 | | MEAN\_E1CellBodyShapeLWRCh2 | 1 | 100 | | MEAN\_E1CellBodyShapeP2ACh2 | 1 | 100 | | MEAN\_E1CellBodySpotAvgIntenCh4 | 1 | 100 | | MEAN\_E1CellBodySpotTotalAreaCh3 | 2.01840225892216 | 201.840225892216 | | MEAN\_E1CellBodySpotTotalAreaCh4 | 1 | 100 | | MEAN\_E1CellBodySpotTotalCountCh3 | 1 | 100 | | MEAN\_E1CellBodySpotTotalIntenCh3 | 8.07360903568864 | 807.360903568864 | | MEAN\_E1CellBodySpotTotalIntenCh4 | 1 | 100 | | MEAN\_E1CellBodyVarIntenCh2 | 1 | 100 | | MEAN\_E1NeuriteAvgIntenCh2 | 4 | 400 | | MEDIAN\_E1NeuriteSpotOverlapAreaCh4 | 1 | 100 | | MEDIAN\_E1NeuriteSpotTotalCountCh3 | 1 | 100 | | MEDIAN\_E1NeuriteTotalLengthCh2 | 1.42070484581498 | 142.070484581498 | | MEDIAN\_E1TotalIntenCh3 | 8.07360903568864 | 807.360903568864 | | MEDIAN\_E2BranchPointAvgDistFromCellBodyCh2 | 1.42070484581498 | 142.070484581498 | | MEDIAN\_E2BranchPointCountPerNeuriteLengthCh2 | 0.703875968992248 | 70.3875968992248 | | MEDIAN\_E2CellBodyNucTotalAreaCh1 | 2.01840225892216 | 201.840225892216 | | MEDIAN\_E2CellBodyNucTotalIntenCh1 | 8.07360903568864 | 807.360903568864 | | MEDIAN\_E2CellBodySpot%OverlapCountCh4 | 1 | 100 | | MEDIAN\_E2CellBodySpotOverlapAreaCh4 | 1 | 100 | | MEDIAN\_E2CellBodySpotOverlapCountCh4 | 1 | 100 | | MEDIAN\_E2CrossPointTotalCountCh2 | 1 | 100 | | MEDIAN\_E2NeuriteAvgLengthCh2 | 1.42070484581498 | 142.070484581498 | | MEDIAN\_E2NeuriteMaxLengthWithBranchesCh2 | 1.42070484581498 | 142.070484581498 | | MEDIAN\_E2NeuriteMaxLengthWithoutBranchesCh2 | 1.42070484581498 | 142.070484581498 | | MEDIAN\_E2NeuriteSpot%OverlapCountCh4 | 1 | 100 | | MEDIAN\_E2NeuriteSpotAvgCountCh3 | 1 | 100 | | MEDIAN\_E2NeuriteSpotOverlapCountCh4 | 1 | 100 | | MEDIAN\_E2NeuriteSpotTotalAreaCh3 | 2.01840225892216 | 201.840225892216 | | MEDIAN\_E2NeuriteSpotTotalAreaRatioCh4Ch3 | 1 | 100 | | MEDIAN\_E2NeuriteSpotTotalCountCh3 | 1 | 100 | | MEDIAN\_E2NeuriteTotalAreaCh2 | 2.01840225892216 | 201.840225892216 | | SD\_E1NeuriteSpotOverlapAreaCh4 | 1 | 100 | | SD\_E1NeuriteSpotTotalCountCh3 | 1 | 100 | | SD\_E1NeuriteSpotTotalCountCh4 | 1 | 100 | | %HIGH\_E3CellBodySpotTotalIntenRatioCh4Ch2 | 1 | 100 | | %HIGH\_E3CellBodySpotTotalIntenRatioCh4Ch3 | 1 | 100 | | %HIGH\_E3NeuriteAvgLengthCh2 | 1 | 100 | | %HIGH\_E3NeuriteMaxLengthWithBranchesCh2 | 1 | 100 | | %HIGH\_E3NeuriteSpotAvgAreaCh3 | 1 | 100 | | %HIGH\_E3NeuriteSpotAvgCountCh3 | 1 | 100 | | %HIGH\_E3NeuriteSpotAvgIntenCh4 | 1 | 100 | | %HIGH\_E3NeuriteSpotTotalAreaCh3 | 1 | 100 | | %HIGH\_E3NeuriteSpotTotalIntenCh4 | 1 | 100 | | %HIGH\_E3NeuriteSpotTotalIntenRatioCh4Ch2 | 1 | 100 | | %HIGH\_E3NeuriteTotalLengthCh2 | 1 | 100 | | %HIGH\_NeuriteCriticalValueCh2 | 1 | 100 | | %HIGH\_NeuriteDendriteMaxCh2 | 1 | 100 | | %HIGH\_NeuriteRamificationIndexCh2 | 1 | 100 | | %HIGH\_NeuriteSpot%OverlapCountCh4 | 1 | 100 | | %HIGH\_NeuriteSpotAvgIntenCh3 | 1 | 100 | | %HIGH\_NeuriteSpotOverlapAreaCh4 | 1 | 100 | | %HIGH\_NeuriteSpotOverlapCountCh3 | 1 | 100 | | %HIGH\_NeuriteSpotOverlapCountCh4 | 1 | 100 | | %HIGH\_NeuriteSpotTotalAreaRatioCh4Ch3 | 1 | 100 | | %HIGH\_NeuriteSpotTotalIntenRatioCh3Ch2 | 1 | 100 | | %HIGH\_NeuriteTotalCountCh2 | 1 | 100 | | %NeuronalCells | 1 | 100 | | BranchPointTotalCountPerWellCh2 | 1 | 100 | | CellBodySpot%OverlapAreaPerWellCh4 | 1 | 100 | | CV\_NeuriteSpotTotalAreaCh4 | 1 | 100 | | CV\_NeuriteSpotTotalCountCh3 | 1 | 100 | | CV\_NeuriteSpotTotalIntenCh4 | 1 | 100 | | CV\_NeuriteTotalAreaCh2 | 1 | 100 | | CV\_TotalIntenCh3 | 1 | 100 | | E1\_EventType1NeuronCount | 1 | 100 | | E2\_%EventType3Neurons | 1 | 100 | | E2\_EventType1NeuronCount | 1 | 100 | | E3\_%EventType3Neurons | 1 | 100 | | MAD\_CellBodyAvgIntenCh2 | 4 | 400 | | MAD\_CellBodySpotTotalAreaCh3 | 2.01840225892216 | 201.840225892216 | | MAD\_CellBodySpotTotalAreaRatioCh4Ch3 | 1 | 100 | | MAD\_CellBodySpotTotalCountCh3 | 1 | 100 | | MAD\_CellBodySpotTotalIntenRatioCh4Ch2 | 1 | 100 | | MAD\_CellBodySpotTotalIntenRatioCh4Ch3 | 1 | 100 | | MAD\_E1CellBodyShapeLWRCh2 | 1 | 100 | | MAD\_E1CellBodyShapeP2ACh2 | 1 | 100 | | MAD\_E1CellBodySpotAvgIntenCh3 | 4 | 400 | | MAD\_E1CellBodySpotAvgIntenCh4 | 1 | 100 | | MAD\_E1CellBodySpotTotalAreaCh3 | 2.01840225892216 | 201.840225892216 | | MAD\_E1CellBodySpotTotalIntenCh3 | 8.07360903568864 | 807.360903568864 | | MAD\_E1CellBodySpotTotalIntenRatioCh4Ch2 | 1 | 100 | | MAD\_E1CellBodySpotTotalIntenRatioCh4Ch3 | 1 | 100 | | MEAN\_E2NeuriteVarIntenCh2 | 4 | 400 | | MAD\_CellBodySpotTotalIntenCh4 | 1 | 100 | | MAD\_CellBodyTotalIntenCh2 | 8.07360903568864 | 807.360903568864 | | MAD\_CrossPointTotalCountCh2 | 1 | 100 | | MAD\_E1CellBodyAreaCh2 | 2.01840225892216 | 201.840225892216 | | MAD\_E1CellBodyNucAvgIntenCh1 | 4 | 400 | | MAD\_E1CellBodySpotOverlapCountCh3 | 1 | 100 | | MAD\_E1CellBodySpotOverlapCountCh4 | 1 | 100 | | MAD\_E1CellBodySpotTotalCountCh3 | 1 | 100 | | MAD\_E1CellBodySpotTotalCountCh4 | 1 | 100 | | MAD\_E1CellBodySpotTotalIntenCh4 | 1 | 100 | | MEAN\_E2TotalIntenCh4 | 1 | 100 | | MEAN\_E3AvgIntenCh3 | 4 | 400 | | MEAN\_E3BranchPointAvgCountCh2 | 1 | 100 | | MEAN\_E3CellBodyAreaCh2 | 2.01840225892216 | 201.840225892216 | | MEAN\_E3CellBodySpot%OverlapAreaCh4 | 1 | 100 | | MEAN\_E3CellBodySpot%OverlapCountCh4 | 1 | 100 | | MEAN\_E3CellBodySpotAvgIntenCh4 | 1 | 100 | | MEAN\_E3CellBodySpotTotalIntenCh3 | 8.07360903568864 | 807.360903568864 | | MEAN\_E3CellBodySpotTotalIntenRatioCh3Ch2 | 1 | 100 | | MEAN\_E3NeuriteAvgLengthCh2 | 1.42070484581498 | 142.070484581498 | | MEAN\_E3NeuriteRamificationIndexCh2 | 1 | 100 | | MEAN\_E3NeuriteSpotAvgIntenCh3 | 4 | 400 | | MEAN\_E3NeuriteSpotOverlapAreaCh4 | 1 | 100 | | MEAN\_E3NeuriteSpotOverlapCountCh3 | 1 | 100 | | MEAN\_E3NeuriteSpotOverlapCountCh4 | 1 | 100 | | MEAN\_E3NeuriteSpotTotalCountCh3 | 1 | 100 | | MEAN\_E3NeuriteSpotTotalIntenCh4 | 1 | 100 | | MEAN\_E3NeuriteTotalCountCh2 | 1 | 100 | | MEAN\_E3NeuriteTotalIntenCh2 | 8.07360903568864 | 807.360903568864 | | MEAN\_NeuriteCriticalValueCh2 | 1.42070484581498 | 142.070484581498 | | MEAN\_NeuriteMaxLengthWithBranchesCh2 | 1.42070484581498 | 142.070484581498 | | MEAN\_NeuriteSpotAvgAreaCh4 | 1 | 100 | | MEAN\_NeuriteSpotAvgCountCh4 | 1 | 100 | | MEAN\_NeuriteSpotTotalAreaRatioCh4Ch3 | 1 | 100 | | MEDIAN\_NeuriteSpotTotalIntenCh3 | 8.07360903568864 | 807.360903568864 | | MEDIAN\_NeuriteSpotTotalIntenRatioCh3Ch2 | 1 | 100 | | MEDIAN\_NeuriteTotalAreaCh2 | 2.01840225892216 | 201.840225892216 | | MEDIAN\_TotalIntenCh3 | 8.07360903568864 | 807.360903568864 | | NA\_NeuriteSpot%OverlapAreaPerWellCh4 | 1 | 100 | | NA\_NeuriteSpotOverlapTotalAreaPerNeuriteLengthCh4 | 1 | 100 | | NA\_NeuriteSpotOverlapTotalAreaPerWellCh4 | 1 | 100 | | NA\_NeuriteSpotTotalAreaPerNeuriteLengthCh3 | 0 | 100 | | NA\_NeuriteSpotTotalAreaPerWellCh3 | 0 | 100 | | NA\_NeuriteSpotTotalIntenPerNeuriteLengthCh3 | 0 | 100 | | NA\_NeuriteSpotTotalIntenPerWellCh3 | 0 | 100 | | NeuriteSpot%OverlapAreaPerWellCh4 | 1 | 100 | | NeuriteSpot%OverlapCountPerWellCh3 | 1 | 100 | | NeuriteSpotAvgIntenPerFieldCh3 | 4 | 400 | | NeuriteSpotOverlapTotalAreaPerFieldCh4 | 1 | 100 | | NeuriteSpotOverlapTotalAreaPerNeuriteCh4 | 1 | 100 | | NeuriteSpotOverlapTotalAreaPerNeuronCh4 | 1 | 100 | | MEAN\_E3AvgIntenCh4 | 1 | 100 | | MEAN\_E3CellBodyAvgIntenCh2 | 4 | 400 | | MEAN\_E3CellBodyShapeLWRCh2 | 1 | 100 | | MEAN\_E3CellBodySpot%OverlapCountCh3 | 1 | 100 | | MEAN\_E3CellBodySpotOverlapAreaCh3 | 2.01840225892216 | 201.840225892216 | | MEAN\_E3CellBodySpotOverlapCountCh3 | 1 | 100 | | MEAN\_E3CellBodySpotTotalAreaCh4 | 1 | 100 | | MEAN\_E3CellBodySpotTotalCountCh4 | 1 | 100 | | MEAN\_E3CellBodyTotalIntenCh2 | 8.07360903568864 | 807.360903568864 | | MEAN\_E3NeuriteCriticalValueCh2 | 1.42070484581498 | 142.070484581498 | | MEAN\_E3NeuriteDendriteMaxCh2 | 1 | 100 | | MEAN\_E3NeuriteSpot%OverlapAreaCh3 | 1 | 100 | | MEAN\_E3NeuriteSpot%OverlapAreaCh4 | 1 | 100 | | MEAN\_E3NeuriteSpot%OverlapCountCh3 | 1 | 100 | | MEAN\_E3NeuriteSpotAvgCountCh4 | 1 | 100 | | MEAN\_E3NeuriteSpotOverlapAreaCh3 | 2.01840225892216 | 201.840225892216 | | MEAN\_E3NeuriteSpotTotalAreaCh4 | 1 | 100 | | MEAN\_E3NeuriteSpotTotalCountCh4 | 1 | 100 | | MEAN\_E3NeuriteVarIntenCh2 | 4 | 400 | | MEAN\_E3TotalIntenCh3 | 8.07360903568864 | 807.360903568864 | | MEAN\_E3TotalIntenCh4 | 1 | 100 | | MEAN\_NeuriteAvgIntenCh2 | 4 | 400 | | MEAN\_NeuriteSpotAvgAreaCh3 | 2.01840225892216 | 201.840225892216 | | MEAN\_NeuriteSpotAvgCountCh3 | 1 | 100 | | MEAN\_NeuriteSpotAvgIntenCh4 | 1 | 100 | | MEAN\_NeuriteSpotTotalAreaCh3 | 2.01840225892216 | 201.840225892216 | | MEDIAN\_NeuriteSpotTotalCountCh4 | 1 | 100 | | MEDIAN\_NeuriteTotalCountCh2 | 1 | 100 | | MEDIAN\_NeuriteTotalLengthCh2 | 1.42070484581498 | 142.070484581498 | | MEDIAN\_TotalIntenCh4 | 1 | 100 | | NA\_NeuriteAvgIntenPerWellCh2 | 0 | 100 | | NA\_NeuriteSpot%OverlapCountPerWellCh3 | 0 | 100 | | NA\_NeuriteSpotAvgIntenPerNeuriteLengthCh3 | 0 | 100 | | NA\_NeuriteSpotOverlapTotalCountPerNeuriteLengthCh4 | 1 | 100 | | NA\_NeuriteSpotOverlapTotalCountPerWellCh4 | 1 | 100 | | NA\_NeuriteSpotTotalCountPerNeuriteLengthCh4 | 1 | 100 | | NA\_NeuriteSpotTotalCountPerWellCh3 | 0 | 100 | | NeuriteAvgIntenPerFieldCh2 | 4 | 400 | | NeuriteSpotOverlapTotalAreaPerNeuriteCh3 | 2.01840225892216 | 201.840225892216 | | NeuriteSpotOverlapTotalAreaPerNeuriteLengthCh4 | 1 | 100 | | NeuriteSpotOverlapTotalAreaPerWellCh4 | 1 | 100 | | NeuriteSpotOverlapTotalCountPerNeuriteCh4 | 1 | 100 | | NeuriteSpotOverlapTotalCountPerNeuriteLengthCh3 | 0.703875968992248 | 70.3875968992248 | | NeuriteSpotOverlapTotalCountPerNeuronCh3 | 1 | 100 | | NeuriteSpotTotalCountPerFieldCh3 | 1 | 100 | | NeuriteSpotTotalCountPerNeuriteCh3 | 1 | 100 | | NeuriteSpotTotalCountPerNeuriteLengthCh4 | 1 | 100 | | NeuriteSpotTotalCountPerNeuronCh4 | 1 | 100 | | NeuriteSpotTotalIntenPerFieldCh4 | 1 | 100 | | NeuriteSpotTotalIntenPerNeuriteCh4 | 1 | 100 | | SE\_CellBodyNucTotalAreaCh1 | 2.01840225892216 | 201.840225892216 | | %HIGH\_E1TotalIntenCh3 | 1 | 100 | | %HIGH\_E2BranchPointCountPerNeuriteLengthCh2 | 1 | 100 | | %HIGH\_E2CellBodyNucAvgIntenCh1 | 1 | 100 | | %HIGH\_E2CellBodyNucTotalAreaCh1 | 1 | 100 | | %HIGH\_E2CellBodySpot%OverlapAreaCh4 | 1 | 100 | | %HIGH\_E2CellBodySpot%OverlapCountCh4 | 1 | 100 | | %HIGH\_E2CellBodySpotAvgIntenCh4 | 1 | 100 | | %HIGH\_E2CellBodySpotOverlapCountCh3 | 1 | 100 | | %HIGH\_E2CellBodySpotTotalAreaCh4 | 1 | 100 | | %HIGH\_E2CellBodySpotTotalCountCh3 | 1 | 100 | | %HIGH\_E2CellBodyTotalIntenCh2 | 1 | 100 | | %HIGH\_E2CellBodyVarIntenCh2 | 1 | 100 | | %HIGH\_E2CrossPointTotalCountCh2 | 1 | 100 | | %HIGH\_E2NeuriteCriticalValueCh2 | 1 | 100 | | %HIGH\_E2NeuriteMaxLengthWithBranchesCh2 | 1 | 100 | | %HIGH\_E2NeuriteSpot%OverlapCountCh3 | 1 | 100 | | %HIGH\_E2NeuriteSpotAvgAreaCh3 | 1 | 100 | | %HIGH\_E2NeuriteSpotAvgCountCh3 | 1 | 100 | | %HIGH\_E2NeuriteSpotOverlapAreaCh4 | 1 | 100 | | %HIGH\_E2NeuriteSpotOverlapCountCh4 | 1 | 100 | | %HIGH\_E2NeuriteSpotTotalIntenCh4 | 1 | 100 | | %HIGH\_E2NeuriteTotalAreaCh2 | 1 | 100 | | %HIGH\_E3AvgIntenCh3 | 1 | 100 | | %HIGH\_E3AvgIntenCh4 | 1 | 100 | | %HIGH\_E3CellBodyNucAvgIntenCh1 | 1 | 100 | | %HIGH\_E3CellBodySpot%OverlapCountCh4 | 1 | 100 | | %HIGH\_E3CellBodySpotOverlapAreaCh3 | 1 | 100 | | CV\_E3AvgIntenCh4 | 1 | 100 | | CV\_E3CellBodyNucAvgIntenCh1 | 1 | 100 | | CV\_E3CellBodyNucTotalAreaCh1 | 1 | 100 | | CV\_E3CellBodySpot%OverlapCountCh4 | 1 | 100 | | CV\_E3CellBodySpotOverlapAreaCh3 | 1 | 100 | | CV\_E3CellBodySpotTotalAreaCh4 | 1 | 100 | | CV\_E3CellBodySpotTotalAreaRatioCh4Ch3 | 1 | 100 | | CV\_E3CellBodySpotTotalCountCh3 | 1 | 100 | | CV\_E3CellBodySpotTotalIntenCh3 | 1 | 100 | | CV\_E3CellBodyTotalIntenCh2 | 1 | 100 | | CV\_E3CrossPointTotalCountCh2 | 1 | 100 | | CV\_E3NeuriteCriticalValueCh2 | 1 | 100 | | CV\_E3NeuriteMaxLengthWithBranchesCh2 | 1 | 100 | | CV\_E3NeuriteRamificationIndexCh2 | 1 | 100 | | CV\_E3NeuriteSpot%OverlapAreaCh3 | 1 | 100 | | CV\_E3NeuriteSpotAvgAreaCh3 | 1 | 100 | | CV\_E3NeuriteSpotAvgIntenCh3 | 1 | 100 | | CV\_E3NeuriteSpotOverlapCountCh4 | 1 | 100 | | CV\_E3NeuriteSpotTotalAreaCh4 | 1 | 100 | | CV\_E3NeuriteSpotTotalIntenRatioCh4Ch3 | 1 | 100 | | CV\_E3NeuriteTotalAreaCh2 | 1 | 100 | | CV\_NeuriteAvgIntenCh2 | 1 | 100 | | CV\_NeuriteSpotAvgCountCh4 | 1 | 100 | | CV\_NeuriteSpotOverlapAreaCh3 | 1 | 100 | | CV\_NeuriteSpotOverlapAreaCh4 | 1 | 100 | | CV\_NeuriteSpotOverlapCountCh3 | 1 | 100 | | CV\_NeuriteSpotTotalAreaCh3 | 1 | 100 | | MAD\_NeuriteSpotOverlapAreaCh4 | 1 | 100 | | MAD\_NeuriteSpotOverlapCountCh3 | 1 | 100 | | MAD\_NeuriteSpotOverlapCountCh4 | 1 | 100 | | MAD\_NeuriteSpotTotalAreaCh4 | 1 | 100 | | MAD\_NeuriteSpotTotalCountCh3 | 1 | 100 | | MAD\_NeuriteSpotTotalIntenCh4 | 1 | 100 | | MAD\_NeuriteWidthCh2 | 1.42070484581498 | 142.070484581498 | | MEAN\_AvgIntenCh3 | 4 | 400 | | MEAN\_AvgIntenCh4 | 1 | 100 | | MEAN\_BranchPointAvgDistFromCellBodyCh2 | 1.42070484581498 | 142.070484581498 | | MEAN\_CellBodyAreaCh2 | 2.01840225892216 | 201.840225892216 | | MEAN\_CellBodyShapeLWRCh2 | 1 | 100 | | MEAN\_CellBodySpot%OverlapCountCh4 | 1 | 100 | | MEAN\_CellBodySpotOverlapAreaCh3 | 2.01840225892216 | 201.840225892216 | | MEAN\_CellBodySpotTotalAreaCh4 | 1 | 100 | | MEAN\_CellBodySpotTotalAreaRatioCh4Ch3 | 1 | 100 | | MEAN\_CellBodySpotTotalCountCh3 | 1 | 100 | | MEAN\_CellBodySpotTotalIntenCh3 | 8.07360903568864 | 807.360903568864 | | MEAN\_E1AvgIntenCh3 | 4 | 400 | | MEAN\_E1AvgIntenCh4 | 1 | 100 | | MEAN\_E1BranchPointAvgDistFromCellBodyCh2 | 1.42070484581498 | 142.070484581498 | | MEAN\_E1CellBodyAvgIntenCh2 | 4 | 400 | | MEAN\_E1CellBodyNucTotalIntenCh1 | 8.07360903568864 | 807.360903568864 | | MEAN\_E1CellBodySpotTotalAreaRatioCh4Ch3 | 1 | 100 | | MEAN\_E1CellBodySpotTotalIntenRatioCh3Ch2 | 1 | 100 | | MEAN\_E1CellBodySpotTotalIntenRatioCh4Ch2 | 1 | 100 | | MEAN\_E1CrossPointAvgCountCh2 | 1 | 100 | | MEAN\_E1NeuriteDendriteMaxCh2 | 1 | 100 | | MEAN\_E1NeuriteMaxLengthWithoutBranchesCh2 | 1.42070484581498 | 142.070484581498 | | MEDIAN\_E1NeuriteSpotOverlapAreaCh3 | 2.01840225892216 | 201.840225892216 | | MEDIAN\_E1NeuriteSpotOverlapCountCh3 | 1 | 100 | | MEDIAN\_E1NeuriteSpotOverlapCountCh4 | 1 | 100 | | MEDIAN\_E1NeuriteSpotTotalAreaCh3 | 2.01840225892216 | 201.840225892216 | | MEDIAN\_E1NeuriteSpotTotalAreaCh4 | 1 | 100 | | MEDIAN\_E1NeuriteSpotTotalCountCh4 | 1 | 100 | | MEDIAN\_E1NeuriteSpotTotalIntenRatioCh4Ch3 | 1 | 100 | | MEDIAN\_E1NeuriteWidthCh2 | 1.42070484581498 | 142.070484581498 | | MEDIAN\_E2BranchPointTotalCountCh2 | 1 | 100 | | MEDIAN\_E2CellBodyAvgIntenCh2 | 4 | 400 | | MEDIAN\_E2CellBodyNucCountCh2 | 1 | 100 | | MEDIAN\_E2CellBodySpot%OverlapAreaCh3 | 1 | 100 | | MEDIAN\_E2CellBodySpot%OverlapCountCh3 | 1 | 100 | | MEDIAN\_E2CellBodySpotTotalAreaCh3 | 2.01840225892216 | 201.840225892216 | | MEDIAN\_E2CellBodySpotTotalAreaRatioCh4Ch3 | 1 | 100 | | MEDIAN\_E2CellBodySpotTotalIntenRatioCh4Ch2 | 1 | 100 | | MEDIAN\_E2CrossPointAvgCountCh2 | 1 | 100 | | MEDIAN\_E2NeuriteAvgIntenCh2 | 4 | 400 | | MEDIAN\_E2NeuriteSpot%OverlapAreaCh4 | 1 | 100 | | MEDIAN\_E2NeuriteSpotAvgIntenCh4 | 1 | 100 | | %EventType1Neurons | 1 | 100 | | %HIGH\_BranchPointCountPerNeuriteLengthCh2 | 1 | 100 | | %HIGH\_BranchPointTotalCountCh2 | 1 | 100 | | %HIGH\_CellBodyAvgIntenCh2 | 1 | 100 | | CellBodySpotOverlapTotalAreaPerFieldCh4 | 1 | 100 | | CellBodySpotOverlapTotalAreaPerNeuronCh4 | 1 | 100 | | CellBodySpotOverlapTotalCountPerWellCh3 | 1 | 100 | | CellBodySpotTotalCountPerFieldCh4 | 1 | 100 | | CellBodySpotTotalCountPerNeuronCh4 | 1 | 100 | | CellBodySpotTotalCountPerWellCh4 | 1 | 100 | | CellBodySpotTotalIntenPerFieldCh3 | 8.07360903568864 | 807.360903568864 | | CellBodySpotTotalIntenPerNeuronCh3 | 8.07360903568864 | 807.360903568864 | | CellBodySpotTotalIntenPerWellCh3 | 8.07360903568864 | 807.360903568864 | | CV\_BranchPointCountPerNeuriteLengthCh2 | 1 | 100 | | CV\_BranchPointTotalCountCh2 | 1 | 100 | | CV\_CellBodyAvgIntenCh2 | 1 | 100 | | CV\_CellBodyNucCountCh2 | 1 | 100 | | CV\_CellBodyNucTotalIntenCh1 | 1 | 100 | | CV\_CellBodySpot%OverlapAreaCh4 | 1 | 100 | | CV\_CellBodySpot%OverlapCountCh3 | 1 | 100 | | CV\_CellBodySpotAvgIntenCh4 | 1 | 100 | | CV\_CellBodySpotTotalIntenRatioCh3Ch2 | 1 | 100 | | CV\_CellBodySpotTotalIntenRatioCh4Ch3 | 1 | 100 | | CV\_E1BranchPointCountPerNeuriteLengthCh2 | 1 | 100 | | CV\_E1CellBodyNucTotalAreaCh1 | 1 | 100 | | CV\_E1CellBodyShapeP2ACh2 | 1 | 100 | | CV\_E1CellBodySpot%OverlapAreaCh3 | 1 | 100 | | CV\_E1CellBodySpot%OverlapAreaCh4 | 1 | 100 | | CV\_E1CellBodySpot%OverlapCountCh4 | 1 | 100 | | CV\_E1CellBodySpotAvgIntenCh3 | 1 | 100 | | CV\_E1CellBodySpotAvgIntenCh4 | 1 | 100 | | CV\_E1CellBodySpotTotalAreaCh3 | 1 | 100 | | MAD\_E1CrossPointAvgCountCh2 | 1 | 100 | | MAD\_E1NeuriteDendriteMaxCh2 | 1 | 100 | | MAD\_E1NeuriteSpotOverlapAreaCh3 | 2.01840225892216 | 201.840225892216 | | MAD\_E1NeuriteSpotOverlapCountCh4 | 1 | 100 | | MAD\_E1NeuriteSpotTotalAreaCh3 | 2.01840225892216 | 201.840225892216 | | MAD\_E1NeuriteSpotTotalAreaCh4 | 1 | 100 | | MAD\_E1NeuriteSpotTotalIntenCh3 | 8.07360903568864 | 807.360903568864 | | MAD\_E1NeuriteSpotTotalIntenRatioCh4Ch3 | 1 | 100 | | MAD\_E1NeuriteTotalLengthCh2 | 1.42070484581498 | 142.070484581498 | | MAD\_E1NeuriteWidthCh2 | 1.42070484581498 | 142.070484581498 | | MAD\_E2AvgIntenCh4 | 1 | 100 | | MAD\_E2BranchPointTotalCountCh2 | 1 | 100 | | MAD\_E2CellBodyNucCountCh2 | 1 | 100 | | MAD\_E2CellBodyShapeP2ACh2 | 1 | 100 | | MAD\_E2CellBodySpot%OverlapAreaCh3 | 1 | 100 | | MAD\_E2CellBodySpotAvgIntenCh3 | 4 | 400 | | MAD\_E2CellBodySpotTotalAreaCh3 | 2.01840225892216 | 201.840225892216 | | MAD\_E2CellBodySpotTotalAreaRatioCh4Ch3 | 1 | 100 | | MAD\_E2NeuriteAvgIntenCh2 | 4 | 400 | | MAD\_E2NeuriteSpot%OverlapAreaCh4 | 1 | 100 | | MAD\_E2NeuriteSpotTotalAreaCh4 | 1 | 100 | | MEAN\_E1NeuriteSpot%OverlapAreaCh4 | 1 | 100 | | MEAN\_E1NeuriteSpotOverlapCountCh3 | 1 | 100 | | MEAN\_E1NeuriteSpotTotalAreaCh3 | 2.01840225892216 | 201.840225892216 | | MEAN\_E1NeuriteSpotTotalAreaCh4 | 1 | 100 | | MEAN\_E1NeuriteSpotTotalCountCh3 | 1 | 100 | | MEAN\_E1NeuriteSpotTotalCountCh4 | 1 | 100 | | MEAN\_E1NeuriteSpotTotalIntenRatioCh4Ch3 | 1 | 100 | | MEAN\_E1NeuriteWidthCh2 | 1.42070484581498 | 142.070484581498 | | MEAN\_E2BranchPointTotalCountCh2 | 1 | 100 | | MEAN\_E2CellBodyAvgIntenCh2 | 4 | 400 | | MEAN\_E2CellBodyNucCountCh2 | 1 | 100 | | MEAN\_E2CellBodyNucTotalIntenCh1 | 8.07360903568864 | 807.360903568864 | | MEAN\_E2CellBodySpot%OverlapAreaCh3 | 1 | 100 | | MEAN\_E2CellBodySpot%OverlapCountCh3 | 1 | 100 | | MEAN\_E2CellBodySpotOverlapAreaCh4 | 1 | 100 | | MEAN\_E2CellBodySpotTotalAreaCh3 | 2.01840225892216 | 201.840225892216 | | MEAN\_E2CellBodySpotTotalIntenRatioCh4Ch2 | 1 | 100 | | MEAN\_E2CrossPointAvgCountCh2 | 1 | 100 | | MEAN\_E2NeuriteDendriteMaxCh2 | 1 | 100 | | MEAN\_E2NeuriteSpot%OverlapAreaCh4 | 1 | 100 | | MEAN\_E2NeuriteSpotAvgIntenCh4 | 1 | 100 | | MEAN\_E2NeuriteSpotOverlapAreaCh3 | 2.01840225892216 | 201.840225892216 | | MEAN\_E2NeuriteSpotTotalCountCh4 | 1 | 100 | | MEAN\_E2NeuriteSpotTotalIntenCh3 | 8.07360903568864 | 807.360903568864 | | MEAN\_E2NeuriteSpotTotalIntenRatioCh4Ch3 | 1 | 100 | | MEAN\_E2NeuriteTotalLengthCh2 | 1.42070484581498 | 142.070484581498 | | MEDIAN\_E3BranchPointCountPerNeuriteLengthCh2 | 0.703875968992248 | 70.3875968992248 | | MEDIAN\_E3BranchPointTotalCountCh2 | 1 | 100 | | MEDIAN\_E3CellBodyNucCountCh2 | 1 | 100 | | MEDIAN\_E3CellBodyShapeP2ACh2 | 1 | 100 | | MEDIAN\_E3CellBodySpotAvgIntenCh3 | 4 | 400 | | MEDIAN\_E3CellBodySpotOverlapAreaCh4 | 1 | 100 | | MEDIAN\_E3CellBodySpotOverlapCountCh3 | 1 | 100 | | MEDIAN\_E3CellBodySpotTotalIntenCh4 | 1 | 100 | | MEDIAN\_E3CellBodySpotTotalIntenRatioCh4Ch2 | 1 | 100 | | MEDIAN\_E3NeuriteAvgIntenCh2 | 4 | 400 | | MEDIAN\_E3NeuriteMaxLengthWithBranchesCh2 | 1.42070484581498 | 142.070484581498 | | MEDIAN\_E3NeuriteSpot%OverlapAreaCh4 | 1 | 100 | | MEDIAN\_E3NeuriteSpot%OverlapCountCh3 | 1 | 100 | | MEDIAN\_E3NeuriteSpot%OverlapCountCh4 | 1 | 100 | | MEDIAN\_E3NeuriteSpotAvgCountCh3 | 1 | 100 | | MEDIAN\_E3NeuriteSpotAvgIntenCh4 | 1 | 100 | | MEDIAN\_E3NeuriteSpotTotalCountCh4 | 1 | 100 | | MEDIAN\_E3NeuriteSpotTotalIntenRatioCh3Ch2 | 1 | 100 | | MEDIAN\_E3NeuriteTotalLengthCh2 | 1.42070484581498 | 142.070484581498 | | MEDIAN\_NeuriteDendriteMaxCh2 | 1 | 100 | | MEDIAN\_NeuriteMaxLengthWithoutBranchesCh2 | 1.42070484581498 | 142.070484581498 | | MEDIAN\_NeuriteSpotAvgIntenCh3 | 4 | 400 | | MEDIAN\_NeuriteSpotOverlapCountCh4 | 1 | 100 | | %HIGH\_E3CellBodySpotTotalAreaCh4 | 1 | 100 | | %HIGH\_E3CellBodySpotTotalAreaRatioCh4Ch3 | 1 | 100 | | %HIGH\_E3CellBodySpotTotalCountCh3 | 1 | 100 | | %HIGH\_E3CellBodySpotTotalIntenCh3 | 1 | 100 | | %HIGH\_E3CellBodyTotalIntenCh2 | 1 | 100 | | %HIGH\_E3CrossPointTotalCountCh2 | 1 | 100 | | %HIGH\_E3NeuriteCriticalValueCh2 | 1 | 100 | | %HIGH\_E3NeuriteRamificationIndexCh2 | 1 | 100 | | %HIGH\_E3NeuriteSpot%OverlapAreaCh3 | 1 | 100 | | %HIGH\_E3NeuriteSpotAvgIntenCh3 | 1 | 100 | | %HIGH\_E3NeuriteSpotOverlapCountCh4 | 1 | 100 | | %HIGH\_E3NeuriteSpotTotalAreaCh4 | 1 | 100 | | %HIGH\_E3NeuriteSpotTotalCountCh3 | 1 | 100 | | %HIGH\_E3NeuriteSpotTotalIntenRatioCh4Ch3 | 1 | 100 | | %HIGH\_E3NeuriteTotalAreaCh2 | 1 | 100 | | %HIGH\_E3NeuriteTotalCountCh2 | 1 | 100 | | %HIGH\_NeuriteAvgIntenCh2 | 1 | 100 | | %HIGH\_NeuriteMaxLengthWithBranchesCh2 | 1 | 100 | | %HIGH\_NeuriteSpotAvgCountCh4 | 1 | 100 | | %HIGH\_NeuriteSpotOverlapAreaCh3 | 1 | 100 | | %HIGH\_NeuriteSpotTotalAreaCh3 | 1 | 100 | | %HIGH\_NeuriteSpotTotalIntenCh3 | 1 | 100 | | %HIGH\_NeuriteSpotTotalIntenRatioCh4Ch2 | 1 | 100 | | %HIGH\_NeuriteTotalLengthCh2 | 1 | 100 | | %HIGH\_TotalIntenCh4 | 1 | 100 | | BranchPointCountPerNeuriteLengthCh2 | 0.703875968992248 | 70.3875968992248 | | BranchPointTotalCountPerFieldCh2 | 1 | 100 | | BranchPointTotalCountPerNeuriteCh2 | 1 | 100 | | BranchPointTotalCountPerNeuronCh2 | 1 | 100 | | CellBodySpotAvgIntenPerFieldCh3 | 4 | 400 | | CV\_NeuriteSpotTotalIntenRatioCh4Ch2 | 1 | 100 | | CV\_NeuriteTotalLengthCh2 | 1 | 100 | | CV\_NeuriteWidthCh2 | 1 | 100 | | CV\_TotalIntenCh4 | 1 | 100 | | EventType1NeuronCount | 1 | 100 | | MAD\_AvgIntenCh3 | 4 | 400 | | MAD\_BranchPointAvgDistFromCellBodyCh2 | 1.42070484581498 | 142.070484581498 | | MAD\_CellBodyNucTotalIntenCh1 | 8.07360903568864 | 807.360903568864 | | MAD\_CellBodyShapeLWRCh2 | 1 | 100 | | MAD\_CellBodySpot%OverlapCountCh4 | 1 | 100 | | MAD\_CellBodySpotAvgIntenCh4 | 1 | 100 | | MAD\_CellBodySpotTotalAreaCh4 | 1 | 100 | | MAD\_CellBodySpotTotalIntenCh3 | 8.07360903568864 | 807.360903568864 | | MAD\_CellBodySpotTotalIntenRatioCh3Ch2 | 1 | 100 | | MAD\_CellBodyVarIntenCh2 | 1 | 100 | | MAD\_CrossPointAvgCountCh2 | 1 | 100 | | MAD\_E1AvgIntenCh4 | 1 | 100 | | MAD\_E1BranchPointTotalCountCh2 | 1 | 100 | | MAD\_E1CellBodyAvgIntenCh2 | 4 | 400 | | MAD\_E1CellBodyNucCountCh2 | 1 | 100 | | MAD\_E1CellBodySpot%OverlapCountCh3 | 1 | 100 | | MAD\_E1CellBodySpot%OverlapCountCh4 | 1 | 100 | | MAD\_E1CellBodySpotTotalAreaCh4 | 1 | 100 | | MAD\_E1CellBodySpotTotalAreaRatioCh4Ch3 | 1 | 100 | | MAD\_E1CellBodySpotTotalIntenRatioCh3Ch2 | 1 | 100 | | MEAN\_E2NeuriteWidthCh2 | 1.42070484581498 | 142.070484581498 | | MEAN\_E2TotalIntenCh3 | 8.07360903568864 | 807.360903568864 | | MEAN\_E3BranchPointTotalCountCh2 | 1 | 100 | | MEAN\_E3CellBodyNucCountCh2 | 1 | 100 | | MEAN\_E3CellBodyShapeP2ACh2 | 1 | 100 | | MEAN\_E3CellBodySpot%OverlapAreaCh3 | 1 | 100 | | MEAN\_E3CellBodySpotAvgIntenCh3 | 4 | 400 | | MEAN\_E3CellBodySpotOverlapAreaCh4 | 1 | 100 | | MEAN\_E3CellBodySpotTotalIntenCh4 | 1 | 100 | | MEAN\_E3CellBodySpotTotalIntenRatioCh4Ch2 | 1 | 100 | | MEAN\_E3CellBodyVarIntenCh2 | 1 | 100 | | MEAN\_E3CrossPointAvgCountCh2 | 1 | 100 | | MEAN\_E3NeuriteAvgIntenCh2 | 4 | 400 | | MEAN\_E3NeuriteSpot%OverlapCountCh4 | 1 | 100 | | MEAN\_E3NeuriteSpotAvgAreaCh4 | 1 | 100 | | MEAN\_E3NeuriteSpotAvgCountCh3 | 1 | 100 | | MEAN\_E3NeuriteSpotAvgIntenCh4 | 1 | 100 | | MEAN\_E3NeuriteSpotTotalIntenRatioCh3Ch2 | 1 | 100 | | MEAN\_E3NeuriteTotalLengthCh2 | 1.42070484581498 | 142.070484581498 | | MEAN\_E3NeuriteWidthCh2 | 1.42070484581498 | 142.070484581498 | | MEAN\_NeuriteDendriteMaxCh2 | 1 | 100 | | MEAN\_NeuriteMaxLengthWithoutBranchesCh2 | 1.42070484581498 | 142.070484581498 | | MEAN\_NeuriteSpot%OverlapAreaCh3 | 1 | 100 | | MEAN\_NeuriteSpotAvgIntenCh3 | 4 | 400 | | MEAN\_NeuriteSpotOverlapCountCh4 | 1 | 100 | | MEAN\_NeuriteSpotTotalAreaCh4 | 1 | 100 | | MEAN\_NeuriteSpotTotalCountCh3 | 1 | 100 | | MEDIAN\_NeuriteSpotTotalIntenCh4 | 1 | 100 | | NA\_NeuriteSpot%OverlapAreaPerWellCh3 | 0 | 100 | | NA\_NeuriteSpotAvgIntenPerWellCh4 | 1 | 100 | | NA\_NeuriteSpotOverlapTotalAreaPerNeuriteLengthCh3 | 0 | 100 | | NA\_NeuriteSpotOverlapTotalAreaPerWellCh3 | 0 | 100 | | NA\_NeuriteSpotTotalAreaPerNeuriteLengthCh4 | 1 | 100 | | NA\_NeuriteSpotTotalIntenPerNeuriteLengthCh4 | 1 | 100 | | NA\_NeuriteSpotTotalIntenPerWellCh4 | 1 | 100 | | NA\_NeuriteTotalLengthPerWellCh2 | 0 | 100 | | NeuriteSpot%OverlapAreaPerWellCh3 | 1 | 100 | | NeuriteSpot%OverlapCountPerWellCh4 | 1 | 100 | | NeuriteSpotAvgIntenPerFieldCh4 | 1 | 100 | | NeuriteSpotOverlapTotalAreaPerFieldCh3 | 2.01840225892216 | 201.840225892216 | | NeuriteSpotOverlapTotalAreaPerNeuronCh3 | 2.01840225892216 | 201.840225892216 | | NeuriteSpotOverlapTotalAreaPerWellCh3 | 2.01840225892216 | 201.840225892216 | | NeuriteSpotOverlapTotalCountPerFieldCh4 | 1 | 100 | | NeuriteSpotOverlapTotalCountPerWellCh4 | 1 | 100 | | NeuriteSpotTotalAreaPerFieldCh3 | 2.01840225892216 | 201.840225892216 | | NeuriteSpotTotalAreaPerNeuriteCh3 | 2.01840225892216 | 201.840225892216 | | NeuriteSpotTotalAreaPerNeuronCh3 | 2.01840225892216 | 201.840225892216 | | MEAN\_E3BranchPointAvgDistFromCellBodyCh2 | 1.42070484581498 | 142.070484581498 | | MEAN\_E3BranchPointCountPerNeuriteLengthCh2 | 0.703875968992248 | 70.3875968992248 | | MEAN\_E3CellBodyNucAvgIntenCh1 | 4 | 400 | | MEAN\_E3CellBodyNucTotalAreaCh1 | 2.01840225892216 | 201.840225892216 | | MEAN\_E3CellBodyNucTotalIntenCh1 | 8.07360903568864 | 807.360903568864 | | MEAN\_E3CellBodySpotOverlapCountCh4 | 1 | 100 | | MEAN\_E3CellBodySpotTotalAreaCh3 | 2.01840225892216 | 201.840225892216 | | MEAN\_E3CellBodySpotTotalAreaRatioCh4Ch3 | 1 | 100 | | MEAN\_E3CellBodySpotTotalCountCh3 | 1 | 100 | | MEAN\_E3CellBodySpotTotalIntenRatioCh4Ch3 | 1 | 100 | | MEAN\_E3CrossPointTotalCountCh2 | 1 | 100 | | MEAN\_E3NeuriteMaxLengthWithBranchesCh2 | 1.42070484581498 | 142.070484581498 | | MEAN\_E3NeuriteMaxLengthWithoutBranchesCh2 | 1.42070484581498 | 142.070484581498 | | MEAN\_E3NeuriteSpotAvgAreaCh3 | 2.01840225892216 | 201.840225892216 | | MEAN\_E3NeuriteSpotTotalAreaCh3 | 2.01840225892216 | 201.840225892216 | | MEAN\_E3NeuriteSpotTotalAreaRatioCh4Ch3 | 1 | 100 | | MEAN\_E3NeuriteSpotTotalIntenCh3 | 8.07360903568864 | 807.360903568864 | | MEAN\_E3NeuriteSpotTotalIntenRatioCh4Ch2 | 1 | 100 | | MEAN\_E3NeuriteSpotTotalIntenRatioCh4Ch3 | 1 | 100 | | MEAN\_E3NeuriteTotalAreaCh2 | 2.01840225892216 | 201.840225892216 | | MEAN\_NeuriteAvgLengthCh2 | 1.42070484581498 | 142.070484581498 | | MEAN\_NeuriteRamificationIndexCh2 | 1 | 100 | | MEAN\_NeuriteSpot%OverlapAreaCh4 | 1 | 100 | | MEAN\_NeuriteSpot%OverlapCountCh3 | 1 | 100 | | MEAN\_NeuriteSpot%OverlapCountCh4 | 1 | 100 | | MEAN\_NeuriteSpotOverlapAreaCh3 | 2.01840225892216 | 201.840225892216 | | MEAN\_NeuriteSpotOverlapAreaCh4 | 1 | 100 | | MEAN\_NeuriteSpotOverlapCountCh3 | 1 | 100 | | MEDIAN\_NeuriteSpotTotalIntenRatioCh4Ch2 | 1 | 100 | | MEDIAN\_NeuriteSpotTotalIntenRatioCh4Ch3 | 1 | 100 | | MEDIAN\_NeuriteTotalIntenCh2 | 8.07360903568864 | 807.360903568864 | | MEDIAN\_NeuriteVarIntenCh2 | 4 | 400 | | MEDIAN\_NeuriteWidthCh2 | 1.42070484581498 | 142.070484581498 | | NA\_NeuriteSpot%OverlapCountPerWellCh4 | 1 | 100 | | NA\_NeuriteSpotAvgIntenPerNeuriteLengthCh4 | 1 | 100 | | NA\_NeuriteSpotAvgIntenPerWellCh3 | 0 | 100 | | NA\_NeuriteSpotOverlapTotalCountPerNeuriteLengthCh3 | 0 | 100 | | NA\_NeuriteSpotOverlapTotalCountPerWellCh3 | 0 | 100 | | NA\_NeuriteSpotTotalAreaPerWellCh4 | 1 | 100 | | NA\_NeuriteSpotTotalCountPerNeuriteLengthCh3 | 0 | 100 | | NA\_NeuriteSpotTotalCountPerWellCh4 | 1 | 100 | | NA\_NeuriteTotalIntenPerWellCh2 | 0 | 100 | | NeuriteSpotOverlapTotalAreaPerNeuriteLengthCh3 | 1.42070484581498 | 142.070484581498 | | NeuriteSpotOverlapTotalCountPerFieldCh3 | 1 | 100 | | NeuriteSpotOverlapTotalCountPerNeuriteCh3 | 1 | 100 | | NeuriteSpotOverlapTotalCountPerNeuriteLengthCh4 | 1 | 100 | | NeuriteSpotOverlapTotalCountPerNeuronCh4 | 1 | 100 | | NeuriteSpotTotalAreaPerNeuriteLengthCh3 | 1.42070484581498 | 142.070484581498 | | NeuriteSpotTotalAreaPerWellCh4 | 1 | 100 | | NeuriteSpotTotalIntenPerWellCh4 | 1 | 100 | | NeuriteTotalCountPerFieldCh2 | 1 | 100 | | NeuriteTotalIntenPerFieldCh2 | 8.07360903568864 | 807.360903568864 | | NeuriteTotalLengthPerWellCh2 | 1 | 100 | | NeuriteVarIntenPerFieldCh2 | 4 | 400 | | NonNeuronalNucleusCount | 1 | 100 | | SD\_CellBodySpotOverlapCountCh4 | 1 | 100 | | SD\_CellBodySpotTotalAreaCh3 | 2.01840225892216 | 201.840225892216 | | SD\_CellBodySpotTotalAreaRatioCh4Ch3 | 1 | 100 | | SD\_CellBodySpotTotalCountCh3 | 1 | 100 | | SD\_CellBodySpotTotalIntenRatioCh4Ch3 | 1 | 100 | | SD\_E1CellBodyAvgIntenCh2 | 4 | 400 | | SD\_E1CellBodyShapeLWRCh2 | 1 | 100 | | SD\_E1CellBodySpotAvgIntenCh3 | 4 | 400 | | SD\_E1CellBodySpotTotalAreaRatioCh4Ch3 | 1 | 100 | | SD\_E1CellBodySpotTotalIntenCh3 | 8.07360903568864 | 807.360903568864 | | SD\_E1CellBodySpotTotalIntenRatioCh3Ch2 | 1 | 100 | | SD\_E1CellBodySpotTotalIntenRatioCh4Ch2 | 1 | 100 | | SD\_E1CellBodySpotTotalIntenRatioCh4Ch3 | 1 | 100 | | SD\_E1CellBodyTotalIntenCh2 | 8.07360903568864 | 807.360903568864 | | SD\_E1NeuriteCriticalValueCh2 | 1.42070484581498 | 142.070484581498 | | SD\_E1NeuriteDendriteMaxCh2 | 1 | 100 | | SD\_E1NeuriteSpotAvgIntenCh3 | 4 | 400 | | SE\_E1NeuriteWidthCh2 | 1.42070484581498 | 142.070484581498 | | SE\_E1TotalIntenCh4 | 1 | 100 | | SE\_E2CellBodyNucAvgIntenCh1 | 4 | 400 | | SE\_E2CellBodyNucCountCh2 | 1 | 100 | | SE\_E2CellBodyNucTotalIntenCh1 | 8.07360903568864 | 807.360903568864 | | SE\_E2CellBodySpot%OverlapAreaCh4 | 1 | 100 | | SE\_E2CellBodySpotOverlapAreaCh4 | 1 | 100 | | SE\_E2CellBodySpotTotalAreaCh3 | 2.01840225892216 | 201.840225892216 | | SE\_E2CellBodySpotTotalCountCh3 | 1 | 100 | | SE\_E2CrossPointAvgCountCh2 | 1 | 100 | | SE\_E2CrossPointTotalCountCh2 | 1 | 100 | | SE\_E2NeuriteSpot%OverlapAreaCh4 | 1 | 100 | | SE\_E2NeuriteSpot%OverlapCountCh4 | 1 | 100 | | SE\_E2NeuriteSpotAvgAreaCh3 | 2.01840225892216 | 201.840225892216 | | SE\_E2NeuriteSpotAvgIntenCh3 | 4 | 400 | | SE\_E2NeuriteSpotOverlapAreaCh4 | 1 | 100 | | SE\_E2NeuriteSpotTotalCountCh4 | 1 | 100 | | SE\_E2NeuriteSpotTotalIntenCh3 | 8.07360903568864 | 807.360903568864 | | SE\_E2NeuriteTotalAreaCh2 | 2.01840225892216 | 201.840225892216 | | SE\_E2NeuriteTotalIntenCh2 | 8.07360903568864 | 807.360903568864 | | SE\_E2NeuriteWidthCh2 | 1.42070484581498 | 142.070484581498 | | SE\_E2TotalIntenCh4 | 1 | 100 | | SE\_E3CellBodyAreaCh2 | 2.01840225892216 | 201.840225892216 | | SE\_E3CellBodyNucAvgIntenCh1 | 4 | 400 | | SE\_E3CellBodyNucCountCh2 | 1 | 100 | | SE\_E3CellBodySpot%OverlapAreaCh4 | 1 | 100 | | SE\_E3CellBodySpot%OverlapCountCh4 | 1 | 100 | | SE\_E3CellBodySpotAvgIntenCh3 | 4 | 400 | | SE\_E3CellBodySpotOverlapAreaCh4 | 1 | 100 | | MEDIAN\_E2NeuriteSpotOverlapAreaCh3 | 2.01840225892216 | 201.840225892216 | | NeuriteSpotOverlapTotalCountPerWellCh3 | 1 | 100 | | NeuriteSpotTotalAreaPerFieldCh4 | 1 | 100 | | NeuriteSpotTotalAreaPerNeuriteCh4 | 1 | 100 | | MEDIAN\_E1NeuriteRamificationIndexCh2 | 1 | 100 | | MEDIAN\_E1NeuriteSpot%OverlapCountCh3 | 1 | 100 | | MEDIAN\_E1NeuriteSpotAvgAreaCh3 | 2.01840225892216 | 201.840225892216 | | MEDIAN\_E1NeuriteSpotAvgCountCh3 | 1 | 100 | | NeuriteTotalIntenPerWellCh2 | 8.07360903568864 | 807.360903568864 | | NeuriteTotalLengthPerFieldCh2 | 1.42070484581498 | 142.070484581498 | | NeuriteTotalLengthPerNeuriteCh2 | 1.42070484581498 | 142.070484581498 | | NeuriteTotalLengthPerNeuronCh2 | 1.42070484581498 | 142.070484581498 | | NeuronNucleusRatio | 1 | 100 | | SD\_CellBodyNucAvgIntenCh1 | 4 | 400 | | SD\_CellBodyNucTotalAreaCh1 | 2.01840225892216 | 201.840225892216 | | SD\_CellBodySpotAvgIntenCh3 | 4 | 400 | | SD\_CellBodySpotOverlapAreaCh4 | 1 | 100 | | SD\_CellBodySpotOverlapCountCh3 | 1 | 100 | | SD\_CellBodySpotTotalIntenCh4 | 1 | 100 | | SD\_CellBodySpotTotalIntenRatioCh4Ch2 | 1 | 100 | | SD\_CellBodyTotalIntenCh2 | 8.07360903568864 | 807.360903568864 | | SD\_CrossPointTotalCountCh2 | 1 | 100 | | SD\_E1BranchPointAvgCountCh2 | 1 | 100 | | SD\_E1CellBodyAreaCh2 | 2.01840225892216 | 201.840225892216 | | SD\_E1CellBodySpotOverlapAreaCh3 | 2.01840225892216 | 201.840225892216 | | SD\_E1CellBodySpotOverlapAreaCh4 | 1 | 100 | | SD\_E1CellBodySpotOverlapCountCh3 | 1 | 100 | | SD\_E1CellBodySpotOverlapCountCh4 | 1 | 100 | | SD\_E1CellBodySpotTotalCountCh3 | 1 | 100 | | SD\_E1CellBodySpotTotalIntenCh4 | 1 | 100 | | SD\_E1CellBodyVarIntenCh2 | 1 | 100 | | SD\_E1NeuriteAvgLengthCh2 | 1.42070484581498 | 142.070484581498 | | SD\_E1NeuriteRamificationIndexCh2 | 1 | 100 | | SD\_E1NeuriteSpot%OverlapAreaCh3 | 1 | 100 | | SD\_E1NeuriteSpot%OverlapCountCh3 | 1 | 100 | | SD\_E1NeuriteSpot%OverlapCountCh4 | 1 | 100 | | SD\_E1NeuriteSpotAvgAreaCh3 | 2.01840225892216 | 201.840225892216 | | SD\_E1NeuriteSpotAvgAreaCh4 | 1 | 100 | | SE\_E1NeuriteTotalIntenCh2 | 8.07360903568864 | 807.360903568864 | | SE\_E1NeuriteVarIntenCh2 | 4 | 400 | | SE\_E2AvgIntenCh3 | 4 | 400 | | SE\_E2BranchPointAvgCountCh2 | 1 | 100 | | SE\_E2BranchPointAvgDistFromCellBodyCh2 | 1.42070484581498 | 142.070484581498 | | SE\_E2CellBodyAreaCh2 | 2.01840225892216 | 201.840225892216 | | SE\_E2CellBodyShapeLWRCh2 | 1 | 100 | | SE\_E2CellBodySpotOverlapCountCh4 | 1 | 100 | | SE\_E2CellBodySpotTotalCountCh4 | 1 | 100 | | SE\_E2CellBodySpotTotalIntenCh3 | 8.07360903568864 | 807.360903568864 | | SE\_E2CellBodySpotTotalIntenCh4 | 1 | 100 | | SE\_E2CellBodySpotTotalIntenRatioCh3Ch2 | 1 | 100 | | SE\_E2NeuriteAvgLengthCh2 | 1.42070484581498 | 142.070484581498 | | SE\_E2NeuriteMaxLengthWithoutBranchesCh2 | 1.42070484581498 | 142.070484581498 | | SE\_E2NeuriteSpotAvgAreaCh4 | 1 | 100 | | SE\_CellBodySpot%OverlapCountCh4 | 1 | 100 | | SE\_CellBodySpotOverlapCountCh4 | 1 | 100 | | SE\_CellBodySpotTotalAreaCh3 | 2.01840225892216 | 201.840225892216 | | MEDIAN\_E1NeuriteAvgLengthCh2 | 1.42070484581498 | 142.070484581498 | | MEDIAN\_E1NeuriteMaxLengthWithBranchesCh2 | 1.42070484581498 | 142.070484581498 | | MEDIAN\_E1NeuriteSpot%OverlapAreaCh3 | 1 | 100 | | MEDIAN\_E1NeuriteSpot%OverlapAreaCh4 | 1 | 100 | | MEDIAN\_E1NeuriteSpot%OverlapCountCh4 | 1 | 100 | | NeuriteSpotTotalIntenPerNeuronCh3 | 8.07360903568864 | 807.360903568864 | | NeuriteSpotTotalIntenPerWellCh3 | 8.07360903568864 | 807.360903568864 | | NeuriteTotalCountPerNeuronCh2 | 1 | 100 | | NeuriteTotalCountPerWellCh2 | 1 | 100 | | NeuronalNucleusCount | 1 | 100 | | SD\_AvgIntenCh4 | 1 | 100 | | SD\_BranchPointAvgCountCh2 | 1 | 100 | | SD\_BranchPointAvgDistFromCellBodyCh2 | 1.42070484581498 | 142.070484581498 | | SD\_BranchPointCountPerNeuriteLengthCh2 | 0.703875968992248 | 70.3875968992248 | | SD\_CellBodyAreaCh2 | 2.01840225892216 | 201.840225892216 | | SD\_CellBodyNucCountCh2 | 1 | 100 | | SD\_CellBodyShapeP2ACh2 | 1 | 100 | | SD\_CellBodySpot%OverlapAreaCh3 | 1 | 100 | | SD\_CellBodySpot%OverlapCountCh3 | 1 | 100 | | SD\_CellBodySpotOverlapAreaCh3 | 2.01840225892216 | 201.840225892216 | | SD\_CellBodySpotTotalAreaCh4 | 1 | 100 | | SD\_CellBodySpotTotalCountCh4 | 1 | 100 | | SD\_E1AvgIntenCh3 | 4 | 400 | | SD\_E1BranchPointAvgDistFromCellBodyCh2 | 1.42070484581498 | 142.070484581498 | | SD\_E1BranchPointCountPerNeuriteLengthCh2 | 0.703875968992248 | 70.3875968992248 | | SD\_E1CellBodyNucAvgIntenCh1 | 4 | 400 | | SD\_E1CellBodyNucTotalAreaCh1 | 2.01840225892216 | 201.840225892216 | | SD\_E1CellBodyNucTotalIntenCh1 | 8.07360903568864 | 807.360903568864 | | SD\_E1CellBodySpot%OverlapAreaCh3 | 1 | 100 | | SD\_E1CellBodySpot%OverlapAreaCh4 | 1 | 100 | | SD\_E1CellBodySpotTotalCountCh4 | 1 | 100 | | SD\_E1CrossPointTotalCountCh2 | 1 | 100 | | SD\_E1NeuriteMaxLengthWithBranchesCh2 | 1.42070484581498 | 142.070484581498 | | SD\_E1NeuriteMaxLengthWithoutBranchesCh2 | 1.42070484581498 | 142.070484581498 | | SD\_E1NeuriteSpot%OverlapAreaCh4 | 1 | 100 | | SD\_E1NeuriteSpotAvgCountCh3 | 1 | 100 | | SD\_E1NeuriteSpotAvgCountCh4 | 1 | 100 | | SE\_E2BranchPointTotalCountCh2 | 1 | 100 | | SE\_E2CellBodyAvgIntenCh2 | 4 | 400 | | SE\_E2CellBodySpot%OverlapAreaCh3 | 1 | 100 | | SE\_E2CellBodySpot%OverlapCountCh3 | 1 | 100 | | SE\_E2CellBodySpotAvgIntenCh4 | 1 | 100 | | SE\_E2CellBodySpotOverlapAreaCh3 | 2.01840225892216 | 201.840225892216 | | SE\_E2CellBodySpotOverlapCountCh3 | 1 | 100 | | SE\_E2CellBodySpotTotalIntenRatioCh4Ch2 | 1 | 100 | | SE\_E2CellBodySpotTotalIntenRatioCh4Ch3 | 1 | 100 | | SE\_E2CellBodyTotalIntenCh2 | 8.07360903568864 | 807.360903568864 | | SE\_E2NeuriteDendriteMaxCh2 | 1 | 100 | | MEDIAN\_E2NeuriteSpotOverlapCountCh3 | 1 | 100 | | MEDIAN\_E2NeuriteSpotTotalAreaCh4 | 1 | 100 | | MEDIAN\_E2NeuriteSpotTotalIntenCh4 | 1 | 100 | | MEDIAN\_E2NeuriteVarIntenCh2 | 4 | 400 | | SD\_E1NeuriteSpotAvgIntenCh4 | 1 | 100 | | SD\_E1NeuriteSpotOverlapCountCh3 | 1 | 100 | | SD\_E1NeuriteSpotTotalIntenRatioCh3Ch2 | 1 | 100 | | SD\_E1NeuriteSpotTotalIntenRatioCh4Ch2 | 1 | 100 | | SD\_E1NeuriteSpotTotalIntenRatioCh4Ch3 | 1 | 100 | | SD\_E1NeuriteVarIntenCh2 | 4 | 400 | | SD\_E2CellBodyAvgIntenCh2 | 4 | 400 | | SD\_E2CellBodySpot%OverlapCountCh3 | 1 | 100 | | SD\_E2CellBodySpotAvgIntenCh4 | 1 | 100 | | SD\_E2CellBodySpotOverlapAreaCh3 | 2.01840225892216 | 201.840225892216 | | SD\_E2CellBodySpotOverlapCountCh3 | 1 | 100 | | SD\_E2CellBodySpotTotalIntenCh4 | 1 | 100 | | SD\_E2CellBodySpotTotalIntenRatioCh4Ch2 | 1 | 100 | | SD\_E2CellBodyTotalIntenCh2 | 8.07360903568864 | 807.360903568864 | | SD\_E2CellBodyVarIntenCh2 | 1 | 100 | | SD\_E2NeuriteCriticalValueCh2 | 1.42070484581498 | 142.070484581498 | | SD\_E2NeuriteDendriteMaxCh2 | 1 | 100 | | SD\_E2NeuriteRamificationIndexCh2 | 1 | 100 | | SD\_E2NeuriteSpot%OverlapAreaCh3 | 1 | 100 | | SD\_E2NeuriteSpot%OverlapCountCh3 | 1 | 100 | | SD\_E2NeuriteSpotAvgIntenCh4 | 1 | 100 | | SD\_E2NeuriteSpotOverlapAreaCh3 | 2.01840225892216 | 201.840225892216 | | SD\_E2NeuriteSpotTotalIntenCh4 | 1 | 100 | | SD\_E2NeuriteSpotTotalIntenRatioCh4Ch2 | 1 | 100 | | SD\_E2NeuriteTotalCountCh2 | 1 | 100 | | SD\_E3AvgIntenCh3 | 4 | 400 | | SD\_E3BranchPointTotalCountCh2 | 1 | 100 | | SD\_E3CellBodyAvgIntenCh2 | 4 | 400 | | SE\_E3CellBodySpotTotalIntenCh3 | 8.07360903568864 | 807.360903568864 | | SE\_E3CellBodySpotTotalIntenRatioCh3Ch2 | 1 | 100 | | SE\_E3CrossPointAvgCountCh2 | 1 | 100 | | SE\_E3NeuriteAvgIntenCh2 | 4 | 400 | | SE\_E3NeuriteSpot%OverlapCountCh4 | 1 | 100 | | SE\_E3NeuriteSpotAvgIntenCh3 | 4 | 400 | | SE\_E3NeuriteSpotOverlapAreaCh4 | 1 | 100 | | SE\_E3NeuriteSpotOverlapCountCh3 | 1 | 100 | | SE\_E3NeuriteSpotOverlapCountCh4 | 1 | 100 | | SE\_E3NeuriteSpotTotalIntenRatioCh3Ch2 | 1 | 100 | | SE\_E3NeuriteTotalIntenCh2 | 8.07360903568864 | 807.360903568864 | | SE\_E3NeuriteWidthCh2 | 1.42070484581498 | 142.070484581498 | | SE\_NeuriteMaxLengthWithBranchesCh2 | 1.42070484581498 | 142.070484581498 | | SE\_NeuriteMaxLengthWithoutBranchesCh2 | 1.42070484581498 | 142.070484581498 | | SE\_NeuriteSpot%OverlapAreaCh3 | 1 | 100 | | SE\_NeuriteSpotAvgAreaCh4 | 1 | 100 | | SE\_NeuriteSpotAvgCountCh4 | 1 | 100 | | SE\_NeuriteSpotTotalAreaCh4 | 1 | 100 | | SE\_NeuriteTotalAreaCh2 | 2.01840225892216 | 201.840225892216 | | SE\_TotalIntenCh3 | 8.07360903568864 | 807.360903568864 | | SE\_E2NeuriteRamificationIndexCh2 | 1 | 100 | | SE\_E2NeuriteSpot%OverlapAreaCh3 | 1 | 100 | | SE\_E2NeuriteSpotAvgIntenCh4 | 1 | 100 | | SE\_E2NeuriteSpotOverlapAreaCh3 | 2.01840225892216 | 201.840225892216 | | SE\_E2NeuriteSpotTotalIntenCh4 | 1 | 100 | | SE\_E2NeuriteSpotTotalIntenRatioCh4Ch2 | 1 | 100 | | SE\_E2NeuriteTotalCountCh2 | 1 | 100 | | SE\_E2NeuriteTotalLengthCh2 | 1.42070484581498 | 142.070484581498 | | SE\_E2TotalIntenCh3 | 8.07360903568864 | 807.360903568864 | | SE\_E3AvgIntenCh3 | 4 | 400 | | SE\_E3BranchPointAvgCountCh2 | 1 | 100 | | SE\_E3BranchPointTotalCountCh2 | 1 | 100 | | SE\_E3CellBodySpot%OverlapAreaCh3 | 1 | 100 | | SE\_E3CellBodySpotAvgIntenCh4 | 1 | 100 | | SD\_E1NeuriteTotalAreaCh2 | 2.01840225892216 | 201.840225892216 | | SD\_E1TotalIntenCh4 | 1 | 100 | | SD\_E2BranchPointAvgDistFromCellBodyCh2 | 1.42070484581498 | 142.070484581498 | | SD\_E2CellBodyNucAvgIntenCh1 | 4 | 400 | | SD\_E2CellBodyNucCountCh2 | 1 | 100 | | SD\_E2CellBodyNucTotalIntenCh1 | 8.07360903568864 | 807.360903568864 | | SD\_E2CellBodySpot%OverlapAreaCh4 | 1 | 100 | | SD\_E2CellBodySpot%OverlapCountCh4 | 1 | 100 | | SD\_E2CellBodySpotOverlapAreaCh4 | 1 | 100 | | SD\_E2CellBodySpotTotalAreaCh3 | 2.01840225892216 | 201.840225892216 | | SD\_E2CellBodySpotTotalCountCh3 | 1 | 100 | | SD\_E2CrossPointAvgCountCh2 | 1 | 100 | | SD\_E2CrossPointTotalCountCh2 | 1 | 100 | | SD\_E2NeuriteMaxLengthWithoutBranchesCh2 | 1.42070484581498 | 142.070484581498 | | SD\_E2NeuriteSpot%OverlapAreaCh4 | 1 | 100 | | SD\_E2NeuriteSpot%OverlapCountCh4 | 1 | 100 | | SD\_E2NeuriteSpotAvgAreaCh3 | 2.01840225892216 | 201.840225892216 | | SD\_E2NeuriteSpotOverlapAreaCh4 | 1 | 100 | | SD\_E2NeuriteSpotTotalAreaCh3 | 2.01840225892216 | 201.840225892216 | | SD\_E2NeuriteSpotTotalIntenCh3 | 8.07360903568864 | 807.360903568864 | | SD\_E2NeuriteTotalAreaCh2 | 2.01840225892216 | 201.840225892216 | | SD\_E2NeuriteWidthCh2 | 1.42070484581498 | 142.070484581498 | | SD\_E3CellBodyAreaCh2 | 2.01840225892216 | 201.840225892216 | | SE\_E3CellBodySpotTotalIntenCh4 | 1 | 100 | | SE\_E3CellBodySpotTotalIntenRatioCh4Ch2 | 1 | 100 | | SE\_E3CellBodyVarIntenCh2 | 1 | 100 | | SE\_E3NeuriteAvgLengthCh2 | 1.42070484581498 | 142.070484581498 | | SE\_E3NeuriteRamificationIndexCh2 | 1 | 100 | | SE\_E3NeuriteSpotAvgAreaCh4 | 1 | 100 | | SE\_E3NeuriteSpotAvgCountCh3 | 1 | 100 | | SE\_E3NeuriteSpotAvgIntenCh4 | 1 | 100 | | SE\_E3NeuriteSpotTotalCountCh3 | 1 | 100 | | SE\_E3NeuriteSpotTotalIntenCh4 | 1 | 100 | | SE\_E3NeuriteTotalCountCh2 | 1 | 100 | | SE\_E3NeuriteTotalLengthCh2 | 1.42070484581498 | 142.070484581498 | | SE\_E3TotalIntenCh3 | 8.07360903568864 | 807.360903568864 | | SE\_NeuriteCriticalValueCh2 | 1.42070484581498 | 142.070484581498 | | SE\_NeuriteDendriteMaxCh2 | 1 | 100 | | SE\_E2NeuriteSpotAvgCountCh4 | 1 | 100 | | SE\_E2NeuriteSpotOverlapCountCh3 | 1 | 100 | | SE\_E2NeuriteSpotTotalAreaCh3 | 2.01840225892216 | 201.840225892216 | | SE\_E2NeuriteSpotTotalAreaRatioCh4Ch3 | 1 | 100 | | SE\_E2NeuriteSpotTotalCountCh3 | 1 | 100 | | SE\_E2NeuriteVarIntenCh2 | 4 | 400 | | SE\_E3BranchPointAvgDistFromCellBodyCh2 | 1.42070484581498 | 142.070484581498 | | SE\_E3CellBodyAvgIntenCh2 | 4 | 400 | | SE\_E3CellBodyNucTotalIntenCh1 | 8.07360903568864 | 807.360903568864 | | SE\_E3CellBodyShapeLWRCh2 | 1 | 100 | | SE\_E3CellBodySpot%OverlapCountCh3 | 1 | 100 | | SE\_E3CellBodySpotOverlapCountCh4 | 1 | 100 | | SE\_E3CellBodySpotTotalAreaCh3 | 2.01840225892216 | 201.840225892216 | | MEDIAN\_E2NeuriteTotalIntenCh2 | 8.07360903568864 | 807.360903568864 | | MEDIAN\_E2TotalIntenCh4 | 1 | 100 | | SD\_E1NeuriteSpotTotalAreaRatioCh4Ch3 | 1 | 100 | | SD\_E1NeuriteTotalCountCh2 | 1 | 100 | | SD\_E1NeuriteTotalIntenCh2 | 8.07360903568864 | 807.360903568864 | | SD\_E2AvgIntenCh3 | 4 | 400 | | SD\_E2BranchPointAvgCountCh2 | 1 | 100 | | SD\_E2CellBodyAreaCh2 | 2.01840225892216 | 201.840225892216 | | SD\_E2CellBodyShapeLWRCh2 | 1 | 100 | | SD\_E2CellBodySpotOverlapCountCh4 | 1 | 100 | | SD\_E2CellBodySpotTotalCountCh4 | 1 | 100 | | SD\_E2CellBodySpotTotalIntenCh3 | 8.07360903568864 | 807.360903568864 | | SD\_E2CellBodySpotTotalIntenRatioCh3Ch2 | 1 | 100 | | SD\_E2CellBodySpotTotalIntenRatioCh4Ch3 | 1 | 100 | | SD\_E2NeuriteAvgLengthCh2 | 1.42070484581498 | 142.070484581498 | | SD\_E2NeuriteSpotAvgAreaCh4 | 1 | 100 | | SD\_E2NeuriteSpotAvgCountCh4 | 1 | 100 | | SD\_E2NeuriteSpotAvgIntenCh3 | 4 | 400 | | SD\_E2NeuriteSpotOverlapCountCh3 | 1 | 100 | | SD\_E2NeuriteSpotTotalAreaRatioCh4Ch3 | 1 | 100 | | SD\_E2NeuriteSpotTotalCountCh3 | 1 | 100 | | SD\_E2NeuriteTotalIntenCh2 | 8.07360903568864 | 807.360903568864 | | SD\_E2NeuriteVarIntenCh2 | 4 | 400 | | SD\_E2TotalIntenCh4 | 1 | 100 | | SD\_E3BranchPointAvgCountCh2 | 1 | 100 | | SD\_E3BranchPointAvgDistFromCellBodyCh2 | 1.42070484581498 | 142.070484581498 | | SE\_E3CellBodySpotTotalCountCh4 | 1 | 100 | | SE\_E3CellBodySpotTotalIntenRatioCh4Ch3 | 1 | 100 | | SE\_E3NeuriteDendriteMaxCh2 | 1 | 100 | | SE\_E3NeuriteMaxLengthWithoutBranchesCh2 | 1.42070484581498 | 142.070484581498 | | SE\_E3NeuriteSpotAvgCountCh4 | 1 | 100 | | SE\_E3NeuriteSpotOverlapAreaCh3 | 2.01840225892216 | 201.840225892216 | | SE\_E3NeuriteSpotTotalAreaCh3 | 2.01840225892216 | 201.840225892216 | | SE\_E3NeuriteSpotTotalAreaRatioCh4Ch3 | 1 | 100 | | SE\_E3NeuriteSpotTotalIntenCh3 | 8.07360903568864 | 807.360903568864 | | SE\_E3NeuriteSpotTotalIntenRatioCh4Ch2 | 1 | 100 | | SE\_E3NeuriteVarIntenCh2 | 4 | 400 | | SE\_E3TotalIntenCh4 | 1 | 100 | | SE\_NeuriteAvgLengthCh2 | 1.42070484581498 | 142.070484581498 | | SE\_TotalIntenCh4 | 1 | 100 | | NeuriteSpotTotalCountPerNeuriteCh4 | 1 | 100 | | NeuriteSpotTotalCountPerNeuriteLengthCh3 | 0.703875968992248 | 70.3875968992248 | | NeuriteSpotTotalCountPerNeuronCh3 | 1 | 100 | | NeuriteSpotTotalIntenPerFieldCh3 | 8.07360903568864 | 807.360903568864 | | NeuriteSpotTotalIntenPerNeuriteCh3 | 8.07360903568864 | 807.360903568864 | | SE\_AvgIntenCh4 | 1 | 100 | | SE\_BranchPointAvgDistFromCellBodyCh2 | 1.42070484581498 | 142.070484581498 | | SE\_BranchPointCountPerNeuriteLengthCh2 | 0.703875968992248 | 70.3875968992248 | | SE\_CellBodyAreaCh2 | 2.01840225892216 | 201.840225892216 | | SE\_CellBodyShapeLWRCh2 | 1 | 100 | | SE\_CellBodyShapeP2ACh2 | 1 | 100 | | SE\_CellBodySpot%OverlapAreaCh3 | 1 | 100 | | SE\_CellBodySpot%OverlapCountCh3 | 1 | 100 | | SE\_CellBodySpotOverlapAreaCh3 | 2.01840225892216 | 201.840225892216 | | SE\_CellBodySpotTotalAreaCh4 | 1 | 100 | | SE\_CellBodySpotTotalCountCh4 | 1 | 100 | | SE\_CellBodySpotTotalIntenCh4 | 1 | 100 | | SE\_CrossPointTotalCountCh2 | 1 | 100 | | SE\_E1AvgIntenCh3 | 4 | 400 | | SE\_E1AvgIntenCh4 | 1 | 100 | | SE\_E1BranchPointAvgDistFromCellBodyCh2 | 1.42070484581498 | 142.070484581498 | | SE\_E1CellBodyNucAvgIntenCh1 | 4 | 400 | | SE\_E1CellBodyNucTotalAreaCh1 | 2.01840225892216 | 201.840225892216 | | SE\_E1CellBodyNucTotalIntenCh1 | 8.07360903568864 | 807.360903568864 | | SE\_E1CellBodySpot%OverlapAreaCh4 | 1 | 100 | | SE\_E1CellBodySpotTotalCountCh4 | 1 | 100 | | SE\_E1CrossPointAvgCountCh2 | 1 | 100 | | SE\_E1CrossPointTotalCountCh2 | 1 | 100 | | SE\_E1NeuriteMaxLengthWithoutBranchesCh2 | 1.42070484581498 | 142.070484581498 | | SE\_E1NeuriteSpot%OverlapAreaCh4 | 1 | 100 | | SE\_E1NeuriteSpotAvgCountCh3 | 1 | 100 | | SE\_E1NeuriteSpotAvgCountCh4 | 1 | 100 | | SE\_E1NeuriteSpotTotalAreaCh3 | 2.01840225892216 | 201.840225892216 | | SE\_E1NeuriteSpotTotalAreaCh4 | 1 | 100 | | SE\_E1NeuriteSpotTotalCountCh3 | 1 | 100 | | SE\_E1NeuriteSpotTotalCountCh4 | 1 | 100 | | SE\_E1NeuriteTotalAreaCh2 | 2.01840225892216 | 201.840225892216 | | MEDIAN\_NeuriteSpotAvgIntenCh4 | 1 | 100 | | MEDIAN\_NeuriteSpotTotalAreaCh3 | 2.01840225892216 | 201.840225892216 | | SD\_E3CellBodySpot%OverlapAreaCh3 | 1 | 100 | | SD\_E3CellBodySpotAvgIntenCh4 | 1 | 100 | | SD\_E3CellBodySpotOverlapAreaCh3 | 2.01840225892216 | 201.840225892216 | | SD\_E3CellBodySpotTotalAreaCh4 | 1 | 100 | | SD\_E3CellBodySpotTotalIntenCh4 | 1 | 100 | | SD\_E3CellBodyVarIntenCh2 | 1 | 100 | | SD\_E3NeuriteDendriteMaxCh2 | 1 | 100 | | SD\_E3NeuriteRamificationIndexCh2 | 1 | 100 | | SD\_E3NeuriteSpotAvgAreaCh4 | 1 | 100 | | SD\_E3NeuriteSpotTotalAreaCh4 | 1 | 100 | | SD\_E3NeuriteSpotTotalCountCh3 | 1 | 100 | | SD\_E3NeuriteSpotTotalIntenCh4 | 1 | 100 | | SE\_NeuriteSpotAvgAreaCh3 | 2.01840225892216 | 201.840225892216 | | SE\_NeuriteSpotAvgIntenCh3 | 4 | 400 | | SE\_NeuriteSpotOverlapCountCh4 | 1 | 100 | | SE\_NeuriteSpotTotalAreaRatioCh4Ch3 | 1 | 100 | | SE\_NeuriteSpotTotalIntenCh3 | 8.07360903568864 | 807.360903568864 | | SE\_NeuriteSpotTotalIntenRatioCh3Ch2 | 1 | 100 | | SE\_NeuriteSpotTotalIntenRatioCh4Ch3 | 1 | 100 | | SE\_NeuriteVarIntenCh2 | 4 | 400 | | ValidNeuronCount\* | 1 | 100 | | SE\_CellBodySpotTotalAreaRatioCh4Ch3 | 1 | 100 | | SE\_CellBodySpotTotalCountCh3 | 1 | 100 | | SE\_CellBodySpotTotalIntenCh3 | 8.07360903568864 | 807.360903568864 | | SE\_E1CellBodyAvgIntenCh2 | 4 | 400 | | SE\_E1CellBodyShapeLWRCh2 | 1 | 100 | | SE\_E1CellBodySpotAvgIntenCh3 | 4 | 400 | | SE\_E1CellBodySpotOverlapCountCh3 | 1 | 100 | | SE\_E1CellBodySpotTotalAreaRatioCh4Ch3 | 1 | 100 | | SE\_E1CellBodySpotTotalIntenRatioCh3Ch2 | 1 | 100 | | SE\_E1CellBodySpotTotalIntenRatioCh4Ch2 | 1 | 100 | | SE\_E1CellBodySpotTotalIntenRatioCh4Ch3 | 1 | 100 | | SE\_E1CellBodyTotalIntenCh2 | 8.07360903568864 | 807.360903568864 | | SE\_E1NeuriteCriticalValueCh2 | 1.42070484581498 | 142.070484581498 | | SE\_E1NeuriteDendriteMaxCh2 | 1 | 100 | | SE\_E1NeuriteRamificationIndexCh2 | 1 | 100 | | SE\_E1NeuriteSpot%OverlapCountCh3 | 1 | 100 | | SE\_E1NeuriteSpotOverlapCountCh3 | 1 | 100 | | SE\_E1NeuriteSpotTotalAreaRatioCh4Ch3 | 1 | 100 | | SE\_E1NeuriteSpotTotalIntenRatioCh3Ch2 | 1 | 100 | | SE\_E1NeuriteSpotTotalIntenRatioCh4Ch2 | 1 | 100 | | SE\_E1NeuriteSpotTotalIntenRatioCh4Ch3 | 1 | 100 | | MEDIAN\_E3NeuriteTotalAreaCh2 | 2.01840225892216 | 201.840225892216 | | MEDIAN\_E3NeuriteWidthCh2 | 1.42070484581498 | 142.070484581498 | | MEDIAN\_NeuriteAvgLengthCh2 | 1.42070484581498 | 142.070484581498 | | MEDIAN\_NeuriteSpot%OverlapAreaCh3 | 1 | 100 | | MEDIAN\_NeuriteSpot%OverlapAreaCh4 | 1 | 100 | | MEDIAN\_NeuriteSpot%OverlapCountCh3 | 1 | 100 | | MEDIAN\_NeuriteSpotAvgCountCh4 | 1 | 100 | | MEDIAN\_NeuriteSpotOverlapAreaCh3 | 2.01840225892216 | 201.840225892216 | | MEDIAN\_NeuriteSpotOverlapAreaCh4 | 1 | 100 | | MEDIAN\_NeuriteSpotOverlapCountCh3 | 1 | 100 | | SD\_E3CellBodyNucAvgIntenCh1 | 4 | 400 | | SD\_E3CellBodyNucCountCh2 | 1 | 100 | | SD\_E3CellBodySpot%OverlapCountCh4 | 1 | 100 | | SD\_E3CellBodySpotAvgIntenCh3 | 4 | 400 | | SD\_E3CellBodySpotOverlapAreaCh4 | 1 | 100 | | SD\_E3CellBodySpotOverlapCountCh4 | 1 | 100 | | SD\_E3CellBodySpotTotalAreaCh3 | 2.01840225892216 | 201.840225892216 | | SD\_E3CellBodySpotTotalIntenCh3 | 8.07360903568864 | 807.360903568864 | | SD\_E3CrossPointAvgCountCh2 | 1 | 100 | | SD\_E3CrossPointTotalCountCh2 | 1 | 100 | | SD\_E3NeuriteSpot%OverlapCountCh4 | 1 | 100 | | SD\_E3NeuriteTotalCountCh2 | 1 | 100 | | SD\_E3NeuriteTotalLengthCh2 | 1.42070484581498 | 142.070484581498 | | SD\_E3TotalIntenCh3 | 8.07360903568864 | 807.360903568864 | | SD\_NeuriteAvgIntenCh2 | 4 | 400 | | SD\_NeuriteCriticalValueCh2 | 1.42070484581498 | 142.070484581498 | | SD\_NeuriteSpotAvgAreaCh3 | 2.01840225892216 | 201.840225892216 | | SD\_NeuriteSpotAvgIntenCh3 | 4 | 400 | | SD\_NeuriteSpotTotalAreaRatioCh4Ch3 | 1 | 100 | | SD\_NeuriteSpotTotalIntenCh3 | 8.07360903568864 | 807.360903568864 | | SD\_NeuriteSpotTotalIntenRatioCh4Ch2 | 1 | 100 | | SD\_NeuriteSpotTotalIntenRatioCh4Ch3 | 1 | 100 | | SD\_NeuriteVarIntenCh2 | 4 | 400 | | SE\_AvgIntenCh3 | 4 | 400 | | SE\_NeuriteRamificationIndexCh2 | 1 | 100 | | SE\_NeuriteSpot%OverlapAreaCh4 | 1 | 100 | | SE\_NeuriteSpot%OverlapCountCh3 | 1 | 100 | | SE\_NeuriteSpot%OverlapCountCh4 | 1 | 100 | | SE\_NeuriteSpotAvgCountCh3 | 1 | 100 | | SE\_NeuriteSpotAvgIntenCh4 | 1 | 100 | | SE\_NeuriteSpotTotalCountCh4 | 1 | 100 | | SE\_NeuriteTotalCountCh2 | 1 | 100 | | SE\_NeuriteTotalIntenCh2 | 8.07360903568864 | 807.360903568864 | | SelectedNeuronCount | 1 | 100 | | ValidNucleusCount | 1 | 100 | | NeuriteSpotTotalAreaPerNeuriteLengthCh4 | 1 | 100 | | NeuriteSpotTotalAreaPerNeuronCh4 | 1 | 100 | | NeuriteSpotTotalCountPerWellCh3 | 1 | 100 | | NeuriteSpotTotalIntenPerNeuriteLengthCh3 | 8.07360903568864 | 807.360903568864 | | SE\_BranchPointAvgCountCh2 | 1 | 100 | | SE\_CellBodyNucAvgIntenCh1 | 4 | 400 | | SE\_CellBodySpotAvgIntenCh3 | 4 | 400 | | SE\_CellBodySpotOverlapAreaCh4 | 1 | 100 | | SE\_CellBodySpotOverlapCountCh3 | 1 | 100 | | SE\_CellBodySpotTotalIntenRatioCh4Ch2 | 1 | 100 | | SE\_CellBodyTotalIntenCh2 | 8.07360903568864 | 807.360903568864 | | SE\_E1BranchPointAvgCountCh2 | 1 | 100 | | SE\_E1CellBodyAreaCh2 | 2.01840225892216 | 201.840225892216 | | SE\_E1CellBodySpotOverlapAreaCh3 | 2.01840225892216 | 201.840225892216 | | SE\_E1CellBodySpotOverlapAreaCh4 | 1 | 100 | | SE\_E1CellBodySpotOverlapCountCh4 | 1 | 100 | | SE\_E1CellBodySpotTotalCountCh3 | 1 | 100 | | SE\_E1CellBodySpotTotalIntenCh3 | 8.07360903568864 | 807.360903568864 | | SE\_E1CellBodySpotTotalIntenCh4 | 1 | 100 | | SE\_E1CellBodyVarIntenCh2 | 1 | 100 | | SE\_E1NeuriteAvgLengthCh2 | 1.42070484581498 | 142.070484581498 | | SE\_E1NeuriteSpot%OverlapAreaCh3 | 1 | 100 | | SE\_E1NeuriteSpot%OverlapCountCh4 | 1 | 100 | | SE\_E1NeuriteSpotAvgAreaCh3 | 2.01840225892216 | 201.840225892216 | | SE\_E1NeuriteSpotAvgAreaCh4 | 1 | 100 | | SE\_E1NeuriteSpotAvgIntenCh3 | 4 | 400 | | SE\_E1NeuriteSpotAvgIntenCh4 | 1 | 100 | | SE\_E1NeuriteTotalCountCh2 | 1 | 100 | | MEDIAN\_NeuriteMaxLengthWithBranchesCh2 | 1.42070484581498 | 142.070484581498 | | MEDIAN\_NeuriteRamificationIndexCh2 | 1 | 100 | | MEDIAN\_NeuriteSpot%OverlapCountCh4 | 1 | 100 | | MEDIAN\_NeuriteSpotAvgAreaCh4 | 1 | 100 | | MEDIAN\_NeuriteSpotTotalAreaRatioCh4Ch3 | 1 | 100 | | SD\_E3CellBodyNucTotalIntenCh1 | 8.07360903568864 | 807.360903568864 | | SD\_E3CellBodyShapeLWRCh2 | 1 | 100 | | SD\_E3CellBodySpot%OverlapAreaCh4 | 1 | 100 | | SD\_E3CellBodySpot%OverlapCountCh3 | 1 | 100 | | SD\_E3CellBodySpotTotalCountCh4 | 1 | 100 | | SD\_E3CellBodySpotTotalIntenRatioCh3Ch2 | 1 | 100 | | SD\_E3CellBodySpotTotalIntenRatioCh4Ch3 | 1 | 100 | | SD\_E3NeuriteAvgLengthCh2 | 1.42070484581498 | 142.070484581498 | | SD\_E3NeuriteSpotAvgIntenCh3 | 4 | 400 | | SD\_E3NeuriteSpotOverlapCountCh3 | 1 | 100 | | SD\_E3NeuriteSpotOverlapCountCh4 | 1 | 100 | | SD\_E3NeuriteSpotTotalAreaRatioCh4Ch3 | 1 | 100 | | SD\_E3NeuriteSpotTotalIntenRatioCh3Ch2 | 1 | 100 | | SD\_E3NeuriteTotalAreaCh2 | 2.01840225892216 | 201.840225892216 | | SD\_E3NeuriteTotalIntenCh2 | 8.07360903568864 | 807.360903568864 | | SD\_E3NeuriteWidthCh2 | 1.42070484581498 | 142.070484581498 | | SD\_NeuriteAvgLengthCh2 | 1.42070484581498 | 142.070484581498 | | SD\_NeuriteMaxLengthWithBranchesCh2 | 1.42070484581498 | 142.070484581498 | | SD\_NeuriteMaxLengthWithoutBranchesCh2 | 1.42070484581498 | 142.070484581498 | | SD\_NeuriteSpot%OverlapAreaCh3 | 1 | 100 | | SD\_NeuriteSpot%OverlapAreaCh4 | 1 | 100 | | SD\_NeuriteSpotAvgCountCh4 | 1 | 100 | | SD\_NeuriteSpotOverlapAreaCh3 | 2.01840225892216 | 201.840225892216 | | SD\_NeuriteSpotTotalAreaCh4 | 1 | 100 | | SD\_NeuriteSpotTotalCountCh4 | 1 | 100 | | SD\_TotalIntenCh3 | 8.07360903568864 | 807.360903568864 | | SD\_TotalIntenCh4 | 1 | 100 | | SD\_E3NeuriteMaxLengthWithoutBranchesCh2 | 1.42070484581498 | 142.070484581498 | | SD\_E3NeuriteSpotAvgCountCh4 | 1 | 100 | | SD\_E3NeuriteSpotOverlapAreaCh3 | 2.01840225892216 | 201.840225892216 | | SD\_E3NeuriteSpotOverlapAreaCh4 | 1 | 100 | | SD\_E3NeuriteSpotTotalAreaCh3 | 2.01840225892216 | 201.840225892216 | | SD\_E3NeuriteSpotTotalIntenCh3 | 8.07360903568864 | 807.360903568864 | | SD\_E3NeuriteSpotTotalIntenRatioCh4Ch2 | 1 | 100 | | SD\_E3NeuriteVarIntenCh2 | 4 | 400 | | SD\_E3TotalIntenCh4 | 1 | 100 | | SD\_NeuriteRamificationIndexCh2 | 1 | 100 | | SD\_NeuriteSpot%OverlapCountCh3 | 1 | 100 | | SD\_NeuriteSpot%OverlapCountCh4 | 1 | 100 | | SD\_NeuriteSpotAvgAreaCh4 | 1 | 100 | | SD\_NeuriteSpotAvgCountCh3 | 1 | 100 | | SD\_NeuriteSpotAvgIntenCh4 | 1 | 100 | | SD\_NeuriteSpotTotalIntenRatioCh3Ch2 | 1 | 100 | | SD\_NeuriteTotalAreaCh2 | 2.01840225892216 | 201.840225892216 | | SD\_NeuriteTotalCountCh2 | 1 | 100 | | SD\_NeuriteTotalIntenCh2 | 8.07360903568864 | 807.360903568864 | | NeuriteSpotTotalAreaPerWellCh3 | 2.01840225892216 | 201.840225892216 | | MEDIAN\_E1NeuriteDendriteMaxCh2 | 1 | 100 | | MEDIAN\_E1NeuriteMaxLengthWithoutBranchesCh2 | 1.42070484581498 | 142.070484581498 | | SD\_AvgIntenCh3 | 4 | 400 | | SD\_BranchPointTotalCountCh2 | 1 | 100 | | SD\_CellBodyAvgIntenCh2 | 4 | 400 | | SD\_CellBodyNucTotalIntenCh1 | 8.07360903568864 | 807.360903568864 | | SD\_CellBodyShapeLWRCh2 | 1 | 100 | | SD\_CellBodySpot%OverlapAreaCh4 | 1 | 100 | | SD\_CellBodySpot%OverlapCountCh4 | 1 | 100 | | SD\_CellBodySpotAvgIntenCh4 | 1 | 100 | | SD\_CellBodySpotTotalIntenCh3 | 8.07360903568864 | 807.360903568864 | | SD\_CellBodySpotTotalIntenRatioCh3Ch2 | 1 | 100 | | SD\_CellBodyVarIntenCh2 | 1 | 100 | | SD\_CrossPointAvgCountCh2 | 1 | 100 | | SD\_E1AvgIntenCh4 | 1 | 100 | | SD\_E1BranchPointTotalCountCh2 | 1 | 100 | | SD\_E1CellBodyNucCountCh2 | 1 | 100 | | SD\_E1CellBodyShapeP2ACh2 | 1 | 100 | | SD\_E1CellBodySpot%OverlapCountCh3 | 1 | 100 | | SD\_E1CellBodySpot%OverlapCountCh4 | 1 | 100 | | SD\_E1CellBodySpotAvgIntenCh4 | 1 | 100 | | SD\_E1CellBodySpotTotalAreaCh3 | 2.01840225892216 | 201.840225892216 | | SD\_E1CellBodySpotTotalAreaCh4 | 1 | 100 | | SD\_E1CrossPointAvgCountCh2 | 1 | 100 | | SD\_E1NeuriteAvgIntenCh2 | 4 | 400 | | SE\_E1NeuriteTotalLengthCh2 | 1.42070484581498 | 142.070484581498 | | SE\_E1TotalIntenCh3 | 8.07360903568864 | 807.360903568864 | | SE\_E2AvgIntenCh4 | 1 | 100 | | SE\_E2BranchPointCountPerNeuriteLengthCh2 | 0.703875968992248 | 70.3875968992248 | | SE\_E2CellBodyNucTotalAreaCh1 | 2.01840225892216 | 201.840225892216 | | SE\_E2CellBodyShapeP2ACh2 | 1 | 100 | | SE\_E2CellBodySpot%OverlapCountCh4 | 1 | 100 | | SE\_E2CellBodySpotAvgIntenCh3 | 4 | 400 | | SE\_E2CellBodySpotTotalAreaCh4 | 1 | 100 | | SE\_E2CellBodySpotTotalAreaRatioCh4Ch3 | 1 | 100 | | SE\_E2CellBodyVarIntenCh2 | 1 | 100 | | SE\_E2NeuriteAvgIntenCh2 | 4 | 400 | | SE\_E2NeuriteCriticalValueCh2 | 1.42070484581498 | 142.070484581498 | | SE\_E2NeuriteMaxLengthWithBranchesCh2 | 1.42070484581498 | 142.070484581498 | | SE\_E2NeuriteSpot%OverlapCountCh3 | 1 | 100 | | SE\_E2NeuriteSpotAvgCountCh3 | 1 | 100 | | SE\_E2NeuriteSpotOverlapCountCh4 | 1 | 100 | | SE\_E2NeuriteSpotTotalAreaCh4 | 1 | 100 | | SE\_E2NeuriteSpotTotalIntenRatioCh3Ch2 | 1 | 100 | | SE\_E2NeuriteSpotTotalIntenRatioCh4Ch3 | 1 | 100 | | SE\_E3AvgIntenCh4 | 1 | 100 | | SE\_E3BranchPointCountPerNeuriteLengthCh2 | 0.703875968992248 | 70.3875968992248 | | SE\_E3CellBodyNucTotalAreaCh1 | 2.01840225892216 | 201.840225892216 | | SE\_E3CellBodyShapeP2ACh2 | 1 | 100 | | SE\_E3CellBodySpotOverlapAreaCh3 | 2.01840225892216 | 201.840225892216 | | SE\_E3CellBodySpotOverlapCountCh3 | 1 | 100 | | SE\_E3CellBodySpotTotalAreaCh4 | 1 | 100 | | MEDIAN\_E2NeuriteSpotTotalCountCh4 | 1 | 100 | | MEDIAN\_E2NeuriteSpotTotalIntenCh3 | 8.07360903568864 | 807.360903568864 | | MEDIAN\_E2NeuriteSpotTotalIntenRatioCh3Ch2 | 1 | 100 | | MEDIAN\_E2NeuriteSpotTotalIntenRatioCh4Ch3 | 1 | 100 | | MEDIAN\_E2NeuriteTotalLengthCh2 | 1.42070484581498 | 142.070484581498 | | MEDIAN\_E2NeuriteWidthCh2 | 1.42070484581498 | 142.070484581498 | | MEDIAN\_E2TotalIntenCh3 | 8.07360903568864 | 807.360903568864 | | SD\_E1NeuriteSpotOverlapAreaCh3 | 2.01840225892216 | 201.840225892216 | | SD\_E1NeuriteSpotOverlapCountCh4 | 1 | 100 | | SD\_E1NeuriteSpotTotalAreaCh3 | 2.01840225892216 | 201.840225892216 | | SD\_E1NeuriteSpotTotalAreaCh4 | 1 | 100 | | SD\_E1NeuriteSpotTotalIntenCh3 | 8.07360903568864 | 807.360903568864 | | SD\_E1NeuriteSpotTotalIntenCh4 | 1 | 100 | | SD\_E1NeuriteTotalLengthCh2 | 1.42070484581498 | 142.070484581498 | | SD\_E1NeuriteWidthCh2 | 1.42070484581498 | 142.070484581498 | | SD\_E1TotalIntenCh3 | 8.07360903568864 | 807.360903568864 | | SD\_E2AvgIntenCh4 | 1 | 100 | | SD\_E2BranchPointCountPerNeuriteLengthCh2 | 0.703875968992248 | 70.3875968992248 | | SD\_E2BranchPointTotalCountCh2 | 1 | 100 | | SD\_E2CellBodyNucTotalAreaCh1 | 2.01840225892216 | 201.840225892216 | | SD\_E2CellBodyShapeP2ACh2 | 1 | 100 | | SD\_E2CellBodySpot%OverlapAreaCh3 | 1 | 100 | | SD\_E2CellBodySpotAvgIntenCh3 | 4 | 400 | | SD\_E2CellBodySpotTotalAreaCh4 | 1 | 100 | | SD\_E2CellBodySpotTotalAreaRatioCh4Ch3 | 1 | 100 | | SD\_E2NeuriteAvgIntenCh2 | 4 | 400 | | SD\_E2NeuriteMaxLengthWithBranchesCh2 | 1.42070484581498 | 142.070484581498 | | SD\_E2NeuriteSpotAvgCountCh3 | 1 | 100 | | SD\_E2NeuriteSpotOverlapCountCh4 | 1 | 100 | | SD\_E2NeuriteSpotTotalAreaCh4 | 1 | 100 | | SD\_E2NeuriteSpotTotalCountCh4 | 1 | 100 | | SD\_E2NeuriteSpotTotalIntenRatioCh3Ch2 | 1 | 100 | | SD\_E2NeuriteSpotTotalIntenRatioCh4Ch3 | 1 | 100 | | SD\_E2NeuriteTotalLengthCh2 | 1.42070484581498 | 142.070484581498 | | SD\_E2TotalIntenCh3 | 8.07360903568864 | 807.360903568864 | | SD\_E3AvgIntenCh4 | 1 | 100 | | SD\_E3BranchPointCountPerNeuriteLengthCh2 | 0.703875968992248 | 70.3875968992248 | | SE\_E3CellBodySpotTotalAreaRatioCh4Ch3 | 1 | 100 | | SE\_E3CellBodySpotTotalCountCh3 | 1 | 100 | | SE\_E3CellBodyTotalIntenCh2 | 8.07360903568864 | 807.360903568864 | | SE\_E3CrossPointTotalCountCh2 | 1 | 100 | | SE\_E3NeuriteCriticalValueCh2 | 1.42070484581498 | 142.070484581498 | | SE\_E3NeuriteMaxLengthWithBranchesCh2 | 1.42070484581498 | 142.070484581498 | | SE\_E3NeuriteSpot%OverlapAreaCh3 | 1 | 100 | | SE\_E3NeuriteSpot%OverlapAreaCh4 | 1 | 100 | | SE\_E3NeuriteSpot%OverlapCountCh3 | 1 | 100 | | SE\_E3NeuriteSpotAvgAreaCh3 | 2.01840225892216 | 201.840225892216 | | SE\_E3NeuriteSpotTotalAreaCh4 | 1 | 100 | | SE\_E3NeuriteSpotTotalCountCh4 | 1 | 100 | | SE\_E3NeuriteSpotTotalIntenRatioCh4Ch3 | 1 | 100 | | SE\_E3NeuriteTotalAreaCh2 | 2.01840225892216 | 201.840225892216 | | SE\_NeuriteAvgIntenCh2 | 4 | 400 | | SE\_NeuriteSpotOverlapAreaCh3 | 2.01840225892216 | 201.840225892216 | | SE\_NeuriteSpotOverlapAreaCh4 | 1 | 100 | | SE\_NeuriteSpotOverlapCountCh3 | 1 | 100 | | SE\_NeuriteSpotTotalAreaCh3 | 2.01840225892216 | 201.840225892216 | | SE\_NeuriteSpotTotalCountCh3 | 1 | 100 | | SE\_NeuriteSpotTotalIntenCh4 | 1 | 100 | | SE\_NeuriteSpotTotalIntenRatioCh4Ch2 | 1 | 100 | | SE\_NeuriteTotalLengthCh2 | 1.42070484581498 | 142.070484581498 | | SE\_NeuriteWidthCh2 | 1.42070484581498 | 142.070484581498 | | SelectedNeuronCountPerValidField | 2.30351211803678 | 230.351211803678 | | ValidFieldCount | 1 | 100 | | NeuriteSpotTotalCountPerFieldCh4 | 1 | 100 | | NeuriteSpotTotalCountPerWellCh4 | 1 | 100 | | NeuriteSpotTotalIntenPerNeuriteLengthCh4 | 1 | 100 | | SE\_BranchPointTotalCountCh2 | 1 | 100 | | SE\_CellBodyAvgIntenCh2 | 4 | 400 | | SE\_CellBodyNucCountCh2 | 1 | 100 | | SE\_CellBodyNucTotalIntenCh1 | 8.07360903568864 | 807.360903568864 | | SE\_CellBodySpot%OverlapAreaCh4 | 1 | 100 | | SE\_CellBodySpotAvgIntenCh4 | 1 | 100 | | SE\_CellBodySpotTotalIntenRatioCh3Ch2 | 1 | 100 | | SE\_CellBodySpotTotalIntenRatioCh4Ch3 | 1 | 100 | | SE\_CellBodyVarIntenCh2 | 1 | 100 | | SE\_CrossPointAvgCountCh2 | 1 | 100 | | SE\_E1BranchPointCountPerNeuriteLengthCh2 | 0.703875968992248 | 70.3875968992248 | | SE\_E1BranchPointTotalCountCh2 | 1 | 100 | | SE\_E1CellBodyNucCountCh2 | 1 | 100 | | SE\_E1CellBodyShapeP2ACh2 | 1 | 100 | | SE\_E1CellBodySpot%OverlapAreaCh3 | 1 | 100 | | SE\_E1CellBodySpot%OverlapCountCh3 | 1 | 100 | | SE\_E1CellBodySpot%OverlapCountCh4 | 1 | 100 | | SE\_E1CellBodySpotAvgIntenCh4 | 1 | 100 | | SE\_E1CellBodySpotTotalAreaCh3 | 2.01840225892216 | 201.840225892216 | | SE\_E1CellBodySpotTotalAreaCh4 | 1 | 100 | | SE\_E1NeuriteAvgIntenCh2 | 4 | 400 | | SE\_E1NeuriteMaxLengthWithBranchesCh2 | 1.42070484581498 | 142.070484581498 | | SE\_E1NeuriteSpotOverlapAreaCh3 | 2.01840225892216 | 201.840225892216 | | SE\_E1NeuriteSpotOverlapAreaCh4 | 1 | 100 | | SE\_E1NeuriteSpotOverlapCountCh4 | 1 | 100 | | SE\_E1NeuriteSpotTotalIntenCh3 | 8.07360903568864 | 807.360903568864 | | SE\_E1NeuriteSpotTotalIntenCh4 | 1 | 100 | | MEDIAN\_NeuriteSpotTotalAreaCh4 | 1 | 100 | | MEDIAN\_NeuriteSpotTotalCountCh3 | 1 | 100 | | SD\_E3CellBodyNucTotalAreaCh1 | 2.01840225892216 | 201.840225892216 | | SD\_E3CellBodyShapeP2ACh2 | 1 | 100 | | SD\_E3CellBodySpotOverlapCountCh3 | 1 | 100 | | SD\_E3CellBodySpotTotalAreaRatioCh4Ch3 | 1 | 100 | | SD\_E3CellBodySpotTotalCountCh3 | 1 | 100 | | SD\_E3CellBodySpotTotalIntenRatioCh4Ch2 | 1 | 100 | | SD\_E3CellBodyTotalIntenCh2 | 8.07360903568864 | 807.360903568864 | | SD\_E3NeuriteAvgIntenCh2 | 4 | 400 | | SD\_E3NeuriteCriticalValueCh2 | 1.42070484581498 | 142.070484581498 | | SD\_E3NeuriteMaxLengthWithBranchesCh2 | 1.42070484581498 | 142.070484581498 | | SD\_E3NeuriteSpot%OverlapAreaCh3 | 1 | 100 | | SD\_E3NeuriteSpot%OverlapAreaCh4 | 1 | 100 | | SD\_E3NeuriteSpot%OverlapCountCh3 | 1 | 100 | | SD\_E3NeuriteSpotAvgAreaCh3 | 2.01840225892216 | 201.840225892216 | | SD\_E3NeuriteSpotAvgCountCh3 | 1 | 100 | | SD\_E3NeuriteSpotAvgIntenCh4 | 1 | 100 | | SD\_E3NeuriteSpotTotalCountCh4 | 1 | 100 | | SD\_E3NeuriteSpotTotalIntenRatioCh4Ch3 | 1 | 100 | | SD\_NeuriteDendriteMaxCh2 | 1 | 100 | | SD\_NeuriteSpotOverlapAreaCh4 | 1 | 100 | | SD\_NeuriteSpotOverlapCountCh3 | 1 | 100 | | SD\_NeuriteSpotOverlapCountCh4 | 1 | 100 | | SD\_NeuriteSpotTotalAreaCh3 | 2.01840225892216 | 201.840225892216 | | SD\_NeuriteSpotTotalCountCh3 | 1 | 100 | | SD\_NeuriteSpotTotalIntenCh4 | 1 | 100 | | SD\_NeuriteTotalLengthCh2 | 1.42070484581498 | 142.070484581498 | | SD\_NeuriteWidthCh2 | 1.42070484581498 | 142.070484581498 | |  |  |  | | --- | --- | --- | | \* Indicates the default well feature for the Assay Protocol   \*\* Indicates feature extents are dependent upon system reference well settings | | |     ---  |  |  |  |  |  |  |  |  |  |  |  |  |  |  |  |  |  |  |  |  |  |  |  |  |  |  |  |  |  |  |  |  |  |  |  |  |  |  |  |  |  |  |  |  |  |  |  |  |  |  |  |  |  |  |  |  |  |  |  |  |  |  |  |  |  |  |  |  |  |  |  |  |  |  |  |  |  |  |  |  |  |  |  |  |  |  |  |  |  |  |  |  |  |  |  |  |  |  |  |  |  |  |  |  |  |  |  |  |  |  |  |  |  |  |  |  |  |  |  |  |  |  |  |  |  |  |  |  |  |  |  |  |  |  |  |  |  |  |  |  |  |  |  |  |  |  |  |  |  |  |  |  |  |  |  |  |  |  |  |  |  |  |  |  |  |  |  |  |  |  |  |  |  |  |  |  |  |  |  |  |  |  |  |  |  |  |  |  |  |  |  |  |  |  |  |  |  |  |  |  |  |  |  |  |  |  |  |  |  |  |  |  |  |  |  |  |  |  |  |  |  |  |  |  |  |  |  |  |  |  |  |  |  |  |  |  |  |  |  |  |  |  |  |  |  |  |  |  |  |  |  |  |  |  |  |  |  |  |  |  |  |  |  |  |  |  |  |  |  |  |  |  |  |  |  |  |  |  |  |  |  |  |  |  |  |  |  |  |  |  |  |  |  |  |  |  |  |  |  |  |  |  |  |  |  |  |  |  |  |  |  |  |  |  |  |  |  |  |  |  |  |  |  |  |  |  |  |  |  |  |  |  |  |  |  |  |  |  |  |  |  |  |  |  |  |  |  |  |  |  |  |  |  |  |  |  |  |  |  |  |  |  |  |  |  |  |  |  |  |  |  |  |  |  |  |  |  |  |  |  |  |  |  |  |  |  |  |  |  |  |  |  |  |  |  |  |  |  |  |  |  |  |  |  |  |  |  |  |  |  |  |  |  |  |  |  |  |  |  |  |  |  |  |  |  |  |  |  |  |  |  |  |  |  |  |  |  |  |  |  |  |  |  |  |  |  |  |  |  |  |  |  |  |  |  |  |  |  |  |  |  |  |  |  |  |  |  |  |  |  |  |  |  |  |  |  |  |  |  |  |  |  |  |  |  |  |  |  |  |  |  |  |  |  |  |  |  |  |  |  |  |  |  |  |  |  |  |  |  |  |  |  |  |  |  |  |  |  |  |  |  |  |  |  |  |  |  |  |  |  |  |  |  |  |  |  |  |  |  |  |  |  |  |  |  |  |  |  |  |  |  |  |  |  |  |  |  |  |  |  |  |  |  |  |  |  |  |  |  |  |  |  |  |  |  |  |  |  |  |  |  |  |  |  |  |  |  |  |  |  |  |  |  |  |  |  |  |  |  |  |  |  |  |  |  |  |  |  |  |  |  |  |  |  |  |  |  |  |  |  |  |  |  |  |  |  |  |  |  |  |  |  |  |  |  |  |  |  |  |  |  |  |  |  |  |  |  |  |  |  |  |  |  |  |  |  |  |  |  |  |  |  |  |  |  |  |  |  |  |  |  |  |  |  |  |  |  |  |  |  |  |  |  |  |  |  |  |  |  |  |  |  |  |  |  |  |  |  |  |  |  |  |  |  |  |  |  |  |  |  |  |  |  |  |  |  |  |  |  |  |  |  |  |  |  |  |  |  |  |  |  |  |  |  |  |  |  |  |  |  |  |  |  |  |  |  |  |  |  |  |  |  |  |  |  |  |  |  |  |  |  |  |  |  |  |  |  |  |  |  |  |  |  |  |  |  |  |  |  |  |  |  |  |  |  |  |  |  |  |  |  |  |  |  |  |  |  |  |  |  |  |  |  |  |  |  |  |  |  |  |  |  |  |  |  |  |  |  |  |  |  |  |  |  |  |  |  |  |  |  |  |  |  |  |  |  |  |  |  |  |  |  |  |  |  |  |  |  |  |  |  |  |  |  |  |  |  |  |  |  |  |  |  |  |  |  |  |  |  |  |  |  |  |  |  |  |  |  |  |  |  |  |  |  |  |  |  |  |  |  |  |  |  |  |  |  |  |  |  |  |  |  |  |  |  |  |  |  |  |  |  |  |  |  |  |  |  |  |  |  |  |  |  |  |  |  |  |  |  |  |  |  |  |  |  |  |  |  |  |  |  |  |  |  |  |  |  |  |  |  |  |  |  |  |  |  |  |  |  |  |  |  |  |  |  |  |  |  |  |  |  |  |  |  |  |  |  |  |  |  |  |  |  |  |  |  |  |  |  |  |  |  |  |  |  |  |  |  |  |  |  |  |  |  |  |  |  |  |  |  |  |  |  |  |  |  |  |  |  |  |  |  |  |  |  |  |  |  |  |  |  |  |  |  |  |  |  |  |  |  |  |  |  |  |  |  |  |  |  |  |  |  |  |  |  |  |  |  |  |  |  |  |  |  |  |  |  |  |  |  |  |  |  |  |  |  |  |  |  |  |  |  |  |  |  |  |  |  |  |  |  |  |  |  |  |  |  |  |  |  |  |  |  |  |  |  |  |  |  |  |  |  |  |  |  |  |  |  |  |  |  |  |  |  |  |  |  |  |  |  |  |  |  |  |  |  |  |  |  |  |  |  |  |  |  |  |  |  |  |  |  |  |  |  |  |  |  |  |  |  |  |  |  |  |  |  |  |  |  |  |  |  |  |  |  |  |  |  |  |  |  |  |  |  |  |  |  |  |  |  |  |  |  |  |  |  |  |  |  |  |  |  |  |  |  |  |  |  |  |  |  |  |  |  |  |  |  |  |  |  |  |  |  |  |  |  |  |  |  |  |  |  |  |  |  |  |  |  |  |  |  |  |  |  |  |  |  |  |  |  |  |  |  |  |  |  |  |  |  |  |  |  |  |  |  |  |  |  |  |  |  |  |  |  |  |  |  |  |  |  |  |  |  |  |  |  |  |  |  |  |  |  |  |  |  |  |  |  |  |  |  |  |  |  |  |  |  |  |  |  |  |  |  |  |  |  |  |  |  |  |  |  |  |  |  |  |  |  |  |  |  |  |  |  |  |  |  |  |  |  |  |  |  |  |  |  |  |  |  |  |  |  |  |  |  |  |  |  |  |  |  |  |  |  |  |  |  |  |  |  |  |  |  |  |  |  |  |  |  |  |  |  |  |  |  |  |  |  |  |  |  |  |  |  |  |  |  |  |  |  |  |  |  |  |  |  |  |  |  |  |  |  |  |  |  |  |  |  |  |  |  |  |  |  |  |  |  |  |  |  |  |  |  |  |  |  |  |  |  |  |  |  |  |  |  |  |  |  |  |  |  |  |  |  |  |  |  |  |  |  |  |  |  |  |  |  |  |  |  |  |  |  |  |  |  |  |  |  |  |  |  |  |  |  |  |  |  |  |  |  |  |  |  |  |  |  |  |  |  |  |  |  |  |  |  |  |  |  |  |  |  |  |  |  |  |  |  |  |  |  |  |  |  |  |  |  |  |  |  |  |  |  |  |  |  |  |  |  |  |  |  |  |  |  |  |  |  |  |  |  |  |  |  |  |  |  |  |  |  |  |  |  |  |  |  |  |  |  |  |  |  |  |  |  |  |  |  |  |  |  |  |  |  |  |  |  |  |  |  |  |  |  |  |  |  |  |  |  |  |  |  |  |  |  |  |  |  |  |  |  |  |  |  |  |  |  |  |  |  |  |  |  |  |  |  |  |  |  |  |  |  |  |  |  |  |  |  |  |  |  |  |  |  |  |  |  |  |  |  |  |  |  |  |  |  |  |  |  |  |  |  |  |  |  |  |  |  |  |  |  |  | | --- | --- | --- | --- | --- | --- | --- | --- | --- | --- | --- | --- | --- | --- | --- | --- | --- | --- | --- | --- | --- | --- | --- | --- | --- | --- | --- | --- | --- | --- | --- | --- | --- | --- | --- | --- | --- | --- | --- | --- | --- | --- | --- | --- | --- | --- | --- | --- | --- | --- | --- | --- | --- | --- | --- | --- | --- | --- | --- | --- | --- | --- | --- | --- | --- | --- | --- | --- | --- | --- | --- | --- | --- | --- | --- | --- | --- | --- | --- | --- | --- | --- | --- | --- | --- | --- | --- | --- | --- | --- | --- | --- | --- | --- | --- | --- | --- | --- | --- | --- | --- | --- | --- | --- | --- | --- | --- | --- | --- | --- | --- | --- | --- | --- | --- | --- | --- | --- | --- | --- | --- | --- | --- | --- | --- | --- | --- | --- | --- | --- | --- | --- | --- | --- | --- | --- | --- | --- | --- | --- | --- | --- | --- | --- | --- | --- | --- | --- | --- | --- | --- | --- | --- | --- | --- | --- | --- | --- | --- | --- | --- | --- | --- | --- | --- | --- | --- | --- | --- | --- | --- | --- | --- | --- | --- | --- | --- | --- | --- | --- | --- | --- | --- | --- | --- | --- | --- | --- | --- | --- | --- | --- | --- | --- | --- | --- | --- | --- | --- | --- | --- | --- | --- | --- | --- | --- | --- | --- | --- | --- | --- | --- | --- | --- | --- | --- | --- | --- | --- | --- | --- | --- | --- | --- | --- | --- | --- | --- | --- | --- | --- | --- | --- | --- | --- | --- | --- | --- | --- | --- | --- | --- | --- | --- | --- | --- | --- | --- | --- | --- | --- | --- | --- | --- | --- | --- | --- | --- | --- | --- | --- | --- | --- | --- | --- | --- | --- | --- | --- | --- | --- | --- | --- | --- | --- | --- | --- | --- | --- | --- | --- | --- | --- | --- | --- | --- | --- | --- | --- | --- | --- | --- | --- | --- | --- | --- | --- | --- | --- | --- | --- | --- | --- | --- | --- | --- | --- | --- | --- | --- | --- | --- | --- | --- | --- | --- | --- | --- | --- | --- | --- | --- | --- | --- | --- | --- | --- | --- | --- | --- | --- | --- | --- | --- | --- | --- | --- | --- | --- | --- | --- | --- | --- | --- | --- | --- | --- | --- | --- | --- | --- | --- | --- | --- | --- | --- | --- | --- | --- | --- | --- | --- | --- | --- | --- | --- | --- | --- | --- | --- | --- | --- | --- | --- | --- | --- | --- | --- | --- | --- | --- | --- | --- | --- | --- | --- | --- | --- | --- | --- | --- | --- | --- | --- | --- | --- | --- | --- | --- | --- | --- | --- | --- | --- | --- | --- | --- | --- | --- | --- | --- | --- | --- | --- | --- | --- | --- | --- | --- | --- | --- | --- | --- | --- | --- | --- | --- | --- | --- | --- | --- | --- | --- | --- | --- | --- | --- | --- | --- | --- | --- | --- | --- | --- | --- | --- | --- | --- | --- | --- | --- | --- | --- | --- | --- | --- | --- | --- | --- | --- | --- | --- | --- | --- | --- | --- | --- | --- | --- | --- | --- | --- | --- | --- | --- | --- | --- | --- | --- | --- | --- | --- | --- | --- | --- | --- | --- | --- | --- | --- | --- | --- | --- | --- | --- | --- | --- | --- | --- | --- | --- | --- | --- | --- | --- | --- | --- | --- | --- | --- | --- | --- | --- | --- | --- | --- | --- | --- | --- | --- | --- | --- | --- | --- | --- | --- | --- | --- | --- | --- | --- | --- | --- | --- | --- | --- | --- | --- | --- | --- | --- | --- | --- | --- | --- | --- | --- | --- | --- | --- | --- | --- | --- | --- | --- | --- | --- | --- | --- | --- | --- | --- | --- | --- | --- | --- | --- | --- | --- | --- | --- | --- | --- | --- | --- | --- | --- | --- | --- | --- | --- | --- | --- | --- | --- | --- | --- | --- | --- | --- | --- | --- | --- | --- | --- | --- | --- | --- | --- | --- | --- | --- | --- | --- | --- | --- | --- | --- | --- | --- | --- | --- | --- | --- | --- | --- | --- | --- | --- | --- | --- | --- | --- | --- | --- | --- | --- | --- | --- | --- | --- | --- | --- | --- | --- | --- | --- | --- | --- | --- | --- | --- | --- | --- | --- | --- | --- | --- | --- | --- | --- | --- | --- | --- | --- | --- | --- | --- | --- | --- | --- | --- | --- | --- | --- | --- | --- | --- | --- | --- | --- | --- | --- | --- | --- | --- | --- | --- | --- | --- | --- | --- | --- | --- | --- | --- | --- | --- | --- | --- | --- | --- | --- | --- | --- | --- | --- | --- | --- | --- | --- | --- | --- | --- | --- | --- | --- | --- | --- | --- | --- | --- | --- | --- | --- | --- | --- | --- | --- | --- | --- | --- | --- | --- | --- | --- | --- | --- | --- | --- | --- | --- | --- | --- | --- | --- | --- | --- | --- | --- | --- | --- | --- | --- | --- | --- | --- | --- | --- | --- | --- | --- | --- | --- | --- | --- | --- | --- | --- | --- | --- | --- | --- | --- | --- | --- | --- | --- | --- | --- | --- | --- | --- | --- | --- | --- | --- | --- | --- | --- | --- | --- | --- | --- | --- | --- | --- | --- | --- | --- | --- | --- | --- | --- | --- | --- | --- | --- | --- | --- | --- | --- | --- | --- | --- | --- | --- | --- | --- | --- | --- | --- | --- | --- | --- | --- | --- | --- | --- | --- | --- | --- | --- | --- | --- | --- | --- | --- | --- | --- | --- | --- | --- | --- | --- | --- | --- | --- | --- | --- | --- | --- | --- | --- | --- | --- | --- | --- | --- | --- | --- | --- | --- | --- | --- | --- | --- | --- | --- | --- | --- | --- | --- | --- | --- | --- | --- | --- | --- | --- | --- | --- | --- | --- | --- | --- | --- | --- | --- | --- | --- | --- | --- | --- | --- | --- | --- | --- | --- | --- | --- | --- | --- | --- | --- | --- | --- | --- | --- | --- | --- | --- | --- | --- | --- | --- | --- | --- | --- | --- | --- | --- | --- | --- | --- | --- | --- | --- | --- | --- | --- | --- | --- | --- | --- | --- | --- | --- | --- | --- | --- | --- | --- | --- | --- | --- | --- | --- | --- | --- | --- | --- | --- | --- | --- | --- | --- | --- | --- | --- | --- | --- | --- | --- | --- | --- | --- | --- | --- | --- | --- | --- | --- | --- | --- | --- | --- | --- | --- | --- | --- | --- | --- | --- | --- | --- | --- | --- | --- | --- | --- | --- | --- | --- | --- | --- | --- | --- | --- | --- | --- | --- | --- | --- | --- | --- | --- | --- | --- | --- | --- | --- | --- | --- | --- | --- | --- | --- | --- | --- | --- | --- | --- | --- | --- | --- | --- | --- | --- | --- | --- | --- | --- | --- | --- | --- | --- | --- | --- | --- | --- | --- | --- | --- | --- | --- | --- | --- | --- | --- | --- | --- | --- | --- | --- | --- | --- | --- | --- | --- | --- | --- | --- | --- | --- | --- | --- | --- | --- | --- | --- | --- | --- | --- | --- | --- | --- | --- | --- | --- | --- | --- | --- | --- | --- | --- | --- | --- | --- | --- | --- | --- | --- | --- | --- | --- | --- | --- | --- | --- | --- | --- | --- | --- | --- | --- | --- | --- | --- | --- | --- | --- | --- | --- | --- | --- | --- | --- | --- | --- | --- | --- | --- | --- | --- | --- | --- | --- | --- | --- | --- | --- | --- | --- | --- | --- | --- | --- | --- | --- | --- | --- | --- | --- | --- | --- | --- | --- | --- | --- | --- | --- | --- | --- | --- | --- | --- | --- | --- | --- | --- | --- | --- | --- | --- | --- | --- | --- | --- | --- | --- | --- | --- | --- | --- | --- | --- | --- | --- | --- | --- | --- | --- | --- | --- | --- | --- | --- | --- | --- | --- | --- | --- | --- | --- | --- | --- | --- | --- | --- | --- | --- | --- | --- | --- | --- | --- | --- | --- | --- | --- | --- | --- | --- | --- | --- | --- | --- | --- | --- | --- | --- | --- | --- | --- | --- | --- | --- | --- | --- | --- | --- | --- | --- | --- | --- | --- | --- | --- | --- | --- | --- | --- | --- | --- | --- | --- | --- | --- | --- | --- | --- | --- | --- | --- | --- | --- | --- | --- | --- | --- | --- | --- | --- | --- | --- | --- | --- | --- | --- | --- | --- | --- | --- | --- | --- | --- | --- | --- | --- | --- | --- | --- | --- | --- | --- | --- | --- | --- | --- | --- | --- | --- | --- | --- | --- | --- | --- | --- | --- | --- | --- | --- | --- | --- | --- | --- | --- | --- | --- | --- | --- | --- | --- | --- | --- | --- | --- | --- | --- | --- | --- | --- | --- | --- | --- | --- | --- | --- | --- | --- | --- | --- | --- | --- | --- | --- | --- | --- | --- | --- | --- | --- | --- | --- | --- | --- | --- | --- | --- | --- | --- | --- | --- | --- | --- | --- | --- | --- | --- | --- | --- | --- | --- | --- | --- | --- | --- | --- | --- | --- | --- | --- | --- | --- | --- | --- | --- | --- | --- | --- | --- | --- | --- | --- | --- | --- | --- | --- | --- | --- | --- | --- | --- | --- | --- | --- | --- | --- | --- | --- | --- | --- | --- | --- | --- | --- | --- | --- | --- | --- | --- | --- | --- | --- | --- | --- | --- | --- | --- | --- | --- | --- | --- | --- | --- | --- | --- | --- | --- | --- | --- | --- | --- | --- | --- | --- | --- | --- | --- | --- | --- | --- | --- | --- | --- | --- | --- | --- | --- | --- | --- | --- | --- | --- | --- | --- | --- | --- | --- | --- | --- | --- | --- | --- | --- | --- | --- | --- | --- | --- | --- | --- | --- | --- | --- | --- | --- | --- | --- | --- | --- | --- | --- | --- | --- | --- | --- | --- | --- | --- | --- | --- | --- | --- | --- | --- | --- | --- | --- | --- | --- | --- | --- | --- | --- | --- | --- | --- | --- | --- | --- | --- | --- | --- | --- | --- | --- | --- | --- | --- | --- | --- | --- | --- | --- | --- | --- | --- | --- | --- | --- | --- | --- | --- | --- | --- | --- | --- | --- | --- | --- | --- | --- | --- | --- | --- | --- | --- | --- | --- | --- | --- | --- | --- | --- | --- | --- | --- | --- | --- | --- | --- | --- | --- | --- | --- | --- | --- | --- | --- | --- | --- | --- | --- | --- | --- | --- | --- | --- | --- | --- | --- | --- | --- | --- | --- | --- | --- | --- | --- | --- | --- | --- | --- | --- | --- | --- | --- | --- | --- | --- | --- | --- | --- | --- | --- | --- | --- | --- | --- | --- | --- | --- | --- | --- | --- | --- | --- | --- | --- | --- | --- | --- | --- | --- | --- | --- | --- | --- | --- | --- | --- | --- | --- | --- | --- | --- | --- | --- | --- | --- | --- | --- | --- | --- | --- | --- | --- | --- | --- | --- | --- | --- | --- | --- | --- | --- | --- | --- | --- | --- | --- | --- | --- | --- | --- | --- | --- | --- | --- | --- | --- | --- | --- | --- | --- | --- | --- | --- | --- | | | Selected Well Features to Store | | --- | | NeuronalProfilingV42Well:%EventType1Neurons | | NeuronalProfilingV42Well:%EventType2Neurons | | NeuronalProfilingV42Well:%EventType3Neurons | | NeuronalProfilingV42Well:%HIGH\_AvgIntenCh3 | | NeuronalProfilingV42Well:%HIGH\_BranchPointAvgCountCh2 | | NeuronalProfilingV42Well:%HIGH\_BranchPointAvgDistFromCellBodyCh2 | | NeuronalProfilingV42Well:%HIGH\_BranchPointCountPerNeuriteLengthCh2 | | NeuronalProfilingV42Well:%HIGH\_BranchPointTotalCountCh2 | | NeuronalProfilingV42Well:%HIGH\_CellBodyAreaCh2 | | NeuronalProfilingV42Well:%HIGH\_CellBodyAvgIntenCh2 | | NeuronalProfilingV42Well:%HIGH\_CellBodyNucAvgIntenCh1 | | NeuronalProfilingV42Well:%HIGH\_CellBodyNucCountCh2 | | NeuronalProfilingV42Well:%HIGH\_CellBodyNucTotalAreaCh1 | | NeuronalProfilingV42Well:%HIGH\_CellBodyNucTotalIntenCh1 | | NeuronalProfilingV42Well:%HIGH\_CellBodyShapeLWRCh2 | | NeuronalProfilingV42Well:%HIGH\_CellBodyShapeP2ACh2 | | NeuronalProfilingV42Well:%HIGH\_CellBodySpot%OverlapAreaCh3 | | NeuronalProfilingV42Well:%HIGH\_CellBodySpot%OverlapCountCh3 | | NeuronalProfilingV42Well:%HIGH\_CellBodySpotAvgIntenCh3 | | NeuronalProfilingV42Well:%HIGH\_CellBodySpotOverlapAreaCh3 | | NeuronalProfilingV42Well:%HIGH\_CellBodySpotOverlapCountCh3 | | NeuronalProfilingV42Well:%HIGH\_CellBodySpotTotalAreaCh3 | | NeuronalProfilingV42Well:%HIGH\_CellBodySpotTotalCountCh3 | | NeuronalProfilingV42Well:%HIGH\_CellBodySpotTotalIntenCh3 | | NeuronalProfilingV42Well:%HIGH\_CellBodySpotTotalIntenRatioCh3Ch2 | | NeuronalProfilingV42Well:%HIGH\_CellBodyTotalIntenCh2 | | NeuronalProfilingV42Well:%HIGH\_CrossPointAvgCountCh2 | | NeuronalProfilingV42Well:%HIGH\_CrossPointTotalCountCh2 | | NeuronalProfilingV42Well:%HIGH\_E1AvgIntenCh3 | | NeuronalProfilingV42Well:%HIGH\_E1BranchPointAvgCountCh2 | | NeuronalProfilingV42Well:%HIGH\_E1BranchPointAvgDistFromCellBodyCh2 | | NeuronalProfilingV42Well:%HIGH\_E1BranchPointCountPerNeuriteLengthCh2 | | NeuronalProfilingV42Well:%HIGH\_E1BranchPointTotalCountCh2 | | NeuronalProfilingV42Well:%HIGH\_E1CellBodyAreaCh2 | | NeuronalProfilingV42Well:%HIGH\_E1CellBodyAvgIntenCh2 | | NeuronalProfilingV42Well:%HIGH\_E1CellBodyNucAvgIntenCh1 | | NeuronalProfilingV42Well:%HIGH\_E1CellBodyNucCountCh2 | | NeuronalProfilingV42Well:%HIGH\_E1CellBodyNucTotalAreaCh1 | | NeuronalProfilingV42Well:%HIGH\_E1CellBodyNucTotalIntenCh1 | | NeuronalProfilingV42Well:%HIGH\_E1CellBodyShapeLWRCh2 | | NeuronalProfilingV42Well:%HIGH\_E1CellBodyShapeP2ACh2 | | NeuronalProfilingV42Well:%HIGH\_E1CellBodySpot%OverlapAreaCh3 | | NeuronalProfilingV42Well:%HIGH\_E1CellBodySpot%OverlapCountCh3 | | NeuronalProfilingV42Well:%HIGH\_E1CellBodySpotAvgIntenCh3 | | NeuronalProfilingV42Well:%HIGH\_E1CellBodySpotOverlapAreaCh3 | | NeuronalProfilingV42Well:%HIGH\_E1CellBodySpotOverlapCountCh3 | | NeuronalProfilingV42Well:%HIGH\_E1CellBodySpotTotalAreaCh3 | | NeuronalProfilingV42Well:%HIGH\_E1CellBodySpotTotalCountCh3 | | NeuronalProfilingV42Well:%HIGH\_E1CellBodySpotTotalIntenCh3 | | NeuronalProfilingV42Well:%HIGH\_E1CellBodySpotTotalIntenRatioCh3Ch2 | | NeuronalProfilingV42Well:%HIGH\_E1CellBodyTotalIntenCh2 | | NeuronalProfilingV42Well:%HIGH\_E1CrossPointAvgCountCh2 | | NeuronalProfilingV42Well:%HIGH\_E1CrossPointTotalCountCh2 | | NeuronalProfilingV42Well:%HIGH\_E1NeuriteAvgIntenCh2 | | NeuronalProfilingV42Well:%HIGH\_E1NeuriteAvgLengthCh2 | | NeuronalProfilingV42Well:%HIGH\_E1NeuriteCriticalValueCh2 | | NeuronalProfilingV42Well:%HIGH\_E1NeuriteDendriteMaxCh2 | | NeuronalProfilingV42Well:%HIGH\_E1NeuriteMaxLengthWithBranchesCh2 | | NeuronalProfilingV42Well:%HIGH\_E1NeuriteMaxLengthWithoutBranchesCh2 | | NeuronalProfilingV42Well:%HIGH\_E1NeuriteRamificationIndexCh2 | | NeuronalProfilingV42Well:%HIGH\_E1NeuriteSpot%OverlapAreaCh3 | | NeuronalProfilingV42Well:%HIGH\_E1NeuriteSpot%OverlapCountCh3 | | NeuronalProfilingV42Well:%HIGH\_E1NeuriteSpotAvgAreaCh3 | | NeuronalProfilingV42Well:%HIGH\_E1NeuriteSpotAvgCountCh3 | | NeuronalProfilingV42Well:%HIGH\_E1NeuriteSpotAvgIntenCh3 | | NeuronalProfilingV42Well:%HIGH\_E1NeuriteSpotOverlapAreaCh3 | | NeuronalProfilingV42Well:%HIGH\_E1NeuriteSpotOverlapCountCh3 | | NeuronalProfilingV42Well:%HIGH\_E1NeuriteSpotTotalAreaCh3 | | NeuronalProfilingV42Well:%HIGH\_E1NeuriteSpotTotalCountCh3 | | NeuronalProfilingV42Well:%HIGH\_E1NeuriteSpotTotalIntenCh3 | | NeuronalProfilingV42Well:%HIGH\_E1NeuriteSpotTotalIntenRatioCh3Ch2 | | NeuronalProfilingV42Well:%HIGH\_E1NeuriteTotalAreaCh2 | | NeuronalProfilingV42Well:%HIGH\_E1NeuriteTotalCountCh2 | | NeuronalProfilingV42Well:%HIGH\_E1NeuriteTotalIntenCh2 | | NeuronalProfilingV42Well:%HIGH\_E1NeuriteTotalLengthCh2 | | NeuronalProfilingV42Well:%HIGH\_E1NeuriteVarIntenCh2 | | NeuronalProfilingV42Well:%HIGH\_E1NeuriteWidthCh2 | | NeuronalProfilingV42Well:%HIGH\_E1TotalIntenCh3 | | NeuronalProfilingV42Well:%HIGH\_E2AvgIntenCh3 | | NeuronalProfilingV42Well:%HIGH\_E2BranchPointAvgCountCh2 | | NeuronalProfilingV42Well:%HIGH\_E2BranchPointAvgDistFromCellBodyCh2 | | NeuronalProfilingV42Well:%HIGH\_E2BranchPointCountPerNeuriteLengthCh2 | | NeuronalProfilingV42Well:%HIGH\_E2BranchPointTotalCountCh2 | | NeuronalProfilingV42Well:%HIGH\_E2CellBodyAreaCh2 | | NeuronalProfilingV42Well:%HIGH\_E2CellBodyAvgIntenCh2 | | NeuronalProfilingV42Well:%HIGH\_E2CellBodyNucAvgIntenCh1 | | NeuronalProfilingV42Well:%HIGH\_E2CellBodyNucCountCh2 | | NeuronalProfilingV42Well:%HIGH\_E2CellBodyNucTotalAreaCh1 | | NeuronalProfilingV42Well:%HIGH\_E2CellBodyNucTotalIntenCh1 | | NeuronalProfilingV42Well:%HIGH\_E2CellBodyShapeLWRCh2 | | NeuronalProfilingV42Well:%HIGH\_E2CellBodyShapeP2ACh2 | | NeuronalProfilingV42Well:%HIGH\_E2CellBodySpot%OverlapAreaCh3 | | NeuronalProfilingV42Well:%HIGH\_E2CellBodySpot%OverlapCountCh3 | | NeuronalProfilingV42Well:%HIGH\_E2CellBodySpotAvgIntenCh3 | | NeuronalProfilingV42Well:%HIGH\_E2CellBodySpotOverlapAreaCh3 | | NeuronalProfilingV42Well:%HIGH\_E2CellBodySpotOverlapCountCh3 | | NeuronalProfilingV42Well:%HIGH\_E2CellBodySpotTotalAreaCh3 | | NeuronalProfilingV42Well:%HIGH\_E2CellBodySpotTotalCountCh3 | | NeuronalProfilingV42Well:%HIGH\_E2CellBodySpotTotalIntenCh3 | | NeuronalProfilingV42Well:%HIGH\_E2CellBodySpotTotalIntenRatioCh3Ch2 | | NeuronalProfilingV42Well:%HIGH\_E2CellBodyTotalIntenCh2 | | NeuronalProfilingV42Well:%HIGH\_E2CrossPointAvgCountCh2 | | NeuronalProfilingV42Well:%HIGH\_E2CrossPointTotalCountCh2 | | NeuronalProfilingV42Well:%HIGH\_E2NeuriteAvgIntenCh2 | | NeuronalProfilingV42Well:%HIGH\_E2NeuriteAvgLengthCh2 | | NeuronalProfilingV42Well:%HIGH\_E2NeuriteCriticalValueCh2 | | NeuronalProfilingV42Well:%HIGH\_E2NeuriteDendriteMaxCh2 | | NeuronalProfilingV42Well:%HIGH\_E2NeuriteMaxLengthWithBranchesCh2 | | NeuronalProfilingV42Well:%HIGH\_E2NeuriteMaxLengthWithoutBranchesCh2 | | NeuronalProfilingV42Well:%HIGH\_E2NeuriteRamificationIndexCh2 | | NeuronalProfilingV42Well:%HIGH\_E2NeuriteSpot%OverlapAreaCh3 | | NeuronalProfilingV42Well:%HIGH\_E2NeuriteSpot%OverlapCountCh3 | | NeuronalProfilingV42Well:%HIGH\_E2NeuriteSpotAvgAreaCh3 | | NeuronalProfilingV42Well:%HIGH\_E2NeuriteSpotAvgCountCh3 | | NeuronalProfilingV42Well:%HIGH\_E2NeuriteSpotAvgIntenCh3 | | NeuronalProfilingV42Well:%HIGH\_E2NeuriteSpotOverlapAreaCh3 | | NeuronalProfilingV42Well:%HIGH\_E2NeuriteSpotOverlapCountCh3 | | NeuronalProfilingV42Well:%HIGH\_E2NeuriteSpotTotalAreaCh3 | | NeuronalProfilingV42Well:%HIGH\_E2NeuriteSpotTotalCountCh3 | | NeuronalProfilingV42Well:%HIGH\_E2NeuriteSpotTotalIntenCh3 | | NeuronalProfilingV42Well:%HIGH\_E2NeuriteSpotTotalIntenRatioCh3Ch2 | | NeuronalProfilingV42Well:%HIGH\_E2NeuriteTotalAreaCh2 | | NeuronalProfilingV42Well:%HIGH\_E2NeuriteTotalCountCh2 | | NeuronalProfilingV42Well:%HIGH\_E2NeuriteTotalIntenCh2 | | NeuronalProfilingV42Well:%HIGH\_E2NeuriteTotalLengthCh2 | | NeuronalProfilingV42Well:%HIGH\_E2NeuriteVarIntenCh2 | | NeuronalProfilingV42Well:%HIGH\_E2NeuriteWidthCh2 | | NeuronalProfilingV42Well:%HIGH\_E2TotalIntenCh3 | | NeuronalProfilingV42Well:%HIGH\_E3AvgIntenCh3 | | NeuronalProfilingV42Well:%HIGH\_E3BranchPointAvgCountCh2 | | NeuronalProfilingV42Well:%HIGH\_E3BranchPointAvgDistFromCellBodyCh2 | | NeuronalProfilingV42Well:%HIGH\_E3BranchPointCountPerNeuriteLengthCh2 | | NeuronalProfilingV42Well:%HIGH\_E3BranchPointTotalCountCh2 | | NeuronalProfilingV42Well:%HIGH\_E3CellBodyAreaCh2 | | NeuronalProfilingV42Well:%HIGH\_E3CellBodyAvgIntenCh2 | | NeuronalProfilingV42Well:%HIGH\_E3CellBodyNucAvgIntenCh1 | | NeuronalProfilingV42Well:%HIGH\_E3CellBodyNucCountCh2 | | NeuronalProfilingV42Well:%HIGH\_E3CellBodyNucTotalAreaCh1 | | NeuronalProfilingV42Well:%HIGH\_E3CellBodyNucTotalIntenCh1 | | NeuronalProfilingV42Well:%HIGH\_E3CellBodyShapeLWRCh2 | | NeuronalProfilingV42Well:%HIGH\_E3CellBodyShapeP2ACh2 | | NeuronalProfilingV42Well:%HIGH\_E3CellBodySpot%OverlapAreaCh3 | | NeuronalProfilingV42Well:%HIGH\_E3CellBodySpot%OverlapCountCh3 | | NeuronalProfilingV42Well:%HIGH\_E3CellBodySpotAvgIntenCh3 | | NeuronalProfilingV42Well:%HIGH\_E3CellBodySpotOverlapAreaCh3 | | NeuronalProfilingV42Well:%HIGH\_E3CellBodySpotOverlapCountCh3 | | NeuronalProfilingV42Well:%HIGH\_E3CellBodySpotTotalAreaCh3 | | NeuronalProfilingV42Well:%HIGH\_E3CellBodySpotTotalCountCh3 | | NeuronalProfilingV42Well:%HIGH\_E3CellBodySpotTotalIntenCh3 | | NeuronalProfilingV42Well:%HIGH\_E3CellBodySpotTotalIntenRatioCh3Ch2 | | NeuronalProfilingV42Well:%HIGH\_E3CellBodyTotalIntenCh2 | | NeuronalProfilingV42Well:%HIGH\_E3CrossPointAvgCountCh2 | | NeuronalProfilingV42Well:%HIGH\_E3CrossPointTotalCountCh2 | | NeuronalProfilingV42Well:%HIGH\_E3NeuriteAvgIntenCh2 | | NeuronalProfilingV42Well:%HIGH\_E3NeuriteAvgLengthCh2 | | NeuronalProfilingV42Well:%HIGH\_E3NeuriteCriticalValueCh2 | | NeuronalProfilingV42Well:%HIGH\_E3NeuriteDendriteMaxCh2 | | NeuronalProfilingV42Well:%HIGH\_E3NeuriteMaxLengthWithBranchesCh2 | | NeuronalProfilingV42Well:%HIGH\_E3NeuriteMaxLengthWithoutBranchesCh2 | | NeuronalProfilingV42Well:%HIGH\_E3NeuriteRamificationIndexCh2 | | NeuronalProfilingV42Well:%HIGH\_E3NeuriteSpot%OverlapAreaCh3 | | NeuronalProfilingV42Well:%HIGH\_E3NeuriteSpot%OverlapCountCh3 | | NeuronalProfilingV42Well:%HIGH\_E3NeuriteSpotAvgAreaCh3 | | NeuronalProfilingV42Well:%HIGH\_E3NeuriteSpotAvgCountCh3 | | NeuronalProfilingV42Well:%HIGH\_E3NeuriteSpotAvgIntenCh3 | | NeuronalProfilingV42Well:%HIGH\_E3NeuriteSpotOverlapAreaCh3 | | NeuronalProfilingV42Well:%HIGH\_E3NeuriteSpotOverlapCountCh3 | | NeuronalProfilingV42Well:%HIGH\_E3NeuriteSpotTotalAreaCh3 | | NeuronalProfilingV42Well:%HIGH\_E3NeuriteSpotTotalCountCh3 | | NeuronalProfilingV42Well:%HIGH\_E3NeuriteSpotTotalIntenCh3 | | NeuronalProfilingV42Well:%HIGH\_E3NeuriteSpotTotalIntenRatioCh3Ch2 | | NeuronalProfilingV42Well:%HIGH\_E3NeuriteTotalAreaCh2 | | NeuronalProfilingV42Well:%HIGH\_E3NeuriteTotalCountCh2 | | NeuronalProfilingV42Well:%HIGH\_E3NeuriteTotalIntenCh2 | | NeuronalProfilingV42Well:%HIGH\_E3NeuriteTotalLengthCh2 | | NeuronalProfilingV42Well:%HIGH\_E3NeuriteVarIntenCh2 | | NeuronalProfilingV42Well:%HIGH\_E3NeuriteWidthCh2 | | NeuronalProfilingV42Well:%HIGH\_E3TotalIntenCh3 | | NeuronalProfilingV42Well:%HIGH\_NeuriteAvgIntenCh2 | | NeuronalProfilingV42Well:%HIGH\_NeuriteAvgLengthCh2 | | NeuronalProfilingV42Well:%HIGH\_NeuriteCriticalValueCh2 | | NeuronalProfilingV42Well:%HIGH\_NeuriteDendriteMaxCh2 | | NeuronalProfilingV42Well:%HIGH\_NeuriteMaxLengthWithBranchesCh2 | | NeuronalProfilingV42Well:%HIGH\_NeuriteMaxLengthWithoutBranchesCh2 | | NeuronalProfilingV42Well:%HIGH\_NeuriteRamificationIndexCh2 | | NeuronalProfilingV42Well:%HIGH\_NeuriteSpot%OverlapAreaCh3 | | NeuronalProfilingV42Well:%HIGH\_NeuriteSpot%OverlapCountCh3 | | NeuronalProfilingV42Well:%HIGH\_NeuriteSpotAvgAreaCh3 | | NeuronalProfilingV42Well:%HIGH\_NeuriteSpotAvgCountCh3 | | NeuronalProfilingV42Well:%HIGH\_NeuriteSpotAvgIntenCh3 | | NeuronalProfilingV42Well:%HIGH\_NeuriteSpotOverlapAreaCh3 | | NeuronalProfilingV42Well:%HIGH\_NeuriteSpotOverlapCountCh3 | | NeuronalProfilingV42Well:%HIGH\_NeuriteSpotTotalAreaCh3 | | NeuronalProfilingV42Well:%HIGH\_NeuriteSpotTotalCountCh3 | | NeuronalProfilingV42Well:%HIGH\_NeuriteSpotTotalIntenCh3 | | NeuronalProfilingV42Well:%HIGH\_NeuriteSpotTotalIntenRatioCh3Ch2 | | NeuronalProfilingV42Well:%HIGH\_NeuriteTotalAreaCh2 | | NeuronalProfilingV42Well:%HIGH\_NeuriteTotalCountCh2 | | NeuronalProfilingV42Well:%HIGH\_NeuriteTotalIntenCh2 | | NeuronalProfilingV42Well:%HIGH\_NeuriteTotalLengthCh2 | | NeuronalProfilingV42Well:%HIGH\_NeuriteVarIntenCh2 | | NeuronalProfilingV42Well:%HIGH\_NeuriteWidthCh2 | | NeuronalProfilingV42Well:%HIGH\_TotalIntenCh3 | | NeuronalProfilingV42Well:%NeuronalCells | | NeuronalProfilingV42Well:%NonNeuronalCells | | NeuronalProfilingV42Well:%SelectedNeurons | | NeuronalProfilingV42Well:BranchPointCountPerNeuriteLengthCh2 | | NeuronalProfilingV42Well:BranchPointTotalCountPerFieldCh2 | | NeuronalProfilingV42Well:BranchPointTotalCountPerNeuriteCh2 | | NeuronalProfilingV42Well:BranchPointTotalCountPerNeuronCh2 | | NeuronalProfilingV42Well:BranchPointTotalCountPerWellCh2 | | NeuronalProfilingV42Well:CellBodySpotAvgIntenPerFieldCh3 | | NeuronalProfilingV42Well:CellBodySpotTotalAreaPerFieldCh3 | | NeuronalProfilingV42Well:CellBodySpotTotalAreaPerNeuronCh3 | | NeuronalProfilingV42Well:CellBodySpotTotalAreaPerWellCh3 | | NeuronalProfilingV42Well:CellBodySpotTotalCountPerFieldCh3 | | NeuronalProfilingV42Well:CellBodySpotTotalCountPerNeuronCh3 | | NeuronalProfilingV42Well:CellBodySpotTotalCountPerWellCh3 | | NeuronalProfilingV42Well:CellBodySpotTotalIntenPerFieldCh3 | | NeuronalProfilingV42Well:CellBodySpotTotalIntenPerNeuronCh3 | | NeuronalProfilingV42Well:CellBodySpotTotalIntenPerWellCh3 | | NeuronalProfilingV42Well:CV\_AvgIntenCh3 | | NeuronalProfilingV42Well:CV\_BranchPointAvgCountCh2 | | NeuronalProfilingV42Well:CV\_BranchPointAvgDistFromCellBodyCh2 | | NeuronalProfilingV42Well:CV\_BranchPointCountPerNeuriteLengthCh2 | | NeuronalProfilingV42Well:CV\_BranchPointTotalCountCh2 | | NeuronalProfilingV42Well:CV\_CellBodyAreaCh2 | | NeuronalProfilingV42Well:CV\_CellBodyAvgIntenCh2 | | NeuronalProfilingV42Well:CV\_CellBodyNucAvgIntenCh1 | | NeuronalProfilingV42Well:CV\_CellBodyNucCountCh2 | | NeuronalProfilingV42Well:CV\_CellBodyNucTotalAreaCh1 | | NeuronalProfilingV42Well:CV\_CellBodyNucTotalIntenCh1 | | NeuronalProfilingV42Well:CV\_CellBodyShapeLWRCh2 | | NeuronalProfilingV42Well:CV\_CellBodyShapeP2ACh2 | | NeuronalProfilingV42Well:CV\_CellBodySpot%OverlapAreaCh3 | | NeuronalProfilingV42Well:CV\_CellBodySpot%OverlapCountCh3 | | NeuronalProfilingV42Well:CV\_CellBodySpotAvgIntenCh3 | | NeuronalProfilingV42Well:CV\_CellBodySpotOverlapAreaCh3 | | NeuronalProfilingV42Well:CV\_CellBodySpotOverlapCountCh3 | | NeuronalProfilingV42Well:CV\_CellBodySpotTotalAreaCh3 | | NeuronalProfilingV42Well:CV\_CellBodySpotTotalCountCh3 | | NeuronalProfilingV42Well:CV\_CellBodySpotTotalIntenCh3 | | NeuronalProfilingV42Well:CV\_CellBodySpotTotalIntenRatioCh3Ch2 | | NeuronalProfilingV42Well:CV\_CellBodyTotalIntenCh2 | | NeuronalProfilingV42Well:CV\_CrossPointAvgCountCh2 | | NeuronalProfilingV42Well:CV\_CrossPointTotalCountCh2 | | NeuronalProfilingV42Well:CV\_E1AvgIntenCh3 | | NeuronalProfilingV42Well:CV\_E1BranchPointAvgCountCh2 | | NeuronalProfilingV42Well:CV\_E1BranchPointAvgDistFromCellBodyCh2 | | NeuronalProfilingV42Well:CV\_E1BranchPointCountPerNeuriteLengthCh2 | | NeuronalProfilingV42Well:CV\_E1BranchPointTotalCountCh2 | | NeuronalProfilingV42Well:CV\_E1CellBodyAreaCh2 | | NeuronalProfilingV42Well:CV\_E1CellBodyAvgIntenCh2 | | NeuronalProfilingV42Well:CV\_E1CellBodyNucAvgIntenCh1 | | NeuronalProfilingV42Well:CV\_E1CellBodyNucCountCh2 | | NeuronalProfilingV42Well:CV\_E1CellBodyNucTotalAreaCh1 | | NeuronalProfilingV42Well:CV\_E1CellBodyNucTotalIntenCh1 | | NeuronalProfilingV42Well:CV\_E1CellBodyShapeLWRCh2 | | NeuronalProfilingV42Well:CV\_E1CellBodyShapeP2ACh2 | | NeuronalProfilingV42Well:CV\_E1CellBodySpot%OverlapAreaCh3 | | NeuronalProfilingV42Well:CV\_E1CellBodySpot%OverlapCountCh3 | | NeuronalProfilingV42Well:CV\_E1CellBodySpotAvgIntenCh3 | | NeuronalProfilingV42Well:CV\_E1CellBodySpotOverlapAreaCh3 | | NeuronalProfilingV42Well:CV\_E1CellBodySpotOverlapCountCh3 | | NeuronalProfilingV42Well:CV\_E1CellBodySpotTotalAreaCh3 | | NeuronalProfilingV42Well:CV\_E1CellBodySpotTotalCountCh3 | | NeuronalProfilingV42Well:CV\_E1CellBodySpotTotalIntenCh3 | | NeuronalProfilingV42Well:CV\_E1CellBodySpotTotalIntenRatioCh3Ch2 | | NeuronalProfilingV42Well:CV\_E1CellBodyTotalIntenCh2 | | NeuronalProfilingV42Well:CV\_E1CrossPointAvgCountCh2 | | NeuronalProfilingV42Well:CV\_E1CrossPointTotalCountCh2 | | NeuronalProfilingV42Well:CV\_E1NeuriteAvgIntenCh2 | | NeuronalProfilingV42Well:CV\_E1NeuriteAvgLengthCh2 | | NeuronalProfilingV42Well:CV\_E1NeuriteCriticalValueCh2 | | NeuronalProfilingV42Well:CV\_E1NeuriteDendriteMaxCh2 | | NeuronalProfilingV42Well:CV\_E1NeuriteMaxLengthWithBranchesCh2 | | NeuronalProfilingV42Well:CV\_E1NeuriteMaxLengthWithoutBranchesCh2 | | NeuronalProfilingV42Well:CV\_E1NeuriteRamificationIndexCh2 | | NeuronalProfilingV42Well:CV\_E1NeuriteSpot%OverlapAreaCh3 | | NeuronalProfilingV42Well:CV\_E1NeuriteSpot%OverlapCountCh3 | | NeuronalProfilingV42Well:CV\_E1NeuriteSpotAvgAreaCh3 | | NeuronalProfilingV42Well:CV\_E1NeuriteSpotAvgCountCh3 | | NeuronalProfilingV42Well:CV\_E1NeuriteSpotAvgIntenCh3 | | NeuronalProfilingV42Well:CV\_E1NeuriteSpotOverlapAreaCh3 | | NeuronalProfilingV42Well:CV\_E1NeuriteSpotOverlapCountCh3 | | NeuronalProfilingV42Well:CV\_E1NeuriteSpotTotalAreaCh3 | | NeuronalProfilingV42Well:CV\_E1NeuriteSpotTotalCountCh3 | | NeuronalProfilingV42Well:CV\_E1NeuriteSpotTotalIntenCh3 | | NeuronalProfilingV42Well:CV\_E1NeuriteSpotTotalIntenRatioCh3Ch2 | | NeuronalProfilingV42Well:CV\_E1NeuriteTotalAreaCh2 | | NeuronalProfilingV42Well:CV\_E1NeuriteTotalCountCh2 | | NeuronalProfilingV42Well:CV\_E1NeuriteTotalIntenCh2 | | NeuronalProfilingV42Well:CV\_E1NeuriteTotalLengthCh2 | | NeuronalProfilingV42Well:CV\_E1NeuriteVarIntenCh2 | | NeuronalProfilingV42Well:CV\_E1NeuriteWidthCh2 | | NeuronalProfilingV42Well:CV\_E1TotalIntenCh3 | | NeuronalProfilingV42Well:CV\_E2AvgIntenCh3 | | NeuronalProfilingV42Well:CV\_E2BranchPointAvgCountCh2 | | NeuronalProfilingV42Well:CV\_E2BranchPointAvgDistFromCellBodyCh2 | | NeuronalProfilingV42Well:CV\_E2BranchPointCountPerNeuriteLengthCh2 | | NeuronalProfilingV42Well:CV\_E2BranchPointTotalCountCh2 | | NeuronalProfilingV42Well:CV\_E2CellBodyAreaCh2 | | NeuronalProfilingV42Well:CV\_E2CellBodyAvgIntenCh2 | | NeuronalProfilingV42Well:CV\_E2CellBodyNucAvgIntenCh1 | | NeuronalProfilingV42Well:CV\_E2CellBodyNucCountCh2 | | NeuronalProfilingV42Well:CV\_E2CellBodyNucTotalAreaCh1 | | NeuronalProfilingV42Well:CV\_E2CellBodyNucTotalIntenCh1 | | NeuronalProfilingV42Well:CV\_E2CellBodyShapeLWRCh2 | | NeuronalProfilingV42Well:CV\_E2CellBodyShapeP2ACh2 | | NeuronalProfilingV42Well:CV\_E2CellBodySpot%OverlapAreaCh3 | | NeuronalProfilingV42Well:CV\_E2CellBodySpot%OverlapCountCh3 | | NeuronalProfilingV42Well:CV\_E2CellBodySpotAvgIntenCh3 | | NeuronalProfilingV42Well:CV\_E2CellBodySpotOverlapAreaCh3 | | NeuronalProfilingV42Well:CV\_E2CellBodySpotOverlapCountCh3 | | NeuronalProfilingV42Well:CV\_E2CellBodySpotTotalAreaCh3 | | NeuronalProfilingV42Well:CV\_E2CellBodySpotTotalCountCh3 | | NeuronalProfilingV42Well:CV\_E2CellBodySpotTotalIntenCh3 | | NeuronalProfilingV42Well:CV\_E2CellBodySpotTotalIntenRatioCh3Ch2 | | NeuronalProfilingV42Well:CV\_E2CellBodyTotalIntenCh2 | | NeuronalProfilingV42Well:CV\_E2CrossPointAvgCountCh2 | | NeuronalProfilingV42Well:CV\_E2CrossPointTotalCountCh2 | | NeuronalProfilingV42Well:CV\_E2NeuriteAvgIntenCh2 | | NeuronalProfilingV42Well:CV\_E2NeuriteAvgLengthCh2 | | NeuronalProfilingV42Well:CV\_E2NeuriteCriticalValueCh2 | | NeuronalProfilingV42Well:CV\_E2NeuriteDendriteMaxCh2 | | NeuronalProfilingV42Well:CV\_E2NeuriteMaxLengthWithBranchesCh2 | | NeuronalProfilingV42Well:CV\_E2NeuriteMaxLengthWithoutBranchesCh2 | | NeuronalProfilingV42Well:CV\_E2NeuriteRamificationIndexCh2 | | NeuronalProfilingV42Well:CV\_E2NeuriteSpot%OverlapAreaCh3 | | NeuronalProfilingV42Well:CV\_E2NeuriteSpot%OverlapCountCh3 | | NeuronalProfilingV42Well:CV\_E2NeuriteSpotAvgAreaCh3 | | NeuronalProfilingV42Well:CV\_E2NeuriteSpotAvgCountCh3 | | NeuronalProfilingV42Well:CV\_E2NeuriteSpotAvgIntenCh3 | | NeuronalProfilingV42Well:CV\_E2NeuriteSpotOverlapAreaCh3 | | NeuronalProfilingV42Well:CV\_E2NeuriteSpotOverlapCountCh3 | | NeuronalProfilingV42Well:CV\_E2NeuriteSpotTotalAreaCh3 | | NeuronalProfilingV42Well:CV\_E2NeuriteSpotTotalCountCh3 | | NeuronalProfilingV42Well:CV\_E2NeuriteSpotTotalIntenCh3 | | NeuronalProfilingV42Well:CV\_E2NeuriteSpotTotalIntenRatioCh3Ch2 | | NeuronalProfilingV42Well:CV\_E2NeuriteTotalAreaCh2 | | NeuronalProfilingV42Well:CV\_E2NeuriteTotalCountCh2 | | NeuronalProfilingV42Well:CV\_E2NeuriteTotalIntenCh2 | | NeuronalProfilingV42Well:CV\_E2NeuriteTotalLengthCh2 | | NeuronalProfilingV42Well:CV\_E2NeuriteVarIntenCh2 | | NeuronalProfilingV42Well:CV\_E2NeuriteWidthCh2 | | NeuronalProfilingV42Well:CV\_E2TotalIntenCh3 | | NeuronalProfilingV42Well:CV\_E3AvgIntenCh3 | | NeuronalProfilingV42Well:CV\_E3BranchPointAvgCountCh2 | | NeuronalProfilingV42Well:CV\_E3BranchPointAvgDistFromCellBodyCh2 | | NeuronalProfilingV42Well:CV\_E3BranchPointCountPerNeuriteLengthCh2 | | NeuronalProfilingV42Well:CV\_E3BranchPointTotalCountCh2 | | NeuronalProfilingV42Well:CV\_E3CellBodyAreaCh2 | | NeuronalProfilingV42Well:CV\_E3CellBodyAvgIntenCh2 | | NeuronalProfilingV42Well:CV\_E3CellBodyNucAvgIntenCh1 | | NeuronalProfilingV42Well:CV\_E3CellBodyNucCountCh2 | | NeuronalProfilingV42Well:CV\_E3CellBodyNucTotalAreaCh1 | | NeuronalProfilingV42Well:CV\_E3CellBodyNucTotalIntenCh1 | | NeuronalProfilingV42Well:CV\_E3CellBodyShapeLWRCh2 | | NeuronalProfilingV42Well:CV\_E3CellBodyShapeP2ACh2 | | NeuronalProfilingV42Well:CV\_E3CellBodySpot%OverlapAreaCh3 | | NeuronalProfilingV42Well:CV\_E3CellBodySpot%OverlapCountCh3 | | NeuronalProfilingV42Well:CV\_E3CellBodySpotAvgIntenCh3 | | NeuronalProfilingV42Well:CV\_E3CellBodySpotOverlapAreaCh3 | | NeuronalProfilingV42Well:CV\_E3CellBodySpotOverlapCountCh3 | | NeuronalProfilingV42Well:CV\_E3CellBodySpotTotalAreaCh3 | | NeuronalProfilingV42Well:CV\_E3CellBodySpotTotalCountCh3 | | NeuronalProfilingV42Well:CV\_E3CellBodySpotTotalIntenCh3 | | NeuronalProfilingV42Well:CV\_E3CellBodySpotTotalIntenRatioCh3Ch2 | | NeuronalProfilingV42Well:CV\_E3CellBodyTotalIntenCh2 | | NeuronalProfilingV42Well:CV\_E3CrossPointAvgCountCh2 | | NeuronalProfilingV42Well:CV\_E3CrossPointTotalCountCh2 | | NeuronalProfilingV42Well:CV\_E3NeuriteAvgIntenCh2 | | NeuronalProfilingV42Well:CV\_E3NeuriteAvgLengthCh2 | | NeuronalProfilingV42Well:CV\_E3NeuriteCriticalValueCh2 | | NeuronalProfilingV42Well:CV\_E3NeuriteDendriteMaxCh2 | | NeuronalProfilingV42Well:CV\_E3NeuriteMaxLengthWithBranchesCh2 | | NeuronalProfilingV42Well:CV\_E3NeuriteMaxLengthWithoutBranchesCh2 | | NeuronalProfilingV42Well:CV\_E3NeuriteRamificationIndexCh2 | | NeuronalProfilingV42Well:CV\_E3NeuriteSpot%OverlapAreaCh3 | | NeuronalProfilingV42Well:CV\_E3NeuriteSpot%OverlapCountCh3 | | NeuronalProfilingV42Well:CV\_E3NeuriteSpotAvgAreaCh3 | | NeuronalProfilingV42Well:CV\_E3NeuriteSpotAvgCountCh3 | | NeuronalProfilingV42Well:CV\_E3NeuriteSpotAvgIntenCh3 | | NeuronalProfilingV42Well:CV\_E3NeuriteSpotOverlapAreaCh3 | | NeuronalProfilingV42Well:CV\_E3NeuriteSpotOverlapCountCh3 | | NeuronalProfilingV42Well:CV\_E3NeuriteSpotTotalAreaCh3 | | NeuronalProfilingV42Well:CV\_E3NeuriteSpotTotalCountCh3 | | NeuronalProfilingV42Well:CV\_E3NeuriteSpotTotalIntenCh3 | | NeuronalProfilingV42Well:CV\_E3NeuriteSpotTotalIntenRatioCh3Ch2 | | NeuronalProfilingV42Well:CV\_E3NeuriteTotalAreaCh2 | | NeuronalProfilingV42Well:CV\_E3NeuriteTotalCountCh2 | | NeuronalProfilingV42Well:CV\_E3NeuriteTotalIntenCh2 | | NeuronalProfilingV42Well:CV\_E3NeuriteTotalLengthCh2 | | NeuronalProfilingV42Well:CV\_E3NeuriteVarIntenCh2 | | NeuronalProfilingV42Well:CV\_E3NeuriteWidthCh2 | | NeuronalProfilingV42Well:CV\_E3TotalIntenCh3 | | NeuronalProfilingV42Well:CV\_NeuriteAvgIntenCh2 | | NeuronalProfilingV42Well:CV\_NeuriteAvgLengthCh2 | | NeuronalProfilingV42Well:CV\_NeuriteCriticalValueCh2 | | NeuronalProfilingV42Well:CV\_NeuriteDendriteMaxCh2 | | NeuronalProfilingV42Well:CV\_NeuriteMaxLengthWithBranchesCh2 | | NeuronalProfilingV42Well:CV\_NeuriteMaxLengthWithoutBranchesCh2 | | NeuronalProfilingV42Well:CV\_NeuriteRamificationIndexCh2 | | NeuronalProfilingV42Well:CV\_NeuriteSpot%OverlapAreaCh3 | | NeuronalProfilingV42Well:CV\_NeuriteSpot%OverlapCountCh3 | | NeuronalProfilingV42Well:CV\_NeuriteSpotAvgAreaCh3 | | NeuronalProfilingV42Well:CV\_NeuriteSpotAvgCountCh3 | | NeuronalProfilingV42Well:CV\_NeuriteSpotAvgIntenCh3 | | NeuronalProfilingV42Well:CV\_NeuriteSpotOverlapAreaCh3 | | NeuronalProfilingV42Well:CV\_NeuriteSpotOverlapCountCh3 | | NeuronalProfilingV42Well:CV\_NeuriteSpotTotalAreaCh3 | | NeuronalProfilingV42Well:CV\_NeuriteSpotTotalCountCh3 | | NeuronalProfilingV42Well:CV\_NeuriteSpotTotalIntenCh3 | | NeuronalProfilingV42Well:CV\_NeuriteSpotTotalIntenRatioCh3Ch2 | | NeuronalProfilingV42Well:CV\_NeuriteTotalAreaCh2 | | NeuronalProfilingV42Well:CV\_NeuriteTotalCountCh2 | | NeuronalProfilingV42Well:CV\_NeuriteTotalIntenCh2 | | NeuronalProfilingV42Well:CV\_NeuriteTotalLengthCh2 | | NeuronalProfilingV42Well:CV\_NeuriteVarIntenCh2 | | NeuronalProfilingV42Well:CV\_NeuriteWidthCh2 | | NeuronalProfilingV42Well:CV\_TotalIntenCh3 | | NeuronalProfilingV42Well:E1\_%EventType1Neurons | | NeuronalProfilingV42Well:E1\_%EventType2Neurons | | NeuronalProfilingV42Well:E1\_%EventType3Neurons | | NeuronalProfilingV42Well:E1\_EventType1NeuronCount | | NeuronalProfilingV42Well:E1\_EventType2NeuronCount | | NeuronalProfilingV42Well:E1\_EventType3NeuronCount | | NeuronalProfilingV42Well:E2\_%EventType1Neurons | | NeuronalProfilingV42Well:E2\_%EventType2Neurons | | NeuronalProfilingV42Well:E2\_%EventType3Neurons | | NeuronalProfilingV42Well:E2\_EventType1NeuronCount | | NeuronalProfilingV42Well:E2\_EventType2NeuronCount | | NeuronalProfilingV42Well:E2\_EventType3NeuronCount | | NeuronalProfilingV42Well:E3\_%EventType1Neurons | | NeuronalProfilingV42Well:E3\_%EventType2Neurons | | NeuronalProfilingV42Well:E3\_%EventType3Neurons | | NeuronalProfilingV42Well:E3\_EventType1NeuronCount | | NeuronalProfilingV42Well:E3\_EventType2NeuronCount | | NeuronalProfilingV42Well:E3\_EventType3NeuronCount | | NeuronalProfilingV42Well:EventType1NeuronCount | | NeuronalProfilingV42Well:EventType2NeuronCount | | NeuronalProfilingV42Well:EventType3NeuronCount | | NeuronalProfilingV42Well:MAD\_AvgIntenCh3 | | NeuronalProfilingV42Well:MAD\_BranchPointAvgCountCh2 | | NeuronalProfilingV42Well:MAD\_BranchPointAvgDistFromCellBodyCh2 | | NeuronalProfilingV42Well:MAD\_BranchPointCountPerNeuriteLengthCh2 | | NeuronalProfilingV42Well:MAD\_BranchPointTotalCountCh2 | | NeuronalProfilingV42Well:MAD\_CellBodyAreaCh2 | | NeuronalProfilingV42Well:MAD\_CellBodyAvgIntenCh2 | | NeuronalProfilingV42Well:MAD\_CellBodyNucAvgIntenCh1 | | NeuronalProfilingV42Well:MAD\_CellBodyNucCountCh2 | | NeuronalProfilingV42Well:MAD\_CellBodyNucTotalAreaCh1 | | NeuronalProfilingV42Well:MAD\_CellBodyNucTotalIntenCh1 | | NeuronalProfilingV42Well:MAD\_CellBodyShapeLWRCh2 | | NeuronalProfilingV42Well:MAD\_CellBodyShapeP2ACh2 | | NeuronalProfilingV42Well:MAD\_CellBodySpot%OverlapAreaCh3 | | NeuronalProfilingV42Well:MAD\_CellBodySpot%OverlapCountCh3 | | NeuronalProfilingV42Well:MAD\_CellBodySpotAvgIntenCh3 | | NeuronalProfilingV42Well:MAD\_CellBodySpotOverlapAreaCh3 | | NeuronalProfilingV42Well:MAD\_CellBodySpotOverlapCountCh3 | | NeuronalProfilingV42Well:MAD\_CellBodySpotTotalAreaCh3 | | NeuronalProfilingV42Well:MAD\_CellBodySpotTotalCountCh3 | | NeuronalProfilingV42Well:MAD\_CellBodySpotTotalIntenCh3 | | NeuronalProfilingV42Well:MAD\_CellBodySpotTotalIntenRatioCh3Ch2 | | NeuronalProfilingV42Well:MAD\_CellBodyTotalIntenCh2 | | NeuronalProfilingV42Well:MAD\_CrossPointAvgCountCh2 | | NeuronalProfilingV42Well:MAD\_CrossPointTotalCountCh2 | | NeuronalProfilingV42Well:MAD\_E1AvgIntenCh3 | | NeuronalProfilingV42Well:MAD\_E1BranchPointAvgCountCh2 | | NeuronalProfilingV42Well:MAD\_E1BranchPointAvgDistFromCellBodyCh2 | | NeuronalProfilingV42Well:MAD\_E1BranchPointCountPerNeuriteLengthCh2 | | NeuronalProfilingV42Well:MAD\_E1BranchPointTotalCountCh2 | | NeuronalProfilingV42Well:MAD\_E1CellBodyAreaCh2 | | NeuronalProfilingV42Well:MAD\_E1CellBodyAvgIntenCh2 | | NeuronalProfilingV42Well:MAD\_E1CellBodyNucAvgIntenCh1 | | NeuronalProfilingV42Well:MAD\_E1CellBodyNucCountCh2 | | NeuronalProfilingV42Well:MAD\_E1CellBodyNucTotalAreaCh1 | | NeuronalProfilingV42Well:MAD\_E1CellBodyNucTotalIntenCh1 | | NeuronalProfilingV42Well:MAD\_E1CellBodyShapeLWRCh2 | | NeuronalProfilingV42Well:MAD\_E1CellBodyShapeP2ACh2 | | NeuronalProfilingV42Well:MAD\_E1CellBodySpot%OverlapAreaCh3 | | NeuronalProfilingV42Well:MAD\_E1CellBodySpot%OverlapCountCh3 | | NeuronalProfilingV42Well:MAD\_E1CellBodySpotAvgIntenCh3 | | NeuronalProfilingV42Well:MAD\_E1CellBodySpotOverlapAreaCh3 | | NeuronalProfilingV42Well:MAD\_E1CellBodySpotOverlapCountCh3 | | NeuronalProfilingV42Well:MAD\_E1CellBodySpotTotalAreaCh3 | | NeuronalProfilingV42Well:MAD\_E1CellBodySpotTotalCountCh3 | | NeuronalProfilingV42Well:MAD\_E1CellBodySpotTotalIntenCh3 | | NeuronalProfilingV42Well:MAD\_E1CellBodySpotTotalIntenRatioCh3Ch2 | | NeuronalProfilingV42Well:MAD\_E1CellBodyTotalIntenCh2 | | NeuronalProfilingV42Well:MAD\_E1CrossPointAvgCountCh2 | | NeuronalProfilingV42Well:MAD\_E1CrossPointTotalCountCh2 | | NeuronalProfilingV42Well:MAD\_E1NeuriteAvgIntenCh2 | | NeuronalProfilingV42Well:MAD\_E1NeuriteAvgLengthCh2 | | NeuronalProfilingV42Well:MAD\_E1NeuriteCriticalValueCh2 | | NeuronalProfilingV42Well:MAD\_E1NeuriteDendriteMaxCh2 | | NeuronalProfilingV42Well:MAD\_E1NeuriteMaxLengthWithBranchesCh2 | | NeuronalProfilingV42Well:MAD\_E1NeuriteMaxLengthWithoutBranchesCh2 | | NeuronalProfilingV42Well:MAD\_E1NeuriteRamificationIndexCh2 | | NeuronalProfilingV42Well:MAD\_E1NeuriteSpot%OverlapAreaCh3 | | NeuronalProfilingV42Well:MAD\_E1NeuriteSpot%OverlapCountCh3 | | NeuronalProfilingV42Well:MAD\_E1NeuriteSpotAvgAreaCh3 | | NeuronalProfilingV42Well:MAD\_E1NeuriteSpotAvgCountCh3 | | NeuronalProfilingV42Well:MAD\_E1NeuriteSpotAvgIntenCh3 | | NeuronalProfilingV42Well:MAD\_E1NeuriteSpotOverlapAreaCh3 | | NeuronalProfilingV42Well:MAD\_E1NeuriteSpotOverlapCountCh3 | | NeuronalProfilingV42Well:MAD\_E1NeuriteSpotTotalAreaCh3 | | NeuronalProfilingV42Well:MAD\_E1NeuriteSpotTotalCountCh3 | | NeuronalProfilingV42Well:MAD\_E1NeuriteSpotTotalIntenCh3 | | NeuronalProfilingV42Well:MAD\_E1NeuriteSpotTotalIntenRatioCh3Ch2 | | NeuronalProfilingV42Well:MAD\_E1NeuriteTotalAreaCh2 | | NeuronalProfilingV42Well:MAD\_E1NeuriteTotalCountCh2 | | NeuronalProfilingV42Well:MAD\_E1NeuriteTotalIntenCh2 | | NeuronalProfilingV42Well:MAD\_E1NeuriteTotalLengthCh2 | | NeuronalProfilingV42Well:MAD\_E1NeuriteVarIntenCh2 | | NeuronalProfilingV42Well:MAD\_E1NeuriteWidthCh2 | | NeuronalProfilingV42Well:MAD\_E1TotalIntenCh3 | | NeuronalProfilingV42Well:MAD\_E2AvgIntenCh3 | | NeuronalProfilingV42Well:MAD\_E2BranchPointAvgCountCh2 | | NeuronalProfilingV42Well:MAD\_E2BranchPointAvgDistFromCellBodyCh2 | | NeuronalProfilingV42Well:MAD\_E2BranchPointCountPerNeuriteLengthCh2 | | NeuronalProfilingV42Well:MAD\_E2BranchPointTotalCountCh2 | | NeuronalProfilingV42Well:MAD\_E2CellBodyAreaCh2 | | NeuronalProfilingV42Well:MAD\_E2CellBodyAvgIntenCh2 | | NeuronalProfilingV42Well:MAD\_E2CellBodyNucAvgIntenCh1 | | NeuronalProfilingV42Well:MAD\_E2CellBodyNucCountCh2 | | NeuronalProfilingV42Well:MAD\_E2CellBodyNucTotalAreaCh1 | | NeuronalProfilingV42Well:MAD\_E2CellBodyNucTotalIntenCh1 | | NeuronalProfilingV42Well:MAD\_E2CellBodyShapeLWRCh2 | | NeuronalProfilingV42Well:MAD\_E2CellBodyShapeP2ACh2 | | NeuronalProfilingV42Well:MAD\_E2CellBodySpot%OverlapAreaCh3 | | NeuronalProfilingV42Well:MAD\_E2CellBodySpot%OverlapCountCh3 | | NeuronalProfilingV42Well:MAD\_E2CellBodySpotAvgIntenCh3 | | NeuronalProfilingV42Well:MAD\_E2CellBodySpotOverlapAreaCh3 | | NeuronalProfilingV42Well:MAD\_E2CellBodySpotOverlapCountCh3 | | NeuronalProfilingV42Well:MAD\_E2CellBodySpotTotalAreaCh3 | | NeuronalProfilingV42Well:MAD\_E2CellBodySpotTotalCountCh3 | | NeuronalProfilingV42Well:MAD\_E2CellBodySpotTotalIntenCh3 | | NeuronalProfilingV42Well:MAD\_E2CellBodySpotTotalIntenRatioCh3Ch2 | | NeuronalProfilingV42Well:MAD\_E2CellBodyTotalIntenCh2 | | NeuronalProfilingV42Well:MAD\_E2CrossPointAvgCountCh2 | | NeuronalProfilingV42Well:MAD\_E2CrossPointTotalCountCh2 | | NeuronalProfilingV42Well:MAD\_E2NeuriteAvgIntenCh2 | | NeuronalProfilingV42Well:MAD\_E2NeuriteAvgLengthCh2 | | NeuronalProfilingV42Well:MAD\_E2NeuriteCriticalValueCh2 | | NeuronalProfilingV42Well:MAD\_E2NeuriteDendriteMaxCh2 | | NeuronalProfilingV42Well:MAD\_E2NeuriteMaxLengthWithBranchesCh2 | | NeuronalProfilingV42Well:MAD\_E2NeuriteMaxLengthWithoutBranchesCh2 | | NeuronalProfilingV42Well:MAD\_E2NeuriteRamificationIndexCh2 | | NeuronalProfilingV42Well:MAD\_E2NeuriteSpot%OverlapAreaCh3 | | NeuronalProfilingV42Well:MAD\_E2NeuriteSpot%OverlapCountCh3 | | NeuronalProfilingV42Well:MAD\_E2NeuriteSpotAvgAreaCh3 | | NeuronalProfilingV42Well:MAD\_E2NeuriteSpotAvgCountCh3 | | NeuronalProfilingV42Well:MAD\_E2NeuriteSpotAvgIntenCh3 | | NeuronalProfilingV42Well:MAD\_E2NeuriteSpotOverlapAreaCh3 | | NeuronalProfilingV42Well:MAD\_E2NeuriteSpotOverlapCountCh3 | | NeuronalProfilingV42Well:MAD\_E2NeuriteSpotTotalAreaCh3 | | NeuronalProfilingV42Well:MAD\_E2NeuriteSpotTotalCountCh3 | | NeuronalProfilingV42Well:MAD\_E2NeuriteSpotTotalIntenCh3 | | NeuronalProfilingV42Well:MAD\_E2NeuriteSpotTotalIntenRatioCh3Ch2 | | NeuronalProfilingV42Well:MAD\_E2NeuriteTotalAreaCh2 | | NeuronalProfilingV42Well:MAD\_E2NeuriteTotalCountCh2 | | NeuronalProfilingV42Well:MAD\_E2NeuriteTotalIntenCh2 | | NeuronalProfilingV42Well:MAD\_E2NeuriteTotalLengthCh2 | | NeuronalProfilingV42Well:MAD\_E2NeuriteVarIntenCh2 | | NeuronalProfilingV42Well:MAD\_E2NeuriteWidthCh2 | | NeuronalProfilingV42Well:MAD\_E2TotalIntenCh3 | | NeuronalProfilingV42Well:MAD\_E3AvgIntenCh3 | | NeuronalProfilingV42Well:MAD\_E3BranchPointAvgCountCh2 | | NeuronalProfilingV42Well:MAD\_E3BranchPointAvgDistFromCellBodyCh2 | | NeuronalProfilingV42Well:MAD\_E3BranchPointCountPerNeuriteLengthCh2 | | NeuronalProfilingV42Well:MAD\_E3BranchPointTotalCountCh2 | | NeuronalProfilingV42Well:MAD\_E3CellBodyAreaCh2 | | NeuronalProfilingV42Well:MAD\_E3CellBodyAvgIntenCh2 | | NeuronalProfilingV42Well:MAD\_E3CellBodyNucAvgIntenCh1 | | NeuronalProfilingV42Well:MAD\_E3CellBodyNucCountCh2 | | NeuronalProfilingV42Well:MAD\_E3CellBodyNucTotalAreaCh1 | | NeuronalProfilingV42Well:MAD\_E3CellBodyNucTotalIntenCh1 | | NeuronalProfilingV42Well:MAD\_E3CellBodyShapeLWRCh2 | | NeuronalProfilingV42Well:MAD\_E3CellBodyShapeP2ACh2 | | NeuronalProfilingV42Well:MAD\_E3CellBodySpot%OverlapAreaCh3 | | NeuronalProfilingV42Well:MAD\_E3CellBodySpot%OverlapCountCh3 | | NeuronalProfilingV42Well:MAD\_E3CellBodySpotAvgIntenCh3 | | NeuronalProfilingV42Well:MAD\_E3CellBodySpotOverlapAreaCh3 | | NeuronalProfilingV42Well:MAD\_E3CellBodySpotOverlapCountCh3 | | NeuronalProfilingV42Well:MAD\_E3CellBodySpotTotalAreaCh3 | | NeuronalProfilingV42Well:MAD\_E3CellBodySpotTotalCountCh3 | | NeuronalProfilingV42Well:MAD\_E3CellBodySpotTotalIntenCh3 | | NeuronalProfilingV42Well:MAD\_E3CellBodySpotTotalIntenRatioCh3Ch2 | | NeuronalProfilingV42Well:MAD\_E3CellBodyTotalIntenCh2 | | NeuronalProfilingV42Well:MAD\_E3CrossPointAvgCountCh2 | | NeuronalProfilingV42Well:MAD\_E3CrossPointTotalCountCh2 | | NeuronalProfilingV42Well:MAD\_E3NeuriteAvgIntenCh2 | | NeuronalProfilingV42Well:MAD\_E3NeuriteAvgLengthCh2 | | NeuronalProfilingV42Well:MAD\_E3NeuriteCriticalValueCh2 | | NeuronalProfilingV42Well:MAD\_E3NeuriteDendriteMaxCh2 | | NeuronalProfilingV42Well:MAD\_E3NeuriteMaxLengthWithBranchesCh2 | | NeuronalProfilingV42Well:MAD\_E3NeuriteMaxLengthWithoutBranchesCh2 | | NeuronalProfilingV42Well:MAD\_E3NeuriteRamificationIndexCh2 | | NeuronalProfilingV42Well:MAD\_E3NeuriteSpot%OverlapAreaCh3 | | NeuronalProfilingV42Well:MAD\_E3NeuriteSpot%OverlapCountCh3 | | NeuronalProfilingV42Well:MAD\_E3NeuriteSpotAvgAreaCh3 | | NeuronalProfilingV42Well:MAD\_E3NeuriteSpotAvgCountCh3 | | NeuronalProfilingV42Well:MAD\_E3NeuriteSpotAvgIntenCh3 | | NeuronalProfilingV42Well:MAD\_E3NeuriteSpotOverlapAreaCh3 | | NeuronalProfilingV42Well:MAD\_E3NeuriteSpotOverlapCountCh3 | | NeuronalProfilingV42Well:MAD\_E3NeuriteSpotTotalAreaCh3 | | NeuronalProfilingV42Well:MAD\_E3NeuriteSpotTotalCountCh3 | | NeuronalProfilingV42Well:MAD\_E3NeuriteSpotTotalIntenCh3 | | NeuronalProfilingV42Well:MAD\_E3NeuriteSpotTotalIntenRatioCh3Ch2 | | NeuronalProfilingV42Well:MAD\_E3NeuriteTotalAreaCh2 | | NeuronalProfilingV42Well:MAD\_E3NeuriteTotalCountCh2 | | NeuronalProfilingV42Well:MAD\_E3NeuriteTotalIntenCh2 | | NeuronalProfilingV42Well:MAD\_E3NeuriteTotalLengthCh2 | | NeuronalProfilingV42Well:MAD\_E3NeuriteVarIntenCh2 | | NeuronalProfilingV42Well:MAD\_E3NeuriteWidthCh2 | | NeuronalProfilingV42Well:MAD\_E3TotalIntenCh3 | | NeuronalProfilingV42Well:MAD\_NeuriteAvgIntenCh2 | | NeuronalProfilingV42Well:MAD\_NeuriteAvgLengthCh2 | | NeuronalProfilingV42Well:MAD\_NeuriteCriticalValueCh2 | | NeuronalProfilingV42Well:MAD\_NeuriteDendriteMaxCh2 | | NeuronalProfilingV42Well:MAD\_NeuriteMaxLengthWithBranchesCh2 | | NeuronalProfilingV42Well:MAD\_NeuriteMaxLengthWithoutBranchesCh2 | | NeuronalProfilingV42Well:MAD\_NeuriteRamificationIndexCh2 | | NeuronalProfilingV42Well:MAD\_NeuriteSpot%OverlapAreaCh3 | | NeuronalProfilingV42Well:MAD\_NeuriteSpot%OverlapCountCh3 | | NeuronalProfilingV42Well:MAD\_NeuriteSpotAvgAreaCh3 | | NeuronalProfilingV42Well:MAD\_NeuriteSpotAvgCountCh3 | | NeuronalProfilingV42Well:MAD\_NeuriteSpotAvgIntenCh3 | | NeuronalProfilingV42Well:MAD\_NeuriteSpotOverlapAreaCh3 | | NeuronalProfilingV42Well:MAD\_NeuriteSpotOverlapCountCh3 | | NeuronalProfilingV42Well:MAD\_NeuriteSpotTotalAreaCh3 | | NeuronalProfilingV42Well:MAD\_NeuriteSpotTotalCountCh3 | | NeuronalProfilingV42Well:MAD\_NeuriteSpotTotalIntenCh3 | | NeuronalProfilingV42Well:MAD\_NeuriteSpotTotalIntenRatioCh3Ch2 | | NeuronalProfilingV42Well:MAD\_NeuriteTotalAreaCh2 | | NeuronalProfilingV42Well:MAD\_NeuriteTotalCountCh2 | | NeuronalProfilingV42Well:MAD\_NeuriteTotalIntenCh2 | | NeuronalProfilingV42Well:MAD\_NeuriteTotalLengthCh2 | | NeuronalProfilingV42Well:MAD\_NeuriteVarIntenCh2 | | NeuronalProfilingV42Well:MAD\_NeuriteWidthCh2 | | NeuronalProfilingV42Well:MAD\_TotalIntenCh3 | | NeuronalProfilingV42Well:MEAN\_AvgIntenCh3 | | NeuronalProfilingV42Well:MEAN\_BranchPointAvgCountCh2 | | NeuronalProfilingV42Well:MEAN\_BranchPointAvgDistFromCellBodyCh2 | | NeuronalProfilingV42Well:MEAN\_BranchPointCountPerNeuriteLengthCh2 | | NeuronalProfilingV42Well:MEAN\_BranchPointTotalCountCh2 | | NeuronalProfilingV42Well:MEAN\_CellBodyAreaCh2 | | NeuronalProfilingV42Well:MEAN\_CellBodyAvgIntenCh2 | | NeuronalProfilingV42Well:MEAN\_CellBodyNucAvgIntenCh1 | | NeuronalProfilingV42Well:MEAN\_CellBodyNucCountCh2 | | NeuronalProfilingV42Well:MEAN\_CellBodyNucTotalAreaCh1 | | NeuronalProfilingV42Well:MEAN\_CellBodyNucTotalIntenCh1 | | NeuronalProfilingV42Well:MEAN\_CellBodyShapeLWRCh2 | | NeuronalProfilingV42Well:MEAN\_CellBodyShapeP2ACh2 | | NeuronalProfilingV42Well:MEAN\_CellBodySpot%OverlapAreaCh3 | | NeuronalProfilingV42Well:MEAN\_CellBodySpot%OverlapCountCh3 | | NeuronalProfilingV42Well:MEAN\_CellBodySpotAvgIntenCh3 | | NeuronalProfilingV42Well:MEAN\_CellBodySpotOverlapAreaCh3 | | NeuronalProfilingV42Well:MEAN\_CellBodySpotOverlapCountCh3 | | NeuronalProfilingV42Well:MEAN\_CellBodySpotTotalAreaCh3 | | NeuronalProfilingV42Well:MEAN\_CellBodySpotTotalCountCh3 | | NeuronalProfilingV42Well:MEAN\_CellBodySpotTotalIntenCh3 | | NeuronalProfilingV42Well:MEAN\_CellBodySpotTotalIntenRatioCh3Ch2 | | NeuronalProfilingV42Well:MEAN\_CellBodyTotalIntenCh2 | | NeuronalProfilingV42Well:MEAN\_CrossPointAvgCountCh2 | | NeuronalProfilingV42Well:MEAN\_CrossPointTotalCountCh2 | | NeuronalProfilingV42Well:MEAN\_E1AvgIntenCh3 | | NeuronalProfilingV42Well:MEAN\_E1BranchPointAvgCountCh2 | | NeuronalProfilingV42Well:MEAN\_E1BranchPointAvgDistFromCellBodyCh2 | | NeuronalProfilingV42Well:MEAN\_E1BranchPointCountPerNeuriteLengthCh2 | | NeuronalProfilingV42Well:MEAN\_E1BranchPointTotalCountCh2 | | NeuronalProfilingV42Well:MEAN\_E1CellBodyAreaCh2 | | NeuronalProfilingV42Well:MEAN\_E1CellBodyAvgIntenCh2 | | NeuronalProfilingV42Well:MEAN\_E1CellBodyNucAvgIntenCh1 | | NeuronalProfilingV42Well:MEAN\_E1CellBodyNucCountCh2 | | NeuronalProfilingV42Well:MEAN\_E1CellBodyNucTotalAreaCh1 | | NeuronalProfilingV42Well:MEAN\_E1CellBodyNucTotalIntenCh1 | | NeuronalProfilingV42Well:MEAN\_E1CellBodyShapeLWRCh2 | | NeuronalProfilingV42Well:MEAN\_E1CellBodyShapeP2ACh2 | | NeuronalProfilingV42Well:MEAN\_E1CellBodySpot%OverlapAreaCh3 | | NeuronalProfilingV42Well:MEAN\_E1CellBodySpot%OverlapCountCh3 | | NeuronalProfilingV42Well:MEAN\_E1CellBodySpotAvgIntenCh3 | | NeuronalProfilingV42Well:MEAN\_E1CellBodySpotOverlapAreaCh3 | | NeuronalProfilingV42Well:MEAN\_E1CellBodySpotOverlapCountCh3 | | NeuronalProfilingV42Well:MEAN\_E1CellBodySpotTotalAreaCh3 | | NeuronalProfilingV42Well:MEAN\_E1CellBodySpotTotalCountCh3 | | NeuronalProfilingV42Well:MEAN\_E1CellBodySpotTotalIntenCh3 | | NeuronalProfilingV42Well:MEAN\_E1CellBodySpotTotalIntenRatioCh3Ch2 | | NeuronalProfilingV42Well:MEAN\_E1CellBodyTotalIntenCh2 | | NeuronalProfilingV42Well:MEAN\_E1CrossPointAvgCountCh2 | | NeuronalProfilingV42Well:MEAN\_E1CrossPointTotalCountCh2 | | NeuronalProfilingV42Well:MEAN\_E1NeuriteAvgIntenCh2 | | NeuronalProfilingV42Well:MEAN\_E1NeuriteAvgLengthCh2 | | NeuronalProfilingV42Well:MEAN\_E1NeuriteCriticalValueCh2 | | NeuronalProfilingV42Well:MEAN\_E1NeuriteDendriteMaxCh2 | | NeuronalProfilingV42Well:MEAN\_E1NeuriteMaxLengthWithBranchesCh2 | | NeuronalProfilingV42Well:MEAN\_E1NeuriteMaxLengthWithoutBranchesCh2 | | NeuronalProfilingV42Well:MEAN\_E1NeuriteRamificationIndexCh2 | | NeuronalProfilingV42Well:MEAN\_E1NeuriteSpot%OverlapAreaCh3 | | NeuronalProfilingV42Well:MEAN\_E1NeuriteSpot%OverlapCountCh3 | | NeuronalProfilingV42Well:MEAN\_E1NeuriteSpotAvgAreaCh3 | | NeuronalProfilingV42Well:MEAN\_E1NeuriteSpotAvgCountCh3 | | NeuronalProfilingV42Well:MEAN\_E1NeuriteSpotAvgIntenCh3 | | NeuronalProfilingV42Well:MEAN\_E1NeuriteSpotOverlapAreaCh3 | | NeuronalProfilingV42Well:MEAN\_E1NeuriteSpotOverlapCountCh3 | | NeuronalProfilingV42Well:MEAN\_E1NeuriteSpotTotalAreaCh3 | | NeuronalProfilingV42Well:MEAN\_E1NeuriteSpotTotalCountCh3 | | NeuronalProfilingV42Well:MEAN\_E1NeuriteSpotTotalIntenCh3 | | NeuronalProfilingV42Well:MEAN\_E1NeuriteSpotTotalIntenRatioCh3Ch2 | | NeuronalProfilingV42Well:MEAN\_E1NeuriteTotalAreaCh2 | | NeuronalProfilingV42Well:MEAN\_E1NeuriteTotalCountCh2 | | NeuronalProfilingV42Well:MEAN\_E1NeuriteTotalIntenCh2 | | NeuronalProfilingV42Well:MEAN\_E1NeuriteTotalLengthCh2 | | NeuronalProfilingV42Well:MEAN\_E1NeuriteVarIntenCh2 | | NeuronalProfilingV42Well:MEAN\_E1NeuriteWidthCh2 | | NeuronalProfilingV42Well:MEAN\_E1TotalIntenCh3 | | NeuronalProfilingV42Well:MEAN\_E2AvgIntenCh3 | | NeuronalProfilingV42Well:MEAN\_E2BranchPointAvgCountCh2 | | NeuronalProfilingV42Well:MEAN\_E2BranchPointAvgDistFromCellBodyCh2 | | NeuronalProfilingV42Well:MEAN\_E2BranchPointCountPerNeuriteLengthCh2 | | NeuronalProfilingV42Well:MEAN\_E2BranchPointTotalCountCh2 | | NeuronalProfilingV42Well:MEAN\_E2CellBodyAreaCh2 | | NeuronalProfilingV42Well:MEAN\_E2CellBodyAvgIntenCh2 | | NeuronalProfilingV42Well:MEAN\_E2CellBodyNucAvgIntenCh1 | | NeuronalProfilingV42Well:MEAN\_E2CellBodyNucCountCh2 | | NeuronalProfilingV42Well:MEAN\_E2CellBodyNucTotalAreaCh1 | | NeuronalProfilingV42Well:MEAN\_E2CellBodyNucTotalIntenCh1 | | NeuronalProfilingV42Well:MEAN\_E2CellBodyShapeLWRCh2 | | NeuronalProfilingV42Well:MEAN\_E2CellBodyShapeP2ACh2 | | NeuronalProfilingV42Well:MEAN\_E2CellBodySpot%OverlapAreaCh3 | | NeuronalProfilingV42Well:MEAN\_E2CellBodySpot%OverlapCountCh3 | | NeuronalProfilingV42Well:MEAN\_E2CellBodySpotAvgIntenCh3 | | NeuronalProfilingV42Well:MEAN\_E2CellBodySpotOverlapAreaCh3 | | NeuronalProfilingV42Well:MEAN\_E2CellBodySpotOverlapCountCh3 | | NeuronalProfilingV42Well:MEAN\_E2CellBodySpotTotalAreaCh3 | | NeuronalProfilingV42Well:MEAN\_E2CellBodySpotTotalCountCh3 | | NeuronalProfilingV42Well:MEAN\_E2CellBodySpotTotalIntenCh3 | | NeuronalProfilingV42Well:MEAN\_E2CellBodySpotTotalIntenRatioCh3Ch2 | | NeuronalProfilingV42Well:MEAN\_E2CellBodyTotalIntenCh2 | | NeuronalProfilingV42Well:MEAN\_E2CrossPointAvgCountCh2 | | NeuronalProfilingV42Well:MEAN\_E2CrossPointTotalCountCh2 | | NeuronalProfilingV42Well:MEAN\_E2NeuriteAvgIntenCh2 | | NeuronalProfilingV42Well:MEAN\_E2NeuriteAvgLengthCh2 | | NeuronalProfilingV42Well:MEAN\_E2NeuriteCriticalValueCh2 | | NeuronalProfilingV42Well:MEAN\_E2NeuriteDendriteMaxCh2 | | NeuronalProfilingV42Well:MEAN\_E2NeuriteMaxLengthWithBranchesCh2 | | NeuronalProfilingV42Well:MEAN\_E2NeuriteMaxLengthWithoutBranchesCh2 | | NeuronalProfilingV42Well:MEAN\_E2NeuriteRamificationIndexCh2 | | NeuronalProfilingV42Well:MEAN\_E2NeuriteSpot%OverlapAreaCh3 | | NeuronalProfilingV42Well:MEAN\_E2NeuriteSpot%OverlapCountCh3 | | NeuronalProfilingV42Well:MEAN\_E2NeuriteSpotAvgAreaCh3 | | NeuronalProfilingV42Well:MEAN\_E2NeuriteSpotAvgCountCh3 | | NeuronalProfilingV42Well:MEAN\_E2NeuriteSpotAvgIntenCh3 | | NeuronalProfilingV42Well:MEAN\_E2NeuriteSpotOverlapAreaCh3 | | NeuronalProfilingV42Well:MEAN\_E2NeuriteSpotOverlapCountCh3 | | NeuronalProfilingV42Well:MEAN\_E2NeuriteSpotTotalAreaCh3 | | NeuronalProfilingV42Well:MEAN\_E2NeuriteSpotTotalCountCh3 | | NeuronalProfilingV42Well:MEAN\_E2NeuriteSpotTotalIntenCh3 | | NeuronalProfilingV42Well:MEAN\_E2NeuriteSpotTotalIntenRatioCh3Ch2 | | NeuronalProfilingV42Well:MEAN\_E2NeuriteTotalAreaCh2 | | NeuronalProfilingV42Well:MEAN\_E2NeuriteTotalCountCh2 | | NeuronalProfilingV42Well:MEAN\_E2NeuriteTotalIntenCh2 | | NeuronalProfilingV42Well:MEAN\_E2NeuriteTotalLengthCh2 | | NeuronalProfilingV42Well:MEAN\_E2NeuriteVarIntenCh2 | | NeuronalProfilingV42Well:MEAN\_E2NeuriteWidthCh2 | | NeuronalProfilingV42Well:MEAN\_E2TotalIntenCh3 | | NeuronalProfilingV42Well:MEAN\_E3AvgIntenCh3 | | NeuronalProfilingV42Well:MEAN\_E3BranchPointAvgCountCh2 | | NeuronalProfilingV42Well:MEAN\_E3BranchPointAvgDistFromCellBodyCh2 | | NeuronalProfilingV42Well:MEAN\_E3BranchPointCountPerNeuriteLengthCh2 | | NeuronalProfilingV42Well:MEAN\_E3BranchPointTotalCountCh2 | | NeuronalProfilingV42Well:MEAN\_E3CellBodyAreaCh2 | | NeuronalProfilingV42Well:MEAN\_E3CellBodyAvgIntenCh2 | | NeuronalProfilingV42Well:MEAN\_E3CellBodyNucAvgIntenCh1 | | NeuronalProfilingV42Well:MEAN\_E3CellBodyNucCountCh2 | | NeuronalProfilingV42Well:MEAN\_E3CellBodyNucTotalAreaCh1 | | NeuronalProfilingV42Well:MEAN\_E3CellBodyNucTotalIntenCh1 | | NeuronalProfilingV42Well:MEAN\_E3CellBodyShapeLWRCh2 | | NeuronalProfilingV42Well:MEAN\_E3CellBodyShapeP2ACh2 | | NeuronalProfilingV42Well:MEAN\_E3CellBodySpot%OverlapAreaCh3 | | NeuronalProfilingV42Well:MEAN\_E3CellBodySpot%OverlapCountCh3 | | NeuronalProfilingV42Well:MEAN\_E3CellBodySpotAvgIntenCh3 | | NeuronalProfilingV42Well:MEAN\_E3CellBodySpotOverlapAreaCh3 | | NeuronalProfilingV42Well:MEAN\_E3CellBodySpotOverlapCountCh3 | | NeuronalProfilingV42Well:MEAN\_E3CellBodySpotTotalAreaCh3 | | NeuronalProfilingV42Well:MEAN\_E3CellBodySpotTotalCountCh3 | | NeuronalProfilingV42Well:MEAN\_E3CellBodySpotTotalIntenCh3 | | NeuronalProfilingV42Well:MEAN\_E3CellBodySpotTotalIntenRatioCh3Ch2 | | NeuronalProfilingV42Well:MEAN\_E3CellBodyTotalIntenCh2 | | NeuronalProfilingV42Well:MEAN\_E3CrossPointAvgCountCh2 | | NeuronalProfilingV42Well:MEAN\_E3CrossPointTotalCountCh2 | | NeuronalProfilingV42Well:MEAN\_E3NeuriteAvgIntenCh2 | | NeuronalProfilingV42Well:MEAN\_E3NeuriteAvgLengthCh2 | | NeuronalProfilingV42Well:MEAN\_E3NeuriteCriticalValueCh2 | | NeuronalProfilingV42Well:MEAN\_E3NeuriteDendriteMaxCh2 | | NeuronalProfilingV42Well:MEAN\_E3NeuriteMaxLengthWithBranchesCh2 | | NeuronalProfilingV42Well:MEAN\_E3NeuriteMaxLengthWithoutBranchesCh2 | | NeuronalProfilingV42Well:MEAN\_E3NeuriteRamificationIndexCh2 | | NeuronalProfilingV42Well:MEAN\_E3NeuriteSpot%OverlapAreaCh3 | | NeuronalProfilingV42Well:MEAN\_E3NeuriteSpot%OverlapCountCh3 | | NeuronalProfilingV42Well:MEAN\_E3NeuriteSpotAvgAreaCh3 | | NeuronalProfilingV42Well:MEAN\_E3NeuriteSpotAvgCountCh3 | | NeuronalProfilingV42Well:MEAN\_E3NeuriteSpotAvgIntenCh3 | | NeuronalProfilingV42Well:MEAN\_E3NeuriteSpotOverlapAreaCh3 | | NeuronalProfilingV42Well:MEAN\_E3NeuriteSpotOverlapCountCh3 | | NeuronalProfilingV42Well:MEAN\_E3NeuriteSpotTotalAreaCh3 | | NeuronalProfilingV42Well:MEAN\_E3NeuriteSpotTotalCountCh3 | | NeuronalProfilingV42Well:MEAN\_E3NeuriteSpotTotalIntenCh3 | | NeuronalProfilingV42Well:MEAN\_E3NeuriteSpotTotalIntenRatioCh3Ch2 | | NeuronalProfilingV42Well:MEAN\_E3NeuriteTotalAreaCh2 | | NeuronalProfilingV42Well:MEAN\_E3NeuriteTotalCountCh2 | | NeuronalProfilingV42Well:MEAN\_E3NeuriteTotalIntenCh2 | | NeuronalProfilingV42Well:MEAN\_E3NeuriteTotalLengthCh2 | | NeuronalProfilingV42Well:MEAN\_E3NeuriteVarIntenCh2 | | NeuronalProfilingV42Well:MEAN\_E3NeuriteWidthCh2 | | NeuronalProfilingV42Well:MEAN\_E3TotalIntenCh3 | | NeuronalProfilingV42Well:MEAN\_NeuriteAvgIntenCh2 | | NeuronalProfilingV42Well:MEAN\_NeuriteAvgLengthCh2 | | NeuronalProfilingV42Well:MEAN\_NeuriteCriticalValueCh2 | | NeuronalProfilingV42Well:MEAN\_NeuriteDendriteMaxCh2 | | NeuronalProfilingV42Well:MEAN\_NeuriteMaxLengthWithBranchesCh2 | | NeuronalProfilingV42Well:MEAN\_NeuriteMaxLengthWithoutBranchesCh2 | | NeuronalProfilingV42Well:MEAN\_NeuriteRamificationIndexCh2 | | NeuronalProfilingV42Well:MEAN\_NeuriteSpot%OverlapAreaCh3 | | NeuronalProfilingV42Well:MEAN\_NeuriteSpot%OverlapCountCh3 | | NeuronalProfilingV42Well:MEAN\_NeuriteSpotAvgAreaCh3 | | NeuronalProfilingV42Well:MEAN\_NeuriteSpotAvgCountCh3 | | NeuronalProfilingV42Well:MEAN\_NeuriteSpotAvgIntenCh3 | | NeuronalProfilingV42Well:MEAN\_NeuriteSpotOverlapAreaCh3 | | NeuronalProfilingV42Well:MEAN\_NeuriteSpotOverlapCountCh3 | | NeuronalProfilingV42Well:MEAN\_NeuriteSpotTotalAreaCh3 | | NeuronalProfilingV42Well:MEAN\_NeuriteSpotTotalCountCh3 | | NeuronalProfilingV42Well:MEAN\_NeuriteSpotTotalIntenCh3 | | NeuronalProfilingV42Well:MEAN\_NeuriteSpotTotalIntenRatioCh3Ch2 | | NeuronalProfilingV42Well:MEAN\_NeuriteTotalAreaCh2 | | NeuronalProfilingV42Well:MEAN\_NeuriteTotalCountCh2 | | NeuronalProfilingV42Well:MEAN\_NeuriteTotalIntenCh2 | | NeuronalProfilingV42Well:MEAN\_NeuriteTotalLengthCh2 | | NeuronalProfilingV42Well:MEAN\_NeuriteVarIntenCh2 | | NeuronalProfilingV42Well:MEAN\_NeuriteWidthCh2 | | NeuronalProfilingV42Well:MEAN\_TotalIntenCh3 | | NeuronalProfilingV42Well:MEDIAN\_AvgIntenCh3 | | NeuronalProfilingV42Well:MEDIAN\_BranchPointAvgCountCh2 | | NeuronalProfilingV42Well:MEDIAN\_BranchPointAvgDistFromCellBodyCh2 | | NeuronalProfilingV42Well:MEDIAN\_BranchPointCountPerNeuriteLengthCh2 | | NeuronalProfilingV42Well:MEDIAN\_BranchPointTotalCountCh2 | | NeuronalProfilingV42Well:MEDIAN\_CellBodyAreaCh2 | | NeuronalProfilingV42Well:MEDIAN\_CellBodyAvgIntenCh2 | | NeuronalProfilingV42Well:MEDIAN\_CellBodyNucAvgIntenCh1 | | NeuronalProfilingV42Well:MEDIAN\_CellBodyNucCountCh2 | | NeuronalProfilingV42Well:MEDIAN\_CellBodyNucTotalAreaCh1 | | NeuronalProfilingV42Well:MEDIAN\_CellBodyNucTotalIntenCh1 | | NeuronalProfilingV42Well:MEDIAN\_CellBodyShapeLWRCh2 | | NeuronalProfilingV42Well:MEDIAN\_CellBodyShapeP2ACh2 | | NeuronalProfilingV42Well:MEDIAN\_CellBodySpot%OverlapAreaCh3 | | NeuronalProfilingV42Well:MEDIAN\_CellBodySpot%OverlapCountCh3 | | NeuronalProfilingV42Well:MEDIAN\_CellBodySpotAvgIntenCh3 | | NeuronalProfilingV42Well:MEDIAN\_CellBodySpotOverlapAreaCh3 | | NeuronalProfilingV42Well:MEDIAN\_CellBodySpotOverlapCountCh3 | | NeuronalProfilingV42Well:MEDIAN\_CellBodySpotTotalAreaCh3 | | NeuronalProfilingV42Well:MEDIAN\_CellBodySpotTotalCountCh3 | | NeuronalProfilingV42Well:MEDIAN\_CellBodySpotTotalIntenCh3 | | NeuronalProfilingV42Well:MEDIAN\_CellBodySpotTotalIntenRatioCh3Ch2 | | NeuronalProfilingV42Well:MEDIAN\_CellBodyTotalIntenCh2 | | NeuronalProfilingV42Well:MEDIAN\_CrossPointAvgCountCh2 | | NeuronalProfilingV42Well:MEDIAN\_CrossPointTotalCountCh2 | | NeuronalProfilingV42Well:MEDIAN\_E1AvgIntenCh3 | | NeuronalProfilingV42Well:MEDIAN\_E1BranchPointAvgCountCh2 | | NeuronalProfilingV42Well:MEDIAN\_E1BranchPointAvgDistFromCellBodyCh2 | | NeuronalProfilingV42Well:MEDIAN\_E1BranchPointCountPerNeuriteLengthCh2 | | NeuronalProfilingV42Well:MEDIAN\_E1BranchPointTotalCountCh2 | | NeuronalProfilingV42Well:MEDIAN\_E1CellBodyAreaCh2 | | NeuronalProfilingV42Well:MEDIAN\_E1CellBodyAvgIntenCh2 | | NeuronalProfilingV42Well:MEDIAN\_E1CellBodyNucAvgIntenCh1 | | NeuronalProfilingV42Well:MEDIAN\_E1CellBodyNucCountCh2 | | NeuronalProfilingV42Well:MEDIAN\_E1CellBodyNucTotalAreaCh1 | | NeuronalProfilingV42Well:MEDIAN\_E1CellBodyNucTotalIntenCh1 | | NeuronalProfilingV42Well:MEDIAN\_E1CellBodyShapeLWRCh2 | | NeuronalProfilingV42Well:MEDIAN\_E1CellBodyShapeP2ACh2 | | NeuronalProfilingV42Well:MEDIAN\_E1CellBodySpot%OverlapAreaCh3 | | NeuronalProfilingV42Well:MEDIAN\_E1CellBodySpot%OverlapCountCh3 | | NeuronalProfilingV42Well:MEDIAN\_E1CellBodySpotAvgIntenCh3 | | NeuronalProfilingV42Well:MEDIAN\_E1CellBodySpotOverlapAreaCh3 | | NeuronalProfilingV42Well:MEDIAN\_E1CellBodySpotOverlapCountCh3 | | NeuronalProfilingV42Well:MEDIAN\_E1CellBodySpotTotalAreaCh3 | | NeuronalProfilingV42Well:MEDIAN\_E1CellBodySpotTotalCountCh3 | | NeuronalProfilingV42Well:MEDIAN\_E1CellBodySpotTotalIntenCh3 | | NeuronalProfilingV42Well:MEDIAN\_E1CellBodySpotTotalIntenRatioCh3Ch2 | | NeuronalProfilingV42Well:MEDIAN\_E1CellBodyTotalIntenCh2 | | NeuronalProfilingV42Well:MEDIAN\_E1CrossPointAvgCountCh2 | | NeuronalProfilingV42Well:MEDIAN\_E1CrossPointTotalCountCh2 | | NeuronalProfilingV42Well:MEDIAN\_E1NeuriteAvgIntenCh2 | | NeuronalProfilingV42Well:MEDIAN\_E1NeuriteAvgLengthCh2 | | NeuronalProfilingV42Well:MEDIAN\_E1NeuriteCriticalValueCh2 | | NeuronalProfilingV42Well:MEDIAN\_E1NeuriteDendriteMaxCh2 | | NeuronalProfilingV42Well:MEDIAN\_E1NeuriteMaxLengthWithBranchesCh2 | | NeuronalProfilingV42Well:MEDIAN\_E1NeuriteMaxLengthWithoutBranchesCh2 | | NeuronalProfilingV42Well:MEDIAN\_E1NeuriteRamificationIndexCh2 | | NeuronalProfilingV42Well:MEDIAN\_E1NeuriteSpot%OverlapAreaCh3 | | NeuronalProfilingV42Well:MEDIAN\_E1NeuriteSpot%OverlapCountCh3 | | NeuronalProfilingV42Well:MEDIAN\_E1NeuriteSpotAvgAreaCh3 | | NeuronalProfilingV42Well:MEDIAN\_E1NeuriteSpotAvgCountCh3 | | NeuronalProfilingV42Well:MEDIAN\_E1NeuriteSpotAvgIntenCh3 | | NeuronalProfilingV42Well:MEDIAN\_E1NeuriteSpotOverlapAreaCh3 | | NeuronalProfilingV42Well:MEDIAN\_E1NeuriteSpotOverlapCountCh3 | | NeuronalProfilingV42Well:MEDIAN\_E1NeuriteSpotTotalAreaCh3 | | NeuronalProfilingV42Well:MEDIAN\_E1NeuriteSpotTotalCountCh3 | | NeuronalProfilingV42Well:MEDIAN\_E1NeuriteSpotTotalIntenCh3 | | NeuronalProfilingV42Well:MEDIAN\_E1NeuriteSpotTotalIntenRatioCh3Ch2 | | NeuronalProfilingV42Well:MEDIAN\_E1NeuriteTotalAreaCh2 | | NeuronalProfilingV42Well:MEDIAN\_E1NeuriteTotalCountCh2 | | NeuronalProfilingV42Well:MEDIAN\_E1NeuriteTotalIntenCh2 | | NeuronalProfilingV42Well:MEDIAN\_E1NeuriteTotalLengthCh2 | | NeuronalProfilingV42Well:MEDIAN\_E1NeuriteVarIntenCh2 | | NeuronalProfilingV42Well:MEDIAN\_E1NeuriteWidthCh2 | | NeuronalProfilingV42Well:MEDIAN\_E1TotalIntenCh3 | | NeuronalProfilingV42Well:MEDIAN\_E2AvgIntenCh3 | | NeuronalProfilingV42Well:MEDIAN\_E2BranchPointAvgCountCh2 | | NeuronalProfilingV42Well:MEDIAN\_E2BranchPointAvgDistFromCellBodyCh2 | | NeuronalProfilingV42Well:MEDIAN\_E2BranchPointCountPerNeuriteLengthCh2 | | NeuronalProfilingV42Well:MEDIAN\_E2BranchPointTotalCountCh2 | | NeuronalProfilingV42Well:MEDIAN\_E2CellBodyAreaCh2 | | NeuronalProfilingV42Well:MEDIAN\_E2CellBodyAvgIntenCh2 | | NeuronalProfilingV42Well:MEDIAN\_E2CellBodyNucAvgIntenCh1 | | NeuronalProfilingV42Well:MEDIAN\_E2CellBodyNucCountCh2 | | NeuronalProfilingV42Well:MEDIAN\_E2CellBodyNucTotalAreaCh1 | | NeuronalProfilingV42Well:MEDIAN\_E2CellBodyNucTotalIntenCh1 | | NeuronalProfilingV42Well:MEDIAN\_E2CellBodyShapeLWRCh2 | | NeuronalProfilingV42Well:MEDIAN\_E2CellBodyShapeP2ACh2 | | NeuronalProfilingV42Well:MEDIAN\_E2CellBodySpot%OverlapAreaCh3 | | NeuronalProfilingV42Well:MEDIAN\_E2CellBodySpot%OverlapCountCh3 | | NeuronalProfilingV42Well:MEDIAN\_E2CellBodySpotAvgIntenCh3 | | NeuronalProfilingV42Well:MEDIAN\_E2CellBodySpotOverlapAreaCh3 | | NeuronalProfilingV42Well:MEDIAN\_E2CellBodySpotOverlapCountCh3 | | NeuronalProfilingV42Well:MEDIAN\_E2CellBodySpotTotalAreaCh3 | | NeuronalProfilingV42Well:MEDIAN\_E2CellBodySpotTotalCountCh3 | | NeuronalProfilingV42Well:MEDIAN\_E2CellBodySpotTotalIntenCh3 | | NeuronalProfilingV42Well:MEDIAN\_E2CellBodySpotTotalIntenRatioCh3Ch2 | | NeuronalProfilingV42Well:MEDIAN\_E2CellBodyTotalIntenCh2 | | NeuronalProfilingV42Well:MEDIAN\_E2CrossPointAvgCountCh2 | | NeuronalProfilingV42Well:MEDIAN\_E2CrossPointTotalCountCh2 | | NeuronalProfilingV42Well:MEDIAN\_E2NeuriteAvgIntenCh2 | | NeuronalProfilingV42Well:MEDIAN\_E2NeuriteAvgLengthCh2 | | NeuronalProfilingV42Well:MEDIAN\_E2NeuriteCriticalValueCh2 | | NeuronalProfilingV42Well:MEDIAN\_E2NeuriteDendriteMaxCh2 | | NeuronalProfilingV42Well:MEDIAN\_E2NeuriteMaxLengthWithBranchesCh2 | | NeuronalProfilingV42Well:MEDIAN\_E2NeuriteMaxLengthWithoutBranchesCh2 | | NeuronalProfilingV42Well:MEDIAN\_E2NeuriteRamificationIndexCh2 | | NeuronalProfilingV42Well:MEDIAN\_E2NeuriteSpot%OverlapAreaCh3 | | NeuronalProfilingV42Well:MEDIAN\_E2NeuriteSpot%OverlapCountCh3 | | NeuronalProfilingV42Well:MEDIAN\_E2NeuriteSpotAvgAreaCh3 | | NeuronalProfilingV42Well:MEDIAN\_E2NeuriteSpotAvgCountCh3 | | NeuronalProfilingV42Well:MEDIAN\_E2NeuriteSpotAvgIntenCh3 | | NeuronalProfilingV42Well:MEDIAN\_E2NeuriteSpotOverlapAreaCh3 | | NeuronalProfilingV42Well:MEDIAN\_E2NeuriteSpotOverlapCountCh3 | | NeuronalProfilingV42Well:MEDIAN\_E2NeuriteSpotTotalAreaCh3 | | NeuronalProfilingV42Well:MEDIAN\_E2NeuriteSpotTotalCountCh3 | | NeuronalProfilingV42Well:MEDIAN\_E2NeuriteSpotTotalIntenCh3 | | NeuronalProfilingV42Well:MEDIAN\_E2NeuriteSpotTotalIntenRatioCh3Ch2 | | NeuronalProfilingV42Well:MEDIAN\_E2NeuriteTotalAreaCh2 | | NeuronalProfilingV42Well:MEDIAN\_E2NeuriteTotalCountCh2 | | NeuronalProfilingV42Well:MEDIAN\_E2NeuriteTotalIntenCh2 | | NeuronalProfilingV42Well:MEDIAN\_E2NeuriteTotalLengthCh2 | | NeuronalProfilingV42Well:MEDIAN\_E2NeuriteVarIntenCh2 | | NeuronalProfilingV42Well:MEDIAN\_E2NeuriteWidthCh2 | | NeuronalProfilingV42Well:MEDIAN\_E2TotalIntenCh3 | | NeuronalProfilingV42Well:MEDIAN\_E3AvgIntenCh3 | | NeuronalProfilingV42Well:MEDIAN\_E3BranchPointAvgCountCh2 | | NeuronalProfilingV42Well:MEDIAN\_E3BranchPointAvgDistFromCellBodyCh2 | | NeuronalProfilingV42Well:MEDIAN\_E3BranchPointCountPerNeuriteLengthCh2 | | NeuronalProfilingV42Well:MEDIAN\_E3BranchPointTotalCountCh2 | | NeuronalProfilingV42Well:MEDIAN\_E3CellBodyAreaCh2 | | NeuronalProfilingV42Well:MEDIAN\_E3CellBodyAvgIntenCh2 | | NeuronalProfilingV42Well:MEDIAN\_E3CellBodyNucAvgIntenCh1 | | NeuronalProfilingV42Well:MEDIAN\_E3CellBodyNucCountCh2 | | NeuronalProfilingV42Well:MEDIAN\_E3CellBodyNucTotalAreaCh1 | | NeuronalProfilingV42Well:MEDIAN\_E3CellBodyNucTotalIntenCh1 | | NeuronalProfilingV42Well:MEDIAN\_E3CellBodyShapeLWRCh2 | | NeuronalProfilingV42Well:MEDIAN\_E3CellBodyShapeP2ACh2 | | NeuronalProfilingV42Well:MEDIAN\_E3CellBodySpot%OverlapAreaCh3 | | NeuronalProfilingV42Well:MEDIAN\_E3CellBodySpot%OverlapCountCh3 | | NeuronalProfilingV42Well:MEDIAN\_E3CellBodySpotAvgIntenCh3 | | NeuronalProfilingV42Well:MEDIAN\_E3CellBodySpotOverlapAreaCh3 | | NeuronalProfilingV42Well:MEDIAN\_E3CellBodySpotOverlapCountCh3 | | NeuronalProfilingV42Well:MEDIAN\_E3CellBodySpotTotalAreaCh3 | | NeuronalProfilingV42Well:MEDIAN\_E3CellBodySpotTotalCountCh3 | | NeuronalProfilingV42Well:MEDIAN\_E3CellBodySpotTotalIntenCh3 | | NeuronalProfilingV42Well:MEDIAN\_E3CellBodySpotTotalIntenRatioCh3Ch2 | | NeuronalProfilingV42Well:MEDIAN\_E3CellBodyTotalIntenCh2 | | NeuronalProfilingV42Well:MEDIAN\_E3CrossPointAvgCountCh2 | | NeuronalProfilingV42Well:MEDIAN\_E3CrossPointTotalCountCh2 | | NeuronalProfilingV42Well:MEDIAN\_E3NeuriteAvgIntenCh2 | | NeuronalProfilingV42Well:MEDIAN\_E3NeuriteAvgLengthCh2 | | NeuronalProfilingV42Well:MEDIAN\_E3NeuriteCriticalValueCh2 | | NeuronalProfilingV42Well:MEDIAN\_E3NeuriteDendriteMaxCh2 | | NeuronalProfilingV42Well:MEDIAN\_E3NeuriteMaxLengthWithBranchesCh2 | | NeuronalProfilingV42Well:MEDIAN\_E3NeuriteMaxLengthWithoutBranchesCh2 | | NeuronalProfilingV42Well:MEDIAN\_E3NeuriteRamificationIndexCh2 | | NeuronalProfilingV42Well:MEDIAN\_E3NeuriteSpot%OverlapAreaCh3 | | NeuronalProfilingV42Well:MEDIAN\_E3NeuriteSpot%OverlapCountCh3 | | NeuronalProfilingV42Well:MEDIAN\_E3NeuriteSpotAvgAreaCh3 | | NeuronalProfilingV42Well:MEDIAN\_E3NeuriteSpotAvgCountCh3 | | NeuronalProfilingV42Well:MEDIAN\_E3NeuriteSpotAvgIntenCh3 | | NeuronalProfilingV42Well:MEDIAN\_E3NeuriteSpotOverlapAreaCh3 | | NeuronalProfilingV42Well:MEDIAN\_E3NeuriteSpotOverlapCountCh3 | | NeuronalProfilingV42Well:MEDIAN\_E3NeuriteSpotTotalAreaCh3 | | NeuronalProfilingV42Well:MEDIAN\_E3NeuriteSpotTotalCountCh3 | | NeuronalProfilingV42Well:MEDIAN\_E3NeuriteSpotTotalIntenCh3 | | NeuronalProfilingV42Well:MEDIAN\_E3NeuriteSpotTotalIntenRatioCh3Ch2 | | NeuronalProfilingV42Well:MEDIAN\_E3NeuriteTotalAreaCh2 | | NeuronalProfilingV42Well:MEDIAN\_E3NeuriteTotalCountCh2 | | NeuronalProfilingV42Well:MEDIAN\_E3NeuriteTotalIntenCh2 | | NeuronalProfilingV42Well:MEDIAN\_E3NeuriteTotalLengthCh2 | | NeuronalProfilingV42Well:MEDIAN\_E3NeuriteVarIntenCh2 | | NeuronalProfilingV42Well:MEDIAN\_E3NeuriteWidthCh2 | | NeuronalProfilingV42Well:MEDIAN\_E3TotalIntenCh3 | | NeuronalProfilingV42Well:MEDIAN\_NeuriteAvgIntenCh2 | | NeuronalProfilingV42Well:MEDIAN\_NeuriteAvgLengthCh2 | | NeuronalProfilingV42Well:MEDIAN\_NeuriteCriticalValueCh2 | | NeuronalProfilingV42Well:MEDIAN\_NeuriteDendriteMaxCh2 | | NeuronalProfilingV42Well:MEDIAN\_NeuriteMaxLengthWithBranchesCh2 | | NeuronalProfilingV42Well:MEDIAN\_NeuriteMaxLengthWithoutBranchesCh2 | | NeuronalProfilingV42Well:MEDIAN\_NeuriteRamificationIndexCh2 | | NeuronalProfilingV42Well:MEDIAN\_NeuriteSpot%OverlapAreaCh3 | | NeuronalProfilingV42Well:MEDIAN\_NeuriteSpot%OverlapCountCh3 | | NeuronalProfilingV42Well:MEDIAN\_NeuriteSpotAvgAreaCh3 | | NeuronalProfilingV42Well:MEDIAN\_NeuriteSpotAvgCountCh3 | | NeuronalProfilingV42Well:MEDIAN\_NeuriteSpotAvgIntenCh3 | | NeuronalProfilingV42Well:MEDIAN\_NeuriteSpotOverlapAreaCh3 | | NeuronalProfilingV42Well:MEDIAN\_NeuriteSpotOverlapCountCh3 | | NeuronalProfilingV42Well:MEDIAN\_NeuriteSpotTotalAreaCh3 | | NeuronalProfilingV42Well:MEDIAN\_NeuriteSpotTotalCountCh3 | | NeuronalProfilingV42Well:MEDIAN\_NeuriteSpotTotalIntenCh3 | | NeuronalProfilingV42Well:MEDIAN\_NeuriteSpotTotalIntenRatioCh3Ch2 | | NeuronalProfilingV42Well:MEDIAN\_NeuriteTotalAreaCh2 | | NeuronalProfilingV42Well:MEDIAN\_NeuriteTotalCountCh2 | | NeuronalProfilingV42Well:MEDIAN\_NeuriteTotalIntenCh2 | | NeuronalProfilingV42Well:MEDIAN\_NeuriteTotalLengthCh2 | | NeuronalProfilingV42Well:MEDIAN\_NeuriteVarIntenCh2 | | NeuronalProfilingV42Well:MEDIAN\_NeuriteWidthCh2 | | NeuronalProfilingV42Well:MEDIAN\_TotalIntenCh3 | | NeuronalProfilingV42Well:NA\_NeuriteAvgIntenPerWellCh2 | | NeuronalProfilingV42Well:NA\_NeuriteSpot%OverlapAreaPerWellCh3 | | NeuronalProfilingV42Well:NA\_NeuriteSpot%OverlapCountPerWellCh3 | | NeuronalProfilingV42Well:NA\_NeuriteSpotAvgIntenPerNeuriteLengthCh3 | | NeuronalProfilingV42Well:NA\_NeuriteSpotAvgIntenPerWellCh3 | | NeuronalProfilingV42Well:NA\_NeuriteSpotOverlapTotalAreaPerNeuriteLengthCh3 | | NeuronalProfilingV42Well:NA\_NeuriteSpotOverlapTotalAreaPerWellCh3 | | NeuronalProfilingV42Well:NA\_NeuriteSpotOverlapTotalCountPerNeuriteLengthCh3 | | NeuronalProfilingV42Well:NA\_NeuriteSpotOverlapTotalCountPerWellCh3 | | NeuronalProfilingV42Well:NA\_NeuriteSpotTotalAreaPerNeuriteLengthCh3 | | NeuronalProfilingV42Well:NA\_NeuriteSpotTotalAreaPerWellCh3 | | NeuronalProfilingV42Well:NA\_NeuriteSpotTotalCountPerNeuriteLengthCh3 | | NeuronalProfilingV42Well:NA\_NeuriteSpotTotalCountPerWellCh3 | | NeuronalProfilingV42Well:NA\_NeuriteSpotTotalIntenPerNeuriteLengthCh3 | | NeuronalProfilingV42Well:NA\_NeuriteSpotTotalIntenPerWellCh3 | | NeuronalProfilingV42Well:NA\_NeuriteTotalIntenPerWellCh2 | | NeuronalProfilingV42Well:NA\_NeuriteTotalLengthPerWellCh2 | | NeuronalProfilingV42Well:NeuriteAvgIntenPerFieldCh2 | | NeuronalProfilingV42Well:NeuriteSpotAvgIntenPerFieldCh3 | | NeuronalProfilingV42Well:NeuriteSpotTotalAreaPerFieldCh3 | | NeuronalProfilingV42Well:NeuriteSpotTotalAreaPerNeuriteCh3 | | NeuronalProfilingV42Well:NeuriteSpotTotalAreaPerNeuriteLengthCh3 | | NeuronalProfilingV42Well:NeuriteSpotTotalAreaPerNeuronCh3 | | NeuronalProfilingV42Well:NeuriteSpotTotalAreaPerWellCh3 | | NeuronalProfilingV42Well:NeuriteSpotTotalCountPerFieldCh3 | | NeuronalProfilingV42Well:NeuriteSpotTotalCountPerNeuriteCh3 | | NeuronalProfilingV42Well:NeuriteSpotTotalCountPerNeuriteLengthCh3 | | NeuronalProfilingV42Well:NeuriteSpotTotalCountPerNeuronCh3 | | NeuronalProfilingV42Well:NeuriteSpotTotalCountPerWellCh3 | | NeuronalProfilingV42Well:NeuriteSpotTotalIntenPerFieldCh3 | | NeuronalProfilingV42Well:NeuriteSpotTotalIntenPerNeuriteCh3 | | NeuronalProfilingV42Well:NeuriteSpotTotalIntenPerNeuriteLengthCh3 | | NeuronalProfilingV42Well:NeuriteSpotTotalIntenPerNeuronCh3 | | NeuronalProfilingV42Well:NeuriteSpotTotalIntenPerWellCh3 | | NeuronalProfilingV42Well:NeuriteTotalCountPerFieldCh2 | | NeuronalProfilingV42Well:NeuriteTotalCountPerNeuronCh2 | | NeuronalProfilingV42Well:NeuriteTotalCountPerWellCh2 | | NeuronalProfilingV42Well:NeuriteTotalIntenPerFieldCh2 | | NeuronalProfilingV42Well:NeuriteTotalIntenPerWellCh2 | | NeuronalProfilingV42Well:NeuriteTotalLengthPerFieldCh2 | | NeuronalProfilingV42Well:NeuriteTotalLengthPerNeuriteCh2 | | NeuronalProfilingV42Well:NeuriteTotalLengthPerNeuronCh2 | | NeuronalProfilingV42Well:NeuriteTotalLengthPerWellCh2 | | NeuronalProfilingV42Well:NeuriteVarIntenPerFieldCh2 | | NeuronalProfilingV42Well:NeuronalNucleusCount | | NeuronalProfilingV42Well:NeuronNucleusRatio | | NeuronalProfilingV42Well:NonNeuronalNucleusCount | | NeuronalProfilingV42Well:SD\_AvgIntenCh3 | | NeuronalProfilingV42Well:SD\_BranchPointAvgCountCh2 | | NeuronalProfilingV42Well:SD\_BranchPointAvgDistFromCellBodyCh2 | | NeuronalProfilingV42Well:SD\_BranchPointCountPerNeuriteLengthCh2 | | NeuronalProfilingV42Well:SD\_BranchPointTotalCountCh2 | | NeuronalProfilingV42Well:SD\_CellBodyAreaCh2 | | NeuronalProfilingV42Well:SD\_CellBodyAvgIntenCh2 | | NeuronalProfilingV42Well:SD\_CellBodyNucAvgIntenCh1 | | NeuronalProfilingV42Well:SD\_CellBodyNucCountCh2 | | NeuronalProfilingV42Well:SD\_CellBodyNucTotalAreaCh1 | | NeuronalProfilingV42Well:SD\_CellBodyNucTotalIntenCh1 | | NeuronalProfilingV42Well:SD\_CellBodyShapeLWRCh2 | | NeuronalProfilingV42Well:SD\_CellBodyShapeP2ACh2 | | NeuronalProfilingV42Well:SD\_CellBodySpot%OverlapAreaCh3 | | NeuronalProfilingV42Well:SD\_CellBodySpot%OverlapCountCh3 | | NeuronalProfilingV42Well:SD\_CellBodySpotAvgIntenCh3 | | NeuronalProfilingV42Well:SD\_CellBodySpotOverlapAreaCh3 | | NeuronalProfilingV42Well:SD\_CellBodySpotOverlapCountCh3 | | NeuronalProfilingV42Well:SD\_CellBodySpotTotalAreaCh3 | | NeuronalProfilingV42Well:SD\_CellBodySpotTotalCountCh3 | | NeuronalProfilingV42Well:SD\_CellBodySpotTotalIntenCh3 | | NeuronalProfilingV42Well:SD\_CellBodySpotTotalIntenRatioCh3Ch2 | | NeuronalProfilingV42Well:SD\_CellBodyTotalIntenCh2 | | NeuronalProfilingV42Well:SD\_CrossPointAvgCountCh2 | | NeuronalProfilingV42Well:SD\_CrossPointTotalCountCh2 | | NeuronalProfilingV42Well:SD\_E1AvgIntenCh3 | | NeuronalProfilingV42Well:SD\_E1BranchPointAvgCountCh2 | | NeuronalProfilingV42Well:SD\_E1BranchPointAvgDistFromCellBodyCh2 | | NeuronalProfilingV42Well:SD\_E1BranchPointCountPerNeuriteLengthCh2 | | NeuronalProfilingV42Well:SD\_E1BranchPointTotalCountCh2 | | NeuronalProfilingV42Well:SD\_E1CellBodyAreaCh2 | | NeuronalProfilingV42Well:SD\_E1CellBodyAvgIntenCh2 | | NeuronalProfilingV42Well:SD\_E1CellBodyNucAvgIntenCh1 | | NeuronalProfilingV42Well:SD\_E1CellBodyNucCountCh2 | | NeuronalProfilingV42Well:SD\_E1CellBodyNucTotalAreaCh1 | | NeuronalProfilingV42Well:SD\_E1CellBodyNucTotalIntenCh1 | | NeuronalProfilingV42Well:SD\_E1CellBodyShapeLWRCh2 | | NeuronalProfilingV42Well:SD\_E1CellBodyShapeP2ACh2 | | NeuronalProfilingV42Well:SD\_E1CellBodySpot%OverlapAreaCh3 | | NeuronalProfilingV42Well:SD\_E1CellBodySpot%OverlapCountCh3 | | NeuronalProfilingV42Well:SD\_E1CellBodySpotAvgIntenCh3 | | NeuronalProfilingV42Well:SD\_E1CellBodySpotOverlapAreaCh3 | | NeuronalProfilingV42Well:SD\_E1CellBodySpotOverlapCountCh3 | | NeuronalProfilingV42Well:SD\_E1CellBodySpotTotalAreaCh3 | | NeuronalProfilingV42Well:SD\_E1CellBodySpotTotalCountCh3 | | NeuronalProfilingV42Well:SD\_E1CellBodySpotTotalIntenCh3 | | NeuronalProfilingV42Well:SD\_E1CellBodySpotTotalIntenRatioCh3Ch2 | | NeuronalProfilingV42Well:SD\_E1CellBodyTotalIntenCh2 | | NeuronalProfilingV42Well:SD\_E1CrossPointAvgCountCh2 | | NeuronalProfilingV42Well:SD\_E1CrossPointTotalCountCh2 | | NeuronalProfilingV42Well:SD\_E1NeuriteAvgIntenCh2 | | NeuronalProfilingV42Well:SD\_E1NeuriteAvgLengthCh2 | | NeuronalProfilingV42Well:SD\_E1NeuriteCriticalValueCh2 | | NeuronalProfilingV42Well:SD\_E1NeuriteDendriteMaxCh2 | | NeuronalProfilingV42Well:SD\_E1NeuriteMaxLengthWithBranchesCh2 | | NeuronalProfilingV42Well:SD\_E1NeuriteMaxLengthWithoutBranchesCh2 | | NeuronalProfilingV42Well:SD\_E1NeuriteRamificationIndexCh2 | | NeuronalProfilingV42Well:SD\_E1NeuriteSpot%OverlapAreaCh3 | | NeuronalProfilingV42Well:SD\_E1NeuriteSpot%OverlapCountCh3 | | NeuronalProfilingV42Well:SD\_E1NeuriteSpotAvgAreaCh3 | | NeuronalProfilingV42Well:SD\_E1NeuriteSpotAvgCountCh3 | | NeuronalProfilingV42Well:SD\_E1NeuriteSpotAvgIntenCh3 | | NeuronalProfilingV42Well:SD\_E1NeuriteSpotOverlapAreaCh3 | | NeuronalProfilingV42Well:SD\_E1NeuriteSpotOverlapCountCh3 | | NeuronalProfilingV42Well:SD\_E1NeuriteSpotTotalAreaCh3 | | NeuronalProfilingV42Well:SD\_E1NeuriteSpotTotalCountCh3 | | NeuronalProfilingV42Well:SD\_E1NeuriteSpotTotalIntenCh3 | | NeuronalProfilingV42Well:SD\_E1NeuriteSpotTotalIntenRatioCh3Ch2 | | NeuronalProfilingV42Well:SD\_E1NeuriteTotalAreaCh2 | | NeuronalProfilingV42Well:SD\_E1NeuriteTotalCountCh2 | | NeuronalProfilingV42Well:SD\_E1NeuriteTotalIntenCh2 | | NeuronalProfilingV42Well:SD\_E1NeuriteTotalLengthCh2 | | NeuronalProfilingV42Well:SD\_E1NeuriteVarIntenCh2 | | NeuronalProfilingV42Well:SD\_E1NeuriteWidthCh2 | | NeuronalProfilingV42Well:SD\_E1TotalIntenCh3 | | NeuronalProfilingV42Well:SD\_E2AvgIntenCh3 | | NeuronalProfilingV42Well:SD\_E2BranchPointAvgCountCh2 | | NeuronalProfilingV42Well:SD\_E2BranchPointAvgDistFromCellBodyCh2 | | NeuronalProfilingV42Well:SD\_E2BranchPointCountPerNeuriteLengthCh2 | | NeuronalProfilingV42Well:SD\_E2BranchPointTotalCountCh2 | | NeuronalProfilingV42Well:SD\_E2CellBodyAreaCh2 | | NeuronalProfilingV42Well:SD\_E2CellBodyAvgIntenCh2 | | NeuronalProfilingV42Well:SD\_E2CellBodyNucAvgIntenCh1 | | NeuronalProfilingV42Well:SD\_E2CellBodyNucCountCh2 | | NeuronalProfilingV42Well:SD\_E2CellBodyNucTotalAreaCh1 | | NeuronalProfilingV42Well:SD\_E2CellBodyNucTotalIntenCh1 | | NeuronalProfilingV42Well:SD\_E2CellBodyShapeLWRCh2 | | NeuronalProfilingV42Well:SD\_E2CellBodyShapeP2ACh2 | | NeuronalProfilingV42Well:SD\_E2CellBodySpot%OverlapAreaCh3 | | NeuronalProfilingV42Well:SD\_E2CellBodySpot%OverlapCountCh3 | | NeuronalProfilingV42Well:SD\_E2CellBodySpotAvgIntenCh3 | | NeuronalProfilingV42Well:SD\_E2CellBodySpotOverlapAreaCh3 | | NeuronalProfilingV42Well:SD\_E2CellBodySpotOverlapCountCh3 | | NeuronalProfilingV42Well:SD\_E2CellBodySpotTotalAreaCh3 | | NeuronalProfilingV42Well:SD\_E2CellBodySpotTotalCountCh3 | | NeuronalProfilingV42Well:SD\_E2CellBodySpotTotalIntenCh3 | | NeuronalProfilingV42Well:SD\_E2CellBodySpotTotalIntenRatioCh3Ch2 | | NeuronalProfilingV42Well:SD\_E2CellBodyTotalIntenCh2 | | NeuronalProfilingV42Well:SD\_E2CrossPointAvgCountCh2 | | NeuronalProfilingV42Well:SD\_E2CrossPointTotalCountCh2 | | NeuronalProfilingV42Well:SD\_E2NeuriteAvgIntenCh2 | | NeuronalProfilingV42Well:SD\_E2NeuriteAvgLengthCh2 | | NeuronalProfilingV42Well:SD\_E2NeuriteCriticalValueCh2 | | NeuronalProfilingV42Well:SD\_E2NeuriteDendriteMaxCh2 | | NeuronalProfilingV42Well:SD\_E2NeuriteMaxLengthWithBranchesCh2 | | NeuronalProfilingV42Well:SD\_E2NeuriteMaxLengthWithoutBranchesCh2 | | NeuronalProfilingV42Well:SD\_E2NeuriteRamificationIndexCh2 | | NeuronalProfilingV42Well:SD\_E2NeuriteSpot%OverlapAreaCh3 | | NeuronalProfilingV42Well:SD\_E2NeuriteSpot%OverlapCountCh3 | | NeuronalProfilingV42Well:SD\_E2NeuriteSpotAvgAreaCh3 | | NeuronalProfilingV42Well:SD\_E2NeuriteSpotAvgCountCh3 | | NeuronalProfilingV42Well:SD\_E2NeuriteSpotAvgIntenCh3 | | NeuronalProfilingV42Well:SD\_E2NeuriteSpotOverlapAreaCh3 | | NeuronalProfilingV42Well:SD\_E2NeuriteSpotOverlapCountCh3 | | NeuronalProfilingV42Well:SD\_E2NeuriteSpotTotalAreaCh3 | | NeuronalProfilingV42Well:SD\_E2NeuriteSpotTotalCountCh3 | | NeuronalProfilingV42Well:SD\_E2NeuriteSpotTotalIntenCh3 | | NeuronalProfilingV42Well:SD\_E2NeuriteSpotTotalIntenRatioCh3Ch2 | | NeuronalProfilingV42Well:SD\_E2NeuriteTotalAreaCh2 | | NeuronalProfilingV42Well:SD\_E2NeuriteTotalCountCh2 | | NeuronalProfilingV42Well:SD\_E2NeuriteTotalIntenCh2 | | NeuronalProfilingV42Well:SD\_E2NeuriteTotalLengthCh2 | | NeuronalProfilingV42Well:SD\_E2NeuriteVarIntenCh2 | | NeuronalProfilingV42Well:SD\_E2NeuriteWidthCh2 | | NeuronalProfilingV42Well:SD\_E2TotalIntenCh3 | | NeuronalProfilingV42Well:SD\_E3AvgIntenCh3 | | NeuronalProfilingV42Well:SD\_E3BranchPointAvgCountCh2 | | NeuronalProfilingV42Well:SD\_E3BranchPointAvgDistFromCellBodyCh2 | | NeuronalProfilingV42Well:SD\_E3BranchPointCountPerNeuriteLengthCh2 | | NeuronalProfilingV42Well:SD\_E3BranchPointTotalCountCh2 | | NeuronalProfilingV42Well:SD\_E3CellBodyAreaCh2 | | NeuronalProfilingV42Well:SD\_E3CellBodyAvgIntenCh2 | | NeuronalProfilingV42Well:SD\_E3CellBodyNucAvgIntenCh1 | | NeuronalProfilingV42Well:SD\_E3CellBodyNucCountCh2 | | NeuronalProfilingV42Well:SD\_E3CellBodyNucTotalAreaCh1 | | NeuronalProfilingV42Well:SD\_E3CellBodyNucTotalIntenCh1 | | NeuronalProfilingV42Well:SD\_E3CellBodyShapeLWRCh2 | | NeuronalProfilingV42Well:SD\_E3CellBodyShapeP2ACh2 | | NeuronalProfilingV42Well:SD\_E3CellBodySpot%OverlapAreaCh3 | | NeuronalProfilingV42Well:SD\_E3CellBodySpot%OverlapCountCh3 | | NeuronalProfilingV42Well:SD\_E3CellBodySpotAvgIntenCh3 | | NeuronalProfilingV42Well:SD\_E3CellBodySpotOverlapAreaCh3 | | NeuronalProfilingV42Well:SD\_E3CellBodySpotOverlapCountCh3 | | NeuronalProfilingV42Well:SD\_E3CellBodySpotTotalAreaCh3 | | NeuronalProfilingV42Well:SD\_E3CellBodySpotTotalCountCh3 | | NeuronalProfilingV42Well:SD\_E3CellBodySpotTotalIntenCh3 | | NeuronalProfilingV42Well:SD\_E3CellBodySpotTotalIntenRatioCh3Ch2 | | NeuronalProfilingV42Well:SD\_E3CellBodyTotalIntenCh2 | | NeuronalProfilingV42Well:SD\_E3CrossPointAvgCountCh2 | | NeuronalProfilingV42Well:SD\_E3CrossPointTotalCountCh2 | | NeuronalProfilingV42Well:SD\_E3NeuriteAvgIntenCh2 | | NeuronalProfilingV42Well:SD\_E3NeuriteAvgLengthCh2 | | NeuronalProfilingV42Well:SD\_E3NeuriteCriticalValueCh2 | | NeuronalProfilingV42Well:SD\_E3NeuriteDendriteMaxCh2 | | NeuronalProfilingV42Well:SD\_E3NeuriteMaxLengthWithBranchesCh2 | | NeuronalProfilingV42Well:SD\_E3NeuriteMaxLengthWithoutBranchesCh2 | | NeuronalProfilingV42Well:SD\_E3NeuriteRamificationIndexCh2 | | NeuronalProfilingV42Well:SD\_E3NeuriteSpot%OverlapAreaCh3 | | NeuronalProfilingV42Well:SD\_E3NeuriteSpot%OverlapCountCh3 | | NeuronalProfilingV42Well:SD\_E3NeuriteSpotAvgAreaCh3 | | NeuronalProfilingV42Well:SD\_E3NeuriteSpotAvgCountCh3 | | NeuronalProfilingV42Well:SD\_E3NeuriteSpotAvgIntenCh3 | | NeuronalProfilingV42Well:SD\_E3NeuriteSpotOverlapAreaCh3 | | NeuronalProfilingV42Well:SD\_E3NeuriteSpotOverlapCountCh3 | | NeuronalProfilingV42Well:SD\_E3NeuriteSpotTotalAreaCh3 | | NeuronalProfilingV42Well:SD\_E3NeuriteSpotTotalCountCh3 | | NeuronalProfilingV42Well:SD\_E3NeuriteSpotTotalIntenCh3 | | NeuronalProfilingV42Well:SD\_E3NeuriteSpotTotalIntenRatioCh3Ch2 | | NeuronalProfilingV42Well:SD\_E3NeuriteTotalAreaCh2 | | NeuronalProfilingV42Well:SD\_E3NeuriteTotalCountCh2 | | NeuronalProfilingV42Well:SD\_E3NeuriteTotalIntenCh2 | | NeuronalProfilingV42Well:SD\_E3NeuriteTotalLengthCh2 | | NeuronalProfilingV42Well:SD\_E3NeuriteVarIntenCh2 | | NeuronalProfilingV42Well:SD\_E3NeuriteWidthCh2 | | NeuronalProfilingV42Well:SD\_E3TotalIntenCh3 | | NeuronalProfilingV42Well:SD\_NeuriteAvgIntenCh2 | | NeuronalProfilingV42Well:SD\_NeuriteAvgLengthCh2 | | NeuronalProfilingV42Well:SD\_NeuriteCriticalValueCh2 | | NeuronalProfilingV42Well:SD\_NeuriteDendriteMaxCh2 | | NeuronalProfilingV42Well:SD\_NeuriteMaxLengthWithBranchesCh2 | | NeuronalProfilingV42Well:SD\_NeuriteMaxLengthWithoutBranchesCh2 | | NeuronalProfilingV42Well:SD\_NeuriteRamificationIndexCh2 | | NeuronalProfilingV42Well:SD\_NeuriteSpot%OverlapAreaCh3 | | NeuronalProfilingV42Well:SD\_NeuriteSpot%OverlapCountCh3 | | NeuronalProfilingV42Well:SD\_NeuriteSpotAvgAreaCh3 | | NeuronalProfilingV42Well:SD\_NeuriteSpotAvgCountCh3 | | NeuronalProfilingV42Well:SD\_NeuriteSpotAvgIntenCh3 | | NeuronalProfilingV42Well:SD\_NeuriteSpotOverlapAreaCh3 | | NeuronalProfilingV42Well:SD\_NeuriteSpotOverlapCountCh3 | | NeuronalProfilingV42Well:SD\_NeuriteSpotTotalAreaCh3 | | NeuronalProfilingV42Well:SD\_NeuriteSpotTotalCountCh3 | | NeuronalProfilingV42Well:SD\_NeuriteSpotTotalIntenCh3 | | NeuronalProfilingV42Well:SD\_NeuriteSpotTotalIntenRatioCh3Ch2 | | NeuronalProfilingV42Well:SD\_NeuriteTotalAreaCh2 | | NeuronalProfilingV42Well:SD\_NeuriteTotalCountCh2 | | NeuronalProfilingV42Well:SD\_NeuriteTotalIntenCh2 | | NeuronalProfilingV42Well:SD\_NeuriteTotalLengthCh2 | | NeuronalProfilingV42Well:SD\_NeuriteVarIntenCh2 | | NeuronalProfilingV42Well:SD\_NeuriteWidthCh2 | | NeuronalProfilingV42Well:SD\_TotalIntenCh3 | | NeuronalProfilingV42Well:SE\_AvgIntenCh3 | | NeuronalProfilingV42Well:SE\_BranchPointAvgCountCh2 | | NeuronalProfilingV42Well:SE\_BranchPointAvgDistFromCellBodyCh2 | | NeuronalProfilingV42Well:SE\_BranchPointCountPerNeuriteLengthCh2 | | NeuronalProfilingV42Well:SE\_BranchPointTotalCountCh2 | | NeuronalProfilingV42Well:SE\_CellBodyAreaCh2 | | NeuronalProfilingV42Well:SE\_CellBodyAvgIntenCh2 | | NeuronalProfilingV42Well:SE\_CellBodyNucAvgIntenCh1 | | NeuronalProfilingV42Well:SE\_CellBodyNucCountCh2 | | NeuronalProfilingV42Well:SE\_CellBodyNucTotalAreaCh1 | | NeuronalProfilingV42Well:SE\_CellBodyNucTotalIntenCh1 | | NeuronalProfilingV42Well:SE\_CellBodyShapeLWRCh2 | | NeuronalProfilingV42Well:SE\_CellBodyShapeP2ACh2 | | NeuronalProfilingV42Well:SE\_CellBodySpot%OverlapAreaCh3 | | NeuronalProfilingV42Well:SE\_CellBodySpot%OverlapCountCh3 | | NeuronalProfilingV42Well:SE\_CellBodySpotAvgIntenCh3 | | NeuronalProfilingV42Well:SE\_CellBodySpotOverlapAreaCh3 | | NeuronalProfilingV42Well:SE\_CellBodySpotOverlapCountCh3 | | NeuronalProfilingV42Well:SE\_CellBodySpotTotalAreaCh3 | | NeuronalProfilingV42Well:SE\_CellBodySpotTotalCountCh3 | | NeuronalProfilingV42Well:SE\_CellBodySpotTotalIntenCh3 | | NeuronalProfilingV42Well:SE\_CellBodySpotTotalIntenRatioCh3Ch2 | | NeuronalProfilingV42Well:SE\_CellBodyTotalIntenCh2 | | NeuronalProfilingV42Well:SE\_CrossPointAvgCountCh2 | | NeuronalProfilingV42Well:SE\_CrossPointTotalCountCh2 | | NeuronalProfilingV42Well:SE\_E1AvgIntenCh3 | | NeuronalProfilingV42Well:SE\_E1BranchPointAvgCountCh2 | | NeuronalProfilingV42Well:SE\_E1BranchPointAvgDistFromCellBodyCh2 | | NeuronalProfilingV42Well:SE\_E1BranchPointCountPerNeuriteLengthCh2 | | NeuronalProfilingV42Well:SE\_E1BranchPointTotalCountCh2 | | NeuronalProfilingV42Well:SE\_E1CellBodyAreaCh2 | | NeuronalProfilingV42Well:SE\_E1CellBodyAvgIntenCh2 | | NeuronalProfilingV42Well:SE\_E1CellBodyNucAvgIntenCh1 | | NeuronalProfilingV42Well:SE\_E1CellBodyNucCountCh2 | | NeuronalProfilingV42Well:SE\_E1CellBodyNucTotalAreaCh1 | | NeuronalProfilingV42Well:SE\_E1CellBodyNucTotalIntenCh1 | | NeuronalProfilingV42Well:SE\_E1CellBodyShapeLWRCh2 | | NeuronalProfilingV42Well:SE\_E1CellBodyShapeP2ACh2 | | NeuronalProfilingV42Well:SE\_E1CellBodySpot%OverlapAreaCh3 | | NeuronalProfilingV42Well:SE\_E1CellBodySpot%OverlapCountCh3 | | NeuronalProfilingV42Well:SE\_E1CellBodySpotAvgIntenCh3 | | NeuronalProfilingV42Well:SE\_E1CellBodySpotOverlapAreaCh3 | | NeuronalProfilingV42Well:SE\_E1CellBodySpotOverlapCountCh3 | | NeuronalProfilingV42Well:SE\_E1CellBodySpotTotalAreaCh3 | | NeuronalProfilingV42Well:SE\_E1CellBodySpotTotalCountCh3 | | NeuronalProfilingV42Well:SE\_E1CellBodySpotTotalIntenCh3 | | NeuronalProfilingV42Well:SE\_E1CellBodySpotTotalIntenRatioCh3Ch2 | | NeuronalProfilingV42Well:SE\_E1CellBodyTotalIntenCh2 | | NeuronalProfilingV42Well:SE\_E1CrossPointAvgCountCh2 | | NeuronalProfilingV42Well:SE\_E1CrossPointTotalCountCh2 | | NeuronalProfilingV42Well:SE\_E1NeuriteAvgIntenCh2 | | NeuronalProfilingV42Well:SE\_E1NeuriteAvgLengthCh2 | | NeuronalProfilingV42Well:SE\_E1NeuriteCriticalValueCh2 | | NeuronalProfilingV42Well:SE\_E1NeuriteDendriteMaxCh2 | | NeuronalProfilingV42Well:SE\_E1NeuriteMaxLengthWithBranchesCh2 | | NeuronalProfilingV42Well:SE\_E1NeuriteMaxLengthWithoutBranchesCh2 | | NeuronalProfilingV42Well:SE\_E1NeuriteRamificationIndexCh2 | | NeuronalProfilingV42Well:SE\_E1NeuriteSpot%OverlapAreaCh3 | | NeuronalProfilingV42Well:SE\_E1NeuriteSpot%OverlapCountCh3 | | NeuronalProfilingV42Well:SE\_E1NeuriteSpotAvgAreaCh3 | | NeuronalProfilingV42Well:SE\_E1NeuriteSpotAvgCountCh3 | | NeuronalProfilingV42Well:SE\_E1NeuriteSpotAvgIntenCh3 | | NeuronalProfilingV42Well:SE\_E1NeuriteSpotOverlapAreaCh3 | | NeuronalProfilingV42Well:SE\_E1NeuriteSpotOverlapCountCh3 | | NeuronalProfilingV42Well:SE\_E1NeuriteSpotTotalAreaCh3 | | NeuronalProfilingV42Well:SE\_E1NeuriteSpotTotalCountCh3 | | NeuronalProfilingV42Well:SE\_E1NeuriteSpotTotalIntenCh3 | | NeuronalProfilingV42Well:SE\_E1NeuriteSpotTotalIntenRatioCh3Ch2 | | NeuronalProfilingV42Well:SE\_E1NeuriteTotalAreaCh2 | | NeuronalProfilingV42Well:SE\_E1NeuriteTotalCountCh2 | | NeuronalProfilingV42Well:SE\_E1NeuriteTotalIntenCh2 | | NeuronalProfilingV42Well:SE\_E1NeuriteTotalLengthCh2 | | NeuronalProfilingV42Well:SE\_E1NeuriteVarIntenCh2 | | NeuronalProfilingV42Well:SE\_E1NeuriteWidthCh2 | | NeuronalProfilingV42Well:SE\_E1TotalIntenCh3 | | NeuronalProfilingV42Well:SE\_E2AvgIntenCh3 | | NeuronalProfilingV42Well:SE\_E2BranchPointAvgCountCh2 | | NeuronalProfilingV42Well:SE\_E2BranchPointAvgDistFromCellBodyCh2 | | NeuronalProfilingV42Well:SE\_E2BranchPointCountPerNeuriteLengthCh2 | | NeuronalProfilingV42Well:SE\_E2BranchPointTotalCountCh2 | | NeuronalProfilingV42Well:SE\_E2CellBodyAreaCh2 | | NeuronalProfilingV42Well:SE\_E2CellBodyAvgIntenCh2 | | NeuronalProfilingV42Well:SE\_E2CellBodyNucAvgIntenCh1 | | NeuronalProfilingV42Well:SE\_E2CellBodyNucCountCh2 | | NeuronalProfilingV42Well:SE\_E2CellBodyNucTotalAreaCh1 | | NeuronalProfilingV42Well:SE\_E2CellBodyNucTotalIntenCh1 | | NeuronalProfilingV42Well:SE\_E2CellBodyShapeLWRCh2 | | NeuronalProfilingV42Well:SE\_E2CellBodyShapeP2ACh2 | | NeuronalProfilingV42Well:SE\_E2CellBodySpot%OverlapAreaCh3 | | NeuronalProfilingV42Well:SE\_E2CellBodySpot%OverlapCountCh3 | | NeuronalProfilingV42Well:SE\_E2CellBodySpotAvgIntenCh3 | | NeuronalProfilingV42Well:SE\_E2CellBodySpotOverlapAreaCh3 | | NeuronalProfilingV42Well:SE\_E2CellBodySpotOverlapCountCh3 | | NeuronalProfilingV42Well:SE\_E2CellBodySpotTotalAreaCh3 | | NeuronalProfilingV42Well:SE\_E2CellBodySpotTotalCountCh3 | | NeuronalProfilingV42Well:SE\_E2CellBodySpotTotalIntenCh3 | | NeuronalProfilingV42Well:SE\_E2CellBodySpotTotalIntenRatioCh3Ch2 | | NeuronalProfilingV42Well:SE\_E2CellBodyTotalIntenCh2 | | NeuronalProfilingV42Well:SE\_E2CrossPointAvgCountCh2 | | NeuronalProfilingV42Well:SE\_E2CrossPointTotalCountCh2 | | NeuronalProfilingV42Well:SE\_E2NeuriteAvgIntenCh2 | | NeuronalProfilingV42Well:SE\_E2NeuriteAvgLengthCh2 | | NeuronalProfilingV42Well:SE\_E2NeuriteCriticalValueCh2 | | NeuronalProfilingV42Well:SE\_E2NeuriteDendriteMaxCh2 | | NeuronalProfilingV42Well:SE\_E2NeuriteMaxLengthWithBranchesCh2 | | NeuronalProfilingV42Well:SE\_E2NeuriteMaxLengthWithoutBranchesCh2 | | NeuronalProfilingV42Well:SE\_E2NeuriteRamificationIndexCh2 | | NeuronalProfilingV42Well:SE\_E2NeuriteSpot%OverlapAreaCh3 | | NeuronalProfilingV42Well:SE\_E2NeuriteSpot%OverlapCountCh3 | | NeuronalProfilingV42Well:SE\_E2NeuriteSpotAvgAreaCh3 | | NeuronalProfilingV42Well:SE\_E2NeuriteSpotAvgCountCh3 | | NeuronalProfilingV42Well:SE\_E2NeuriteSpotAvgIntenCh3 | | NeuronalProfilingV42Well:SE\_E2NeuriteSpotOverlapAreaCh3 | | NeuronalProfilingV42Well:SE\_E2NeuriteSpotOverlapCountCh3 | | NeuronalProfilingV42Well:SE\_E2NeuriteSpotTotalAreaCh3 | | NeuronalProfilingV42Well:SE\_E2NeuriteSpotTotalCountCh3 | | NeuronalProfilingV42Well:SE\_E2NeuriteSpotTotalIntenCh3 | | NeuronalProfilingV42Well:SE\_E2NeuriteSpotTotalIntenRatioCh3Ch2 | | NeuronalProfilingV42Well:SE\_E2NeuriteTotalAreaCh2 | | NeuronalProfilingV42Well:SE\_E2NeuriteTotalCountCh2 | | NeuronalProfilingV42Well:SE\_E2NeuriteTotalIntenCh2 | | NeuronalProfilingV42Well:SE\_E2NeuriteTotalLengthCh2 | | NeuronalProfilingV42Well:SE\_E2NeuriteVarIntenCh2 | | NeuronalProfilingV42Well:SE\_E2NeuriteWidthCh2 | | NeuronalProfilingV42Well:SE\_E2TotalIntenCh3 | | NeuronalProfilingV42Well:SE\_E3AvgIntenCh3 | | NeuronalProfilingV42Well:SE\_E3BranchPointAvgCountCh2 | | NeuronalProfilingV42Well:SE\_E3BranchPointAvgDistFromCellBodyCh2 | | NeuronalProfilingV42Well:SE\_E3BranchPointCountPerNeuriteLengthCh2 | | NeuronalProfilingV42Well:SE\_E3BranchPointTotalCountCh2 | | NeuronalProfilingV42Well:SE\_E3CellBodyAreaCh2 | | NeuronalProfilingV42Well:SE\_E3CellBodyAvgIntenCh2 | | NeuronalProfilingV42Well:SE\_E3CellBodyNucAvgIntenCh1 | | NeuronalProfilingV42Well:SE\_E3CellBodyNucCountCh2 | | NeuronalProfilingV42Well:SE\_E3CellBodyNucTotalAreaCh1 | | NeuronalProfilingV42Well:SE\_E3CellBodyNucTotalIntenCh1 | | NeuronalProfilingV42Well:SE\_E3CellBodyShapeLWRCh2 | | NeuronalProfilingV42Well:SE\_E3CellBodyShapeP2ACh2 | | NeuronalProfilingV42Well:SE\_E3CellBodySpot%OverlapAreaCh3 | | NeuronalProfilingV42Well:SE\_E3CellBodySpot%OverlapCountCh3 | | NeuronalProfilingV42Well:SE\_E3CellBodySpotAvgIntenCh3 | | NeuronalProfilingV42Well:SE\_E3CellBodySpotOverlapAreaCh3 | | NeuronalProfilingV42Well:SE\_E3CellBodySpotOverlapCountCh3 | | NeuronalProfilingV42Well:SE\_E3CellBodySpotTotalAreaCh3 | | NeuronalProfilingV42Well:SE\_E3CellBodySpotTotalCountCh3 | | NeuronalProfilingV42Well:SE\_E3CellBodySpotTotalIntenCh3 | | NeuronalProfilingV42Well:SE\_E3CellBodySpotTotalIntenRatioCh3Ch2 | | NeuronalProfilingV42Well:SE\_E3CellBodyTotalIntenCh2 | | NeuronalProfilingV42Well:SE\_E3CrossPointAvgCountCh2 | | NeuronalProfilingV42Well:SE\_E3CrossPointTotalCountCh2 | | NeuronalProfilingV42Well:SE\_E3NeuriteAvgIntenCh2 | | NeuronalProfilingV42Well:SE\_E3NeuriteAvgLengthCh2 | | NeuronalProfilingV42Well:SE\_E3NeuriteCriticalValueCh2 | | NeuronalProfilingV42Well:SE\_E3NeuriteDendriteMaxCh2 | | NeuronalProfilingV42Well:SE\_E3NeuriteMaxLengthWithBranchesCh2 | | NeuronalProfilingV42Well:SE\_E3NeuriteMaxLengthWithoutBranchesCh2 | | NeuronalProfilingV42Well:SE\_E3NeuriteRamificationIndexCh2 | | NeuronalProfilingV42Well:SE\_E3NeuriteSpot%OverlapAreaCh3 | | NeuronalProfilingV42Well:SE\_E3NeuriteSpot%OverlapCountCh3 | | NeuronalProfilingV42Well:SE\_E3NeuriteSpotAvgAreaCh3 | | NeuronalProfilingV42Well:SE\_E3NeuriteSpotAvgCountCh3 | | NeuronalProfilingV42Well:SE\_E3NeuriteSpotAvgIntenCh3 | | NeuronalProfilingV42Well:SE\_E3NeuriteSpotOverlapAreaCh3 | | NeuronalProfilingV42Well:SE\_E3NeuriteSpotOverlapCountCh3 | | NeuronalProfilingV42Well:SE\_E3NeuriteSpotTotalAreaCh3 | | NeuronalProfilingV42Well:SE\_E3NeuriteSpotTotalCountCh3 | | NeuronalProfilingV42Well:SE\_E3NeuriteSpotTotalIntenCh3 | | NeuronalProfilingV42Well:SE\_E3NeuriteSpotTotalIntenRatioCh3Ch2 | | NeuronalProfilingV42Well:SE\_E3NeuriteTotalAreaCh2 | | NeuronalProfilingV42Well:SE\_E3NeuriteTotalCountCh2 | | NeuronalProfilingV42Well:SE\_E3NeuriteTotalIntenCh2 | | NeuronalProfilingV42Well:SE\_E3NeuriteTotalLengthCh2 | | NeuronalProfilingV42Well:SE\_E3NeuriteVarIntenCh2 | | NeuronalProfilingV42Well:SE\_E3NeuriteWidthCh2 | | NeuronalProfilingV42Well:SE\_E3TotalIntenCh3 | | NeuronalProfilingV42Well:SE\_NeuriteAvgIntenCh2 | | NeuronalProfilingV42Well:SE\_NeuriteAvgLengthCh2 | | NeuronalProfilingV42Well:SE\_NeuriteCriticalValueCh2 | | NeuronalProfilingV42Well:SE\_NeuriteDendriteMaxCh2 | | NeuronalProfilingV42Well:SE\_NeuriteMaxLengthWithBranchesCh2 | | NeuronalProfilingV42Well:SE\_NeuriteMaxLengthWithoutBranchesCh2 | | NeuronalProfilingV42Well:SE\_NeuriteRamificationIndexCh2 | | NeuronalProfilingV42Well:SE\_NeuriteSpot%OverlapAreaCh3 | | NeuronalProfilingV42Well:SE\_NeuriteSpot%OverlapCountCh3 | | NeuronalProfilingV42Well:SE\_NeuriteSpotAvgAreaCh3 | | NeuronalProfilingV42Well:SE\_NeuriteSpotAvgCountCh3 | | NeuronalProfilingV42Well:SE\_NeuriteSpotAvgIntenCh3 | | NeuronalProfilingV42Well:SE\_NeuriteSpotOverlapAreaCh3 | | NeuronalProfilingV42Well:SE\_NeuriteSpotOverlapCountCh3 | | NeuronalProfilingV42Well:SE\_NeuriteSpotTotalAreaCh3 | | NeuronalProfilingV42Well:SE\_NeuriteSpotTotalCountCh3 | | NeuronalProfilingV42Well:SE\_NeuriteSpotTotalIntenCh3 | | NeuronalProfilingV42Well:SE\_NeuriteSpotTotalIntenRatioCh3Ch2 | | NeuronalProfilingV42Well:SE\_NeuriteTotalAreaCh2 | | NeuronalProfilingV42Well:SE\_NeuriteTotalCountCh2 | | NeuronalProfilingV42Well:SE\_NeuriteTotalIntenCh2 | | NeuronalProfilingV42Well:SE\_NeuriteTotalLengthCh2 | | NeuronalProfilingV42Well:SE\_NeuriteVarIntenCh2 | | NeuronalProfilingV42Well:SE\_NeuriteWidthCh2 | | NeuronalProfilingV42Well:SE\_TotalIntenCh3 | | NeuronalProfilingV42Well:SelectedNeuronCount | | NeuronalProfilingV42Well:SelectedNeuronCountPerValidField | | NeuronalProfilingV42Well:ValidFieldCount | | NeuronalProfilingV42Well:ValidNeuronCount | | NeuronalProfilingV42Well:ValidNucleusCount | | | Selected Field Features to Store | | --- | | NeuronalProfilingV42Field:%NeuronalCells | | NeuronalProfilingV42Field:%NonNeuronalCells | | NeuronalProfilingV42Field:%SelectedNeurons | | NeuronalProfilingV42Field:BranchPointCountPerNeuriteLengthCh2 | | NeuronalProfilingV42Field:BranchPointTotalCountPerFieldCh2 | | NeuronalProfilingV42Field:BranchPointTotalCountPerNeuriteCh2 | | NeuronalProfilingV42Field:BranchPointTotalCountPerNeuronCh2 | | NeuronalProfilingV42Field:CellBodySpotAvgIntenPerFieldCh3 | | NeuronalProfilingV42Field:CellBodySpotTotalAreaPerFieldCh3 | | NeuronalProfilingV42Field:CellBodySpotTotalAreaPerNeuronCh3 | | NeuronalProfilingV42Field:CellBodySpotTotalCountPerFieldCh3 | | NeuronalProfilingV42Field:CellBodySpotTotalCountPerNeuronCh3 | | NeuronalProfilingV42Field:CellBodySpotTotalIntenPerFieldCh3 | | NeuronalProfilingV42Field:CellBodySpotTotalIntenPerNeuronCh3 | | NeuronalProfilingV42Field:NA\_NeuriteAvgIntenPerFieldCh2 | | NeuronalProfilingV42Field:NA\_NeuriteSpot%OverlapAreaPerFieldCh3 | | NeuronalProfilingV42Field:NA\_NeuriteSpot%OverlapCountPerFieldCh3 | | NeuronalProfilingV42Field:NA\_NeuriteSpotAvgIntenPerFieldCh3 | | NeuronalProfilingV42Field:NA\_NeuriteSpotAvgIntenPerNeuriteLengthCh3 | | NeuronalProfilingV42Field:NA\_NeuriteSpotOverlapTotalAreaPerFieldCh3 | | NeuronalProfilingV42Field:NA\_NeuriteSpotOverlapTotalAreaPerNeuriteLengthCh3 | | NeuronalProfilingV42Field:NA\_NeuriteSpotOverlapTotalCountPerFieldCh3 | | NeuronalProfilingV42Field:NA\_NeuriteSpotOverlapTotalCountPerNeuriteLengthCh3 | | NeuronalProfilingV42Field:NA\_NeuriteSpotTotalAreaPerFieldCh3 | | NeuronalProfilingV42Field:NA\_NeuriteSpotTotalAreaPerNeuriteLengthCh3 | | NeuronalProfilingV42Field:NA\_NeuriteSpotTotalCountPerFieldCh3 | | NeuronalProfilingV42Field:NA\_NeuriteSpotTotalCountPerNeuriteLengthCh3 | | NeuronalProfilingV42Field:NA\_NeuriteSpotTotalIntenPerFieldCh3 | | NeuronalProfilingV42Field:NA\_NeuriteSpotTotalIntenPerNeuriteLengthCh3 | | NeuronalProfilingV42Field:NA\_NeuriteTotalIntenPerFieldCh2 | | NeuronalProfilingV42Field:NA\_NeuriteTotalLengthPerFieldCh2 | | NeuronalProfilingV42Field:NeuriteAvgIntenPerFieldCh2 | | NeuronalProfilingV42Field:NeuriteSpotAvgIntenPerFieldCh3 | | NeuronalProfilingV42Field:NeuriteSpotTotalAreaPerFieldCh3 | | NeuronalProfilingV42Field:NeuriteSpotTotalAreaPerNeuriteCh3 | | NeuronalProfilingV42Field:NeuriteSpotTotalAreaPerNeuriteLengthCh3 | | NeuronalProfilingV42Field:NeuriteSpotTotalAreaPerNeuronCh3 | | NeuronalProfilingV42Field:NeuriteSpotTotalCountPerFieldCh3 | | NeuronalProfilingV42Field:NeuriteSpotTotalCountPerNeuriteCh3 | | NeuronalProfilingV42Field:NeuriteSpotTotalCountPerNeuriteLengthCh3 | | NeuronalProfilingV42Field:NeuriteSpotTotalCountPerNeuronCh3 | | NeuronalProfilingV42Field:NeuriteSpotTotalIntenPerFieldCh3 | | NeuronalProfilingV42Field:NeuriteSpotTotalIntenPerNeuriteCh3 | | NeuronalProfilingV42Field:NeuriteSpotTotalIntenPerNeuriteLengthCh3 | | NeuronalProfilingV42Field:NeuriteSpotTotalIntenPerNeuronCh3 | | NeuronalProfilingV42Field:NeuriteTotalCountPerFieldCh2 | | NeuronalProfilingV42Field:NeuriteTotalCountPerNeuronCh2 | | NeuronalProfilingV42Field:NeuriteTotalIntenPerFieldCh2 | | NeuronalProfilingV42Field:NeuriteTotalLengthPerFieldCh2 | | NeuronalProfilingV42Field:NeuriteTotalLengthPerNeuriteCh2 | | NeuronalProfilingV42Field:NeuriteTotalLengthPerNeuronCh2 | | NeuronalProfilingV42Field:NeuriteVarIntenPerFieldCh2 | | NeuronalProfilingV42Field:NeuronalNucleusCount | | NeuronalProfilingV42Field:NeuronNucleusRatio | | NeuronalProfilingV42Field:NonNeuronalNucleusCount | | NeuronalProfilingV42Field:SelectedNeuronCount | | NeuronalProfilingV42Field:SelectedNeuronCountPerValidField | | NeuronalProfilingV42Field:ValidFieldCount | | NeuronalProfilingV42Field:ValidNeuronCount | | NeuronalProfilingV42Field:ValidNucleusCount | | | Selected Cell Features to Store | | --- | | NeuronalProfilingV42Cell:AvgIntenCh3 | | NeuronalProfilingV42Cell:AvgIntenCh3Status | | NeuronalProfilingV42Cell:BranchPointAvgCountCh2 | | NeuronalProfilingV42Cell:BranchPointAvgCountCh2Status | | NeuronalProfilingV42Cell:BranchPointAvgDistFromCellBodyCh2 | | NeuronalProfilingV42Cell:BranchPointAvgDistFromCellBodyCh2Status | | NeuronalProfilingV42Cell:BranchPointCountPerNeuriteLengthCh2 | | NeuronalProfilingV42Cell:BranchPointCountPerNeuriteLengthCh2Status | | NeuronalProfilingV42Cell:BranchPointTotalCountCh2 | | NeuronalProfilingV42Cell:BranchPointTotalCountCh2Status | | NeuronalProfilingV42Cell:Cell# | | NeuronalProfilingV42Cell:CellBodyAreaCh2 | | NeuronalProfilingV42Cell:CellBodyAreaCh2Status | | NeuronalProfilingV42Cell:CellBodyAvgIntenCh2 | | NeuronalProfilingV42Cell:CellBodyAvgIntenCh2Status | | NeuronalProfilingV42Cell:CellBodyNucAvgIntenCh1 | | NeuronalProfilingV42Cell:CellBodyNucAvgIntenCh1Status | | NeuronalProfilingV42Cell:CellBodyNucCountCh2 | | NeuronalProfilingV42Cell:CellBodyNucCountCh2Status | | NeuronalProfilingV42Cell:CellBodyNucTotalAreaCh1 | | NeuronalProfilingV42Cell:CellBodyNucTotalAreaCh1Status | | NeuronalProfilingV42Cell:CellBodyNucTotalIntenCh1 | | NeuronalProfilingV42Cell:CellBodyNucTotalIntenCh1Status | | NeuronalProfilingV42Cell:CellBodyShapeLWRCh2 | | NeuronalProfilingV42Cell:CellBodyShapeLWRCh2Status | | NeuronalProfilingV42Cell:CellBodyShapeP2ACh2 | | NeuronalProfilingV42Cell:CellBodyShapeP2ACh2Status | | NeuronalProfilingV42Cell:CellBodySpot%OverlapAreaCh3 | | NeuronalProfilingV42Cell:CellBodySpot%OverlapAreaCh3Status | | NeuronalProfilingV42Cell:CellBodySpot%OverlapCountCh3 | | NeuronalProfilingV42Cell:CellBodySpot%OverlapCountCh3Status | | NeuronalProfilingV42Cell:CellBodySpotAvgIntenCh3 | | NeuronalProfilingV42Cell:CellBodySpotAvgIntenCh3Status | | NeuronalProfilingV42Cell:CellBodySpotOverlapAreaCh3 | | NeuronalProfilingV42Cell:CellBodySpotOverlapAreaCh3Status | | NeuronalProfilingV42Cell:CellBodySpotOverlapCountCh3 | | NeuronalProfilingV42Cell:CellBodySpotOverlapCountCh3Status | | NeuronalProfilingV42Cell:CellBodySpotTotalAreaCh3 | | NeuronalProfilingV42Cell:CellBodySpotTotalAreaCh3Status | | NeuronalProfilingV42Cell:CellBodySpotTotalCountCh3 | | NeuronalProfilingV42Cell:CellBodySpotTotalCountCh3Status | | NeuronalProfilingV42Cell:CellBodySpotTotalIntenCh3 | | NeuronalProfilingV42Cell:CellBodySpotTotalIntenCh3Status | | NeuronalProfilingV42Cell:CellBodySpotTotalIntenRatioCh3Ch2 | | NeuronalProfilingV42Cell:CellBodySpotTotalIntenRatioCh3Ch2Status | | NeuronalProfilingV42Cell:CellBodyTotalIntenCh2 | | NeuronalProfilingV42Cell:CellBodyTotalIntenCh2Status | | NeuronalProfilingV42Cell:CrossPointAvgCountCh2 | | NeuronalProfilingV42Cell:CrossPointAvgCountCh2Status | | NeuronalProfilingV42Cell:CrossPointTotalCountCh2 | | NeuronalProfilingV42Cell:CrossPointTotalCountCh2Status | | NeuronalProfilingV42Cell:EventType1Status | | NeuronalProfilingV42Cell:EventType2Status | | NeuronalProfilingV42Cell:EventType3Status | | NeuronalProfilingV42Cell:EventTypeProfile | | NeuronalProfilingV42Cell:Height | | NeuronalProfilingV42Cell:Left | | NeuronalProfilingV42Cell:NeuriteAvgIntenCh2 | | NeuronalProfilingV42Cell:NeuriteAvgIntenCh2Status | | NeuronalProfilingV42Cell:NeuriteAvgLengthCh2 | | NeuronalProfilingV42Cell:NeuriteAvgLengthCh2Status | | NeuronalProfilingV42Cell:NeuriteCriticalValueCh2 | | NeuronalProfilingV42Cell:NeuriteCriticalValueCh2Status | | NeuronalProfilingV42Cell:NeuriteDendriteMaxCh2 | | NeuronalProfilingV42Cell:NeuriteDendriteMaxCh2Status | | NeuronalProfilingV42Cell:NeuriteMaxLengthWithBranchesCh2 | | NeuronalProfilingV42Cell:NeuriteMaxLengthWithBranchesCh2Status | | NeuronalProfilingV42Cell:NeuriteMaxLengthWithoutBranchesCh2 | | NeuronalProfilingV42Cell:NeuriteMaxLengthWithoutBranchesCh2Status | | NeuronalProfilingV42Cell:NeuriteRamificationIndexCh2 | | NeuronalProfilingV42Cell:NeuriteRamificationIndexCh2Status | | NeuronalProfilingV42Cell:NeuriteSpot%OverlapAreaCh3 | | NeuronalProfilingV42Cell:NeuriteSpot%OverlapAreaCh3Status | | NeuronalProfilingV42Cell:NeuriteSpot%OverlapCountCh3 | | NeuronalProfilingV42Cell:NeuriteSpot%OverlapCountCh3Status | | NeuronalProfilingV42Cell:NeuriteSpotAvgAreaCh3 | | NeuronalProfilingV42Cell:NeuriteSpotAvgAreaCh3Status | | NeuronalProfilingV42Cell:NeuriteSpotAvgCountCh3 | | NeuronalProfilingV42Cell:NeuriteSpotAvgCountCh3Status | | NeuronalProfilingV42Cell:NeuriteSpotAvgIntenCh3 | | NeuronalProfilingV42Cell:NeuriteSpotAvgIntenCh3Status | | NeuronalProfilingV42Cell:NeuriteSpotOverlapAreaCh3 | | NeuronalProfilingV42Cell:NeuriteSpotOverlapAreaCh3Status | | NeuronalProfilingV42Cell:NeuriteSpotOverlapCountCh3 | | NeuronalProfilingV42Cell:NeuriteSpotOverlapCountCh3Status | | NeuronalProfilingV42Cell:NeuriteSpotTotalAreaCh3 | | NeuronalProfilingV42Cell:NeuriteSpotTotalAreaCh3Status | | NeuronalProfilingV42Cell:NeuriteSpotTotalCountCh3 | | NeuronalProfilingV42Cell:NeuriteSpotTotalCountCh3Status | | NeuronalProfilingV42Cell:NeuriteSpotTotalIntenCh3 | | NeuronalProfilingV42Cell:NeuriteSpotTotalIntenCh3Status | | NeuronalProfilingV42Cell:NeuriteSpotTotalIntenRatioCh3Ch2 | | NeuronalProfilingV42Cell:NeuriteSpotTotalIntenRatioCh3Ch2Status | | NeuronalProfilingV42Cell:NeuriteTotalAreaCh2 | | NeuronalProfilingV42Cell:NeuriteTotalAreaCh2Status | | NeuronalProfilingV42Cell:NeuriteTotalCountCh2 | | NeuronalProfilingV42Cell:NeuriteTotalCountCh2Status | | NeuronalProfilingV42Cell:NeuriteTotalIntenCh2 | | NeuronalProfilingV42Cell:NeuriteTotalIntenCh2Status | | NeuronalProfilingV42Cell:NeuriteTotalLengthCh2 | | NeuronalProfilingV42Cell:NeuriteTotalLengthCh2Status | | NeuronalProfilingV42Cell:NeuriteVarIntenCh2 | | NeuronalProfilingV42Cell:NeuriteVarIntenCh2Status | | NeuronalProfilingV42Cell:NeuriteWidthCh2 | | NeuronalProfilingV42Cell:NeuriteWidthCh2Status | | NeuronalProfilingV42Cell:Top | | NeuronalProfilingV42Cell:TotalIntenCh3 | | NeuronalProfilingV42Cell:TotalIntenCh3Status | | NeuronalProfilingV42Cell:Width | | NeuronalProfilingV42Cell:XCentroid | | NeuronalProfilingV42Cell:YCentroid | | | |
