# Supplementary material for: Celf4 controls mRNA translation underlying synaptic development in the prenatal mammalian neocortex
Source: Nat Commun. 2023 Sep 27;14:6025. doi: 10.1038/s41467-023-41730-8 (PMC10533865; doi:10.1038/s41467-023-41730-8)
Supplement: Supplementary file 5 — Reporting Summary [file 41467_2023_41730_MOESM5_ESM.pdf]

Corresponding author(s): Mladen-Roko Rasin
Zeljka Krsnik

Last updated by author(s): Aug 15, 2023

Reporting Summary

Nature Portfolio wishes to improve the reproducibility of the work that we publish. This form provides structure for consistency and transparency in reporting. For further information on Nature Portfolio policies, see our Editorial Policies and the Editorial Policy Checklist.

Please do not complete any field with "not applicable" or n/a. Refer to the help text for what text to use if an item is not relevant to your study.
For final submission: please carefully check your responses for accuracy; you will not be able to make changes later.

Statistics

For all statistical analyses, confirm that the following items are present in the figure legend, table legend, main text, or Methods section.

n/a	Confirmed	
☐	☒	The exact sample size (*n*) for each experimental group/condition, given as a discrete number and unit of measurement
☐	☒	A statement on whether measurements were taken from distinct samples or whether the same sample was measured repeatedly
☐	☒	The statistical test(s) used AND whether they are one- or two-sided<br>*Only common tests should be described solely by name; describe more complex techniques in the Methods section.*
☐	☒	A description of all covariates tested
☐	☒	A description of any assumptions or corrections, such as tests of normality and adjustment for multiple comparisons
☐	☒	A full description of the statistical parameters including central tendency (e.g. means) or other basic estimates (e.g. regression coefficient) AND variation (e.g. standard deviation) or associated estimates of uncertainty (e.g. confidence intervals)
☐	☒	For null hypothesis testing, the test statistic (e.g. *F*, *t*, *r*) with confidence intervals, effect sizes, degrees of freedom and *P* value noted<br>*Give P values as exact values whenever suitable.*
☒	☐	For Bayesian analysis, information on the choice of priors and Markov chain Monte Carlo settings
☒	☐	For hierarchical and complex designs, identification of the appropriate level for tests and full reporting of outcomes
☒	☐	Estimates of effect sizes (e.g. Cohen's *d*, Pearson's *r*), indicating how they were calculated

Our web collection on statistics for biologists contains articles on many of the points above.

Software and code

Policy information about availability of computer code

Data collection	WinDaq acquisition software (version 2.84) was used for the acquisition of polysome profiles. RNA isolated from input, monosome-pooled and polysome-pooled fractions was submitted for quality control testing, library preparations and sequencing reactions using Illumina®HiSeq® at Azenta, Inc. (South Plainfield, NJ).<br>Sample and library preparation for single-nucleus RNAseq were done using the 10X Genomics® Chromium™ 3' gene expression workflow (Drop-Seq technology) by Azenta, Inc. (South Plainfield, NJ), followed by Illumina® NovaSeq™6000 sequencing at Azenta, Inc.<br>Western blot chemiluminescence images were captured using Azure 600® (Azure Byosystems; #AZI600).<br>Human Nissl images were captured using Hamamatsu NanoZoomer 2.0 RS system using a 40x (NA 0.75) objective lens.<br>Human immunofluorescence images were captured using either Hamamatsu LX2000 Lightning exciter or Olympus FV3000 microscope with FV31S-SW Fluoview software.<br>Mouse immunofluorescence images were captured using Olympus BX61WI confocal microscope and Fluoview FV-1000 software for image processing.<br>qRT-PCR data were obtained using QuantStudio3 Real-Time PCR machine (Thermo Fisher Scientific).
Data analysis	Single-nucleus RNAseq data (10x Chromium data) were analyzed utilizing the 10X Genomics Cell Ranger pipeline to generate demultiplexed UMI count matrices. Count data were imported into the single cell 'Seurat' R package (version 4.0.6). The matrices from individual samples were loaded into a single Seurat object, clustered by a shared nearest neighbor (SNN) algorithm, colored by SNN cluster assignment using Seurat FindClusters and visualized per cell type in the two-dimensional space using scCustomize v.1.1.1.<br>Seurat's FindAllMarkers() function was used to perform differential expression analysis.<br>GO term enrichment was performed using either the clusterProfiler package (v3.10.1) in R or PANTHER online open-source knowledgebase (Protein Analysis Through Evolutionary Relationships, http://pantherdb.org).<br>Synaptic gene ontologies analyses were performed using the online open-source knowledgebase SynGO available at https://

www.syngoportal.org/.
For polysome-RNAseq analysis FASTQ files were quality trimmed using Trimmomatic (v.0.3.6). After mapping of the reads to either human genome (hg38/GRCh38 assembly) or mouse genome (GRCm39 assembly) using STAR (v2.5.2b), read counts were summarized at the gene level with the featureCounts() function from the Subread package (v1.5.2).
Differential gene expression was modeled in DESeq2.
Custom script in R (R studio 1.2.5042) was used for NDD- and ASD-risk gene set enrichment analysis.
NDP.view2 Viewing software (U12388-01) was used for processing of human immunohistochemistry images.
CellInsight CX7 High-Content Screening (HCS) platform using the Neuronal Profiling analysis was used for analysis of synaptic markers.
FIJI distribution of ImageJ2 software (v2.3.0/1.53f; https://fiji.sc/) was used for image processing, densitometry analyses and quantitative analyses.
Software Gimp2.10.14 was used for analysis of confocal images.
GraphPad Prism9 software (version 9.4.1) was used for statistical analysis of confocal images and qRT-PCR data.

For manuscripts utilizing custom algorithms or software that are central to the research but not yet described in published literature, software must be made available to editors and reviewers. We strongly encourage code deposition in a community repository (e.g. GitHub). See the Nature Portfolio guidelines for submitting code & software for further information.

Data

Policy information about availability of data

All manuscripts must include a data availability statement. This statement should provide the following information, where applicable:

- Accession codes, unique identifiers, or web links for publicly available datasets
- A description of any restrictions on data availability
- For clinical datasets or third party data, please ensure that the statement adheres to our policy

The RNAseq and snRNAseq datasets generated in this study have been deposited at NCBI GEO, are publicly available as of the date of publication, and can be downloaded from the GEO database under accession number GSE214534. This SuperSeries is composed of the following SubSeries: GSE214272 (polysome RNAseq of human fetal neocortex), GSE214327 (human fetal neocortex CELF4 RIP-RNAseq), GSE214328 (polysome RNAseq of mouse neocortex; WTs and Emx1-Celf4 cKOs), and GSE214532 (snRNAseq in human fetal neocortex). Genome assembly hg38/GRCh38 was used for human sequencing data. Genome assembly GRCm39 was used for mouse sequencing data. Source data are provided with this study.

Human research participants

Policy information about studies involving human research participants and Sex and Gender in Research.

Reporting on sex and gender	The sex was not determined due to the limited availability of human fetal neocortical tissue.
Population characteristics	Postmortem human fetal brain samples from 11 to 21 post-conceptual weeks (PCW) within the six hours of postmortem time.
Recruitment	Postmortem human tissue is donated voluntarily. Written consent to donate is given before any tissue is collected.
Ethics oversight	Human developmental tissues were collected with appropriate maternal consent and approval for use in research. Ethical approval was obtained from the relevant ethics committees (Newcastle and North Tyneside 1 NHS Health Authority Research Ethics Committee, Fulham NHS Health Authority Research Ethics Committee and the Internal Review Board of the Ethical Committee of the University of Zagreb School of Medicine).

Note that full information on the approval of the study protocol must also be provided in the manuscript.

Field-specific reporting

Please select the one below that is the best fit for your research. If you are not sure, read the appropriate sections before making your selection.

☒ Life sciences ☐ Behavioural & social sciences ☐ Ecological, evolutionary & environmental sciences

Life sciences study design

All studies must disclose on these points even when the disclosure is negative.

Sample size	For single-nucleus RNAseq (10x Genomics), whole nuclei were isolated two human necortices per each developmental phase. The number of human cortices and nuclei were determined to ensure the biological replicates for each cell population and the number of cells in each population met or exceed the comparable published single-cell datasets (e.g. Polioudakis et al. 2019).<br><br>Sample sizes were determined based on either the preliminary data or a priori knowledge of experiments. All methods practiced in this paper are standard to our lab, and publication records speak to this. A sample size of three is a standard starting point with minimum of 2 sections

per mouse used for every statistical analysis, unless otherwise notified. The sample sizes used are listed in the respective Figure captions. ThermoFisher CellInsight CX7 is high throughput data acquisition system that significantly increases the number of analyzed morphometric features that can be collected at the same time, 2 pups/genotype were also sufficient for the analysis. Littermate wild-type (WT) and/or knockout (cKO) cortices were used simultaneously in all mouse experiments to minimize the variability between embryos and/or neonates.

Data exclusions	No data was specifically excluded, but filtering was performed as described in the Methods section to exclude nucleus of low quality. All exclusion criteria were pre-established based on previous work.
Replication	All experiments included at least three biological replicates, unless otherwise notified, and differences between replicates were evaluated. All attempts at replication were successful. The number of replicate experiments and samples used is indicated in the figure legend of each figure. Human CELF4 RIP qRT-PCRs were run at a minimum of two technical replicates, while all the other experiments were run at a minimum of three technical replicates.
Randomization	Randomization is not relevant for this study because we are observing the neurodevelopmental effect of Celf4 deletion. There is no treatment or intervention to the samples. Our animal groups consist out of Emx1-Cre/Celf4 cKOs and their littermate controls.
Blinding	Every sample used in this study was assigned a specific numerical number upon collection of the neocortices. Thus, the investigators were blinded to group allocation during data collection and data analysis. The samples were unblinded before statistical analysis. Cell counting was done in double blind fashion where neither the person imaging nor quantifying knew the experimental condition. To avoid bias, all samples were treated equally with the same rigorous criteria.

Reporting for specific materials, systems and methods

We require information from authors about some types of materials, experimental systems and methods used in many studies. Here, indicate whether each material, system or method listed is relevant to your study. If you are not sure if a list item applies to your research, read the appropriate section before selecting a response.

Materials & experimental systems

n/a	Involved in the study	
☐	☒	Antibodies
☒	☐	Eukaryotic cell lines
☒	☐	Palaeontology and archaeology
☐	☒	Animals and other organisms
☒	☐	Clinical data
☒	☐	Dual use research of concern

Methods

n/a	Involved in the study	
☒	☐	ChIP-seq
☒	☐	Flow cytometry
☒	☐	MRI-based neuroimaging

Antibodies

Antibodies used

Primary antibodies and dilutions used on human fetal sections were:
• rabbit polyclonal anti-Celf4/ BRUNOL4 (dilution 1/250, Invitrogen, #PA5-58196, lot.no. UG2806225A) – https://www.thermofisher.com/antibody/product/BRUNOL4-Antibody-Polyclonal/PA5-58196
• rat monoclonal anti-Ctip2 (clone: 25B6, dilution 1/500; Abcam; #ab18465, lot.no. #GR3272266-22) – https://www.abcam.com/products/primary-antibodies/ctip2-antibody-25b6-ab18465.html
• goat polyclonal anti-Nurr1/NGFI-Bβ/NR4A2 (dilution 1/250, R&D Systems, #AF2156, lot. no: UUW0318031) – https://www.rndsystems.com/products/human-mouse-nurr1-ngfi-bbeta-nr4a2-antibody_af2156?gclid=CjwKCAjww7KmBhAyEiwA5-PUSs4yaCP_-tII7j4H92ZxRGF92xePbFkT5kIW7vSmGezrHwmYs0KCYxoCaoEQAvD_BwE&gclsrc=aw.ds
• mouse monoclonal anti-Eif4a2 (clone: G-5, dilution 1/400, Santa Cruz Biotechnology (SCB), #sc137147, lot.no: AO411) – https://www.scbt.com/p/eif4aii-antibody-g-5
• rabbit monoclonal anti-Sv2a (clone: D1L8S, dilution 1/400, Cell Signaling Technology, #66724S, lot.no: 1) – https://www.cellsignal.com/products/primary-antibodies/sv2a-d1l8s-rabbit-mab/66724
• mouse monoclonal anti-TLE4 (clone: E-10, dilution 1/50; Santa Cruz; #sc365406, lot.no. #L1015) – https://www.scbt.com/p/tle4-antibody-e-10
• rabbit polyclonal anti-SERPIN1/Neuroserpin (dilution 1/200, Abcam, #ab330777) – https://www.abcam.com/products/primary-antibodies/neuroserpin-antibody-ab33077.html
• mouse monoclonal anti-Syp (clone: SY38, dilution 1/400, Invitrogen, #MA1-213, lot.no: WJ337774) – https://www.thermofisher.com/antibody/product/Synaptophysin-Antibody-clone-SY38-Monoclonal/MA1-213

Secondary antibodies and dilutions used on human fetal sections were:
• Donkey anti-rabbit Alexa Fluor 488 (dilution 1/1000, ThermoFisher Scientific, #A-32790) – https://www.thermofisher.com/antibody/product/Donkey-anti-Rabbit-IgG-H-L-Highly-Cross-Adsorbed-Secondary-Antibody-Polyclonal/A32790
• Donkey anti-mouse Alexa Fluor 555 (dilution 1/1000, ThermoFisher Scientific, #A-32773) – https://www.thermofisher.com/antibody/product/Donkey-anti-Mouse-IgG-H-L-Highly-Cross-Adsorbed-Secondary-Antibody-Polyclonal/A32773
• Goat anti-rat Alexa Fluor 555 (dilution 1/1000, ThermoFisher Scientific, #A-21434) –https://www.thermofisher.com/antibody/product/Goat-anti-Rat-IgG-H-L-Cross-Adsorbed-Secondary-Antibody-Polyclonal/A-21434
• Donkey anti-goat Alexa Fluor 488 (dilution 1/1000, ThermoFisher Scientific, #A-11055) – https://www.thermofisher.com/antibody/product/Donkey-anti-Goat-IgG-H-L-Cross-Adsorbed-Secondary-Antibody-Polyclonal/A-11055
• Goat anti-mouse Alexa Fluor 488 (dilution 1/1000, ThermoFisher Scientific, #A-11001)
https://www.thermofisher.com/antibody/product/Goat-anti-Mouse-IgG-H-L-Cross-Adsorbed-Secondary-Antibody-Polyclonal/

A-11001
• Donkey anti-mouse Alexa Fluor 647 (dilution 1/1000, ThermoFisher Scientific, #A-31571) – https://www.thermofisher.com/antibody/product/Donkey-anti-Mouse-IgG-H-L-Highly-Cross-Adsorbed-Secondary-Antibody-Polyclonal/A-31571

Primary antibodies and dilutions used on mouse sections were:
• rabbit polyclonal anti-Celf4/ BRUNOL4 (IHC dilution 1/500, IF/FISH dilution 1/50, Invitrogen, #PA5-58196, lot.no. UG2806225A) – https://www.thermofisher.com/antibody/product/BRUNOL4-Antibody-Polyclonal/PA5-58196
• mouse monoclonal anti-Eif4a2 (clone: G-5, dilution 1/400, Santa Cruz Biotechnology (SCB), #sc137147, lot.no: AO411) – https://www.scbt.com/p/eif4aii-antibody-g-5
• rabbit monoclonal anti-Sv2a (clone: D1L8S, dilution 1/300, Cell Signaling Technology, #66724S, lot.no: 1) – https://www.cellsignal.com/products/primary-antibodies/sv2a-d1l8s-rabbit-mab/66724
• mouse monoclonal anti-Syp (clone: SY38, dilution 1/400, Invitrogen, #MA1-213, lot.no: WJ337774) – https://www.thermofisher.com/antibody/product/Synaptophysin-Antibody-clone-SY38-Monoclonal/MA1-213
• mouse monoclonal anti-PSD95/ DLG4 (clone: K28/43, dilution 1/500, UC Davis/NIH NeuroMab Facility, #75-028, RRID:AB_2292909) – https://neuromab.ucdavis.edu/datasheet/K28_43.pdf
• rabbit polyclonal anti-vGlut1 (dilution 1/4000, Synaptic Systems, #135 302, lot. no: 1-41) – https://sysy.com/product/135302
• guinea pig polyclonal anti-vGlut2 (dilution 1/2500, Synaptic Systems, #135 404, lot. no: 2-32) – https://sysy.com/product/135404#list
• guinea pig polyclonal anti-VGAT (dilution 1/2000, Synaptic Systems, #131 004, lot. no: 2-43) – https://sysy.com/product/131004#list
• mouse monoclonal anti-Gephyrin (dilution 1/100, Synaptic Systems, #147 021, lot. no: 1-26) – https://sysy.com/product/147021#list
• rabbit polyclonal anti-Complexin3 (dilution 1/1000, Synaptic Systems, #122 302, lot. no: 1-8) – https://sysy.com/product/122302#list
• goat polyclonal anti-Nurr1/NGFI-Bβ/NR4A2 (dilution 1/250, R&D Systems, #AF2156, lot. no: UUW0318031) – https://www.rndsystems.com/products/human-mouse-nurr1-ngfi-bbeta-nr4a2-antibody_af2156?gclid=CjwKCAjww7KmBhAyEiwA5-PUSs4yaCP_-tII7j4H92ZxRGF92xePbFkT5kIW7vSmGezrHwmYs0KCYxoCaoEQAvD_BwE&gclsrc=aw.ds
• rat monoclonal anti-Ctip2/Bcl11b (clone: 25B6, dilution 1/250, Abcam, #ab18465, lot. no: GR3272266-2) – https://www.abcam.com/products/primary-antibodies/ctip2-antibody-25b6-ab18465.html
• goat polyclonal anti-Brn1/POU3F3 (dilution 1/600, Novus Biologicals, #NBP1-49872, lot.no: P1 E210518) – https://www.novusbio.com/products/pou3f3-antibody_nbp1-49872
• mouse monoclonal anti-Satb2 (clone: SATBA4B10, dilution 1/250, Abcam, #ab51502, lot. no: GR3174877-4) – https://www.abcam.com/products/primary-antibodies/satb1--satb2-antibody-satba4b10-c-terminal-ab51502.html
• rabbit polyclonal anti-CDP/CUX1 (clone: M-222, dilution 1/250, Santa Cruz Biotechnology, #sc13024, lot. no: E0914) – https://www.scbt.com/p/cdp-antibody-m-222
• mouse monoclonal anti-Reelin (clone: G10, dilution 1/800, Millipore Sigma, #MAB5364, lot. no: 3099957) – https://www.emdmillipore.com/US/en/product/Anti-Reelin-Antibody-a.a.-164-496-mreelin-clone-G10,MM_NF-MAB5364
• rat monoclonal anti-BrdU/CldU (clone: BU1/75 (ICR1), dilution 1/200, Abcam, #ab6326, lot. no: GR3365969-5) – https://www.abcam.com/products/primary-antibodies/brdu-antibody-bu175-icr1-proliferation-marker-ab6326.html
• mouse anti-BrdU/IdU (clone: B44, dilution 1/100, BD Biosciences, #347580) – https://www.fishersci.com/shop/products/anti-brdu-bromodeoxyuridine-clone-bd/BDB347580
• goat polyclonal anti-Foxp2 (clone: N16, dilution 1/250, Santa Cruz Biotechnology, #sc-21069, lot. no: E0715) – https://datasheets.scbt.com/sc-21069.pdf
• mouse monoclonal anti-Sv2/Sv2a (myeloma strain: SP2/0, dilution 1/1000, Developmental Studies Hybridoma Bank) – https://dshb.biology.uiowa.edu/SV2
• mouse monoclonal anti-Gapdh (clone: 6C5, dilution 1/2000, Millipore Sigma, #MAB374, lot. no: 3189695) – https://www.emdmillipore.com/US/en/product/Anti-Glyceraldehyde-3-Phosphate-Dehydrogenase-Antibody-clone-6C5,MM_NF-MAB374?ReferrerURL=https%3A%2F%2Fwww.google.com%2F

Secondary antibodies and dilutions used on mouse sections were:
• Alexa Fluor® 488 AffiniPure Donkey Anti-Rabbit IgG (H+L) (dilution 1/250, Jackson ImmunoResearch, # 711-545-152) – https://www.jacksonimmuno.com/catalog/products/711-545-152
• Cy™3 AffiniPure Donkey Anti-Rabbit IgG (H+L) (dilution 1/250, Jackson ImmunoResearch, #711-165-152) – https://www.jacksonimmuno.com/catalog/products/711-165-152
• Cy™5 AffiniPure Donkey Anti-Rabbit IgG (H+L) (dilution 1/250, Jackson ImmunoResearch, # 711-175-152) – https://www.jacksonimmuno.com/catalog/products/711-175-152
• Peroxidase AffiniPure Donkey Anti-Rabbit IgG (H+L) (dilution 1/1500, Jackson ImmunoResearch, #711-035-152) – https://www.jacksonimmuno.com/catalog/products/711-035-152
• Alexa Fluor® 488 AffiniPure Donkey Anti-Mouse IgG (H+L) (dilution 1/250, Jackson ImmunoResearch, #715-545-150) – https://www.jacksonimmuno.com/catalog/products/715-545-150
• Cy™3 AffiniPure Donkey Anti-Mouse IgG (H+L) (dilution 1/250, Jackson ImmunoResearch, #715-165-150) – https://www.jacksonimmuno.com/catalog/products/715-165-150
• Cy™5 AffiniPure Donkey Anti-Mouse IgG (H+L) (dilution 1/250, Jackson ImmunoResearch, #715-175-151) – https://www.jacksonimmuno.com/catalog/products/715-175-151
• Peroxidase AffiniPure Donkey Anti-Mouse IgG (H+L) (dilution 1/1500, Jackson ImmunoResearch, #715-035-150) – https://www.jacksonimmuno.com/catalog/products/715-035-150
• Cy™5 AffiniPure Donkey Anti-Goat IgG (H+L) (dilution 1/250, Jackson ImmunoResearch, #705-175-147) – https://www.jacksonimmuno.com/catalog/products/705-175-147
• Alexa Fluor® 488 AffiniPure Donkey Anti-Rat IgG (H+L) (dilution 1/250, Jackson ImmunoResearch, # 712-545-153) – https://www.jacksonimmuno.com/catalog/products/712-545-153
• Cy™3 AffiniPure Donkey Anti-Rat IgG (H+L) (dilution 1/250, Jackson ImmunoResearch, # 712-165-153) – https://www.jacksonimmuno.com/catalog/products/712-165-153
• Cy™3 AffiniPure Donkey Anti-Guinea Pig IgG (H+L) (dilution 1/250, Jackson ImmunoResearch, #706-165-148) – https://www.jacksonimmuno.com/catalog/products/706-165-148
• Cy™5 AffiniPure Donkey Anti-Guinea Pig IgG (H+L) (dilution 1/250, Jackson ImmunoResearch, # 706-175-148) – https://

www.jacksonimmuno.com/catalog/products/706-175-148

Validation

Well characterized commercial antibodies were used in this study, and were tested by the manufacturer for the method of our interest (available on the manufacturer's website). Moreover, most of these antibodies are regularly used in our lab and were previously published by our lab.

• Celf4/ BRUNOL4 – Validated in our Emx1-Cre/Celf4 knockout (cKO) line (Figure 3e, and in Western blot analyses using WT and cKO neocortical lysates (the band size is checked for the expected molecular weight in Supplementary Figure 7a), and our RIP-RNAseq experiments. To optimize antibody concentration and specificity of the signal, Celf4 antibody was tested using four different dilutions (1/200 - 1/600).
• Ctip2/Bcl11b – Validated by manufacturer using ICC/IF, WB, Flow Cyt (https://www.abcam.com/products/primary-antibodies/ctip2-antibody-25b6-ab18465.html?productWallTab=ShowAll) and in various published manuscripts (i.e. IHC in PMID: 32245946, IHC in PMID: 33907211).
• Nurr1/NGFI-Bβ/NR4A2 – Validated by manufacturer using ICC/IF (https://resources.rndsystems.com/pdfs/datasheets/af2156.pdf?v=20230804&_ga=2.219214489.1271116227.1691166199-842248431.1691166199&_gac=1.60877278.1691186448.CjwKCAjww7KmBhAyEiwA5-PUSs4yaCP_-tIl7j4H92ZxRGF92xePbFkT5kIW7vSmGezrHwmYs0KCYxoCaoEQAvD_BwE) and in various publications (i.e. PMID: 19008461).
• Eif4a2 – Validated by manufacturer using ICC/IF and WB (https://www.scbt.com/p/eif4aii-antibody-g-5), in other publications (i.e. PMID: 27160682, PMID: 26934103), and by our group using WB (the band size is checked for the expected molecular weight).
• Sv2a – Validated by manufacturer using IHC/IF and WB (https://www.cellsignal.com/products/primary-antibodies/sv2a-d1l8s-rabbit-mab/66724) and in other publications (i.e. PMID: 34531262).
• TLE4 – Validated in previous publications (i.e. IHC in PMID: 32245946, immunoblotting in PMID: 27301576, IHC in PMID: 36672166).
• SERPIN1/Neuroserpin – Validated by manufacturer using WB and ICC (https://www.abcam.com/products/primary-antibodies/neuroserpin-antibody-ab33077.html) and on other publications (i.e. IHC in PMID: 30644551 and PMID: 25859180)
• Syp – Validated by manufacturer using WB (https://www.thermofisher.com/antibody/product/Synaptophysin-Antibody-clone-SY38-Monoclonal/MA1-213) and in the previous publications (i.e. IHC in PMID: 32211407, WB in PMID: 34725421).
• PSD95/ DLG4 – Validated in various publications (i.e. IHC in PMID: 33689678, ICC and WB in PMID: 34100899, IHC in PMID: 31618636)
• vGlut1 – K.O. specificity validated by manufacturer (https://sysy.com/product/135302).
• vGlut2 – K.O. specificity validated by manufacturer (https://sysy.com/product/135404#list).
• VGAT – K.O. specificity validated by manufacturer (https://sysy.com/product/131004#list).
• Gephyrin – K.O. specificity validated by manufacturer (https://sysy.com/product/147021#list).
• Complexin3 – K.O. specificity validated by manufacturer (https://sysy.com/product/122302#list).
• Brn1/POU3F3 – Validated in various publications (i.e. IHC in PMID: 34467373, IHC in PMID: 29395907, IHC in PMID: 25273085).
• Satb2 – K.O. validated by manufacturer (https://www.abcam.com/products/primary-antibodies/satb1--satb2-antibody-satba4b10-c-terminal-ab51502.html) and in various publications (i.e. IHC in PMID: 34467373, IHC in PMID: 33907211, IHC in PMID: 32245946).
• CDP/CUX1 – Validated in various publications (i.e. IHC in PMID: 33907211, IHC in PMID: 32245946, IHC in PMID: 28475893). Reelin – Validated by manufacturer for use in WB and IHC (https://www.emdmillipore.com/US/en/product/Anti-Reelin-Antibody-a.a.-164-496-mreelin-clone-G10,MM_NF-MAB5364) and in previous publications (i.e. IHC in PMID: 32579931, IHC in PMID: 31729356).
• BrdU/CldU – Validated by manufacturer for immunofluorescence in human cell lines and tissues and for flow cytometry (https://www.abcam.com/products/primary-antibodies/brdu-antibody-bu175-icr1-proliferation-marker-ab6326.html) and in previous publications (i.e. for Single Molecule Analysis of Replicated DNA in PMID: 27102626, IHC in PMID: 31197141, neurogenesis analysis in PMID: 32245946).
• BrdU/IdU – Validated by IHC for neurogenesis analysis in PMID: 32245946, PMID: 32840212 and PMID: 29358087. Also, antibody was validated for DNA fibre assay in PMID: 27845331.
• Foxp2 – Validated in PMID: 30808549 by WB using K.O. and IHC using Knock-in mice, and previously also shown in PMID: 21765815. Also validated in PMID: 17619227 by WB and immunostaining of Foxp2-cKO and in PMID: 26245956 by IHC and WB.
• Sv2/Sv2a – Initially validated in PMID: 2579958 by IP, IF and WB and used extensively in prior publications.
• Gapdh – Validated by the manufacturer for use in ELISA, IP, IC, IF, IH & WB (https://www.emdmillipore.com/US/en/product/Anti-Glyceraldehyde-3-Phosphate-Dehydrogenase-Antibody-clone-6C5,MM_NF-MAB374?ReferrerURL=https%3A%2F%2Fwww.google.com%2F) and in other publications (i.e. WB in PMID: 31519914 and PMID: 32245946).

All Jackson ImmunoResearch or ThermoFisherScientific secondary antibodies are commercial antibodies with validation procedure described on the manufacturer websites (links provided in the "Antibodies used" section) and have been validated in previous papers (i.e. PMID: 32245946, PMID: 25157170, PMID: 34467373, PMID: 36672166, PMID: 36592215).

Animals and other research organisms

Policy information about studies involving animals; ARRIVE guidelines recommended for reporting animal research, and Sex and Gender in Research

Laboratory animals

Generation of Celf4 conditional-deletion and WT littermate control animals was accomplished by using Jackson Laboratory Emx1–Cre mice (strain name: B6.129S2-Emx1tm1(cre)Krj/J; Jax strain: 005628) crossed with Celf4f/f mice (strain name: B6.129-Celf4tm1.1Frk/Frk; Jax strain: 018126) from Jackson Laboratory that were 6 to 8 weeks old. Mice were kept on a 12:12-h light/dark cycle (lights on at 7:00 am), and received water and food ad libitum at room temperature and 40-60% humidity. All studies used littermate mice of both sex with the same age, unless otherwise specified. 6 to 10 weeks old Emx1-Celf4 females were used for timed pregnancies. We collected prenatal neocortical tissue at embryonic days (E) 11, E13, E15, E17 and postnatal day (P) 0 for immunoflorescence tests, E17 neocortices for RIP-RNAseq experiments, and P0 neocortices for polysome-RNAseq experiments (to yield meaningful replicate, 3 mouse neocortices of the same genotype were pooled together and processed for downstream polysome profiling analyses).

Wild animals

This study did not involve wild animals.

Reporting on sex

Sex was considered in this study, and pups were genotyped by standard protocol from purified tissues (tail, leg). The genomic DNA

Reporting on sex	was isolated from collected tissue, and PCR reactions were made using sex-linked primers (mouse XY forward: 5'- CTG AAG CTT TTG GCT TTG AG -3', mouse XY reverse: 5'- CCA CTG CCA AAT TCT TTG G -3'). To obtain positive control DNA samples of known sexual identity, genomic DNA was extracted from adult males and females identified by conventional visual sexing.<br><br>Sex-based analysis was performed for CX7-quantified synaptic markers (Figure 5. and Supplementary Figure 11.).<br>Only male sample were used in Figure 4. [panels b), e) and f)] and Supplementary figure 9 [panels d) and e)].<br>In other mouse-related experiments, we combined male and female samples to perform the statistical analysis because we did not observe difference between the genotypes (WT vs cKO).<br>Sample size is listed in the figure legends.
Field-collected samples	This study did not involve the samples collected from the field.
Ethics oversight	Animal care and experimental procedures involving animals were performed in accordance with the guidelines established by Rutgers-RWJMS Institutional Animal Care and Use Committee (protocol: I12-065).

Note that full information on the approval of the study protocol must also be provided in the manuscript.

This checklist template is licensed under a Creative Commons Attribution 4.0 International License, which permits use, sharing, adaptation, distribution and reproduction in any medium or format, as long as you give appropriate credit to the original author(s) and the source, provide a link to the Creative Commons license, and indicate if changes were made. The images or other third party material in this article are included in the article's Creative Commons license, unless indicated otherwise in a credit line to the material. If material is not included in the article's Creative Commons license and your intended use is not permitted by statutory regulation or exceeds the permitted use, you will need to obtain permission directly from the copyright holder. To view a copy of this license, visit http://creativecommons.org/licenses/by/4.0/
